# Supplementary material for: Acid Catalyzed Stereocontrolled Ferrier-Type Glycosylation Assisted by Perfluorinated Solvent
Source: Molecules. 2022 Oct 25;27(21):7234. doi: 10.3390/molecules27217234 (PMC9656285; doi:10.3390/molecules27217234)
Supplement: Supplementary file 1 [file molecules-27-07234-s001.zip › molecules-1960760-supplementary.pdf]

Supplementary Materials

Acid Catalyzed Stereocontrolled Ferrier-type Glycosylation Assisted by Perfluorinated Solvent

Zhiqiang Lu [1,2,*,†], Yanzhi Li [1,2,†], Shao-Hua Xiang [3,*], Mengke Zuo [1], Yangxing Sun [2], Xingxing Jiang [1,2], Rongkai Jiao [1], Yinghong Wang [1] and Yuqin Fu [1,*]

1 College of Chemistry and Chemical Engineering and Henan Key Laboratory of Function-Oriented Porous Materials, Luoyang Normal University, Luoyang 471934, China
2 Hubei Key Laboratory of Natural Products Research and Development, Key Laboratory of Functional Yeast (China National Light Industry), College of Biological and Pharmaceutical Sciences, China Three Gorges University, Yichang 443002, China
3 Academy for Advanced Interdisciplinary Studies, Southern University of Science and Technology, Shenzhen 518055, China
* Correspondence: zqlu2000@lynu.edu.cn (Z. L.); xiangsh@sustech.edu.cn (S.-H.X.); lyfyq@lynu.edu.cn (Y. F.)
† These authors contributed equally to this work.

Table of Contents **Page**

Section A: General Information…………………………………………… S1

Section B: General Experimental Procedures……………………………... S2

Section C: Initial NMR Detection for Sub-interaction of Organic Fluorine Investigation………………………………………………………………… S5

Section D: Characterization Data ………………………………………… S11

Section E: NMR Spectra…………………………………………………… S40

Section F: X-ray Crystal Structure Data……………………………......… S106

Section A: General Information

Unless otherwise specified, all reactions of glycosylation were performed in nitrogen conditions. Starting reagents, catalysts, and solvents were all purchased commercially and used as received. Thin layer chromatography (TLC) with a TLC silica gel GF_{254} plate was used to check the reaction progress. UV light was used to visualize compounds on TLC plates under 254 nm. Flash column chromatography was used to isolate products with silica gel (200–300 mesh) and gradient solvent system (petroleum ether, ethyl acetate and dichloromethane mixture as eluent). ^{1}H and ^{13}C NMR spectra were obtained using a 400 MHz Bruker Avance 400 spectrometer. All ^{1}H-NMR chemical shifts were recorded relative to the TMS peak at 0.00 ppm or the solvent residual peak ($CDCl_3$ at 7.26 ppm). ^{13}C NMR chemical shifts are reported relative to the TMS peak at 0.0 ppm or the solvent residual peak ($CDCl_3$ at 77.16 ppm). Multiplicities were reported as s (singlet), d (doublet), t (triplet), q (quartet), quin (quintuplet), sext (sextet), sep (septet), m (multiplet or unsolved), brs (broad singlet), dd (doublet of doublets), doublet of triplets (dt), triplet of doublets (td) or doublet of doublet of doublets (ddd). The number of protons (n) corresponding to a resonance signal was indicated by n H and Spin-spin coupling constants (*J* value) recorded in Hz.

Section B: General Experimental Procedures

The synthesis procedure of the sugar donor series

Take the synthesis of compound glucal (tri-*O*-acetyl D-glycal) (**1a**) as an example: D-glucose (5.0 g, 27.75 mmol) and acetic anhydride 25 mL were added to a 250 mL round bottom flask. The reaction temperature was lowered to 0 °C, 3 drops of perchloric acid were added to the round bottom flask, and the mixture was slowly warmed to room temperature for 20 minutes. After TLC was used to monitor the reaction of the starting material, the reaction mixture was poured into 20 mL of ice water and ethyl acetate 50 mL, using a separating funnel to separate the mixture. The organic phase was adjusted to a neutral pH with a saturated sodium bicarbonate solution, and the aqueous phase was extracted three times with 50 mL ethyl acetate. The organic phase was combined and dried with anhydrous sodium sulfate, concentrated under reduced. We dissolved the previous product in dichloromethane; 20 mL of hydrogen bromide-acetic acid solution (33% *w*/*w*) was slowly added dropwise under ice-cold bath cooling conditions. After the dropwise addition, the reaction solution was slowly raised to room temperature and reacted for 2 hours. After the reaction was completed, the reaction mixture was poured into 20 mL of ice water and dichloromethane 50 mL, using a separating funnel to separate the mixture. The organic phase was adjusted to a neutral pH with a saturated sodium bicarbonate solution, and the aqueous phase was extracted three times with 50 mL dichloromethane. The organic phase was combined and dried with anhydrous sodium sulfate, concentrated under reduced. We dissolved the previous product in ethyl acetate, 50 mL saturated sodium dihydrogen phosphate solution, and 32 g zinc powder was added and reacted for 2 h at room temperature. After the TLC was used to monitor the reaction of the starting material, the mixture was filtered through celite, and the filtrate was extracted with ethyl acetate (5 × 50 mL). The organic layer was combined and adjusted to a neutral pH with a saturated sodium bicarbonate solution, and the aqueous phase was extracted three times with 50 mL ethyl acetate. The organic phase was combined and dried with anhydrous sodium sulfate, concentrated, and purified by chromatography column with ethyl acetate. All other glycals (**1b**-**1f**) were obtained following a similar procedure.

General procedure for preparation of *O*-glycosides

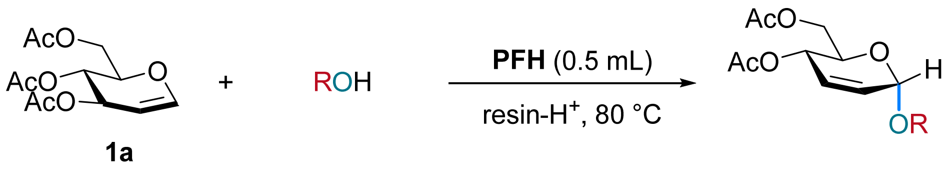

To a mixture of tri-*O*-acetyl-D-glucal (0.138 mmol), cation exchange resin (sulfonic polystyrene type resin) (0.2 mg), nucleophile (0.166 mmol) were added to 0.5 mL perfluorohexane under nitrogen atmosphere. The reaction mixture was stirred at 80 °C and monitored by TLC until the glycal donor was consumed completely. The reaction mixture was removed to afford a crude product which was purified by flash chromatography with a gradient solvent system (petroleum ether/ethyl acetate or dichloromethane as eluent).

The additional substrate of glycosyl donor without C5 substituent experiment

Due to the extensive procedure, xylal and arabinal, the substrate without C5 substituents such as xylal and arabinal were also utilized following this general procedure, and all the results indicated that the direct significance of C-5 substitution in stereoinduction (**Scheme S1.**).

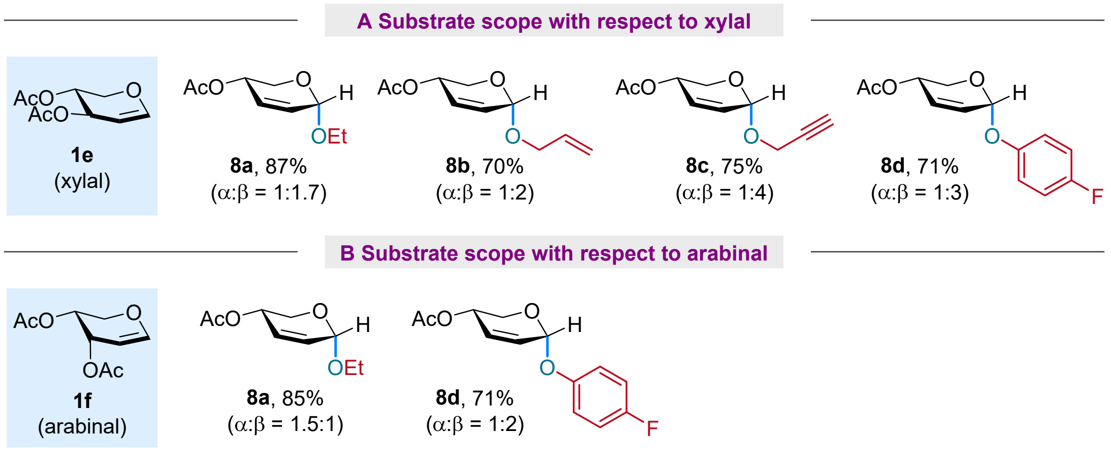

Scheme S1. Substrate generality with respect to glycosyl donors without C5 substituent. A: Subatrate scope with respect to xylal; B: Substrate scope with respect to arabinal.

The reaction is scaled up to gram and the yield is up to 95%

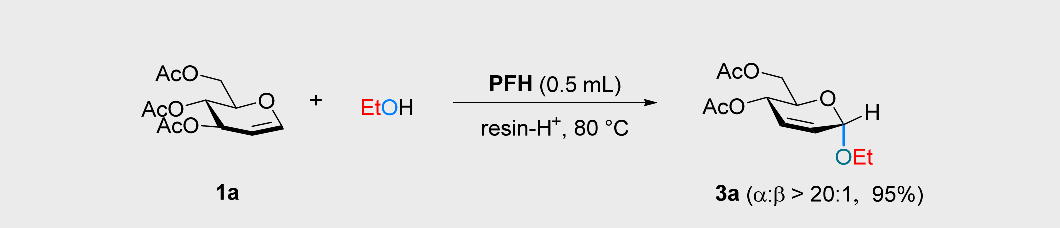

Scheme S2. Scaled-up experiment.

To demonstrate the synthetic value of this work, we first attempted the transformation of **1a** with EtOH on a 3.68 mmol tri-*O*-acetyl-D-glucal with 2 mL PFH loading, which afforded the *O*-glycoside **3a** in 95% yield and high *α*-stereocontrol.

The specific steps of phase separation

We ran the reaction as the general procedure for finishing each cyclic reaction, then kept the reaction mixture until the product could be checked at the upper layer of the reaction system to room temperature. At this point, add the appropriate amount of ethyl acetate (EA) into the reaction system, the products-containing phase should be in the upper organic layer of EA, and the lower layer should be the perfluorohexane phase. Then, the upper layer was decanted out carefully, and the resin-H^+ was still left in the reaction system. The crude product can be obtained until the organic solvent is removed under reduced pressure. Meanwhile, the sugar donor and sugar acceptor as the renewal starting material were added into the residue lower layer solvent. Due to the resin-H^+ being still here, no renewal resin-H^+ was needed anymore, then run the mixture for the next round of reaction. As mentioned above, this procedure can be recyclable for up to 7 rounds, and we cannot find obvious differences in reaction efficiency.

Section C: Initial NMR Detection for Sub-interaction of Organic Fluorine Investigation

In order to investigate the organic fluorine sub-interaction in this reaction, a series of experiments were designed. Perfluorohexane was employed for the investigation of the solvent effect at different temperatures (25 °C, 40 °C, and 60 °C, respectively). The results also showed a broad peak of hydroxy and other discrepancies of proton in DMSO-d_6 compared with and without perfluorohexane. (**Scheme S3-1~6**)

From the above, the changes in the chemical shifts of representative NMR signals could be noted. While these observations could not constitute definitive evidence, they may add credence to our hypothesis that these weak interactions have enhanced the promotion of present chemistry.

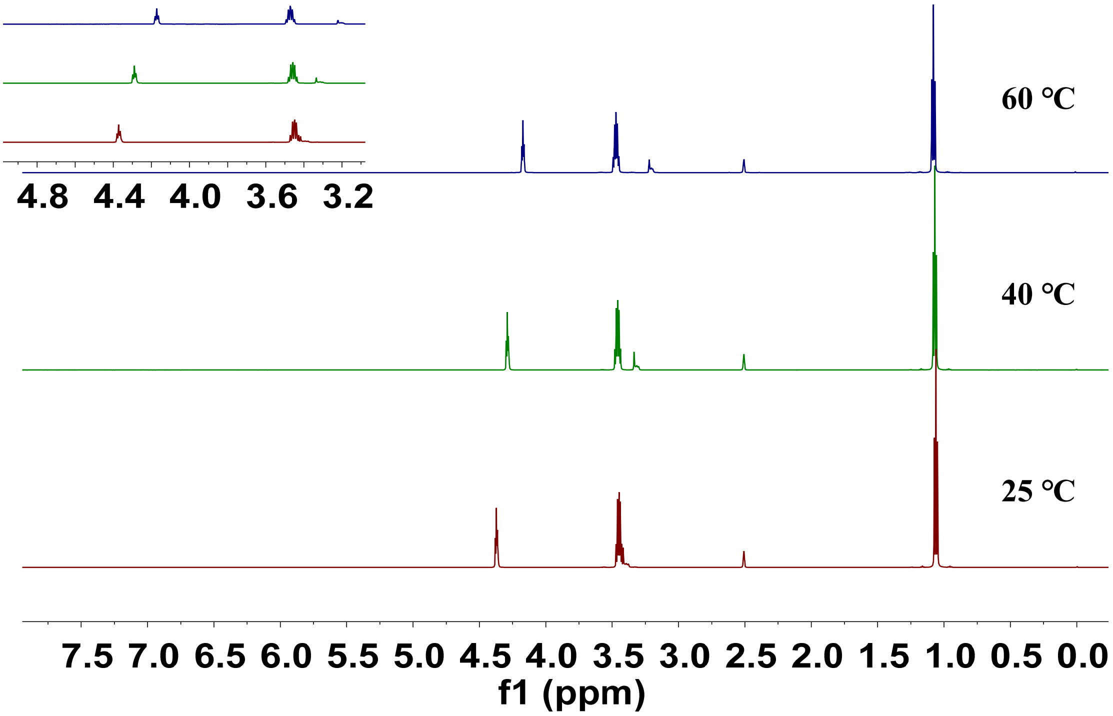

Scheme S3-1. The ^{1}H-NMR determination for ethanol (0.05 mL) as the sugar acceptor in DMSO-d_6 (0.5 mL) at different temperatures (from the bottom to upper: 25 °C, 40 °C, and 60 °C, respectively).

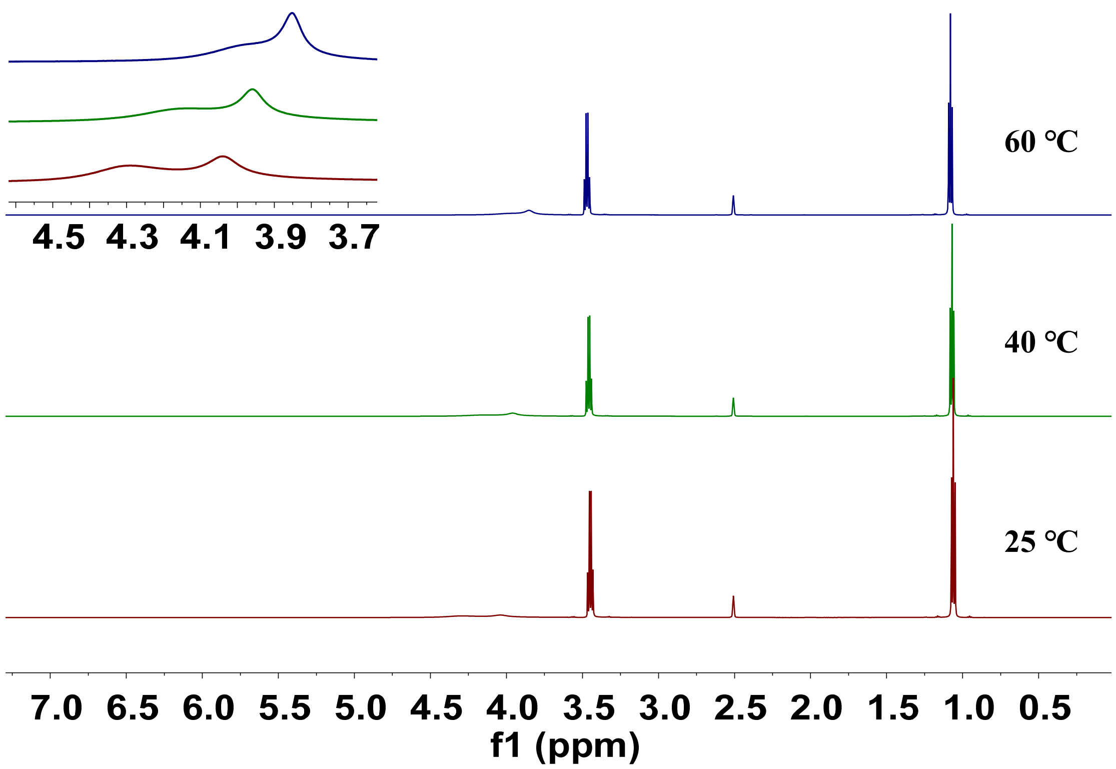

Scheme S3-2. The ^{1}H-NMR determination for the mixture of ethanol (0.05 mL) and perfluorohexane (0.05 mL) in DMSO-d_6 (0.45 mL) at different temperatures (from the bottom to upper: 25 °C, 40 °C, and 60 °C respectively). All the results indicate that the hydroxyl proton is transferred to a broad one compared with the ethanol at the corresponding temperature.

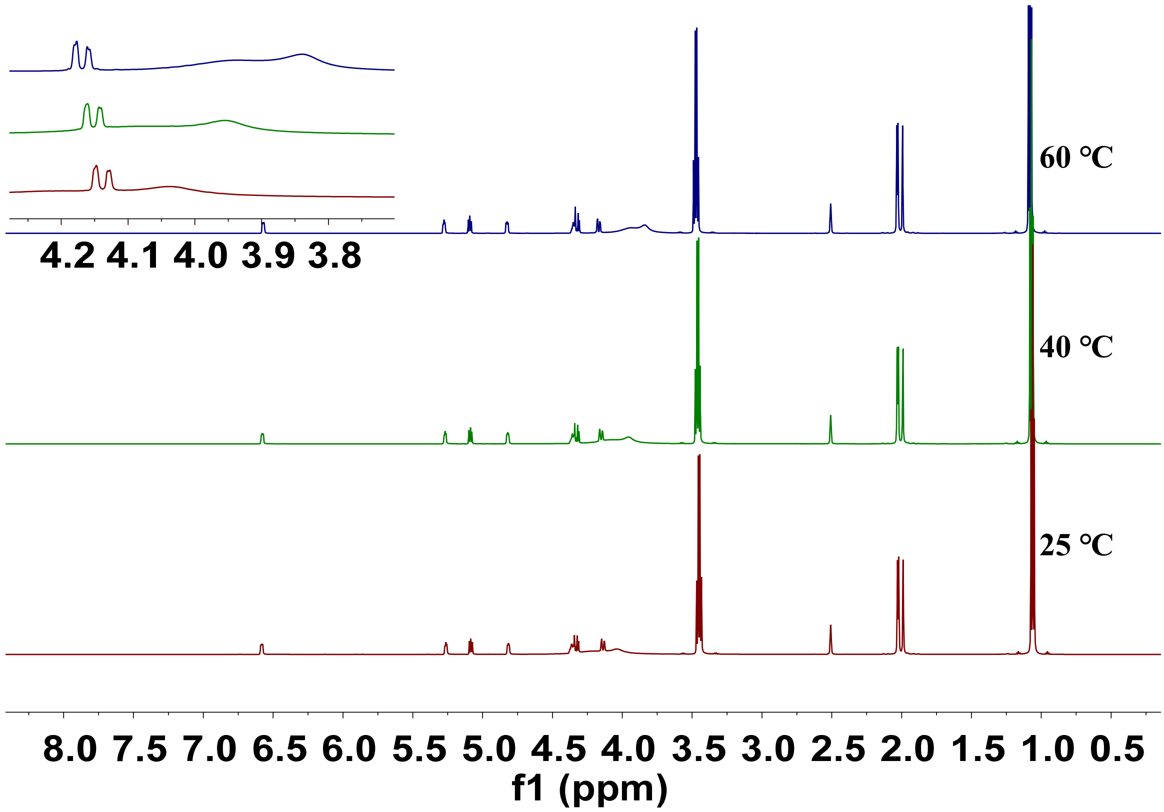

Scheme S3-3. The ^{1}H-NMR determination for mixture of ethanol (0.05 mL) and preacetylglycal (30 mg) with perfluorohexane (0.05 mL) in DMSO-d_6 (0.45 mL) at different temperatures (from the bottom to upper: 25 °C, 40 °C, and 60 °C respectively). All the results indicate that the hydroxyl proton is transferred to a broad one compared with the ethanol at the corresponding temperature.

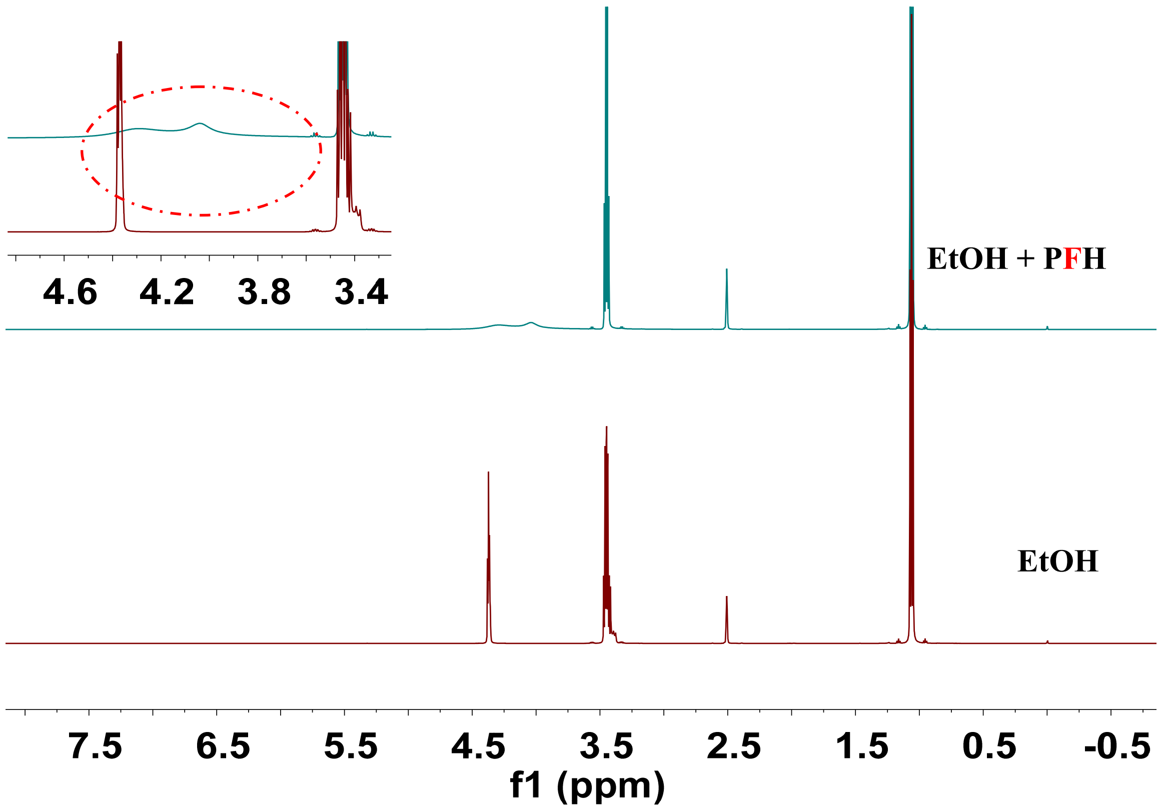

Scheme S3-4. The ^{1}H-NMR determination for ethanol (0.05 mL) (bottom) in DMSO-d_6 (0.5 mL) and ethanol (0.05 mL)-perfluorohexane (0.05 mL) mixture (upper) in the DMSO-d_6 (0.45 mL) at 25 ºC from the bottom to upper respectively. All the results clearly indicate that the hydroxyl proton is transferred to a broad one compared with the ethanol itself.

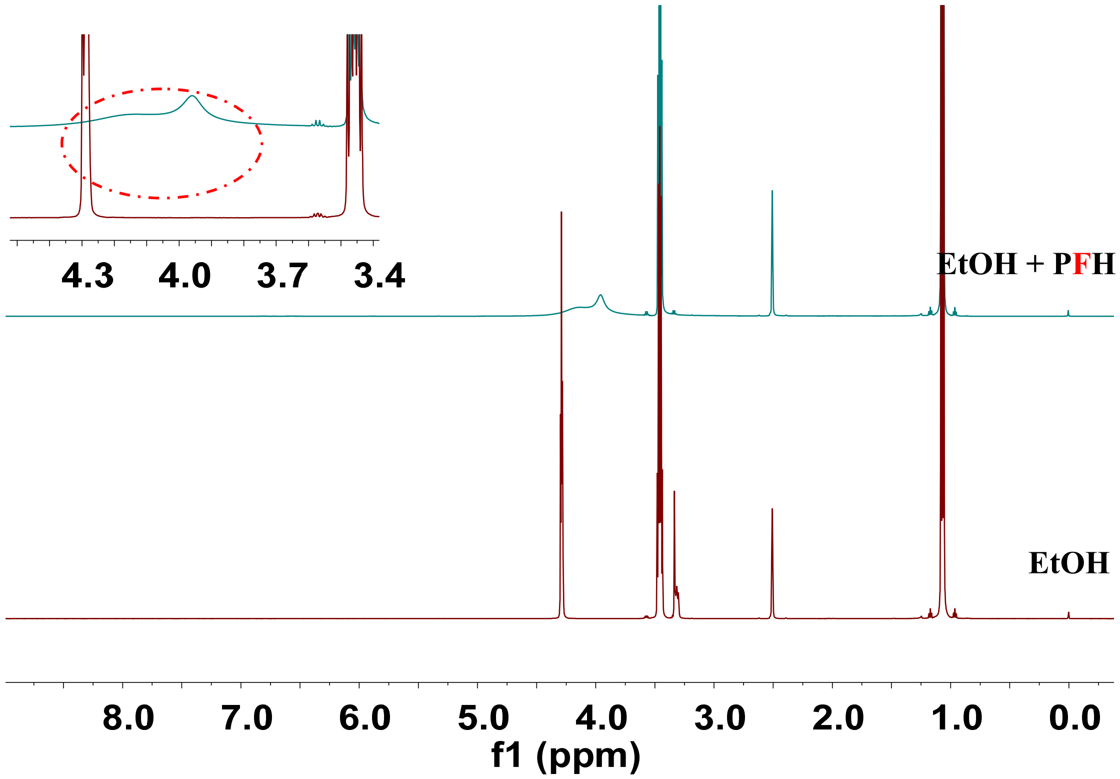

Scheme S3-5. The ^{1}H-NMR determination for ethanol (0.05 mL) (bottom) in DMSO-d_6 (0.5 mL) and ethanol (0.05 mL)-perfluorohexane (0.05 mL) mixture (upper) in the DMSO-d_6 (0.5 mL) at 40 °C from bottom to upper respectively. The results indicate that the hydroxyl's proton is transferred to a broad one compared with the ethanol itself.

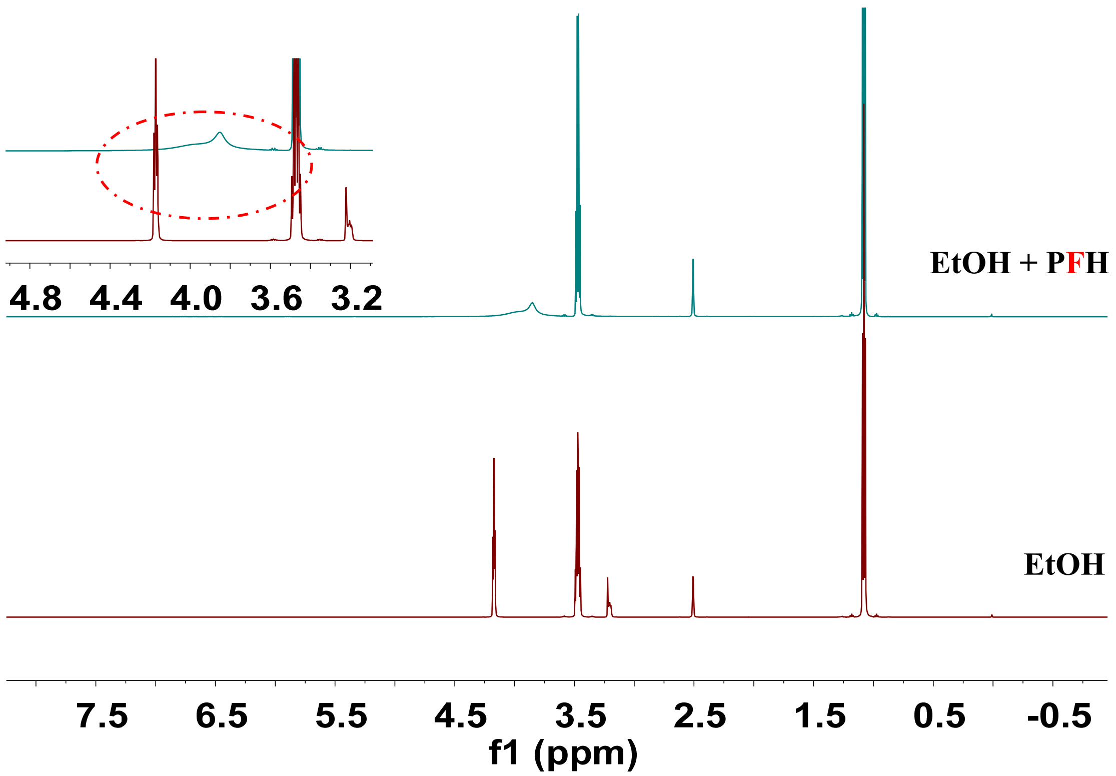

Scheme S3-6. The ^{1}H-NMR determination for ethanol (0.05 mL) (bottom) in DMSO-d_6 (0.5 mL) and ethanol (0.05 mL)-perfluorohexane (0.05 mL) mixture (upper) in DMSO-d_6 (0.45 mL) at 60 ºC from the bottom to upper respectively. The results indicate that the hydroxyl's proton is transferred to a broad one compared with the ethanol itself.

Section D: Characterization Data

((2*R*,3*S*,6*S*)-3-acetoxy-6-ethoxy-3,6-dihydro-2*H*-pyran-2-yl)methyl acetate (3a)

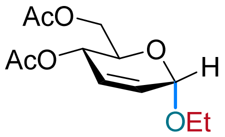

Colorless crystal, yield 95%; m. p.: 64.6-65.1 °C; ^{1}H NMR (400 MHz, Chloroform-*d*) δ 5.94–5.79 (m, 2H), 5.36–5.29 (m, 1H), 5.05 (s, 1H), 4.26 (dd, *J* = 12.1, 5.3 Hz, 1H), 4.18 (dd, *J* = 12.1, 2.4 Hz, 1H), 4.11 (ddt, *J* = 11.6, 9.2, 4.5 Hz, 1H), 3.84 (dq, *J* = 9.7, 7.1 Hz, 1H), 3.58 (dq, *J* = 9.6, 7.1 Hz, 1H), 2.10 (s, 3H), 2.08 (s, 3H), 1.25 (t, *J* = 7.5 Hz, 3H). ^{13}C NMR (100 MHz, Chloroform-*d*) δ 171.0, 170.5, 129.1, 128.1, 94.4, 66.9, 65.4, 64.4, 63.1, 21.1, 20.9, 15.4. HRMS (ESI) *m/z*: calcd. for $C_{12}H_{18}O_6$ Na^+ $(M + Na)^+$ 281.0996, found 281.0935; $[\alpha]_D^{20}$ = 34.793 (c = 0.428, $CHCl_3$).

((2*R*,3*S*,6*S*)-3-acetoxy-6-methoxy-3,6-dihydro-2*H*-pyran-2-yl)methyl acetate (3b) [48]

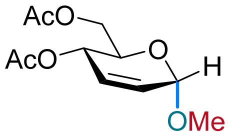

Colorless oil, yield 90%; ^{1}H NMR (400 MHz, Chloroform-*d*) δ 5.93–5.79 (m, 2H), 5.32 (dq, *J* = 9.7, 1.60 Hz, 1H), 4.93 (d, *J* = 2.2 Hz, 1H), 4.26 (ddd, *J* = 12.1, 5.3, 1.4 Hz, 1H), 4.18 (dt, *J* = 12.1, 1.9 Hz, 1H), 4.08 (ddt, *J* = 9.8, 5.2, 2.4 Hz, 1H), 3.45 (d, *J* = 1.4Hz, 3H), 2.11 (d, *J* = 1.5Hz, 3H), 2.08 (d, *J* = 1.4Hz, 3H). ^{13}C NMR (100 MHz, Chloroform-*d*) δ 171.0, 170.5, 129.4, 127.8, 95.6, 67.0, 65.4, 63.1, 56.1, 21.1, 21.0. HRMS (ESI) *m/z*: calcd. for $C_{11}H_{16}O_6$ Na^+ $(M + Na)^+$ 267.0839, found 267.0812; $[\alpha]_D^{13}$ = 73.946 (c = 0.212, MeOH).

((2*R*,3*S*,6*S*)-3-acetoxy-6-butoxy-3,6-dihydro-2*H*-pyran-2-yl)methyl acetate (3c) [49]

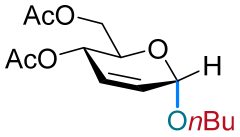

Colourless oil; yield 91%; ^{1}H NMR (400 MHz, Chloroform-*d*) δ 5.91–5.75 (m, 2H),

5.29 (dq, J = 9.7, 1.5 Hz, 1H), 5.06–4.97 (m, 1H), 4.23 (dd, J = 12.0, 5.4 Hz, 1H), 4.16 (dd, J = 12.1, 2.4 Hz, 1H), 4.09 (ddd, J = 9.7, 5.4, 2.4 Hz, 1H), 3.77 (dt, J = 9.5, 6.7 Hz, 1H), 3.50 (dt, J = 9.5, 6.3 Hz, 1H), 2.09 (s, 3H), 2.07 (s, 3H), 1.64–1.51 (m, 2H), 1.38 (dddd, J = 9.5, 7.2, 5.3, 2.2 Hz, 2H), 0.91 (t, J = 7.4 Hz, 3H). ^{13}C NMR (100 MHz, Chloroform-*d*) δ 170.9, 170.4, 129.1, 128.1, 94.5, 68.8, 67.0, 65.4, 63.2, 31.9, 21.1, 20.9, 19.5, 14.0. HRMS (ESI) *m/z*: calcd. for $C_{14}H_{22}O_6$ Na^+ $(M + Na)^+$ 309.1309, found 309.1310; $[\alpha]_D^{20}$ = 83.496 (c = 0.510, MeOH).

((2*R*,3*S*,6*S*)-3-acetoxy-6-isopropoxy-3,6-dihydro-2*H*-pyran-2-yl)methyl acetate (3d) [49]

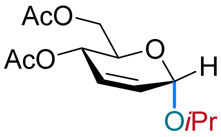

Colorless oil; yield 92%; m. p.: 64.6-65.1 °C; ^{1}H NMR (400 MHz, Chloroform-*d*) δ 5.91–5.76 (m, 2H), 5.33–5.24 (m, 1H), 5.12 (s, 1H), 4.23 (dd, J = 12.2, 5.7 Hz, 1H), 4.18 (d, J = 2.3 Hz, 1H), 4.14 (ddt, J = 7.9, 5.5, 2.4 Hz, 1H), 3.97 (dt, J = 12.4, 6.2 Hz, 1H), 2.08 (s, 3H), 2.07 (s, 3H), 1.24 (d, J = 6.2 Hz, 3H), 1.17 (d, J = 6.2 Hz, 3H). ^{13}C NMR (100 MHz, Chloroform-*d*) δ 170.9, 170.5, 128.9, 128.6, 93.0, 70.9, 66.9, 65.6, 63.3, 23.6, 22.1, 21.1, 20.9. HRMS (ESI) *m/z*: calcd. for $C_{13}H_{20}O_6$ Na^+ $(M + Na)^+$ 295.1152, found 295.1109; $[\alpha]_D^{18}$ = 72.141 (c = 0.354, MeOH).

((2*R*,3*S*,6*S*)-3-acetoxy-6-(cyclohexyloxy)-3,6-dihydro-2*H*-pyran-2-yl)methyl acetate (3e) [49]

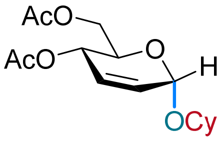

Colorless oil; yield 91%; m. p.: 40.1-41.3 °C; ^{1}H NMR (400 MHz, Chloroform-*d*) δ 5.89–5.75 (m, 2H), 5.28 (d, J = 9.4 Hz, 1H), 5.15 (s, 1H), 4.17 (ddt, J = 14.8, 9.3, 5.3 Hz, 3H), 3.63 (tt, J = 9.4, 4.1 Hz, 1H), 2.07 (s, 3H), 2.06 (s, 3H), 1.97–1.85 (m, 2H), 1.28 (ddt, J = 48.0, 19.8, 11.2 Hz, 8H). ^{13}C NMR (100 MHz, Chloroform-*d*) δ 170.9, 170.4, 128.8, 128.6, 92.9, 66.8, 65.5, 63.2, 33.9, 32.2, 25.6, 24.5, 24.3, 21.1, 20.9. HRMS (ESI) *m/z*: calcd. for $C_{16}H_{24}O_6$ Na^+ $(M + Na)^+$ 335.1465, found 335.1450;

$[\alpha]_D^{32}$ = 72.094 (c = 0.246, $CHCl_3$).

((2*R*,3*S*,6*R*)-3-acetoxy-6-(tert-butoxy)-3,6-dihydro-2*H*-pyran-2-yl)methyl acetate (3f) [50]

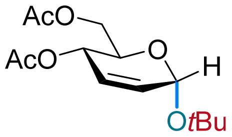

Colorless oil; yield 91%; ^{1}H NMR (400 MHz, Chloroform-*d*) δ 5.83 (d, *J* = 10.0 Hz, 1H), 5.73(dt, *J* = 10.2, 2.3 Hz, 1H), 5.31 (s, 1H), 5.26 (d, *J* = 9.2 Hz, 1H), 4.26–4.20 (m, 1H), 4.20–4.15(m, 1H), 4.15–4.10(m, 1H), 2.07 (s, 3H), 2.06 (s, 3H), 1.28 (s, 9H). ^{13}C NMR (100 MHz, Chloroform-*d*) δ 171.0, 170.52, 129.7, 128.3, 89.1, 77.5, 66.6, 65.4, 63.4, 28.9, 21.1, 21.0. HRMS (ESI) *m/z*: calcd. for $C_{14}H_{22}O_6$ NH_4^+ $(M + NH_4)^+$ 304.1755, found 304.1753; $[\alpha]_D^{18}$ = 70.321 (c = 0.400, MeOH).

((2*R*,3*S*,6*S*)-3-acetoxy-6-(2,2,2-trifluoroethoxy)-3,6-dihydro-2*H*-pyran-2-yl)methyl acetate (3g) [51]

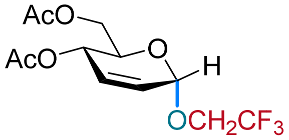

Yellowish oil; yield 94%; ^{1}H NMR (400 MHz, Chloroform-*d*) δ 5.95 (d, *J* = 10.3 Hz, 1H), 5.85 (dt, *J* = 10.2, 2.3 Hz, 1H), 5.37–5.28 (m, 1H), 5.11 (s, 1H), 4.26–4.15 (m, 2H), 4.13–4.07 (m, 1H), 4.07–4.00 (m, 1H), 3.99–3.91(m, 1H), 2.09 (s, 6H). ^{13}C NMR (100 MHz, Chloroform-*d*) δ 170.8, 170.4, 130.6, 126.4, 94.7, 67.6, 65.3, 65.0, 65.0, 62.7, 21.1, 20.9. HRMS (ESI) *m/z*: calcd. for $C_{12}H_{15}F_3O_6$ Na^+ $(M + Na)^+$ 335.0713, found 335.0727; $[\alpha]_D^{13}$ = 113.930 (c = 0.440, MeOH).

((2*R*,3*S*,6*S*)-3-acetoxy-6-(octyloxy)-3,6-dihydro-2*H*-pyran-2-yl)methyl acetate (3h) [49]

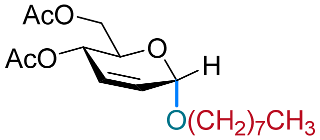

Colourless oil; yield 94%; ^{1}H NMR (400 MHz, Chloroform-*d*) δ 5.91–5.79 (m, 2H),

5.37–5.25 (m, 1H), 5.01 (s, 1H), 4.24 (dd, *J* = 12.1, 5.3 Hz, 1H), 4.16 (dd, *J* = 12.1, 2.2 Hz, 1H), 4.09 (ddd, *J* = 9.4, 5.2, 2.2 Hz, 1H), 3.75 (dt, *J* = 9.4, 6.8 Hz, 1H), 3.49 (dt, *J* = 9.5, 6.5 Hz, 1H), 2.09 (s, 3H), 2.07 (s, 3H), 1.63–1.57 (m, 2H), 1.27 (s, 10H), 0.86 (t, *J* = 6.8 Hz, 3H). ^{13}C NMR (100 MHz, Chloroform-*d*) δ 170.9, 170.4, 129.1, 128.1, 94.5, 69.1, 66.9, 65.4, 63.1, 31.9, 29.8, 29.5, 29.4, 26.4, 22.8, 21.1, 20.9, 14.2. HRMS (ESI) *m/z*: calcd. for $C_{18}H_{30}O_6$ Na^+ $(M + Na)^+$ 365.1934, found 365.1911; $[\alpha]_D^{16}$ = 85.074 (c = 0.336, DCM).

((2*R*,3*S*,6*S*)-3-acetoxy-6-(2-methoxyethoxy)-3,6-dihydro-2*H*-pyran-2-yl)methyl acetate (3i) [52]

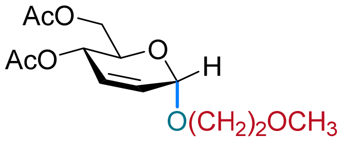

Colourless oil; yield 85%; ^{1}H NMR (400 MHz, Chloroform-*d*) δ 5.87 (d, *J* = 1.4 Hz, 2H), 5.31 (d, *J* = 9.7 Hz, 1H), 5.07 (s, 1H), 4.24 (dd, *J* = 12.1, 5.3 Hz, 1H), 4.18(d, *J* = 2.4 Hz, 1H), 4.12 (ddt, *J* = 12.0, 5.2, 2.4 Hz, 1H), 3.92–3.86 (m, 1H), 3.71–3.65 (m, 1H), 3.57 (dd, *J* = 5.7, 3.8 Hz, 2H), 3.38 (s, 3H), 2.09 (s, 3H), 2.07 (s, 3H). ^{13}C NMR (100 MHz, Chloroform-*d*) δ 170.9, 170.4, 129.3, 127.8, 94.7, 71.8, 67.8, 67.0, 65.3, 63.0, 59.2, 21.1, 20.9. HRMS (ESI) *m/z*: calcd. for $C_{13}H_{20}O_7$ Na^+ $(M + Na)^+$ 311.1101, found 311.1134; $[\alpha]_D^{19}$ = 49.962 (c = 0.310, MeOH).

((2*R*,3*S*,6*R*)-3-acetoxy-6-(tert-pentyloxy)-3,6-dihydro-2*H*-pyran-2-yl)methyl acetate (3j) [50]

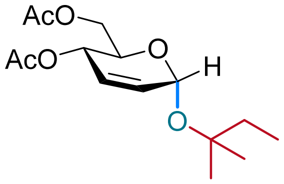

Colorless oil; yield 94%; ^{1}H NMR (400 MHz, Chloroform-*d*) δ 5.92–5.67 (m, 2H), 5.36–5.21 (m, 2H), 4.29–4.24 (m, 1H), 4.24–4.20 (m, 1H), 4.20–4.15 (m, 1H), 2.11 (s, 3H), 2.10 (s, 3H), 1.60 (d, *J* = 6.7 Hz, 2H), 1.27 (d, *J* = 3.1 Hz, 6H), 0.93 (d, *J* = 7.4 Hz, 3H). ^{13}C NMR (100 MHz, Chloroform-*d*) δ 171.0, 170.5, 129.7, 128.2, 88.9, 77.7, 66.8, 65.4, 63.4, 34.3, 26.3, 26.1, 21.2, 21.0, 8.6. HRMS (ESI) *m/z*: calcd. for $C_{15}H_{24}O_6$ Na^+ $(M + Na)^+$ 323.1465, found 323.1485; $[\alpha]_D^{19}$ = 49.669 (c = 0.272,

MeOH).

((2*R*,3*S*,6*S*)-3-acetoxy-6-(allyloxy)-3,6-dihydro-2*H*-pyran-2-yl)methyl acetate (3k) [53]

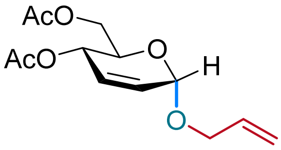

White solid; yield 89%; m. p.: 36.5-37.9 °C; ^{1}H NMR (400 MHz, Chloroform-*d*) δ 6.02–5.92 (m, 1H), 5.92–5.81 (m, 2H), 5.36–5.26 (m, 2H), 5.21 (d, *J* = 10.4 Hz, 1H), 5.08 (s, 1H), 4.28 (d, *J* = 5.1Hz, 1H), 4.26–4.22 (m, 1H), 4.22–4.15(m, 1H), 4.15–4.04 (m, 2H), 2.10 (s, 3H), 2.08 (s, 3H). ^{13}C NMR (100 MHz, Chloroform-*d*) δ 170.9, 170.4, 134.2, 129.4, 127.9, 117.7, 93.8, 69.4, 67.1, 65.4, 63.1, 21.1, 20.9. HRMS (ESI) *m/z*: calcd. for $C_{13}H_{18}O_6$ Na^+ $(M + Na)^+$ 293.0996, found 293.0986; $[\alpha]_D^{21}$ = 89.893 (c = 0.172, MeOH).

((2*R*,3*S*)-3-acetoxy-6-(benzyloxy)-3,6-dihydro-2*H*-pyran-2-yl)methyl acetate (3l) [53]

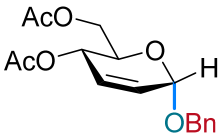

Colorless crystal; yield 89%; m. p.: 40.6-41.7 °C; ^{1}H NMR (400 MHz, Chloroform-*d*) δ 7.36 (d, *J* = 4.4 Hz, 4H), 7.34–7.28 (m, 1H), 5.93–5.82 (m, 2H), 5.37–5.30 (m, 1H), 5.14 (s, 1H), 4.81 (d, *J* = 11.6 Hz, 1H), 4.60 (d, *J* = 11.7 Hz, 1H), 4.25 (dd, *J* = 11.7, 5.0 Hz, 1H), 4.20–4.14 (m, 1H), 4.14–4.08(m, 1H), 2.10 (s, 3H), 2.08 (s, 3H). ^{13}C NMR (100 MHz, Chloroform-*d*) δ 170.9, 170.4, 137.7, 129.4, 128.6, 128.2, 128.0, 127.8, 93.8, 70.4, 67.2, 65.4, 63.0, 21.1, 21.0. HRMS (ESI) *m/z*: calcd. for $C_{17}H_{20}O_6$ Na^+ $(M + Na)^+$ 343.1152, found 343.1143; $[\alpha]_D^{14}$ = 58.781 (c = 0.346, MeOH).

((2*R*,3*S*)-3-acetoxy-6-(benzhydryloxy)-3,6-dihydro-2*H*-pyran-2-yl)methyl acetate (3m) [54]

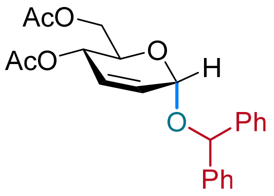

Colorless oil; yield 85%; ^{1}H NMR (400 MHz, Chloroform-*d*) δ 7.41–7.27 (m, 10H), 5.91 (d, *J* = 10.4 Hz, 1H), 5.87(d, *J* = 7.5 Hz, 2H), 5.33(d, *J* = 9.3 Hz, 1H), 5.14(d, *J* = 5.8Hz, 1H), 4.27–4.13 (m, 2H), 4.01 (d, *J* = 10.7 Hz, 1H), 2.08 (s, 3H), 2.06 (s, 3H). ^{13}C NMR (100 MHz, Chloroform-*d*) δ 170.9, 170.4, 142.4, 141.0, 129.5, 128.7, 128.4, 128.0, 128.0, 127.6, 127.2, 92.7, 80.7, 67.5, 65.5, 63.0, 21.1, 20.9. HRMS (ESI) *m/z*: calcd. for $C_{23}H_{24}O_6$ Na^+ $(M + Na)^+$ 419.1465, found 419.1462; $[\alpha]_D^{20}$ = 78.769 (c = 0.218, MeOH).

((2*R*,3*S*,6*S*)-3-acetoxy-6-(prop-2-yn-1-yloxy)-3,6-dihydro-2*H*-pyran-2-yl)methyl acetate (3n) [49]

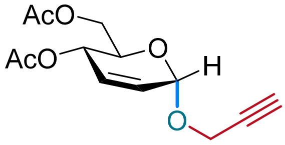

White solid; yield 91%; m. p.: 55.6-56.7 °C; ^{1}H NMR (400 MHz, Chloroform-*d*) δ 5.92 (d, *J* = 10.3 Hz, 1H), 5.84 (d, *J* = 10.7 Hz, 1H), 5.34 (d, *J* = 9.6 Hz, 1H), 5.24 (s, 1H), 4.33–4.29 (m, 2H), 4.25 (dd, *J* = 12.2, 5.1 Hz, 1H), 4.17 (d, *J* = 12.0 Hz, 1H), 4.13–4.05 (m, 1H), 2.49–2.44 (m, 1H), 2.10 (s, 3H), 2.08 (s, 3H). ^{13}C NMR (100 MHz, Chloroform-*d*) δ 171.0, 170.4, 129.9, 127.3, 92.9, 79.2, 75.0, 67.2, 65.2, 62.9, 55.2, 21.1, 21.0. HRMS (ESI) *m/z*: calcd. for $C_{13}H_{16}O_6$ Na^+ $(M + Na)^+$ 291.0839, found 291.0876; $[\alpha]_D^{8}$ = 111.970 (c = 0.304, DCM).

((2*R*,3*S*)-3-acetoxy-6-(((1*R*,3*R*,5*R*,7*R*)-adamantan-2-yl)oxy)-3,6-dihydro-2*H*-pyran-2-yl)methyl acetate (3o)

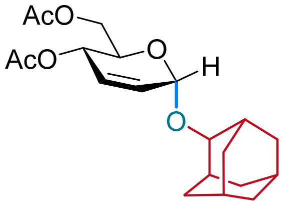

Colorless crystal; yield 87%; m. p.: 67.8-70.6 °C; ^{1}H NMR (400 MHz, Chloroform-*d*) δ 5.97–5.78 (m, 2H), 5.35–5.23 (m, 1H), 5.16 (d, *J* = 2.0 Hz, 1H), 4.30–4.18(m, 1H), 4.18–4.15(m,1H), 4.15–4.09(m, 1H), 3.81 (d, *J* = 3.6 Hz, 1H), 2.07(s, 3H), 2.06 (s, 3H), 2.05–1.93 (m, 6H), 1.84–1.75 (m, 4H), 1.65 (d, *J* = 13.5 Hz, 2H), 1.48 (t, *J* = 9.1 Hz, 2H). ^{13}C NMR (100 MHz, Chloroform-*d*) δ 170.9, 170.4, 129.0, 128.5, 93.3, 81.4, 67.0, 65.6, 63.3, 37.6, 36.8, 36.6, 33.7, 32.0, 31.9, 31.6, 27.5, 27.3, 21.1, 20.9. HRMS (ESI) *m/z*: calcd. for $C_{20}H_{28}O_6$ Na^+ $(M + Na)^+$ 387.1778, found 387.1789; $[\alpha]_D^{19}$ = 74.077 (c = 0.748, MeOH).

((2R,3S,6R)-3-acetoxy-6-phenoxy-3,6-dihydro-2H-pyran-2-yl)methyl acetate (3p) [55]

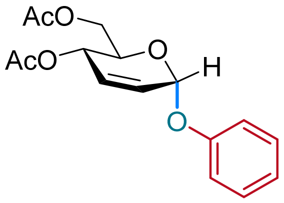

White solid; yield 77%; m. p.: 40.5-41.3 °C; ^{1}H NMR (400 MHz, Chloroform-*d*) δ 7.30 (dd, *J* = 8.5, 7.5 Hz, 2H), 7.10 (d, *J* = 7.8 Hz, 2H), 7.03 (t, *J* = 7.3 Hz, 1H), 6.02 (d, *J* = 3.0 Hz, 2H), 5.70 (s, 1H), 5.39 (d, *J* = 9.4 Hz, 1H), 4.33–4.21 (m, 2H), 4.13 (dd, *J* = 11.6, 1.7 Hz, 1H), 2.10 (s, 3H), 1.97 (s, 3H). ^{13}C NMR (100 MHz, Chloroform-*d*) δ 170.8, 170.3, 157.1, 130.2, 129.5, 127.2, 122.5, 117.1, 93.0, 67.8, 65.1, 62.7, 21.1, 20.8. HRMS (ESI) *m/z*: calcd. for $C_{16}H_{18}O_6$ Na^+ $(M + Na)^+$ 329.0996, found 329.0997; $[\alpha]_D^{21}$ = 490.590 (c = 0.152, MeOH).

((2*R*,3*S*)-3-acetoxy-6-(3,4-dimethylphenoxy)-3,6-dihydro-2*H*-pyran-2-yl)methyl acetate (3q) [55]

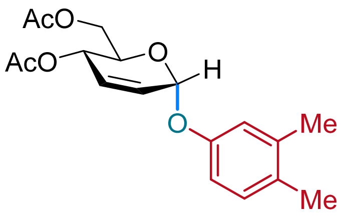

White solid; yield 76%; m. p.: 98.5-101.0 °C; ^{1}H NMR (400 MHz, Chloroform-*d*) δ 7.04 (d, *J* = 8.2 Hz, 1H), 6.90 (d, *J* = 2.5Hz,1H), 6.85 (dd, *J* = 8.2, 2.7Hz, 1H), 6.00 (s, 2H), 5.64 (s, 1H), 5.38 (d, *J* = 9.4 Hz, 1H), 4.30 (dd, *J* = 11.6, 5.5Hz, 1H), 4.27–4.22

(m, 1H), 4.13 (dd, J = 11.7, 1.9 Hz, 1H), 2.24 (s, 3H), 2.20 (s, 3H), 2.11 (s, 3H), 2.01 (s, 3H). ^{13}C NMR (100 MHz, Chloroform-*d*) δ 170.9, 170.4, 155.4, 137.9, 130.8, 130.4, 130.0, 127.4, 118.7, 114.5, 93.5, 67.8, 65.2, 62.9, 21.1, 20.9, 20.1, 19.0. HRMS (ESI) *m/z*: calcd. for $C_{18}H_{22}O_6$ Na^+ $(M + Na)^+$ 357.1309, found 357.1302; $[\alpha]_D^{19}$ = 123.740 (c = 0.300, MeOH).

((2*R*,3*S*)-3-acetoxy-6-(3,5-dimethylphenoxy)-3,6-dihydro-2*H*-pyran-2-yl)methyl acetate (3r)

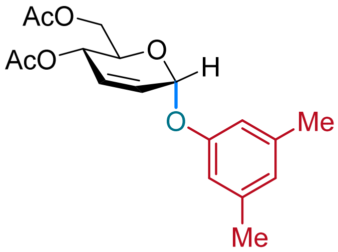

White solid; yield 62%; m. p: 71.9-73.0 °C; ^{1}H NMR (400 MHz, Chloroform-*d*) δ 6.73 (s, 2H), 6.69 (s, 1H), 6.03–5.95 (m, 2H), 5.67 (s, 1H), 5.38 (d, J = 9.6 Hz, 1H), 4.35–4.28 (m, 1H), 4.27–4.20 (m, 1H), 4.12 (dd, J = 12.0, 2.2 Hz, 1H), 2.29 (s, 6H), 2.11 (s, 3H), 2.00 (s, 3H). ^{13}C NMR (100 MHz, Chloroform-*d*) δ 170.9, 170.4, 157.2, 139.4, 130.0, 127.4, 124.4, 114.8, 93.1, 67.8, 65.1, 62.8, 21.5, 21.1, 20.8. HRMS (ESI) *m/z*: calcd. for $C_{18}H_{22}O_6$ Na^+ $(M + Na)^+$ 357.1309, found 357.1312; $[\alpha]_D^{32}$ = 42.077 (c = 0.910, $CHCl_3$).

((2*R*,3*S*,6*R*)-3-acetoxy-6-(p-tolyloxy)-3,6-dihydro-2*H*-pyran-2-yl)methyl acetate (3s) [55]

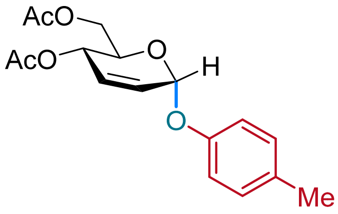

Colorless crystal; yield 75%; m. p.: 48.9-52.5 °C; ^{1}H NMR (400 MHz, Chloroform-*d*) δ 7.18–7.09 (m, 2H), 7.09–6.96 (m, 2H), 6.05 (d, J = 9.3 Hz, 2H), 5.67 (d, J = 2.0 Hz, 1H), 5.41 (d, J = 9.3 Hz, 1H), 4.38–4.23 (m, 2H), 4.23–4.12 (m, 1H), 2.32 (s, 3H), 2.13 (d, J = 3.1 Hz, 3H), 2.04 (d, J = 4.2 Hz, 3H). ^{13}C NMR (100 MHz, Chloroform-*d*)

δ 170.9, 170.4, 155.0, 132.1, 130.1, 130.0, 127.3, 117.2, 93.4, 67.7, 65.1, 62.8, 21.1, 20.9, 20.7. HRMS (ESI) *m/z*: calcd. for $C_{17}H_{20}O_6$ Na^+ $(M + Na)^+$ 343.1152, found 343.1146; $[\alpha]_D^{18}$ = 108.470 (c = 0.244, MeOH).

((2*R*,3*S*)-3-acetoxy-6-(4-methoxyphenoxy)-3,6-dihydro-2*H*-pyran-2-yl)methyl acetate (3t) [55]

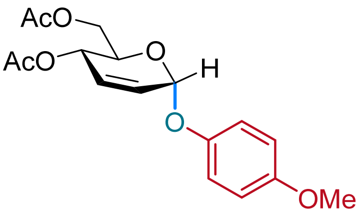

Colorless crystal; yield 79%; m. p.: 69.6-71.4 °C; ^{1}H NMR (400 MHz, Chloroform-*d*) δ 7.09–7.01 (m, 2H), 6.86–6.78 (m, 2H), 6.01 (s, 2H), 5.57 (s, 1H), 5.37 (d, *J* = 9.3 Hz, 1H), 4.31–4.23 (m, 2H), 4.19–4.12 (m, 1H), 3.78 (s, 3H), 2.11 (s, 3H), 2.03 (s, 3H). ^{13}C NMR (100 MHz, Chloroform-*d*) δ 170.9, 170.4, 155.3, 151.2, 130.1, 127.4, 118.8, 114.7, 94.2, 67.7, 65.3, 62.9, 55.8, 21.1, 20.9. HRMS (ESI) *m/z*: calcd. for $C_{17}H_{20}O_7$ Na^+ $(M + Na)^+$ 359.1101, found 359.1135; $[\alpha]_D^{13}$ = 26.096 (c = 0.038, DCM).

((2*R*,3*S*)-3-acetoxy-6-(naphthalen-1-yloxy)-3,6-dihydro-2*H*-pyran-2-yl)methyl acetate (3u) [56]

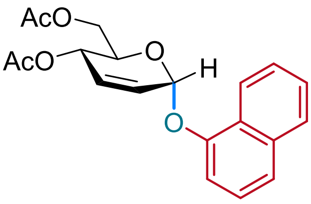

Yellowish oil; yield 60%; ^{1}H NMR (400 MHz, Chloroform-*d*) δ 8.25–8.18 (m, 1H), 7.86–7.79 (m, 1H), 7.56–7.45 (m, 3H), 7.40 (t, *J* = 7.9 Hz, 1H), 7.27 (s, 1H), 6.18–6.07 (m, 2H), 5.90 (s, 1H), 5.48–5.40 (m, 1H), 4.32 (d, *J* = 2.5Hz, 1H), 4.30 (t, *J* = 4.1 Hz, 1H), 4.14 (dd, *J* = 10.9, 5.0 Hz, 1H), 2.13 (s, 3H), 1.94 (s, 3H). ^{13}C NMR (100 MHz, Chloroform-*d*) δ 170.9, 170.5, 153.0, 134.6, 130.4, 127.7, 127.2, 126.5, 126.2, 126.0, 125.6, 122.2, 122.0, 109.5, 93.4, 68.2, 65.2, 62.7, 21.2, 20.8. HRMS (ESI) *m/z*: calcd. for $C_{20}H_{20}O_6$ Na^+ $(M + Na)^+$ 379.1152, found 379.1158; $[\alpha]_D^{20}$ =

20.23 (c = 0.094, DCM).

(2*R*,3*S*,6*R*)-6-([1,1'-biphenyl]-4-yloxy)-2-(acetoxymethyl)-3,6-dihydro-2*H*-pyran-3-yl acetate (3v)

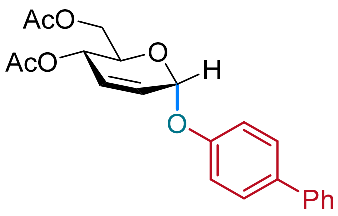

Colorless crystal; yield 79%; m. p.: 110.3-110.5 °C; ^{1}H NMR (400 MHz, Chloroform-*d*) δ 7.63–7.5 (m, 4H), 7.43 (t, *J* = 7.5 Hz, 2H), 7.38–7.28 (m, 1H), 7.25–7.13 (m, 2H), 6.06 (d, *J* = 10.2 Hz, 2H), 5.75 (s, 1H), 5.41 (d, *J* = 9.3 Hz, 1H), 4.30 (ddd, *J* = 17.6, 13.3, 5.7 Hz, 2H), 4.16 (d, *J* = 11.6 Hz, 1H), 2.12 (s, 3H), 1.99 (s, 3H). ^{13}C NMR (100 MHz, Chloroform-*d*) δ 170.9, 170.4, 156.7, 140.7, 135.7, 130.3, 128.9, 128.3, 127.1, 127.0, 126.9, 117.4, 93.1, 67.9, 65.1, 62.8, 21.1, 20.9. HRMS (ESI) *m/z*: calcd. for $C_{22}H_{22}O_6$ Na^+ $(M + Na)^+$ 405.1309, found 405.1302; $[\alpha]_D^{15}$ = 148.850 (c = 0.376, DCM).

((2*R*,3*S*)-3-acetoxy-6-(2-bromophenoxy)-3,6-dihydro-2*H*-pyran-2-yl)methyl acetate (3w)

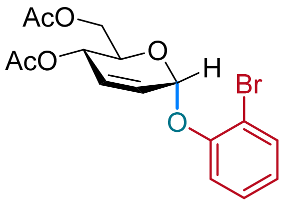

Colorless oil; yield 80%; ^{1}H NMR (400 MHz, Chloroform-*d*) δ 7.58-7.51 (m, 1H), 7.26 (d, *J* = 1.9 Hz, 2H), 7.03–6.88 (m, 1H), 6.07 (s, 2H), 5.67 (s, 1H), 5.39 (d, *J* = 9.5 Hz, 1H), 4.39–4.25 (m, 2H), 4.17 (dd, *J* = 11.9, 2.0 Hz, 1H), 2.12 (s, 3H), 2.02 (s, 3H). ^{13}C NMR (100 MHz, Chloroform-*d*) δ 170.8, 170.4, 154.0, 133.5, 130.7, 128.6, 126.7, 124.4, 119.0, 114.3, 94.6, 68.2, 65.1, 62.7, 21.1, 20.9. HRMS (ESI) *m/z*: calcd. for $C_{16}H_{17}BrO_6$ Na^+ $(M + Na)^+$ 407.0101, found 407.0093; $[\alpha]_D^{19}$ = 145.460 (c = 0.162, MeOH).

((2*R*,3*S*)-3-acetoxy-6-(3-bromophenoxy)-3,6-dihydro-2*H*-pyran-2-yl)methyl acetate (3x)

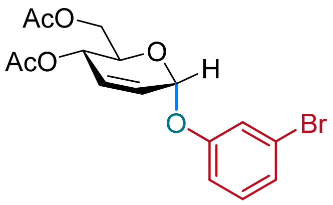

Yellowish oil; yield 70%; ^{1}H NMR (400 MHz, Chloroform-*d*) δ 7.31–7.29 (m, 1H), 7.19–7.13 (m, 2H), 7.03 (dt, *J* = 6.2, 2.6 Hz, 1H), 6.08–5.95 (m, 2H), 5.66 (s, 1H), 5.37 (dq, *J* = 9.6, 1.7 Hz, 1H), 4.28 (dd, *J* = 11.9, 6.0 Hz, 1H), 4.20 (ddd, *J* = 9.6, 6.0, 2.2 Hz, 1H), 4.13(dd, *J* = 11.8, 2.2Hz, 1H), 2.11 (s, 3H), 2.01 (s, 3H). ^{13}C NMR (100 MHz, Chloroform-*d*) δ 170.9, 170.4, 157.9, 130.7, 130.6, 126.8, 125.8, 122.8, 120.7, 116.0, 93.2, 68.1, 65.1, 62.8, 21.1, 20.8. HRMS (ESI) *m/z*: calcd. for $C_{16}H_{17}BrO_6$ Na^+ $(M + Na)^+$ 407.0101, found 407.0094; $[\alpha]_D^{20}$ = 67.246 (c = 0.276, DCM).

((2*R*,3*S*)-3-acetoxy-6-(4-bromophenoxy)-3,6-dihydro-2*H*-pyran-2-yl)methyl acetate (3y) [57]

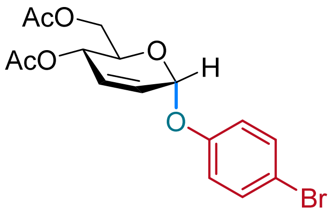

Colorless oil; yield 76%; ^{1}H NMR (400 MHz, Chloroform-*d*) δ 7.39 (dd, *J* = 9.0, 2.1 Hz, 2H), 6.98 (dd, *J* = 9.3, 2.3 Hz, 2H), 6.10–5.92 (m, 2H), 5.64 (s, 1H), 5.37 (dq, *J* = 9.5, 1.6 Hz, 1H), 4.26 (dd, *J* = 11.8, 5.7 Hz, 1H), 4.19(ddd, *J* = 9.6, 5.6, 2.1 Hz, 1H), 4.15–4.08 (m, 1H), 2.10 (s, 3H), 1.98 (s, 3H). ^{13}C NMR (100 MHz, Chloroform-*d*) δ 170.8, 170.3, 156.3, 132.4, 130.5, 126.8, 119.0, 115.0, 93.1, 68.0, 65.1, 62.7, 21.1, 20.8. HRMS (ESI) *m/z*: calcd. for $C_{16}H_{17}BrO_6$ NH_4^+ $(M + NH_4)^+$ 402.0547, found 402.0536; $[\alpha]_D^{14}$ = 131.72 (c = 0.420, MeOH).

((2*R*,3*S*)-3-acetoxy-6-(4-chlorophenoxy)-3,6-dihydro-2*H*-pyran-2-yl)methyl acetate (3z) [58]

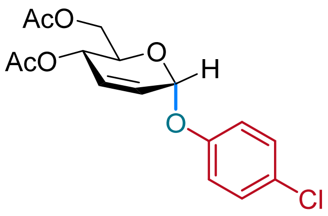

Colorless crystal; yield 75%; m. p.: 46.8-48.1 °C; ^{1}H NMR (400 MHz, Chloroform-*d*) δ 7.24 (s, 2H), 7.07–7.01 (m, 2H), 6.08–5.94 (m, 2H), 5.64 (s, 1H), 5.37 (dt, *J* = 9.6, 1.7 Hz, 1H), 4.27 (dd, *J* = 11.8, 5.6 Hz, 1H), 4.20 (ddd, *J* = 9.7, 5.6, 2.1 Hz, 1H), 4.13 (dd, *J* = 11.8, 2.1 Hz, 1H), 2.11 (s, 3H), 1.99 (s, 3H). ^{13}C NMR (100 MHz, Chloroform-*d*) δ 170.8, 170.4, 155.8, 130.5, 129.5, 127.6, 126.8, 118.5, 93.3, 68.0, 65.1, 62.7, 21.1, 20.8. HRMS (ESI) *m/z*: calcd. for $C_{16}H_{17}ClO_6$ NH_4^+ $(M + NH_4)^+$ 358.1052, found 358.1037; $[\alpha]_D^{20}$ = 102.140 (c = 0.442, MeOH).

((2*R*,3*S*,6*R*)-3-acetoxy-6-(4-fluorophenoxy)-3,6-dihydro-2*H*-pyran-2-yl)methyl acetate (3aa) [59]

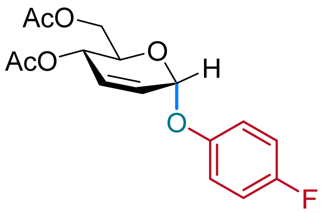

Colorless crystal; yield 85%; m. p.: 51.9-53.6 °C; ^{1}H NMR (400 MHz, Chloroform-*d*) δ 7.11–6.92 (m, 4H), 6.02 (t, *J* = 8.7 Hz, 2H), 5.60 (s, 1H), 5.37 (d, *J* = 8.8 Hz, 1H), 4.29–4.21 (m, 2H), 4.14 (d, *J* = 10.4 Hz, 1H), 2.11 (s, 3H), 2.01 (s, 3H). ^{13}C NMR (100 MHz, Chloroform-*d*) δ 170.9, 170.4, 130.4, 127.0, 118.7, 118.6, 116.1, 115.9, 93.8, 67.9, 65.1, 62.8, 21.1, 20.9. HRMS (ESI) *m/z*: calcd. for $C_{16}H_{17}FO_6$ NH_4^+ $(M + NH_4)^+$ 342.1347 found 342.1355; $[\alpha]_D^{20}$ = 127.08 (c = 0.192, MeOH).

((2*R*,3*S*)-3-acetoxy-6-(4-nitrophenoxy)-3,6-dihydro-2*H*-pyran-2-yl)methyl acetate (3ab) [55]

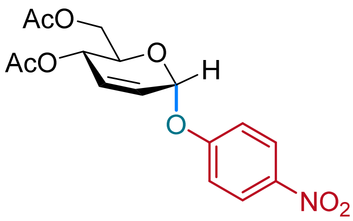

Yellow solid; yield 80%; m. p.: 58.6-59.8 °C; ^{1}H NMR (400 MHz, Chloroform-*d*) δ 8.20 (d, *J* = 9.2 Hz, 2H), 7.17 (d, *J* = 9.2 Hz, 2H), 6.10 (d, *J* = 10.2 Hz, 1H), 6.00 (dt, *J* = 10.2, 2.3 Hz, 1H), 5.80 (s, 1H), 5.40 (d, *J* = 9.7 Hz, 1H), 4.26 (dd, *J* = 12.4, 5.8 Hz, 1H), 4.16 (d, *J* = 7.6 Hz, 1H), 4.14–4.08 (m, 1H), 2.11 (s, 3H), 1.96 (s, 3H). ^{13}C NMR (100 MHz, Chloroform-*d*) δ 170.8, 170.3, 161.9, 142.6, 131.2, 126.0, 125.9, 116.7, 92.7, 68.4, 64.8, 62.5, 21.1, 20.8. HRMS (ESI) *m/z*: calcd. for $C_{16}H_{17}NO_8$ NH_4^+ (M + NH_4)$^+$ 369.1292, found 369.1279; $[\alpha]_D^{20}$ = 198.950 (c = 0.354, MeOH).

((2*R*,3*S*,6*R*)-3-acetoxy-6-(3,4-dichlorophenoxy)-3,6-dihydro-2*H*-pyran-2-yl)methyl acetate (3ac)

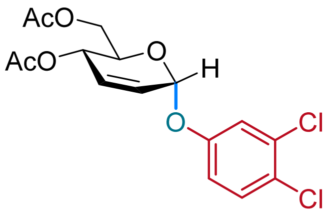

Colorless oil; yield 70%; ^{1}H NMR (400 MHz, Chloroform-*d*) δ 7.34 (d, *J* = 8.9 Hz, 1H), 7.27–7.24 (m, 1H), 6.95 (dd, *J* = 8.9, 2.8 Hz, 1H), 6.18–5.90 (m, 2H), 5.63 (s, 1H), 5.44–5.30 (m, 1H), 4.26 (dd, *J* = 11.8, 5.8 Hz, 1H), 4.23–4.17 (m,1H), 4.17–4.10 (m, 1H), 2.11 (s, 3H), 2.01 (s, 3H). ^{13}C NMR (100 MHz, Chloroform-*d*) δ 170.9, 170.4, 156.1, 132.9, 130.8, 130.7, 126.4, 126.0, 119.3, 117.0, 93.4, 68.1, 64.9, 62.7, 21.1, 20.8. HRMS (ESI) *m/z*: calcd. for $C_{16}H_{16}Cl_2O_6$ Na^+ (M + Na)$^+$ 397.0216, found 397.0211; $[\alpha]_D^{15}$ = 176.03 (c = 0.268, DCM).

((2*R*,3*S*,6*R*)-3-acetoxy-6-(butylthio)-3,6-dihydro-2*H*-pyran-2-yl)methyl acetate (4a) [60]

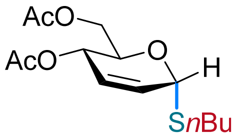

Yellowish oil; yield 89%; ^{1}H NMR (400 MHz, Chloroform-d) δ 5.96 (ddd, *J* = 10.2, 2.9, 2.0 Hz, 1H), 5.79 (d, *J* = 10.2 Hz, 1H), 5.65–5.52 (m, 1H), 5.52–5.29 (m, 1H), 4.34 (ddd, *J* = 11.1, 4.6, 2.1 Hz, 1H), 4.29 (t, *J* = 5.9 Hz, 1H), 4.19 (dd, *J* = 11.8, 1.8 Hz, 1H), 2.75 (dt, *J* = 12.9, 7.4 Hz, 1H), 2.66 (dt, *J* = 12.9, 7.5 Hz, 1H), 2.12 (d, *J* =

4.7 Hz, 6H), 1.64–1.53 (m, 2H), 1.48–1.42 (m, 2H), 0.95 (d, *J* = 7.3 Hz, 3H). 13C NMR (100 MHz, Chloroform-d) δ 170.97, 170.49, 129.33, 126.84, 80.57, 66.93, 65.31, 63.20, 32.18, 31.91, 22.12, 21.15, 20.94, 13.78. HRMS (ESI) *m/z*: calcd. for $C_{14}H_{22}O_5S$ NH_4^+ $(M + NH_4)^+$ 320.1526, found 320.1534; $[\alpha]_D^{11}$ = 54.230 (c = 0.120, MeOH).

((2*R*,3*S*,6*R*)-3-acetoxy-6-(tert-butylthio)-3,6-dihydro-2*H*-pyran-2-yl)methyl acetate (4b)

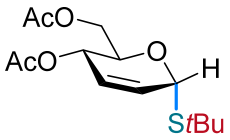

Yellow oil; yield 89%; ^{1}H NMR (400 MHz, Chloroform-*d*) δ 5.89 (ddd, *J* = 8.6, 4.4, 1.9 Hz, 1H), 5.78–5.71 (m, 2H), 5.32 (d, *J* = 11.3 Hz, 1H), 4.34 (ddt, *J* = 11.5, 5.4, 3.5 Hz, 1H), 4.24 (dd, *J* = 12.1, 5.4 Hz, 1H), 4.14 (dd, *J* = 12.0, 2.2 Hz, 1H), 2.07 (s, 3H), 2.07(s, 3H), 1.39 (s, 9H). ^{13}C NMR (100 MHz, Chloroform-*d*) δ 171.0, 170.5, 129.7, 126.6, 78.2, 66.7, 65.1, 63.2, 44.5, 31.6, 21.2, 21.0. HRMS (ESI) *m/z*: calcd. for $C_{14}H_{22}O_5S$ Na^+ $(M + Na)^+$ 325.1080, found 325.1074; $[\alpha]_D^{21}$ = 46.444 (c = 1.180, MeOH).

((2*R*,3*S*)-3-acetoxy-6-((4-fluorophenyl)thio)-3,6-dihydro-2*H*-pyran-2-yl)methyl acetate (4c)

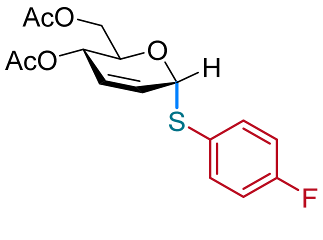

Yellowish oil; yield 72%; ^{1}H NMR (400 MHz, Chloroform-*d*) δ 7.54 (dd, *J* = 8.8, 5.3 Hz, 2H), 7.01 (t, *J* = 8.7 Hz, 2H), 6.04 (ddd, *J* = 10.1, 3.1, 1.9 Hz, 1H), 5.86 (dt, *J* = 10.1, 1.5 Hz, 1H), 5.70–5.56 (m, 1H), 5.37 (dq, *J* = 9.4, 1.8 Hz, 1H), 4.46 (ddd, *J* = 9.0, 5.2, 3.2 Hz, 1H), 4.25 (d, *J* = 5.1 Hz, 1H), 4.23(d, *J* = 9.0 Hz, 1H), 2.11 (s, 3H), 2.09 (s, 3H). ^{13}C NMR (100 MHz, Chloroform-*d*) δ 170.8, 170.4, 134.7, 134.6, 128.5,

127.9, 116.3, 116.1, 84.3, 67.4, 65.3, 63.3, 21.1, 20.9. HRMS (ESI) *m/z*: calcd. for $C_{16}H_{17}FO_5S$ Na^+ $(M + Na)^+$ 363.0673, found 363.0662; $[\alpha]_D^{25}$ = 265.220 (c = 0.428, DCM).

((2*R*,3*R*,4*S*)-3-acetoxy-4-((4-fluorophenyl)thio)-3,4-dihydro-2*H*-pyran-2-yl)methyl acetate (4c')

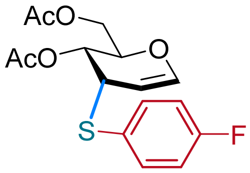

Colorless oil; yield 6%; ^{1}H NMR (400 MHz, Chloroform-*d*) δ 7.42 (dd, *J* = 8.7, 5.3 Hz, 2H), 6.99 (t, *J* = 8.6 Hz, 2H), 6.41 (d, *J* = 5.9 Hz, 1H), 5.17 (dd, *J* = 9.9, 4.6 Hz, 1H), 4.90 (t, *J* = 5.8 Hz, 1H), 4.41 (ddd, *J* = 10.0, 4.3, 2.0 Hz, 1H), 4.35(d, *J* = 4.2 Hz), 4.34–4.29 (m, 2H),4.23 (t, *J* = 5.2 Hz, 1H), 2.06 (s, 3H), 1.59 (s, 3H). ^{13}C NMR (100 MHz, Chloroform-*d*) δ 170.8, 169.9, 145.2, 134.5, 134.4, 116.3, 116.1, 98.6, 70.9, 68.9, 62.3, 44.3, 20.9, 20.2. HRMS (ESI) *m/z*: calcd. for $C_{16}H_{17}FO_5S$ Na^+ $(M + Na)^+$ 363.0673, found 363.0654; $[\alpha]_D^{20}$ = 99.551 (c = 0.598, MeOH).

((2*R*,3*S*)-3-acetoxy-6-(p-tolylthio)-3,6-dihydro-2*H*-pyran-2-yl)methyl acetate (4d) [50]

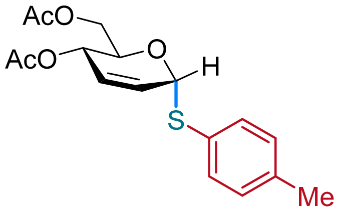

Colorless crystal; yield 70%; m. p.: 56.7-58.6 °C; ^{1}H NMR (400 MHz, Chloroform-*d*) δ 7.44 (d, *J* = 8.1 Hz, 2H), 7.12 (d, *J* = 8.0 Hz, 2H), 6.06 (ddd, *J* = 10.1, 3.1, 1.9 Hz, 1H), 5.91–5.81 (m, 1H), 5.68 (s, 1H), 5.37 (dt, *J* = 9.5, 1.8 Hz, 1H), 4.49 (ddd, *J* = 9.2, 5.7, 2.5 Hz, 1H), 4.27 (dd, *J* = 12.1, 5.8 Hz, 1H), 4.22 (dd, *J* = 12.1, 2.6 Hz, 1H), 2.33 (s, 3H), 2.11 (s, 3H), 2.09 (s, 3H). ^{13}C NMR (100 MHz, Chloroform-*d*) δ 170.9, 170.5, 138.1, 132.6, 131.0, 129.8, 128.8, 127.5, 84.2, 67.2, 65.3, 63.3, 21.3, 21.2, 21.0. HRMS (ESI) *m/z*: calcd. for $C_{17}H_{20}O_5S$ Na^+ $(M + Na)^+$ 359.0924, found 359.0936;

$[\alpha]_D^{18}$ = 80.649 (c = 0.390, MeOH).

((2*R*,3*R*,4*S*)-3-acetoxy-4-(o-tolylthio)-3,4-dihydro-2*H*-pyran-2-yl)methyl acetate (4e) [61]

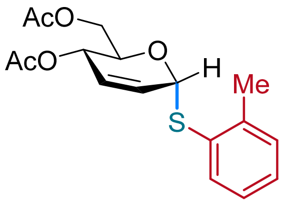

Yellowish oil; yield 75%; ^{1}H NMR (400 MHz, Chloroform-*d*) δ 7.69–7.57 (m, 1H), 7.18 (d, *J* = 6.6 Hz, 3H), 6.14–6.04 (m, 1H), 5.87 (d, *J* = 10.1 Hz, 1H), 5.74 (s, 1H), 5.43–5.34 (m, 1H), 4.45 (dd, *J* = 10.8, 4.1 Hz, 1H), 4.30 (dd, *J* = 12.1, 5.7 Hz, 1H), 4.17 (d, *J* = 12.0 Hz, 1H), 2.43 (s, 3H), 2.11 (s, 3H), 2.05 (s, 3H). ^{13}C NMR (100 MHz, Chloroform-*d*) δ 170.9, 170.4, 139.3, 134.2, 132.2, 130.3, 128.9, 127.8, 127.6, 126.8, 83.3, 67.6, 65.2, 63.1, 21.1, 21.0, 20.9. HRMS (ESI) *m/z*: calcd. for $C_{17}H_{20}O_5S\ Na^+$ $(M + Na)^+$ 359.0924, found 359.0954; $[\alpha]_D^{14}$ = 264.360 (c = 0.62, DCM).

((2*R*,3*S*)-3-acetoxy-6-(o-tolylthio)-3,6-dihydro-2*H*-pyran-2-yl) methyl acetate (4e')

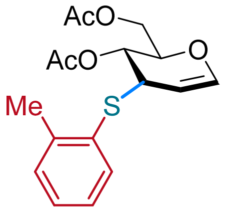

Colorless crystal; yield 8%; m. p.: 64.6-65.7 °C; ^{1}H NMR (400 MHz, Chloroform-*d*) δ 7.41 (dd, *J* = 5.4, 3.7 Hz, 1H), 7.18 (dd, *J* = 5.4, 3.8 Hz, 1H), 7.12 (dd, *J* = 5.7, 3.4 Hz, 2H), 6.42 (d, *J* = 5.8 Hz, 1H), 5.17 (dd, *J* = 10.3, 4.5 Hz, 1H), 4.95 (t, *J* = 5.8 Hz, 1H), 4.56 (ddd, *J* = 10.3, 4.2, 2.3 Hz, 1H), 4.37 (d, *J* = 4.3 Hz, 1H), 4.33(dd, *J* = 12.3, 2.3 Hz, 1H), 4.30–4.24 (m, 1H), 2.46 (s, 3H), 2.07 (s, 3H), 1.44 (s, 3H). ^{13}C NMR (100 MHz, Chloroform-*d*) δ 170.9, 170.0, 145.1, 139.7, 134.8, 132.8, 130.6, 127.5, 126.6, 98.6, 71.0, 68.8, 62.4, 43.3, 21.1, 20.9, 19.9. HRMS (ESI) *m/z*: calcd. for $C_{17}H_{20}O_5S$ Na^+ $(M + Na)^+$ 359.0924, found 359.0923; $[\alpha]_D^{19}$ = 196.330 (c = 0.232, MeOH).

((2*R*,3*S*)-3-acetoxy-6-hydroxy-3,6-dihydro-2*H*-pyran-2-yl) methyl acetate (5)

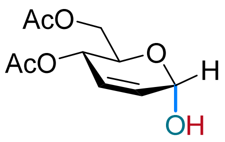

White solid; yield 87%; m. p.:65.1-67.9°C; ^{1}H NMR (400 MHz, Chloroform-*d*) δ 5.93 (dt, *J* = 10.1, 1.3 Hz, 1H), 5.81 (dt, *J* = 10.2, 2.4 Hz, 1H), 5.48–5.39 (m, 1H), 5.32 (dq, *J* = 9.7, 1.8 Hz, 1H), 4.22(dd, *J* = 12.1, 5.4 Hz, 1H), 4.19–4.14 (m, 1H), 4.08 (dtd, *J* = 10.8, 5.4, 2.5 Hz, 1H), 2.08 (s, 3H), 2.06 (s, 3H). ^{13}C NMR (100 MHz, Chloroform-*d*) δ 170.8, 170.3, 129.8, 127.5, 90.6, 67.4, 65.3, 63.0, 21.1, 20.9. HRMS (ESI) *m/z*: calcd. for $C_{10}H_{14}O_6$ Na^+ $(M + Na)^+$ 253.0683, found 253.0654; $[\alpha]_D^{32}$ = – 4.218 (c = 0.318, $CHCl_3$).

((2*R*,3*R*)-3-acetoxy-6-ethoxy-3,6-dihydro-2*H*-pyran-2-yl) methyl acetate (6a) [62]

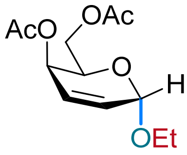

Colorless oil; yield 93%; ^{1}H NMR (400 MHz, Chloroform-*d*) δ 6.11 (dd, *J* = 10.0, 5.4 Hz, 1H), 6.03 (dd, *J* = 10.0, 2.9 Hz, 1H), 5.08 (d, *J* = 2.7 Hz, 1H), 5.02 (dd, *J* = 5.3, 2.5 Hz, 1H), 4.37 (m, 1H), 4.23 (s, 1H), 4.22 (d, *J* = 1.4 Hz, 1H), 3.84 (dq, *J* = 9.6, 7.1 Hz, 1H), 3.58 (dq, *J* = 9.6, 7.0 Hz, 1H), 2.08 (s, 3H), 2.07 (s, 3H), 1.25 (t, *J* = 7.1 Hz, 3H). ^{13}C NMR (100 MHz, Chloroform-*d*) δ 170.8, 170.5, 130.9, 125.3, 93.9, 66.8, 64.1, 63.0, 29.8, 21.0, 20.9, 15.4. HRMS (ESI) *m/z*: calcd. for $C_{12}H_{18}O_6$ Na^+ $(M + Na)^+$ 281.0995, found 281.0987; $[\alpha]_D^{16}$ = – 132.150 (c = 0.126, DCM).

((2*R*,3*R*,6*S*)-3-acetoxy-6-(cyclohexyloxy)-3,6-dihydro-2*H*-pyran-2-yl)methyl acetate (6b) [62]

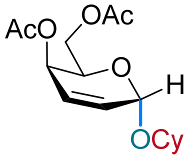

Colorless oil; yield 89%; ^{1}H NMR (400 MHz, Chloroform-*d*) δ 6.10 (dd, *J* = 10.4, 5.3 Hz, 1H), 6.01 (dd, *J* = 10.0, 3.0 Hz, 1H), 5.21 (d, *J* = 2.9 Hz, 1H), 5.02 (dd, *J* = 5.3, 2.5 Hz, 1H), 4.44–4.38 (m, 1H), 4.24–4.19 (m, 2H), 3.66 (tt, *J* = 9.3, 4.1 Hz, 1H),

2.08 (s, 3H), 2.06 (s, 3H), 1.93 (dd, *J* = 20.6, 10.7 Hz, 2H), 1.79–1.69 (m, 2H), 1.41–1.21 (m, 6H). ^{13}C NMR (100 MHz, Chloroform-*d*) δ 170.8, 170.5, 131.4, 125.1, 92.5, 66.8, 63.2, 33.9, 32.3, 25.7, 24.6, 24.4, 21.0, 20.9. HRMS (ESI) *m/z*: calcd. for $C_{16}H_{24}O_6$ Na^+ $(M + Na)^+$ 335.1465, found 335.1459; $[\alpha]_D^{20}$ = – 119.800 (c = 0.104, MeOH).

((2*R*,3*R*,6*R*)-3-acetoxy-6-(tert-butoxy)-3,6-dihydro-2*H*-pyran-2-yl) methyl acetate (6c) [63]

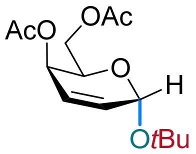

Colorless oil; yield 90%; ^{1}H NMR (400 MHz, Chloroform-*d*) δ 6.09 (ddd, *J* = 9.9, 5.5, 0.9 Hz, 1H), 5.93 (dd, *J* = 10.0, 3.1 Hz, 1H), 5.38 (d, *J* = 2.8 Hz, 1H), 5.01 (dd, *J* = 5.5, 2.4 Hz, 1H), 4.43 (td, *J* = 6.4, 2.5 Hz, 1H), 4.21 (d, *J* = 1.4Hz, 1H), 4.19 (s,1H), 2.08 (s, 3H), 2.05 (s, 3H), 1.29 (s, 9H). ^{13}C NMR (100 MHz, Chloroform-*d*) δ 170.8, 170.6, 132.3, 124.6, 88.8, 75.5, 66.5, 63.2, 63.0, 28.9, 21.0, 20.9. HRMS (ESI) *m/z*: calcd. for $C_{14}H_{22}O_6$ Na^+ $(M + Na)^+$ 309.1308, found 309.1314; $[\alpha]_D^{16}$ = – 133.650 (c = 0.082, DCM).

((2*R*,3*R*,6*S*)-3-acetoxy-6-(allyloxy)-3,6-dihydro-2*H*-pyran-2-yl)methyl acetate (6d) [62]

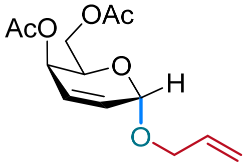

Colorless oil; yield 90%; ^{1}H NMR (400 MHz, Chloroform-*d*) δ 6.18–6.10 (m, 1H), 6.05 (dd, *J* = 10.1, 2.9 Hz, 1H), 5.94 (dddd, *J* = 16.9, 10.3, 6.4, 5.3 Hz, 1H), 5.31 (dq, *J* = 17.2, 1.5 Hz, 1H), 5.26–5.18 (m, 1H), 5.13 (d, *J* = 2.8 Hz, 1H), 5.03 (dd, *J* = 5.4, 2.5 Hz, 1H), 4.37 (td, *J* = 6.4, 5.8, 2.5 Hz, 1H), 4.30–4.23 (m, 1H), 4.24 (s,1H), 4.22 (d, *J* = 0.9 Hz, 1H), 4.08 (ddt, *J* = 12.7, 6.5, 1.2 Hz, 1H), 2.09 (s, 3H), 2.08 (s, 3H). ^{13}C NMR (100 MHz, Chloroform-*d*) δ 170.8, 170.5, 134.1, 130.7, 125.5, 118.0, 93.2, 69.1, 66.9, 63.0, 63.0, 21.0, 21.0. HRMS (ESI) *m/z*: calcd. for $C_{13}H_{18}O_6$ Na^+ (M +

Na)$^+$ 293.0995, found 293.0986; $[\alpha]_D^{15}$ = – 155.680 (c = 0.034, DCM).

((2*R*,3*R*,6*S*)-3-acetoxy-6-(prop-2-yn-1-yloxy)-3,6-dihydro-2*H*-pyran-2-yl)methyl acetate (6e) [62]

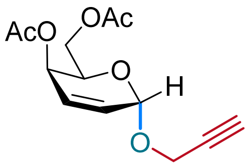

White solid; yield 90%; m. p.: 61.1-63.0 °C; ^{1}H NMR (400 MHz, Chloroform-*d*) δ 6.16 (dd, *J* = 9.9, 5.4 Hz, 1H), 6.09–6.00 (m, 1H), 5.29 (s, 1H), 5.02 (d, *J* = 5.1 Hz, 1H), 4.31 (s, 3H), 4.22 (d, *J* = 6.3 Hz, 2H), 2.48 (s, 1H), 2.08 (s, 6H). ^{13}C NMR (100 MHz, Chloroform-*d*) δ 170.8, 170.5, 130.2, 125.8, 92.1, 79.0, 75.1, 67.1, 62.8, 62.7, 54.7, 21.0, 21.0. HRMS (ESI) *m/z*: calcd. for $C_{13}H_{16}O_6$ NH_4^+ $(M + NH_4)^+$ 286.1285, found 286.1276; $[\alpha]_D^{15}$ = – 121.860 (c = 0.200, DCM).

((2*R*,3*R*)-3-acetoxy-6-(p-tolyloxy)-3,6-dihydro-2*H*-pyran-2-yl) methyl acetat (6f) [62]

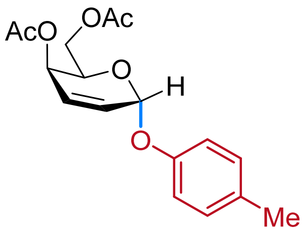

Colorless oil; yield 70%; ^{1}H NMR (400 MHz, Chloroform-*d*) δ 7.09 (d, *J* = 8.4 Hz, 2H), 7.01 (d, *J* = 8.5 Hz, 2H), 6.32–6.13 (m, 2H), 5.70 (d, *J* = 2.8 Hz, 1H), 5.10 (dd, *J* = 5.2, 2.4 Hz, 1H), 4.57 - 4.46 (m, 1H), 4.24 (td, *J* = 11.4, 6.5 Hz, 2H), 2.30 (s, 3H), 2.10 (s, 3H), 1.93 (s, 3H). ^{13}C NMR (100 MHz, Chloroform-*d*) δ 170.8, 170.5, 155.0, 132.1, 130.0, 126.1, 117.4, 93.1, 67.6, 62.7, 62.5, 21.0, 20.8, 20.7. HRMS (ESI) *m/z*: calcd. for $C_{17}H_{20}O_6$ NH_4^+ $(M + NH_4)^+$ 338.1598, found 338.1577; $[\alpha]_D^{16}$ = – 61.889 (c = 0.142, DCM).

((2*R*,3*R*,6*R*)-3-acetoxy-6-(4-chlorophenoxy)-3,6-dihydro-2*H*-pyran-2-yl) methyl acetate (6g) [62]

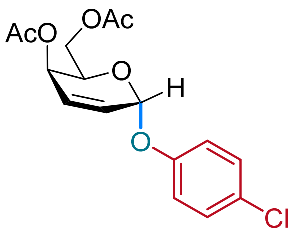

White solid; yield 80%; m. p.: 51.7-52.8 °C; ^{1}H NMR (400 MHz, Chloroform-*d*) δ 7.27–7.22 (m, 2H), 7.08–7.00 (m, 2H), 6.08–5.95 (m, 2H), 5.65 (s, 1H), 5.42–5.35 (m, 1H), 4.27 (dd, *J* = 11.8, 5.7 Hz, 1H), 4.24–4.17 (m, 1H), 4.17–4.10 (m, 1H), 2.11 (s, 3H), 2.00 (s, 3H). ^{13}C NMR (100 MHz, Chloroform-*d*) δ 170.8, 170.4, 155.7, 130.5, 129.5, 127.6, 126.8, 118.5, 93.2, 68.0, 65.0, 62.7, 21.1, 20.8. HRMS (ESI) *m/z*: calcd. for $C_{16}H_{17}ClO_6$ Na^+ $(M + Na)^+$ 363.0606, found 363.0646; $[\alpha]_D^{19}$ = 160.660 (c = 0.400, DCM).

((2*R*,3*R*,6*R*)-3-acetoxy-6-(tert-butylthio)-3,6-dihydro-2*H*-pyran-2-yl) methyl acetate (6h)

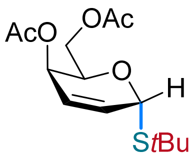

Colorless oil; yield 89%; ^{1}H NMR (400 MHz, Chloroform-*d*) δ 6.10–5.95 (m, 2H), 5.84 (dd, *J*= 2.9, 1.6 Hz, 1H), 5.07 (dd, *J* = 5.1, 2.4 Hz, 1H), 4.56 (td, *J* = 6.3, 2.4 Hz, 1H), 4.21 (d, *J* = 6.3 Hz, 2H), 2.08 (s, 3H), 2.04 (s, 3H), 1.40 (s, 9H). ^{13}C NMR (100 MHz, Chloroform-*d*) δ 170.8, 170.5, 132.5, 123.7, 78.2, 66.7, 63.7, 62.9, 44.5, 31.7, 21.0, 20.9. HRMS (ESI) *m/z*: calcd. for $C_{14}H_{22}O_5S$ Na^+ $(M + Na)^+$ 325.1080, found 325.1085; $[\alpha]_D^{14}$ = – 46.063 (c = 0.152, MeOH).

((2*R*,3*R*,6*R*)-3-acetoxy-6-((4-fluorophenyl)thio)-3,6-dihydro-2*H*-pyran-2-yl) methyl acetate (6i)

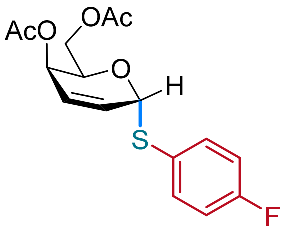

Colorless oil; yield 81%; ^{1}H NMR (400 MHz, Chloroform-*d*) δ 7.62–7.49 (m, 2H), 7.14–6.88 (m, 2H), 6.21 (dd, *J* = 10.0, 3.3 Hz, 1H), 6.11 (ddd, *J* = 10.0, 5.4, 1.7 Hz, 1H), 5.74 (dd, *J* = 3.3, 1.7 Hz, 1H), 5.13 (dd, *J* = 5.4, 2.5 Hz, 1H), 4.69 (ddd, *J* = 7.6, 5.3, 2.5 Hz, 1H), 4.27 (dd, *J* = 6.2, 2.0 Hz, 2H), 2.08 (d, *J* = 2.4 Hz, 6H). ^{13}C NMR (100 MHz, Chloroform-*d*) δ 170.71, 170.44, 134.76, 134.68, 131.27, 124.77, 116.25, 116.03, 84.14, 67.39, 63.42, 62.95, 20.95, 20.91. HRMS (ESI) *m/z*: calcd. for $C_{16}H_{17}FO_5S$ Na^+ $(M + Na)^+$ 363.0673, found 363.0679; $[\alpha]_D^{20}$ = 97.061 (c = 0.038, DCM).

((2*R*,3*S*,6*S*)-3-acetoxy-6-ethoxy-3,6-dihydro-2*H*-pyran-2-yl) methyl acetate (3a)

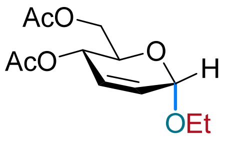

White solid; yield 91%; m. p.: 59.1-62.7 °C; ^{1}H NMR (400 MHz, Chloroform-*d*) δ 5.92–5.80 (m, 2H), 5.36–5.27 (m, 1H), 5.05 (s, 1H), 4.28–4.22 (m, 1H), 4.18 (d, *J* = 2.2 Hz, 1H), 4.16–4.09 (m, 1H), 3.83 (dq, *J* = 9.5, 7.1 Hz, 1H), 3.57 (dq, *J* = 9.5, 7.1 Hz, 1H), 2.10 (s, 3H), 2.08 (s, 3H) 1.25 (d, *J* = 7.1 Hz, 3H). ^{13}C NMR (100 MHz, Chloroform-*d*) δ 171.0, 170.5, 129.1, 128.1, 94.4, 66.9, 65.4, 64.5, 63.1, 21.1, 21.0, 15.4. HRMS (ESI) *m/z*: calcd. for $C_{12}H_{18}O_6$ Na^+ $(M + Na)^+$ 281.0996, found 281.0978; $[\alpha]_D^{13}$ = 83.883 (c = 0.218, DCM).

((2*R*,3*S*,6*R*)-3-acetoxy-6-((4-fluorophenyl)thio)-3,6-dihydro-2*H*-pyran-2-yl)methyl acetate (3aa)

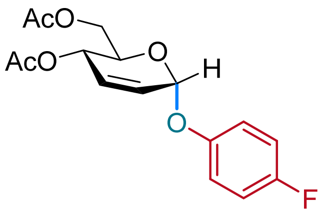

Colorless crystal; yield 81%; m. p.: 56.2-56.5 °C; ^{1}H NMR (400 MHz, Chloroform-*d*) δ 7.06 (dd, *J* = 9.1, 4.5 Hz, 2H), 7.03–6.93 (m, 2H), 6.07–5.96 (m, 2H), 5.61 (s, 1H), 5.37 (d, *J* = 9.4 Hz, 1H), 4.32–4.19 (m, 2H), 4.15 (d, *J* = 11.7 Hz, 1H), 2.11 (d, *J* = 3.6

Hz, 3H), 2.01 (d, *J* = 3.5 Hz, 3H). ^{13}C NMR (100 MHz, Chloroform-*d*) δ 170.9, 170.5, 134.8, 134.7, 128.4, 127.9, 116.3, 116.1, 84.4, 67.3, 65.2, 63.2, 21.2, 21.0. HRMS (ESI) *m/z*: calcd. for $C_{16}H_{17}FO_5S$ Na^+ $(M + Na)^+$ 363.0673, found 363.0676; $[\alpha]_D^{13}$ = 245.730 (c = 0.194, DCM).

((2*R*,3*S*,6*R*)-3-acetoxy-6-(tert-butylthio)-3,6-dihydro-2*H*-pyran-2-yl) methyl acetate (4b)

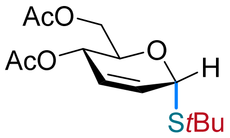

Yellowish oil; yield 86%; ^{1}H NMR (400 MHz, Chloroform-*d*) δ 5.90 (ddd, *J* = 8.1, 4.5, 2.0 Hz, 1H), 5.79–5.72 (m, 2H), 5.32 (dq, *J* = 9.4, 1.8 Hz, 1H), 4.38–4.30 (m, 1H), 4.24 (dd, *J* = 12.0, 5.4 Hz, 1H), 4.14 (dd, *J* = 12.1, 2.2 Hz, 1H), 2.07 (s, 3H), 2.07 (s, 3H),1.39 (s, 9H). ^{13}C NMR (100 MHz, Chloroform-*d*) δ 171.1, 170.6, 129.7, 126.6, 78.2, 66.7, 65.1, 63.3, 44.6, 31.6, 21.2, 21.0. HRMS (ESI) *m/z*: calcd. for $C_{14}H_{22}O_5S$ Na^+ $(M + Na)^+$ 325.1080, found 325.1074; $[\alpha]_D^{14}$ = 145.960 (c = 0.266, DCM).

((2*R*,3*S*,6*R*)-3-acetoxy-6-(4-fluorophenoxy)-3,6-dihydro-2*H*-pyran-2-yl) methyl acetate (4c)

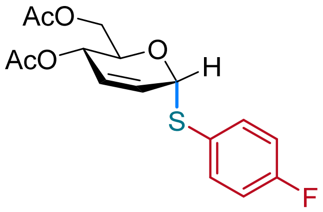

Colorless oil; yield 80%; ^{1}H NMR (400 MHz, Chloroform-*d*) δ 7.57–7.51 (m, 2H), 7.05–6.99 (m, 2H), 6.05 (ddd, *J* = 10.1, 3.0, 2.0 Hz, 1H), 5.90–5.82 (m, 1H), 5.68–5.62 (m, 1H), 5.37 (dd, *J* = 9.5, 1.9 Hz, 1H), 4.47 (ddd, *J* = 9.1, 5.3, 3.0 Hz, 1H), 4.27–4.22 (m, 2H), 2.11 (s, 3H), 2.09 (s, 3H). ^{13}C NMR (100 MHz, Chloroform-*d*) δ 170.9, 170.5, 134.8, 134.7, 128.4, 127.9, 116.3, 116.1, 84.4, 67.3, 65.2, 63.2, 21.2, 21.0. HRMS (ESI) *m/z*: calcd. for $C_{16}H_{17}FO_6$ Na^+ $(M + Na)^+$ 347.0901, found

347.0909; $[\alpha]_D^{15}$ = 145.960 (c = 0.254, DCM).

(2*S*,3*R*,6*R*)-6-ethoxy-2-methyl-3,6-dihydro-2*H*-pyran-3-yl acetate (7a) [64]

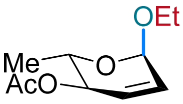

Colorless oil; yield 89%; ^{1}H NMR (400 MHz, Chloroform-*d*) δ 5.83 (t, *J* = 8.9 Hz, 2H), 5.04 (d, *J* = 9.1 Hz, 1H), 4.97 (s, 1H), 4.02–3.93 (m, 1H), 3.83 (dt, *J* = 9.4, 7.2 Hz, 1H), 3.60–3.48 (m, 1H), 2.07 (s, 3H), 1.22 (dd, *J* = 6.7, 3.8 Hz, 6H). ^{13}C NMR (100 MHz, Chloroform-*d*) δ 170.7, 129.6, 128.0, 94.3, 71.0, 64.8, 64.2, 21.3, 18.1, 15.5. HRMS (ESI) *m/z*: calcd. for $C_{10}H_{16}O_4$ Na^+ $(M + Na)^+$ 223.0941, found 223.0934; $[\alpha]_D^{15}$ = – 140.290 (c = 0.214, DCM).

(2*S*,3*R*,6*R*)-6-(allyloxy)-2-methyl-3,6-dihydro-2*H*-pyran-3-yl acetate (7b) [65]

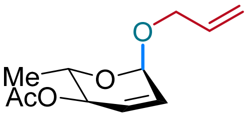

Yellowish oil; yield 77%; ^{1}H NMR (400 MHz, Chloroform-*d*) δ 5.99–5.88 (m, 1H), 5.88–5.78 (m, 2H), 5.30 (dq, *J* = 17.2, 1.5 Hz, 1H), 5.22–5.17 (m, 1H), 5.05 (dd, *J* = 9.2, 1.4 Hz, 1H), 5.00 (s, 1H), 4.25 (ddt, *J* = 12.8, 5.2, 1.4 Hz, 1H), 4.05 (ddt, *J* = 12.8, 6.3, 1.2 Hz, 1H), 3.98 (dq, *J* = 9.2, 6.3 Hz, 1H), 2.08 (s, 3H), 1.22 (d, *J* = 6.3 Hz, 3H). ^{13}C NMR (100 MHz, Chloroform-*d*) δ 170.7, 134.4, 129.9, 127.8, 117.5, 93.6, 71.0, 69.2, 64.9, 21.3, 18.1. HRMS (ESI) *m/z*: calcd. for $C_{11}H_{16}O_4$ Na^+ $(M + Na)^+$ 235.0941, found 235.0936; $[\alpha]_D^{15}$ = – 146.670 (c = 0.182, DCM).

(2*S*,3*R*,6*R*)-2-methyl-6-(prop-2-yn-1-yloxy)-3,6-dihydro-2*H*-pyran-3-yl acetate (7c) [65]

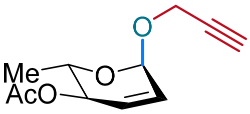

Colorless oil; yield 81%; ^{1}H NMR (400 MHz, Chloroform-*d*) δ 5.88 (d, *J* = 10.2 Hz, 1H), 5.80 (dt, *J* = 10.2, 2.2 Hz, 1H), 5.16 (s, 1H), 5.10–5.02 (m, 1H), 4.29 (d, *J* = 2.4 Hz, 2H), 3.95 (dq, *J* = 7.7, 5.7, 5.1 Hz, 1H), 2.45 (t, *J* = 2.3 Hz, 1H), 2.08 (s, 3H), 1.22 (d, *J* = 6.3 Hz, 3H). ^{13}C NMR (100 MHz, Chloroform-*d*) δ 170.7, 130.4, 127.3,

92.9, 79.5, 74.7, 70.8, 65.2, 55.0, 21.2, 18.0. HRMS (ESI) *m/z*: calcd. for $C_{11}H_{14}O_4$ Na^+ $(M + Na)^+$ 233.0784, found 233.0780; $[\alpha]_D^{15}$ = – 169.230 (c = 0.268, DCM).

(2*S*,3*R*,6*S*)-6-(4-fluorophenoxy)-2-methyl-3,6-dihydro-2*H*-pyran-3-yl acetate (7d)

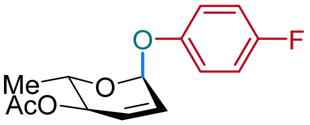

White solid; yield 69%; m. p.: 59.0-59.7 °C; ^{1}H NMR (400 MHz, Chloroform-*d*) δ 7.07–6.95 (m, 4H), 6.03–5.92 (m, 2H), 5.56 (s, 1H), 5.15–5.08 (m, 1H), 4.08 (dq, *J* = 9.4, 6.3 Hz, 1H), 2.11 (s, 3H), 1.22 (d, *J* = 6.2 Hz, 3H). ^{13}C NMR (100 MHz, Chloroform-*d*) δ 170.7, 130.9, 126.9, 118.3, 118.2, 116.1, 115.9, 93.7, 70.6, 65.9, 21.2, 18.1. HRMS (ESI) *m/z*: calcd. for $C_{14}H_{15}FO_4$ Na^+ $(M + Na)^+$ 289.0847, found 289.0842; $[\alpha]_D^{15}$ = – 206.970 (c = 0.104, DCM).

(3*S*,6*S*)-6-ethoxy-3,6-dihydro-2*H*-pyran-3-yl acetate (8a) [66]

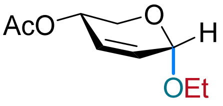

Colorless oil; yield 87%; ^{1}H NMR (400 MHz, Chloroform-*d*) δ 5.98–5.91 (m, 1H), 5.87 (d, *J* = 10.3 Hz, 1H), 5.27 (t, *J* = 6.5 Hz, 1H), 4.95 (s, 1H), 3.90–3.78 (m, 3H), 3.60–3.50 (m, 1H), 2.07 (s, 3H), 1.23 (d, *J* = 7.0 Hz, 3H). ^{13}C NMR (100 MHz, Chloroform-*d*) δ 170.8, 129.6, 128.8, 94.3, 65.1, 64.3, 60.4, 21.2, 15.4. HRMS (ESI) *m/z*: calcd. for $C_9H_{14}O_4$ Na^+ $(M + Na)^+$ 209.0784, found 209.1780; $[\alpha]_D^{14}$ = 150.83 (c = 0.020, DCM).

(3*S*,6*S*)-6-(allyloxy)-3,6-dihydro-2*H*-pyran-3-yl acetate (8b) [67]

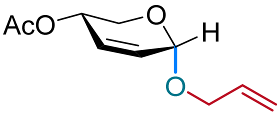

Colorless oil; yield 70%; ^{1}H NMR (400 MHz, Chloroform-*d*) δ 5.99–5.85 (m, 3H), 5.36–5.25 (m, 2H), 5.21 (d, *J* = 10.3 Hz, 1H), 4.99 (s, 1H), 4.28 (dd, *J* = 12.7, 5.2 Hz, 1H), 4.06 (dd, *J* = 12.7, 6.3 Hz, 1H), 3.83 (qd, *J* = 11.0, 6.9 Hz, 2H), 2.07 (s, 3H). ^{13}C NMR (100 MHz, Chloroform-*d*) δ 170.7, 134.3, 129.2, 129.1, 117.7, 93.5, 69.3, 65.1,

60.2, 21.2. HRMS (ESI) *m/z*: calcd. for $C_{10}H_{14}O_4$ Na^+ $(M + Na)^+$ 221.0784, found 221.0776; $[\alpha]_D^8$ = 180.120 (c = 0.042, DCM).

(3*S*,6*S*)-6-(prop-2-yn-1-yloxy)-3,6-dihydro-2*H*-pyran-3-yl acetate (8c) [67]

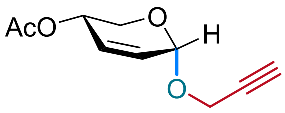

Colorless oil; yield 75%; ^{1}H NMR (400 MHz, Chloroform-*d*) δ 5.97 (d, *J* = 9.6 Hz, 1H), 5.86 (dt, *J* = 10.3, 2.0 Hz, 1H), 5.39–5.25 (m, 1H), 5.16 (s, 1H), 4.31 (t, *J* = 2.1 Hz, 2H), 3.86 (dd, *J* = 10.8, 5.7 Hz, 1H), 3.76 (dd, *J* = 10.8, 8.6 Hz, 1H), 2.46 (t, *J* = 2.4 Hz, 1H), 2.07 (s, 3H). ^{13}C NMR (100 MHz, Chloroform-*d*) δ 170.7, 129.7, 128.5, 92.3, 79.2, 74.9, 65.0, 60.1, 55.0, 21.2. HRMS (ESI) *m/z*: calcd. for $C_{10}H_{12}O_4$ Na^+ $(M + Na)^+$ 219.0628, found 219.0647; $[\alpha]_D^{16}$ = 98.721 (c = 0.266, DCM).

(3*S*,6*R*)-6-(4-fluorophenoxy)-3,6-dihydro-2*H*-pyran-3-yl acetate (8d)

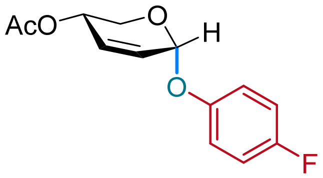

Colorless oil; yield 71%; ^{1}H NMR (400 MHz, Chloroform-*d*) δ 7.01 (ddd, *J* = 17.7, 9.4, 4.5 Hz, 4H), 6.09 (d, *J* = 10.3 Hz, 1H), 6.00 (dt, *J* = 10.3, 2.2 Hz, 1H), 5.56 (s, 1H), 5.43–5.34 (m, 1H), 3.96 (dd, *J* = 10.9, 5.8 Hz, 1H), 3.87 (dd, *J* = 10.8, 9.0 Hz, 1H), 2.09 (s, 3H). ^{13}C NMR (100 MHz, Chloroform-*d*) δ 170.6, 130.4, 127.8, 118.3, 118.2, 116.1, 115.9, 93.2, 64.8, 60.4, 21.1. HRMS (ESI) *m/z*: calcd. for $C_{13}H_{13}FO_4$ Na^+ $(M + Na)^+$ 275.0690, found 275.0685; $[\alpha]_D^{15}$ = 85.907 (c = 0.090, DCM).

(3*R*,6*S*)-6-ethoxy-3,6-dihydro-2*H*-pyran-3-yl acetate (8a)

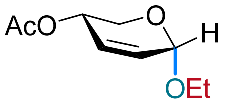

Colorless oil; yield 85%; ^{1}H NMR (400 MHz, Chloroform-*d*) δ 5.94 (ddt, *J* = 10.4, 2.2, 1.0 Hz, 1H), 5.87 (ddd, *J* = 10.3, 2.3, 1.7 Hz, 1H), 5.27 (ddq, *J* = 7.6, 5.8, 1.8 Hz, 1H), 4.95 (dt, *J* = 2.5, 1.3 Hz, 1H), 3.89–3.79 (m, 3H), 3.55 (dq, *J* = 9.5, 7.1 Hz, 1H), 2.07 (s, 3H), 1.24 (t, *J* = 7.1 Hz, 3H). ^{13}C NMR (100 MHz, Chloroform-*d*) δ 170.7,

129.6, 128.8, 94.3, 65.1, 64.3, 60.4, 21.2, 15.4. HRMS (ESI) *m/z*: calcd. for $C_9H_{14}O_4$ Na^+ $(M + Na)^+$ 209.0784, found 209.0790; $[\alpha]_D^{15}$ = 91.458 (c = 0.016, DCM).

(3*R*,6*R*)-6-(4-fluorophenoxy)-3,6-dihydro-2*H*-pyran-3-yl acetate (8d)

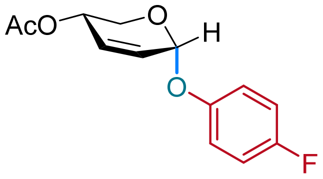

Colorless oil; yield 71%; ^{1}H NMR (400 MHz, Chloroform-*d*) δ 7.04–6.96 (m, 4H), 6.14–6.04 (m, 1H), 6.00 (dt, *J* = 10.3, 2.2 Hz, 1H), 5.56 (s, 1H), 5.38 (dtd, *J* = 9.4, 3.6, 1.7 Hz, 1H), 3.99–3.92 (m, 1H), 3.87 (dd, *J* = 10.7, 9.0 Hz, 1H), 2.09 (s, 3H). ^{13}C NMR (100 MHz, Chloroform-*d*) δ 170.6, 130.4, 127.8, 118.2, 118.1, 116.1, 115.9, 93.1, 64.7, 60.3, 21.1. HRMS (ESI) *m/z*: calcd. for $C_{13}H_{13}FO_4$ Na^+ $(M + Na)^+$ 275.0690, found 275.0685; $[\alpha]_D^{15}$ = 33.773 (c = 0.158, DCM).

((2*R*,3*S*,6*S*)-3-acetoxy-6-(hexadecyloxy)-3,6-dihydro-2*H*-pyran-2-yl) methyl acetate (9a) [68]

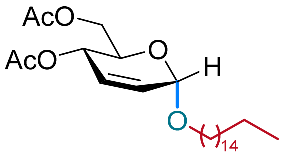

White solid; yield 90%; m. p.: 40.1-41.3 °C; ^{1}H NMR (400 MHz, Chloroform-*d*) δ 5.89–5.80 (m, 2H), 5.30 (dd, *J* = 9.8, 1.8 Hz, 1H), 5.01 (d, *J* = 2.5 Hz, 1H), 4.24 (dd, *J* = 12.1, 5.3 Hz, 1H), 4.16 (dd, *J* = 12.2, 2.3 Hz, 1H), 4.09 (ddd, *J* = 9.8, 5.4, 2.4 Hz, 1H), 3.75 (dt, *J* = 9.3, 6.8 Hz, 1H), 3.49 (dt, *J* = 9.4, 6.6 Hz, 1H), 2.09 (s, 3H), 2.07 (s, 3H), 1.59 (t, *J* = 7.2 Hz, 2H), 1.24 (s, 26H), 0.86 (t, *J* = 6.7 Hz, 3H). ^{13}C NMR (100 MHz, Chloroform-*d*) δ 170.9, 170.4, 129.1, 128.1, 94.5, 69.1, 66.9, 65.4, 63.1, 32.0, 29.8, 29.8, 29.8, 29.7, 29.7, 29.5, 29.5, 26.4, 22.8, 21.1, 20.9, 14.2. HRMS (ESI) *m/z*: calcd. for $C_{26}H_{46}O_6$ Na^+ $(M + Na)^+$ 477.3187, found 477.3179; $[\alpha]_D^{16}$ = 53.409 (c = 0.546, DCM).

((2*R*,3*S*,6*S*)-3-acetoxy-6-((3,3,4,4,5,5,6,6,7,7,8,8,9,9,10,10,10-heptadecafluorodecyl)oxy)-3,6-dihydro-2*H*-pyran-2-yl)methyl acetate (9b)

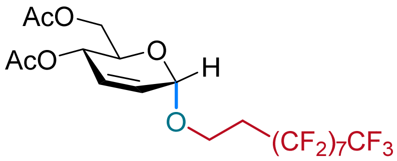

White solid; yield 87%; m. p.: 67.6-68.2 °C; ^{1}H NMR (400 MHz, Chloroform-*d*) δ 6.06–5.79 (m, 2H), 5.34–5.02 (m, 2H), 4.29–4.19 (m, 2H), 4.18–4.02 (m, 2H), 3.83 (m, 1H), 2.47 (ddt, *J* = 25.1, 18.5, 8.8 Hz, 2H), 2.10 (s, 3H), 2.09 (s, 3H). ^{13}C NMR (100 MHz, Chloroform-*d*) δ 171.0, 170.4, 129.9, 129.7, 127.3, 126.8, 95.4, 94.7, 73.0, 67.3, 65.3, 64.1, 63.4, 63.1, 60.7, 21.1, 20.8. HRMS (ESI) *m/z*: calcd. for $C_{20}H_{17}F_{17}O_6$ Na^+ $(M + Na)^+$ 699.0646, found 699.0634; $[\alpha]_D^{20}$ = 41.030 (c = 0.550, MeOH).

((2*R*,3*S*,6*R*)-3-acetoxy-6-(((2*R*,3*R*,4*R*,5*S*)-2-(2,2-dimethyl-1,3-dioxolan-4-yl)-4-isopropoxy-5-methoxytetrahydrofuran-3-yl)oxy)-3,6-dihydro-2*H*-pyran-2-yl)methyl acetate (9c) [53]

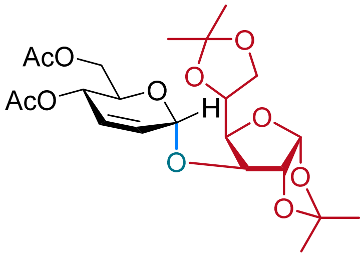

Colorless oil, yield 80%; ^{1}H NMR (400 MHz, Chloroform-*d*) δ 5.92 (dd, *J* = 7.0, 3.4 Hz, 2H), 5.85 (dt, *J* = 10.2, 2.3 Hz, 1H), 5.29 (d, *J* = 8.1 Hz, 2H), 4.65 (d, *J* = 3.5 Hz, 1H), 4.35 (d, *J* = 2.9 Hz, 1H), 4.28–4.09 (m, 6H), 4.01 (dd, *J* = 8.5, 5.0 Hz, 1H), 2.15 (s, 3H), 2.12 (s, 3H), 1.52 (s, 3H), 1.43 (s, 3H), 1.34 (d, *J* = 5.0 Hz, 6H). ^{13}C NMR (100 MHz, Chloroform-*d*) δ 171.0, 170.4, 129.5, 127.4, 112.2, 109.3, 105.5, 95.4, 84.4, 81.4, 81.3, 72.7, 67.9, 67.3, 65.6, 27.1, 27.0, 26.6, 25.5, 21.1, 20.9. HRMS (ESI) *m/z*: calcd. for $C_{23}H_{36}O_{11}$ Na^+ $(M + Na)^+$ 495.1837, found 495.1831; $[\alpha]_D^{14}$ = 42.710 (c = 0.174, MeOH).

((2*R*,3*S*,6*R*)-3-acetoxy-6-(4-(1,2,2-triphenylvinyl)phenoxy)-3,6-dihydro-2*H*-pyran-2-yl)methyl acetate (9d)

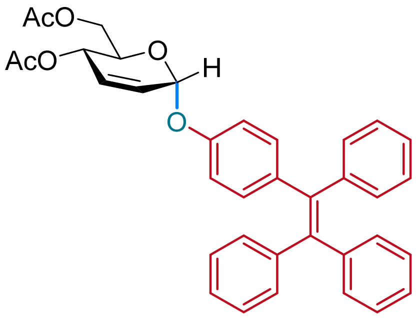

Colorless crystal; yield 69%; m. p.: 32.5 °C; ^{1}H NMR (400 MHz, Chloroform-*d*) δ 7.16–7.09 (m, 9H), 7.05 (ddt, *J* = 9.7, 5.5, 2.2 Hz, 6H), 6.97 (d, *J* = 8.8 Hz, 2H), 6.86 (d, *J* = 8.8 Hz, 2H), 6.00 (q, *J* = 9.8 Hz, 2H), 5.65 (s, 1H), 5.39 (d, *J* = 9.6 Hz, 1H), 4.32 (dd, *J* = 12.0, 5.7 Hz, 1H), 4.26–4.19 (m, 1H), 4.12 (dd, *J* = 12.0, 2.1 Hz, 1H), 2.13 (s, 3H), 1.99 (s, 3H). ^{13}C NMR (100 MHz, Chloroform-*d*) δ 170.9, 170.4, 155.7, 144.0, 140.6, 140.4, 138.0, 132.2, 127.9, 127.7, 127.2, 126.5, 126.5, 116.2, 93.0, 67.8, 65.1, 62.7, 21.1, 21.0. HRMS (ESI) *m/z*: calcd. for $C_{36}H_{32}O_6$ Na^+ $(M + Na)^+$ 583.2091, found 583.2082; $[\alpha]_D^{20}$ = 124.890 (c = 0.266, MeOH).

((2*R*,3*S*,6*S*)-3-acetoxy-6-(((4*S*,5'*R*,6a*R*,6b*S*,8a*S*,8b*R*,9*S*,10*R*,11a*S*,12a*S*,12b*S*)-5',6a,8a,9-tetramethyl-1,3,3',4,4',5,5',6,6a,6b,6',7,8,8a,8b,9,11a,12,12a,12b-icosahydrospiro[naphtho[2',1':4,5]indeno[2,1-b]furan-10,2'-pyran]-4-yl)oxy)-3,6-dihydro-2*H*-pyran-2-yl)methyl acetate (9e)

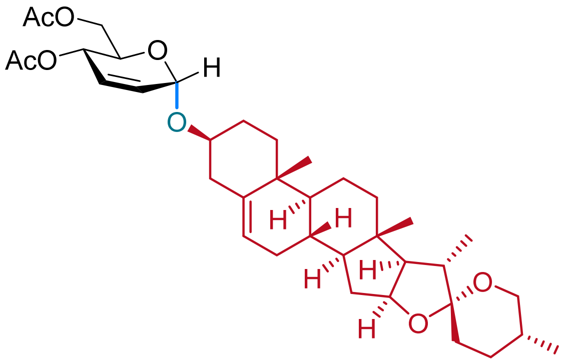

White solid; yield 65%; m. p.: 63.4-64.7 °C; ^{1}H NMR (400 MHz, Chloroform-*d*) δ 6.01–5.81 (m, 2H), 5.37 (dq, *J* = 5.4, 3.1, 2.7 Hz, 1H), 5.34–5.27 (m, 1H), 5.25–5.17 (m, 1H), 4.43 (q, *J* = 7.4 Hz, 1H), 4.26–4.17 (m, 2H), 3.61 (dtt, *J* = 27.4, 11.2, 4.6 Hz, 1H), 3.49 (ddd, *J* = 10.8, 4.4, 1.9 Hz, 1H), 3.39 (t, *J* = 10.9 Hz, 1H), 2.50–2.27 (m, 2H), 2.12 (s, 3H), 2.12 (s, 3H), 2.01 (ddt, *J* = 12.4, 7.4, 4.7 Hz, 2H), 1.90 (dq, *J* = 14.2,

5.7, 4.9 Hz, 3H), 1.78 (ddd, *J* = 14.1, 9.0, 5.2 Hz, 2H), 1.72–1.65 (m, 3H), 1.61 (dt, *J* = 12.0, 2.2 Hz, 3H), 1.57–1.42 (m, 4H), 1.38–1.24 (m, 2H), 1.25–1.08 (m, 3H), 1.04 (s, 3H), 0.99 (d, *J* = 7.0 Hz, 4H), 0.81 (t, *J* = 3.2 Hz, 6H). ^{13}C NMR (100 MHz, Chloroform-*d*) δ 171.0, 170.5, 140.9, 131.2, 129.0, 128.5, 126.1, 121.7, 109.4, 92.9, 81.0, 78.2, 67.0, 65.5, 63.3, 62.2, 56.6, 50.2, 41.7, 40.5, 40.4, 39.9, 37.2, 37.0, 32.2, 32.0, 31.6, 31.5, 30.4, 28.9, 28.4, 21.1, 21.0, 21.0, 19.5, 17.3, 16.4, 14.7. HRMS (ESI) *m/z*: calcd. for $C_{37}H_{54}O_8$ Na^+ $(M + Na)^+$ 649.3711, found 649.3701; $[\alpha]_D^{20}$ = 17.354 (c = 0.002, MeOH).

Section E: NMR Spectra

^{1}H NMR of Glucal 1a

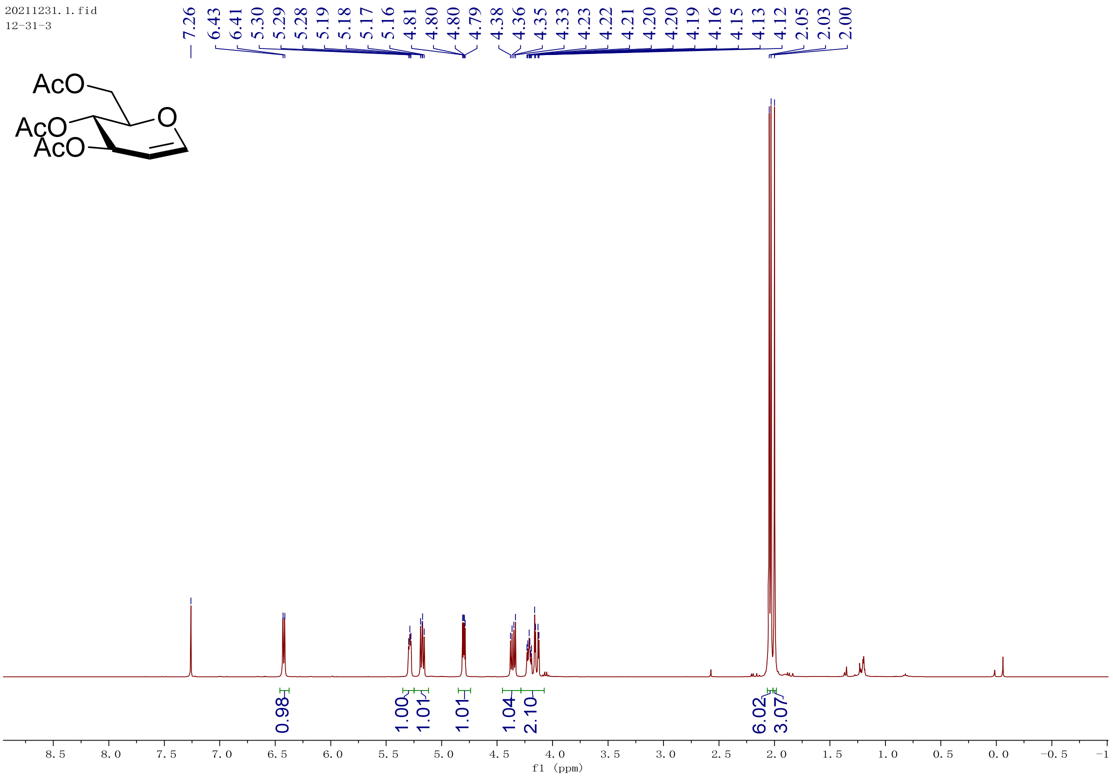

^{13}C NMR of Glucal 1a

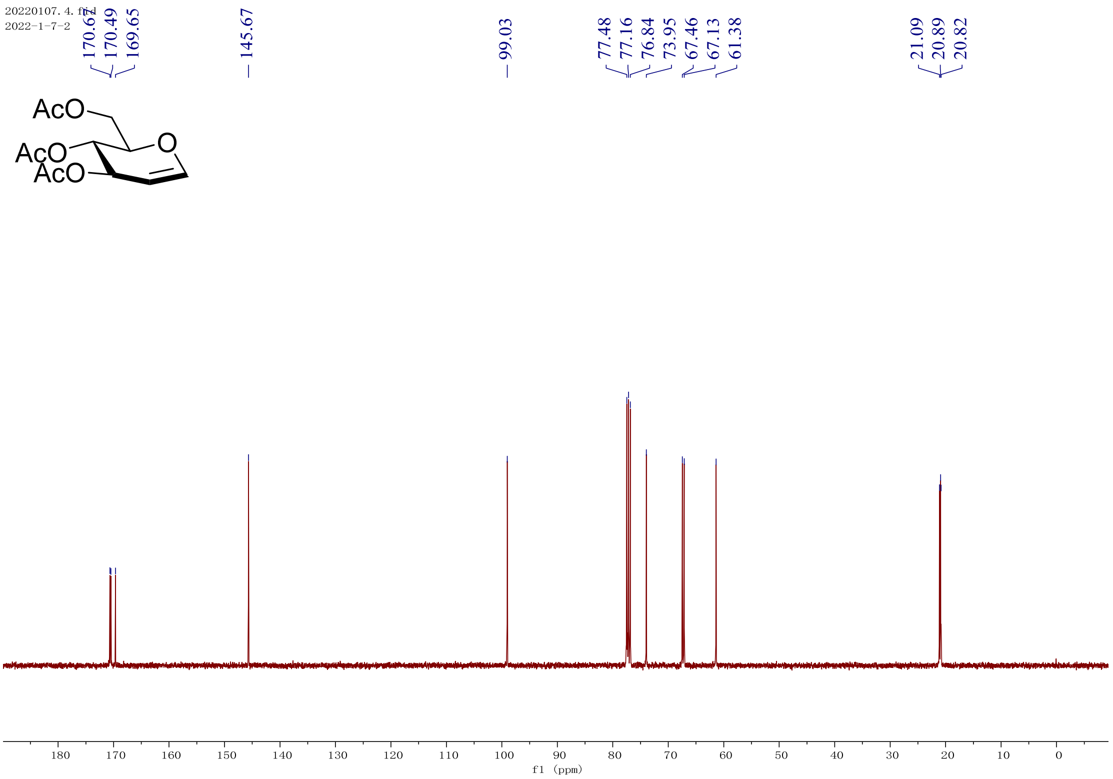

^{1}H NMR of Galactal 1b

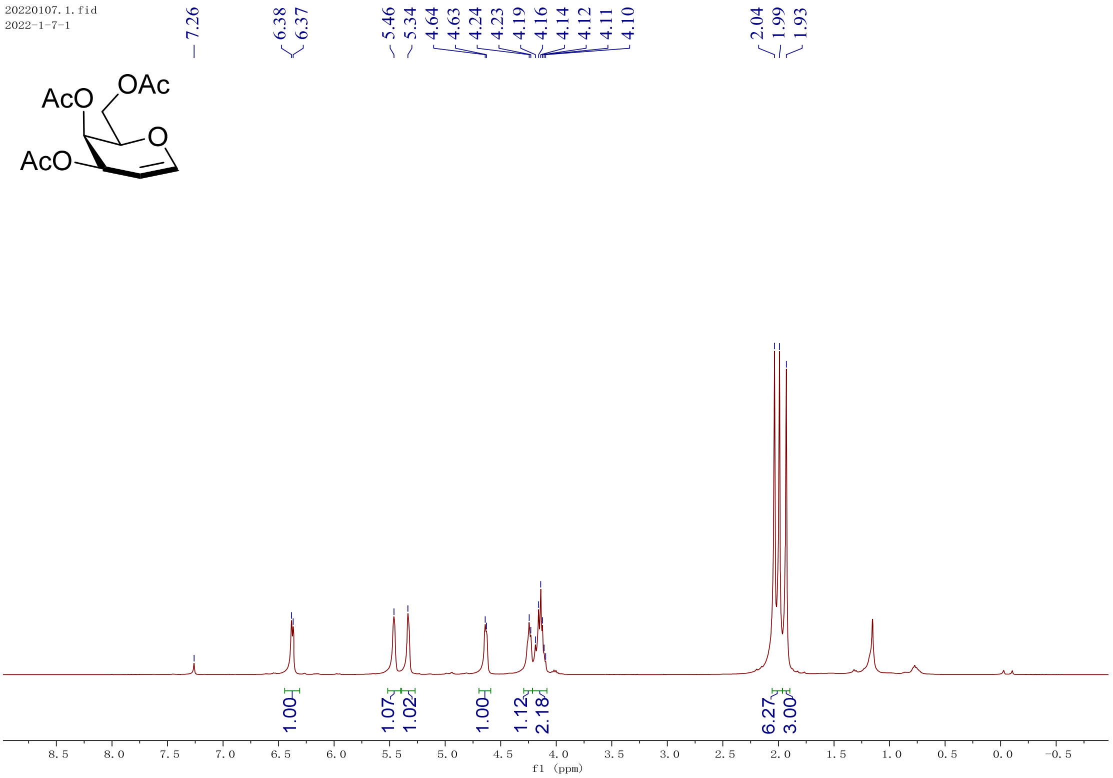

^{13}C NMR of Galactal 1b

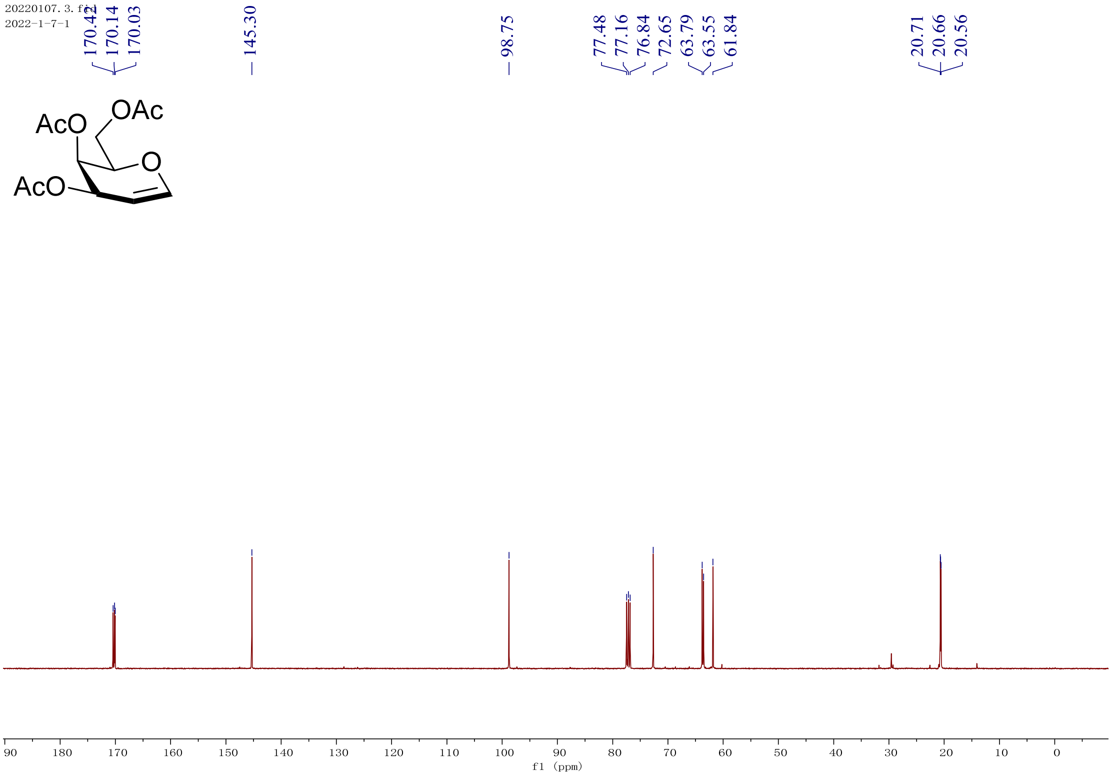

^{1}H NMR of Allal 1c

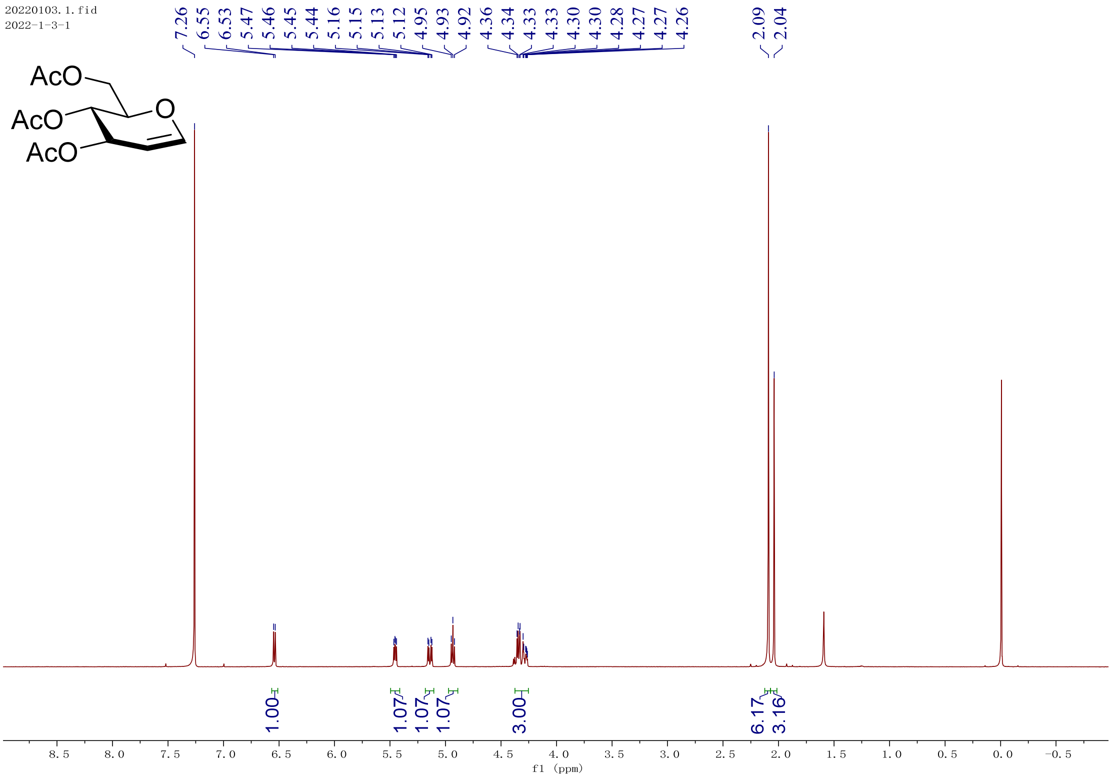

^{13}C NMR of Allal 1c

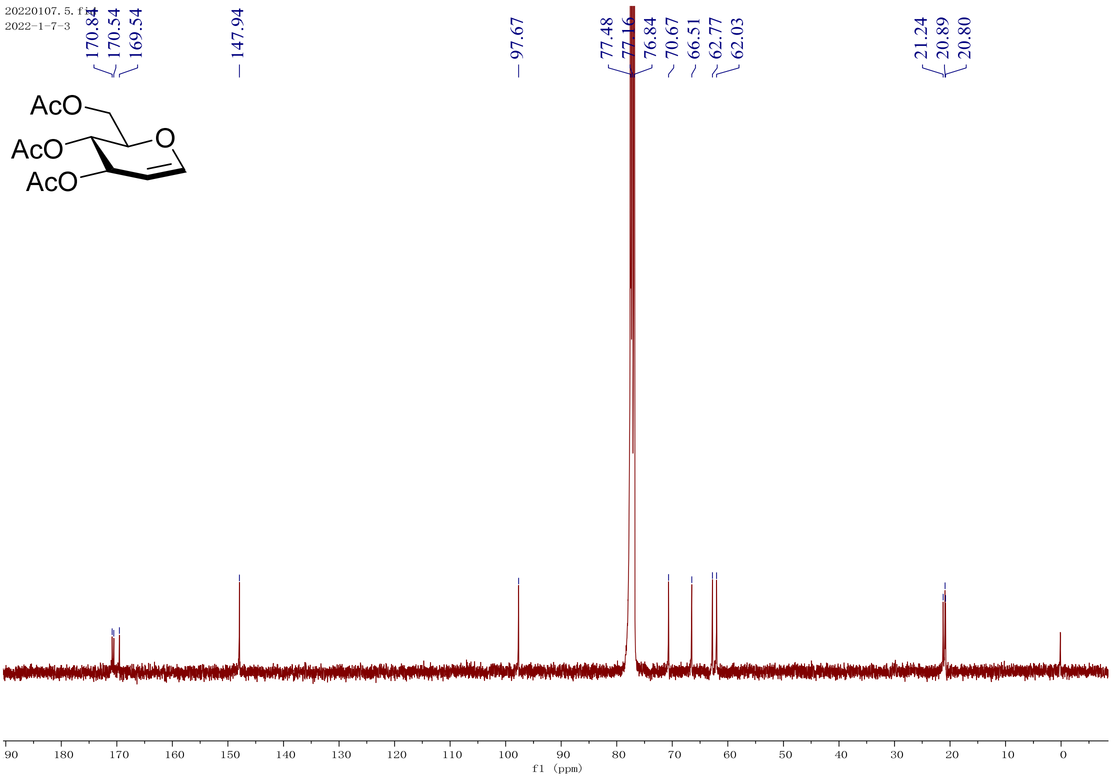

^{1}H NMR of L-Rhamnal 1d

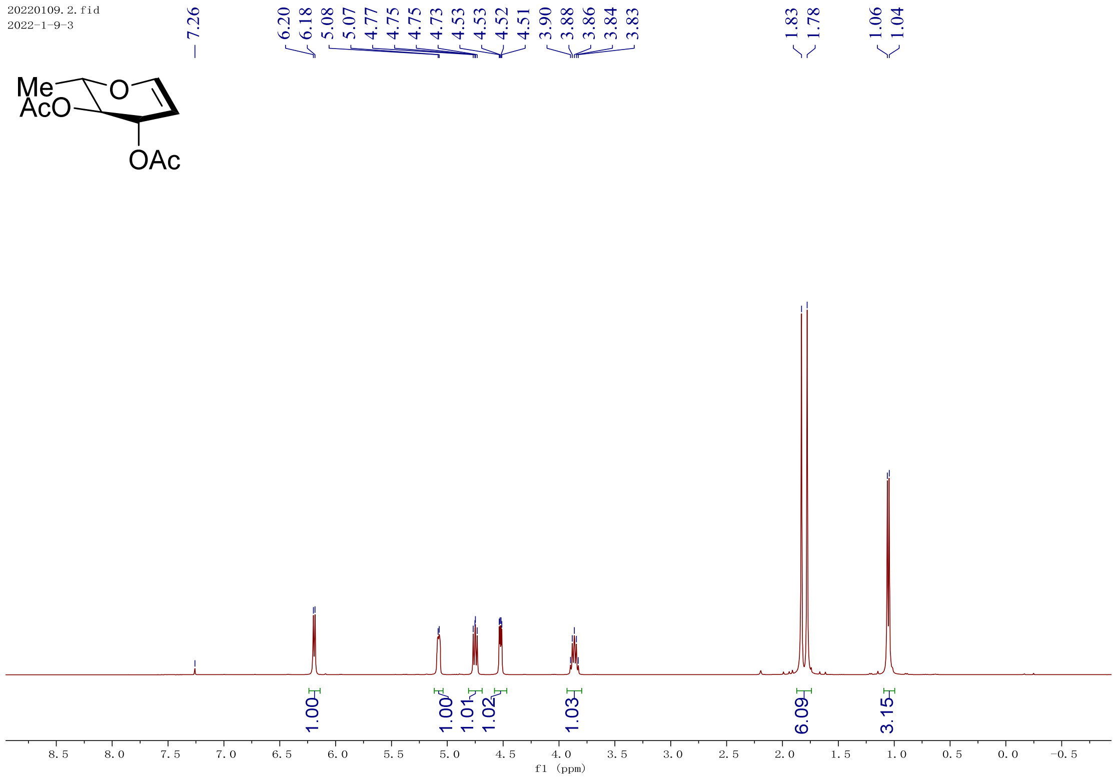

^{13}C NMR of L-Rhamnal 1d

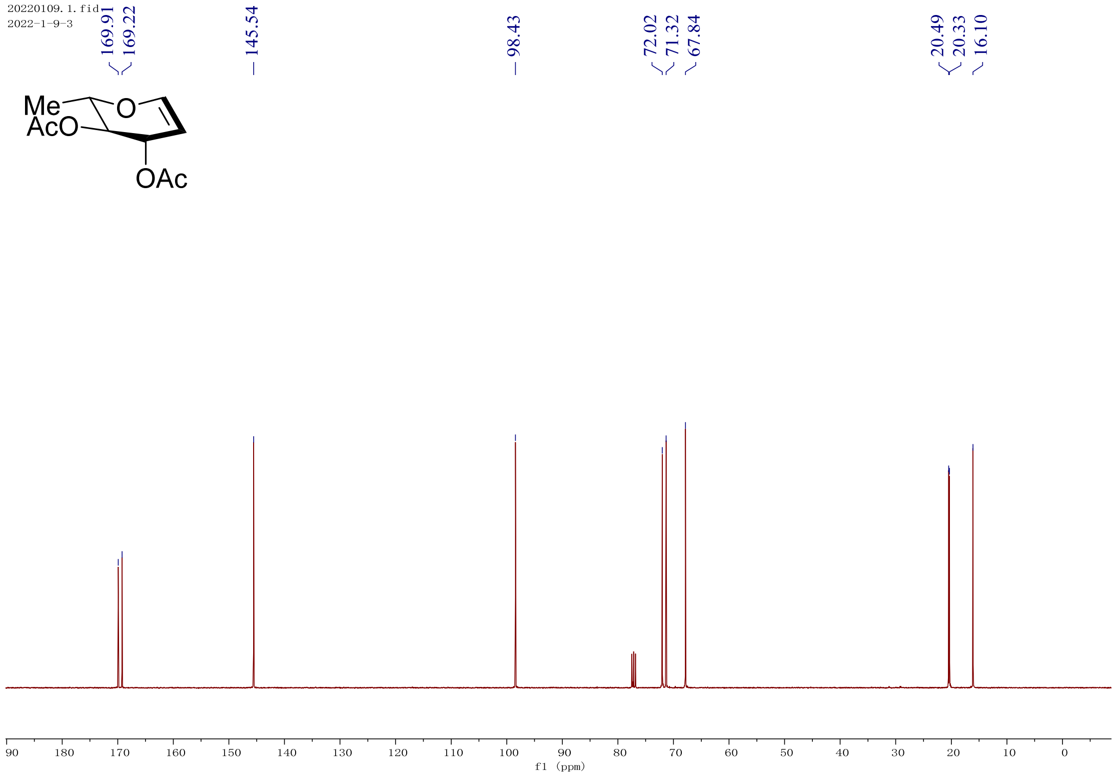

^{1}H NMR of Xylal 1e

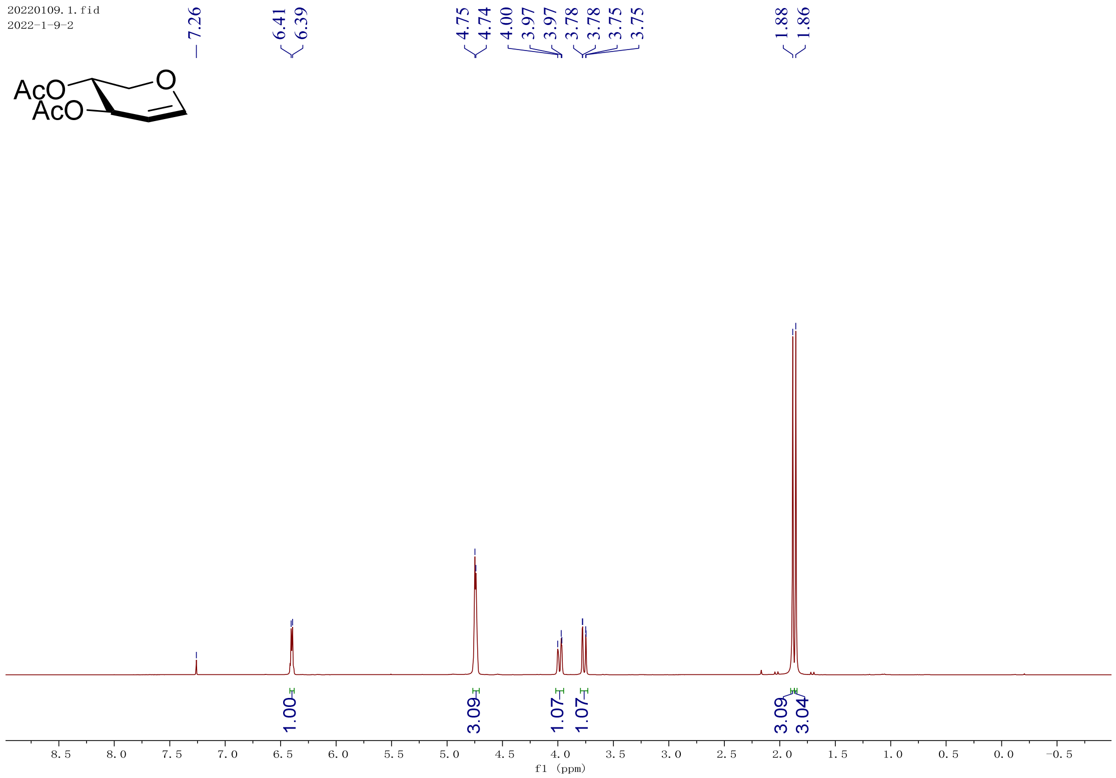

^{13}C NMR of Xylal 1e

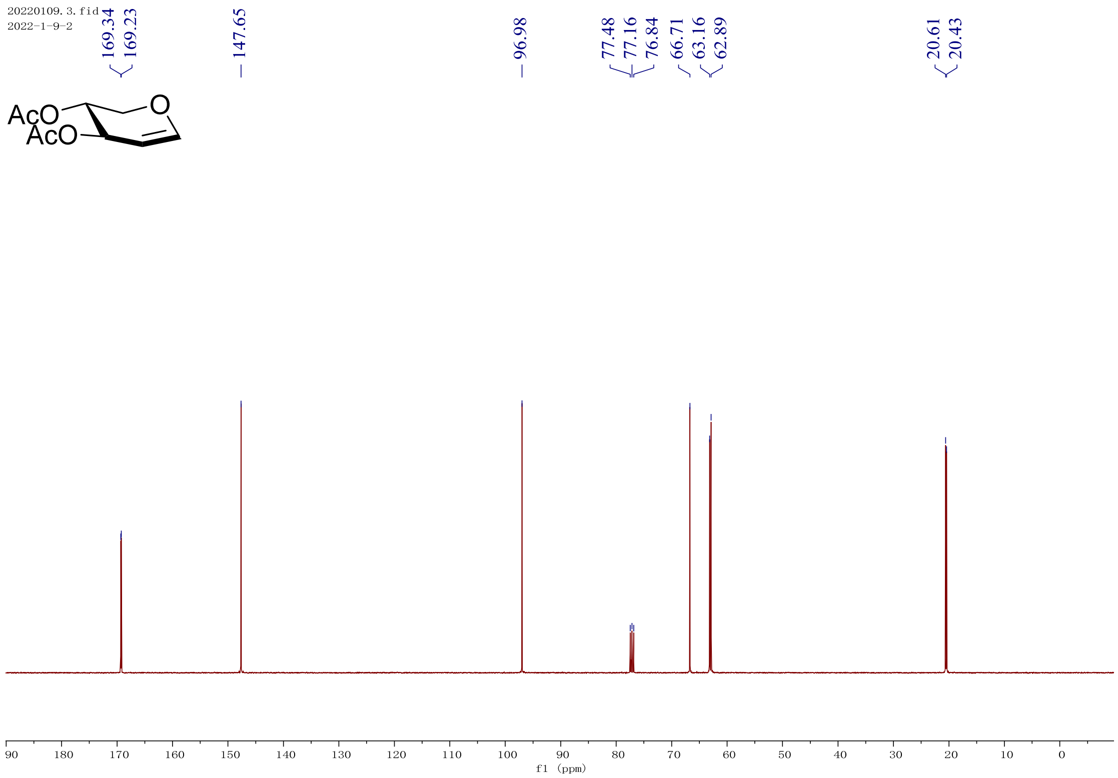

^{1}H NMR of Arabinal 1f

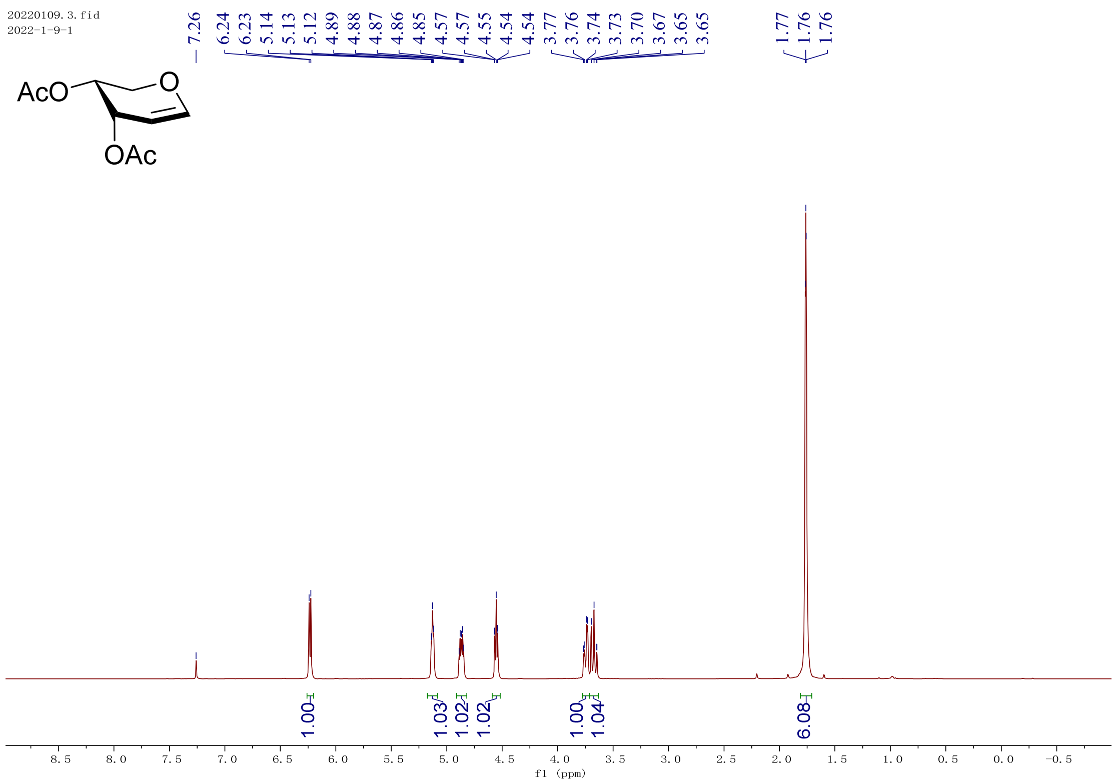

^{13}C NMR of Arabinal 1f

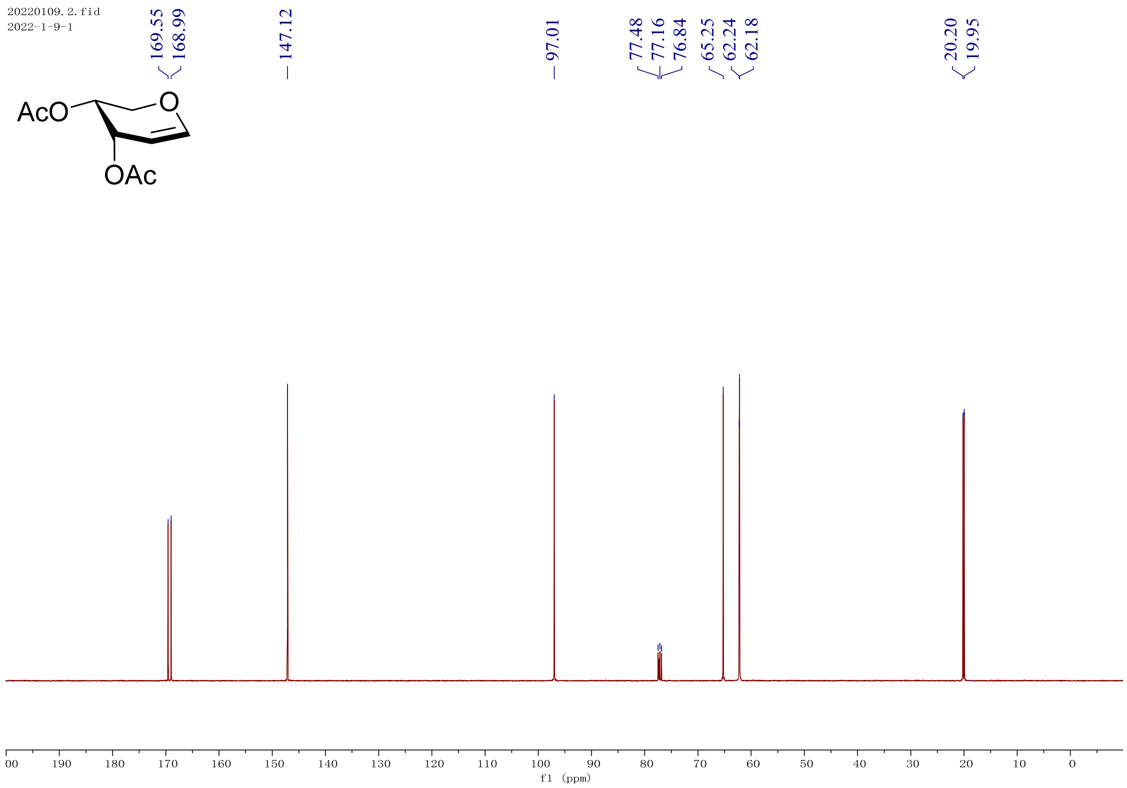

^{1}H NMR of 3a

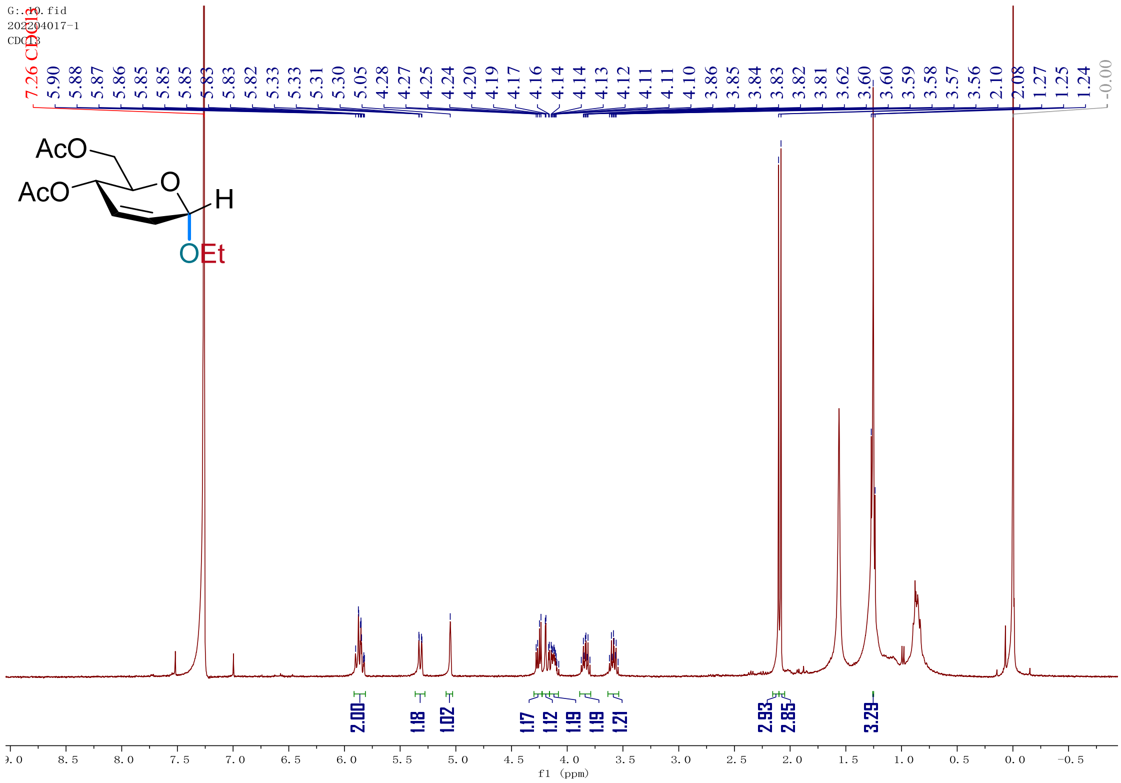

^{13}C NMR of 3a

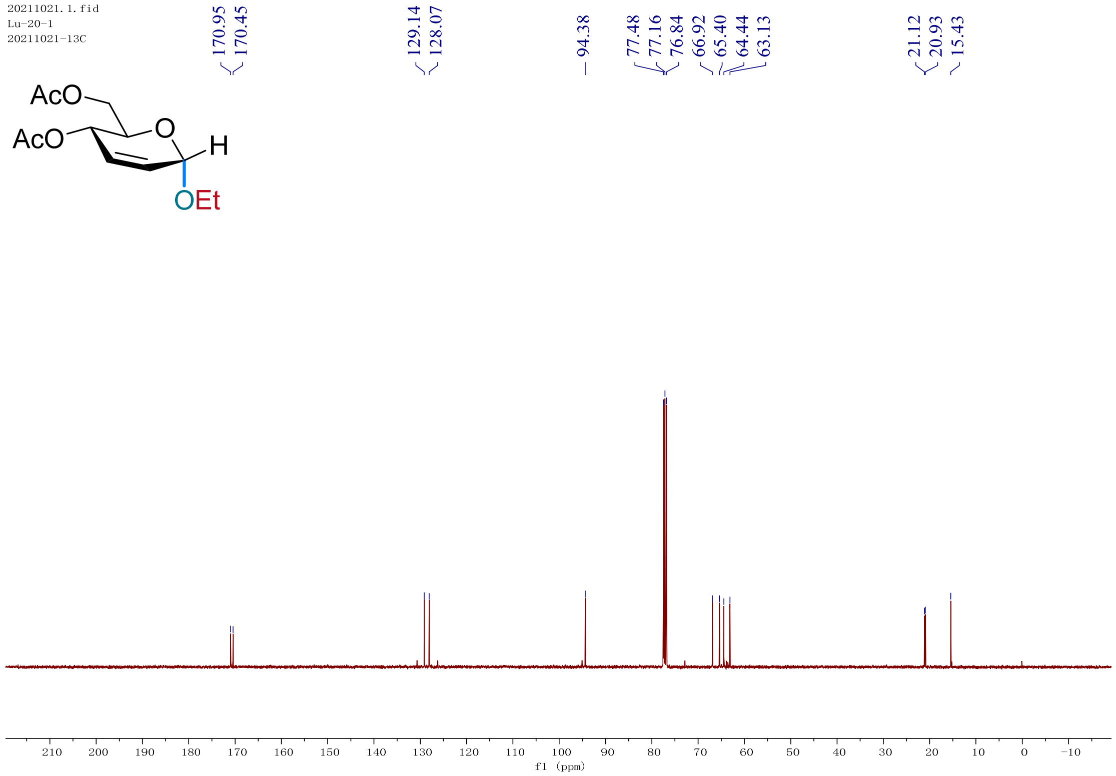

^{1}H NMR of 3b

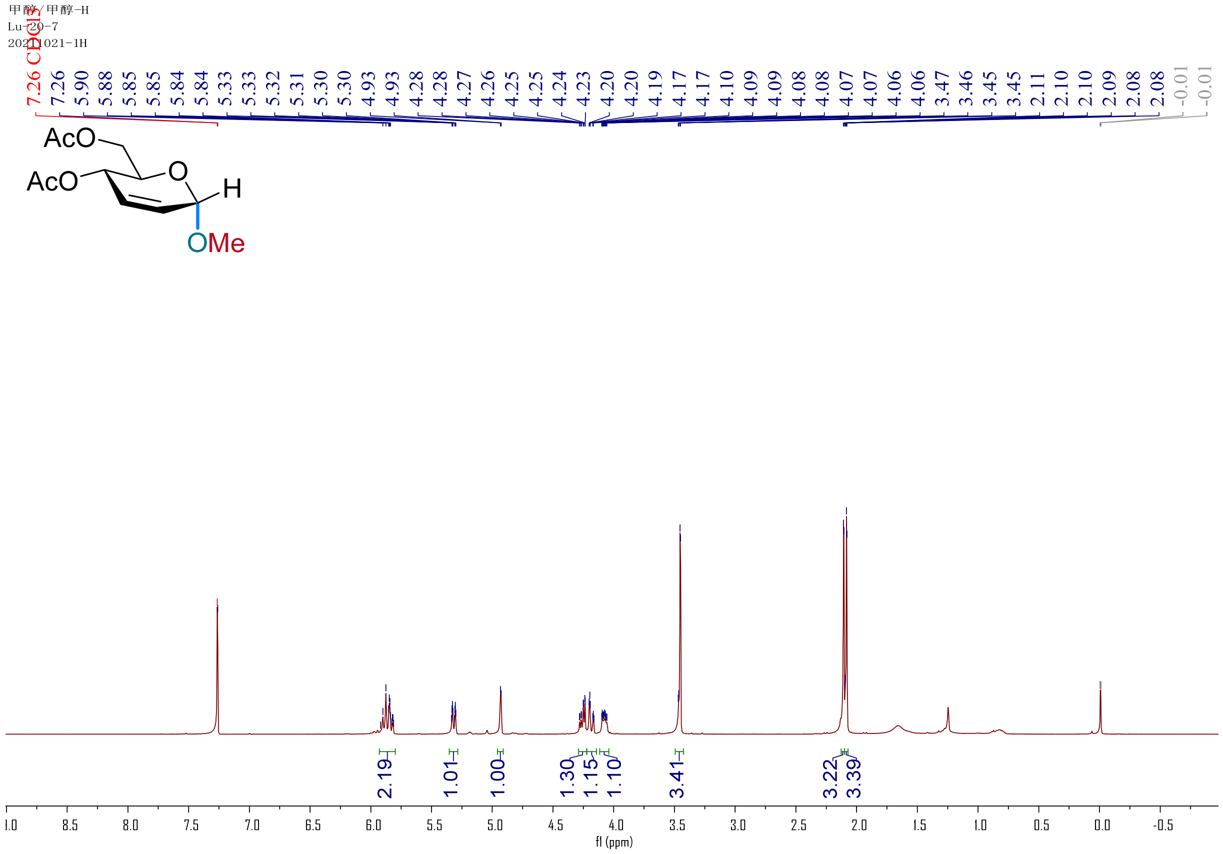

^{13}C NMR of 3b

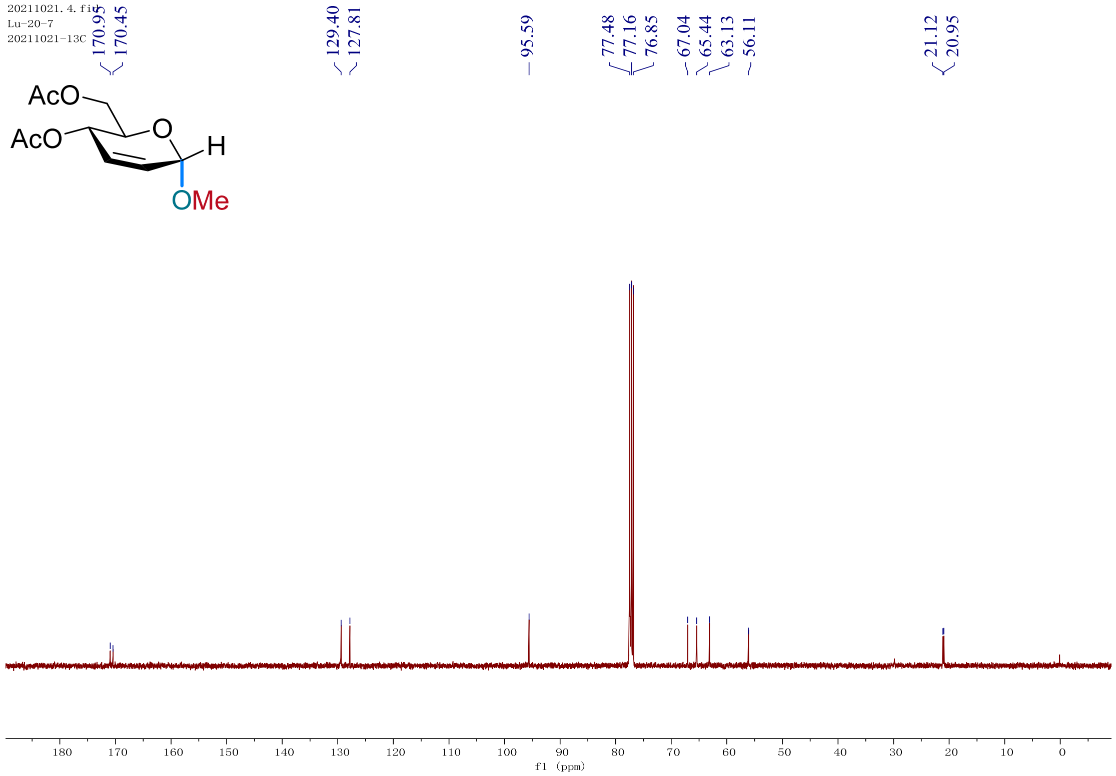

^{1}H NMR of 3c

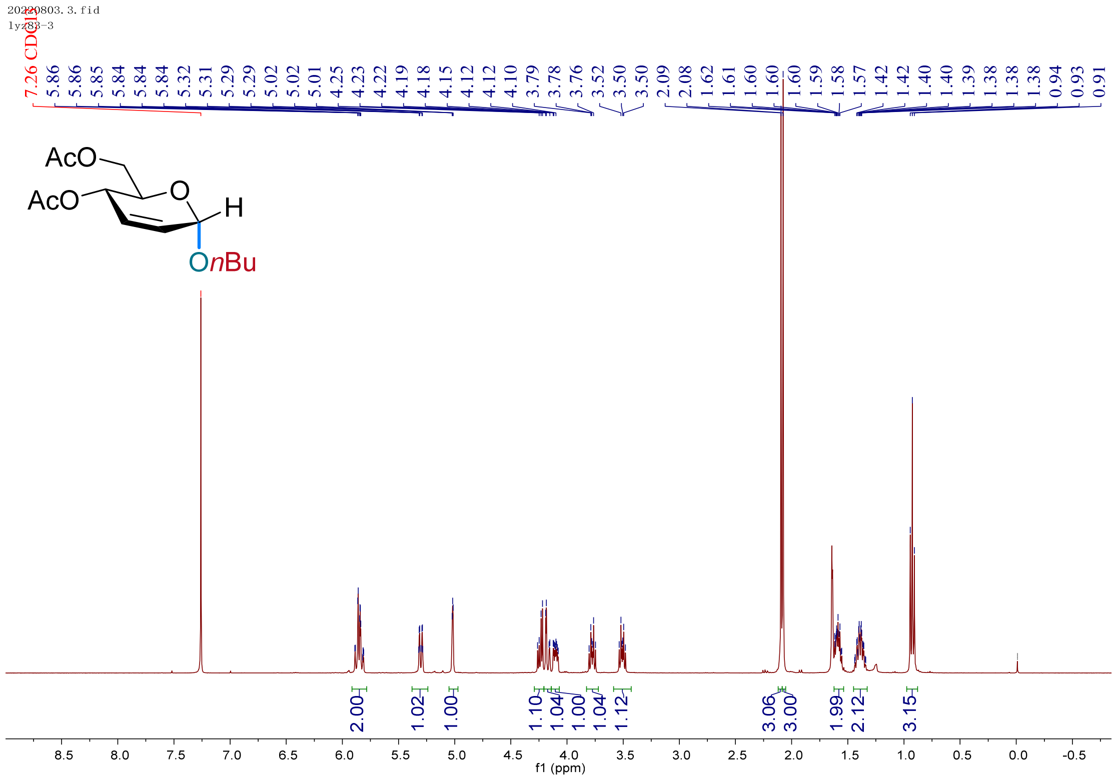

^{13}C NMR of 3c

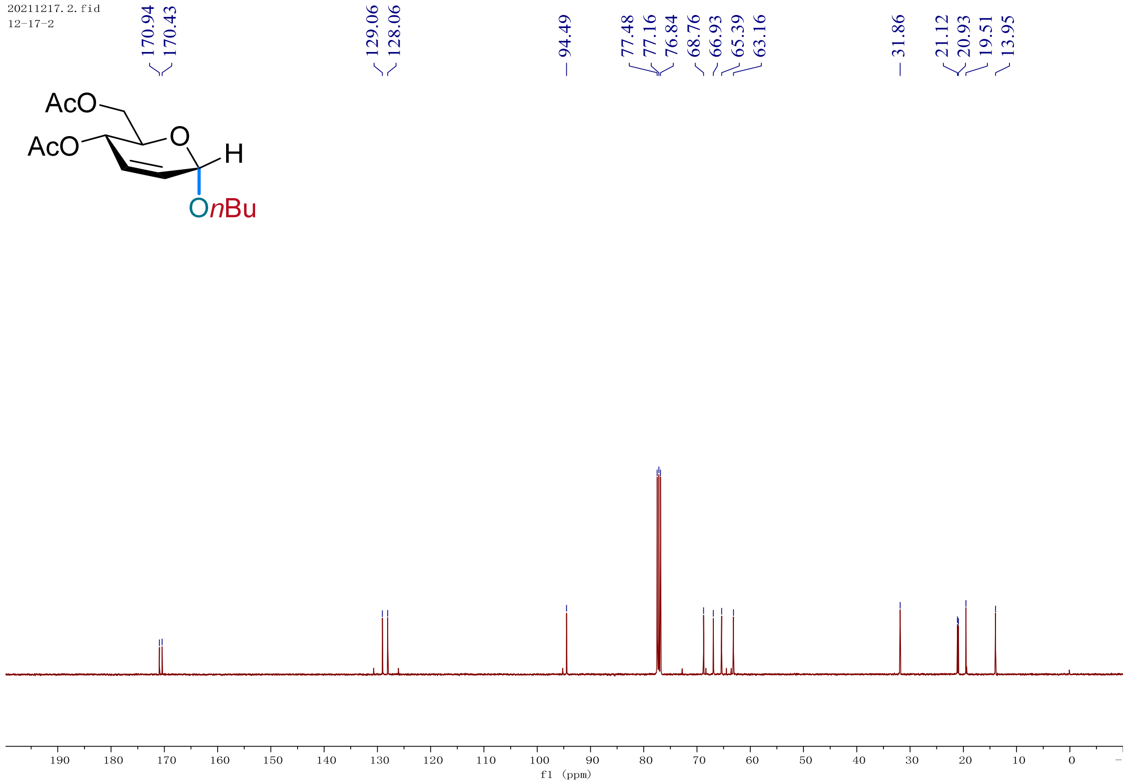

^{1}H NMR of 3d

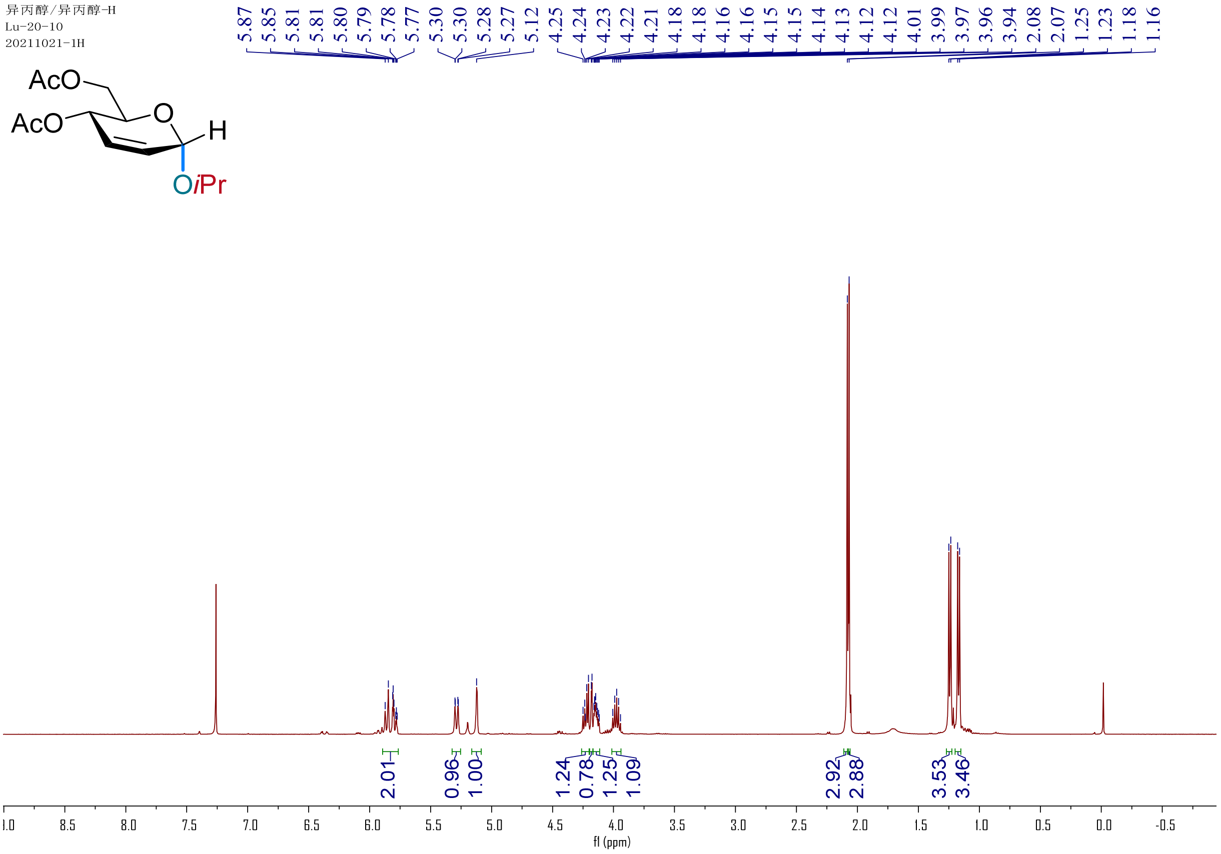

^{13}C NMR of 3d

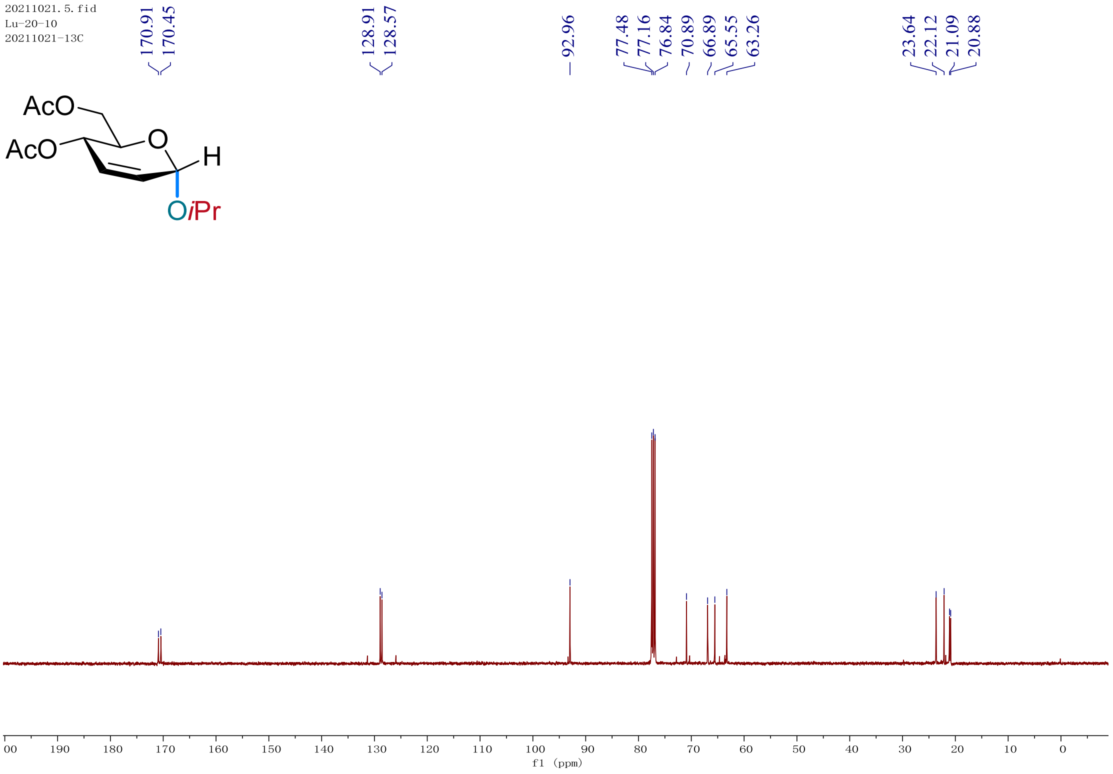

^{1}H NMR of 3e

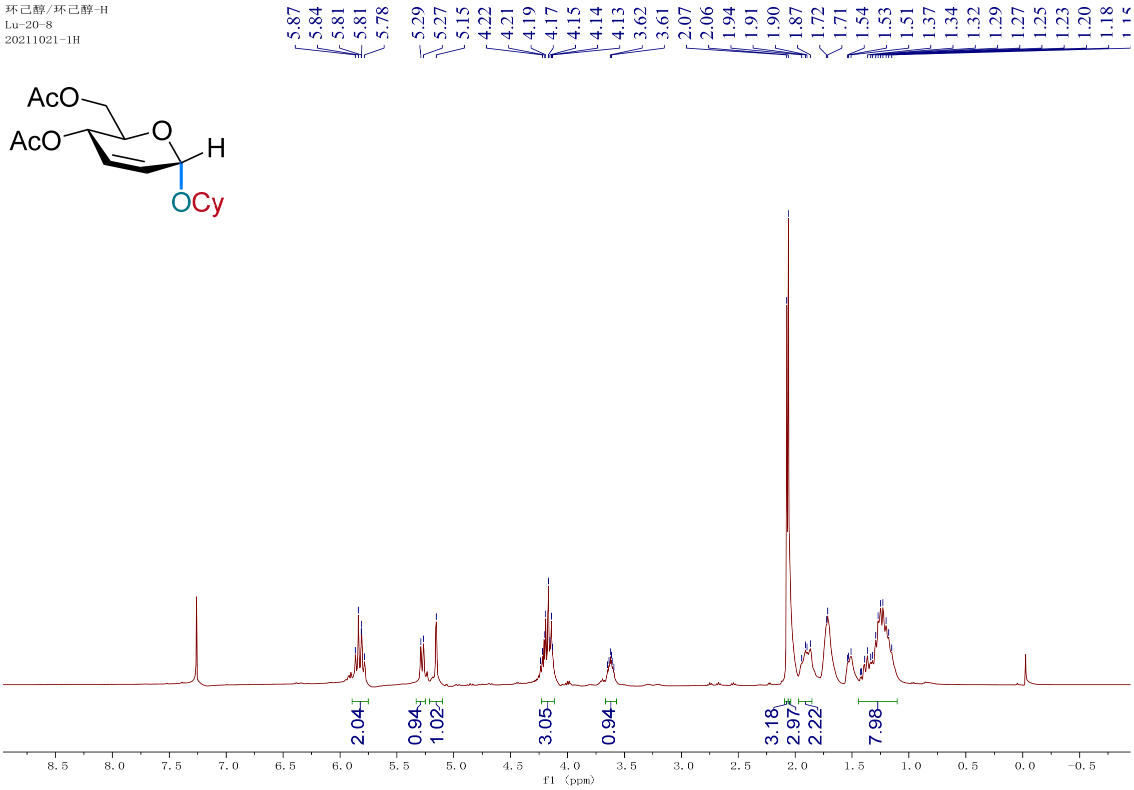

^{13}C NMR of 3e

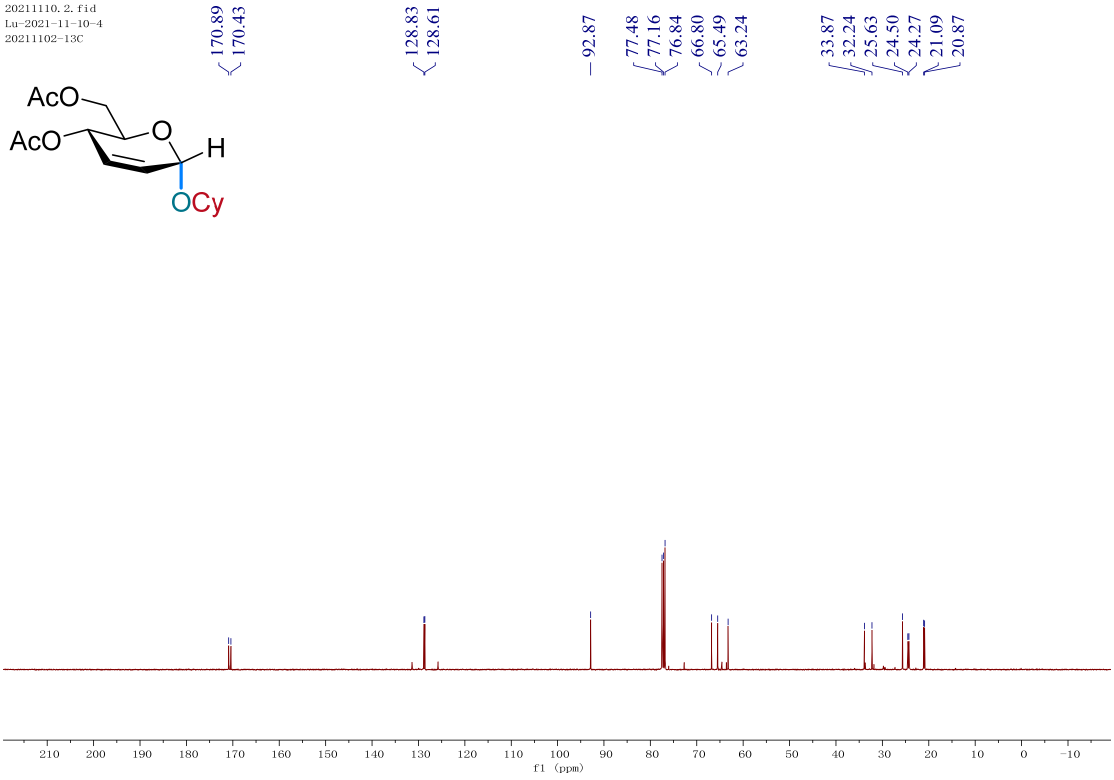

^{1}H NMR of 3f

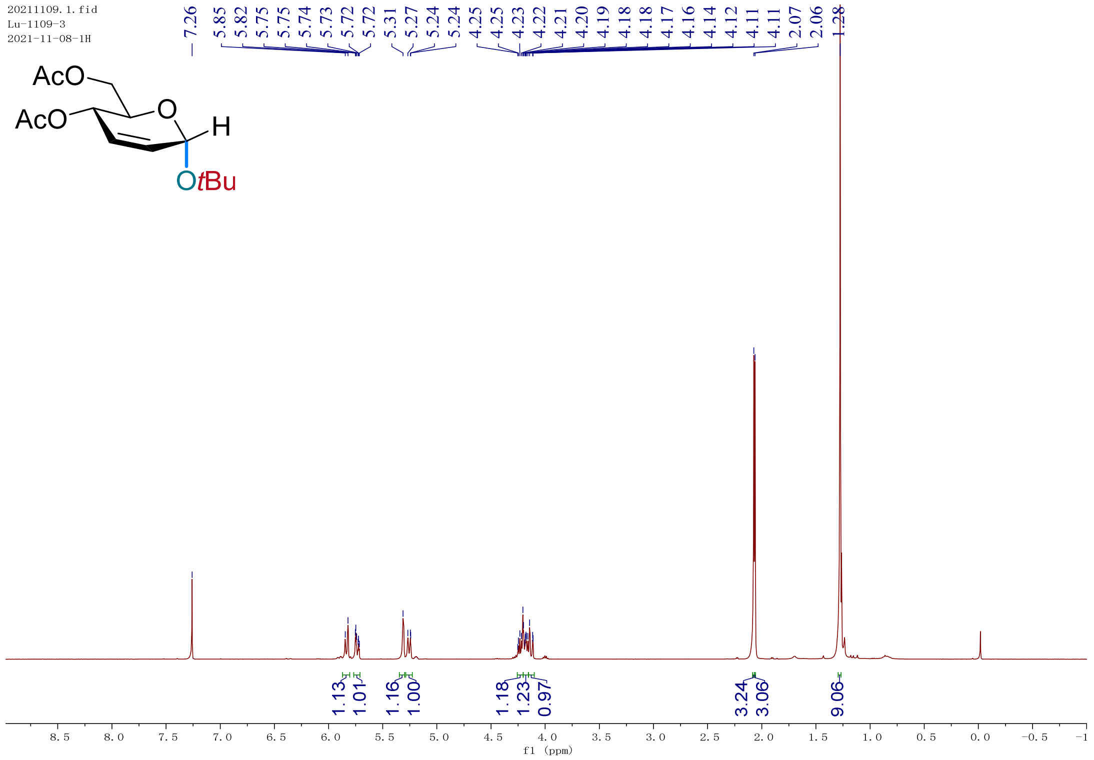

^{13}C NMR of 3f

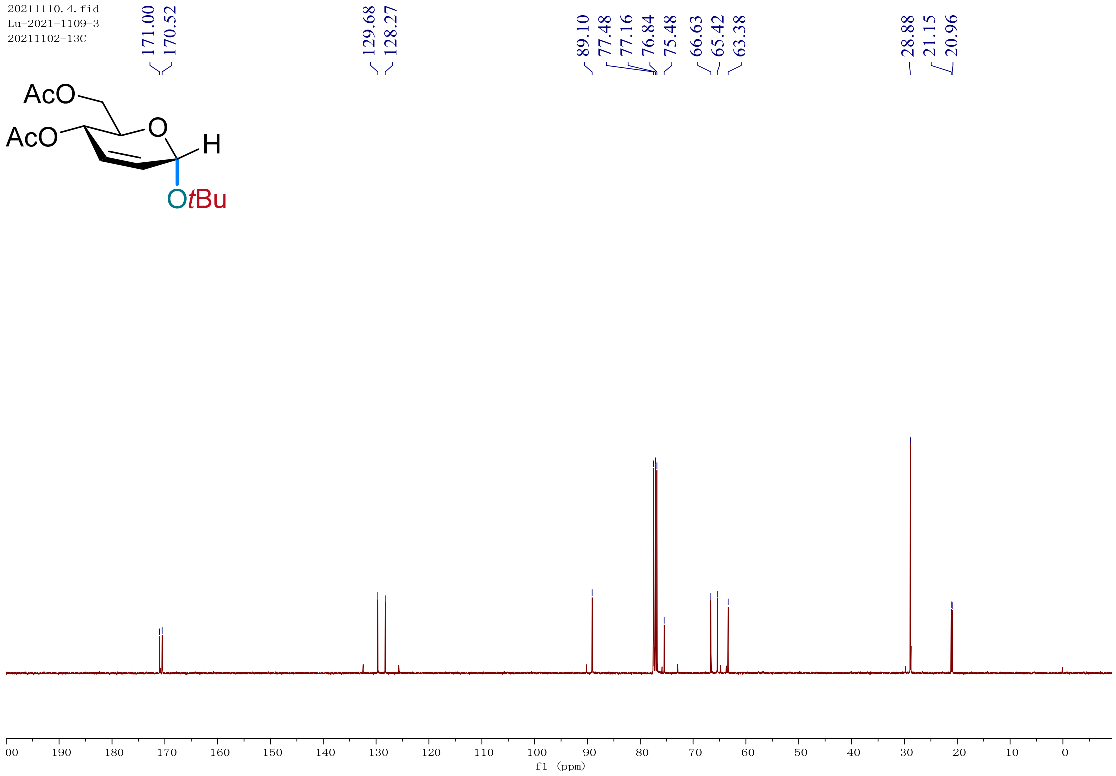

^{1}H NMR of 3g

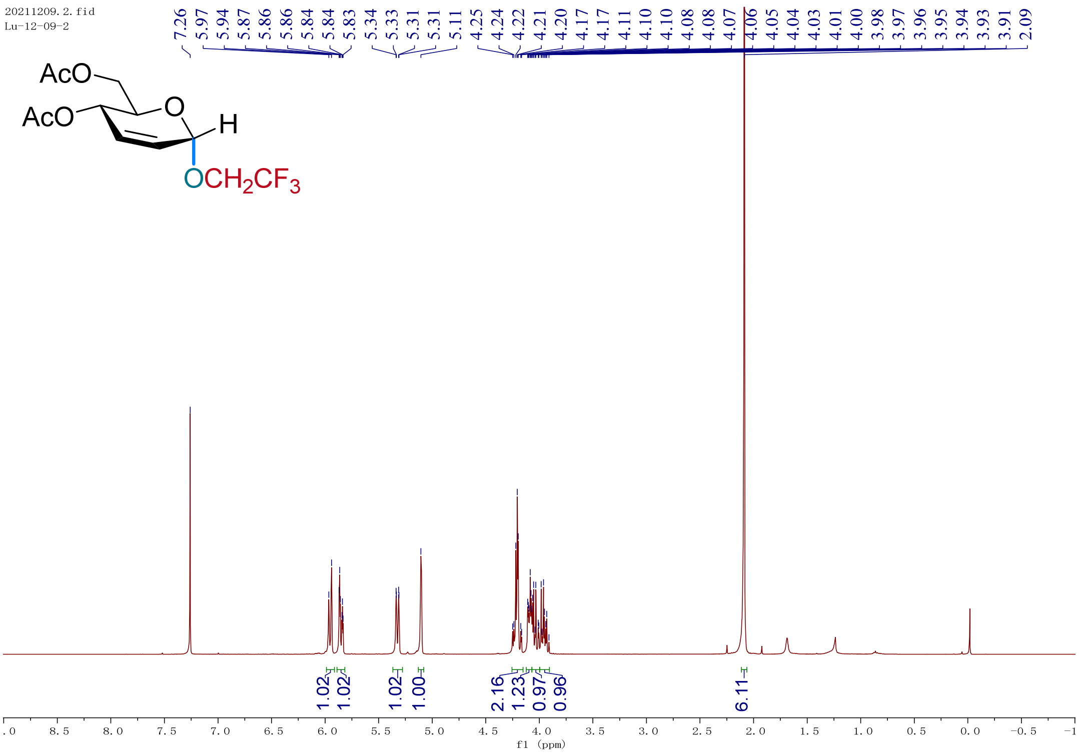

^{13}C NMR of 3g

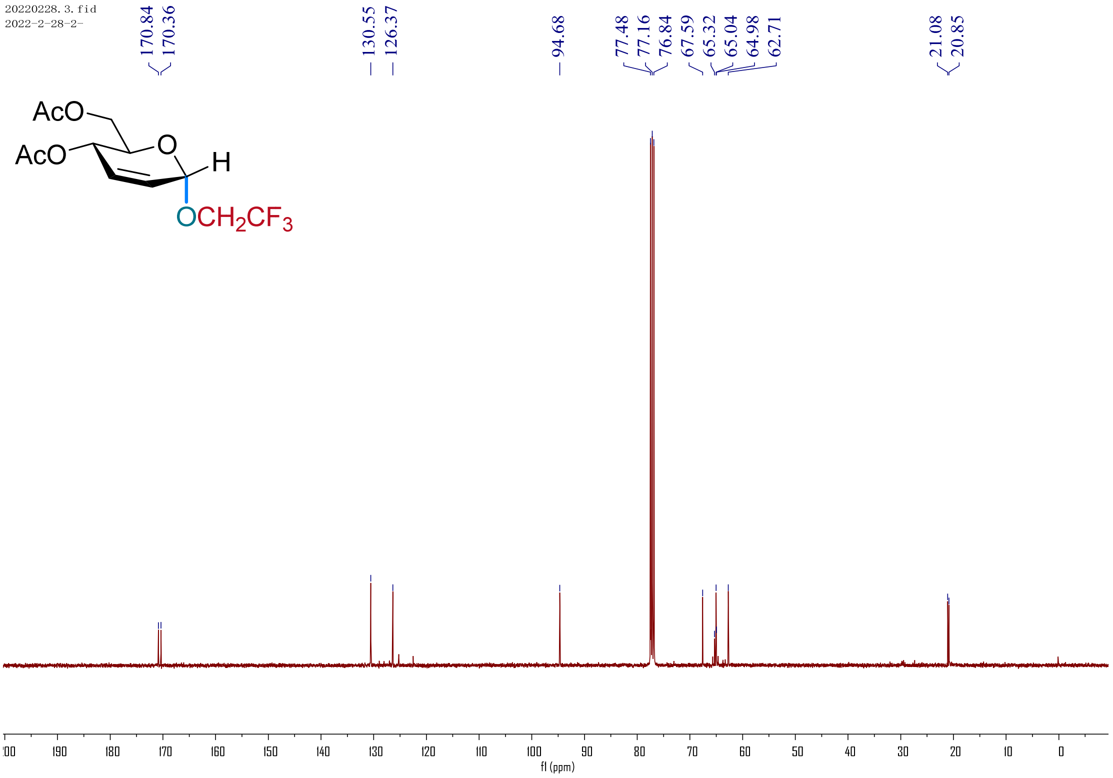

^{1}H NMR of 3h

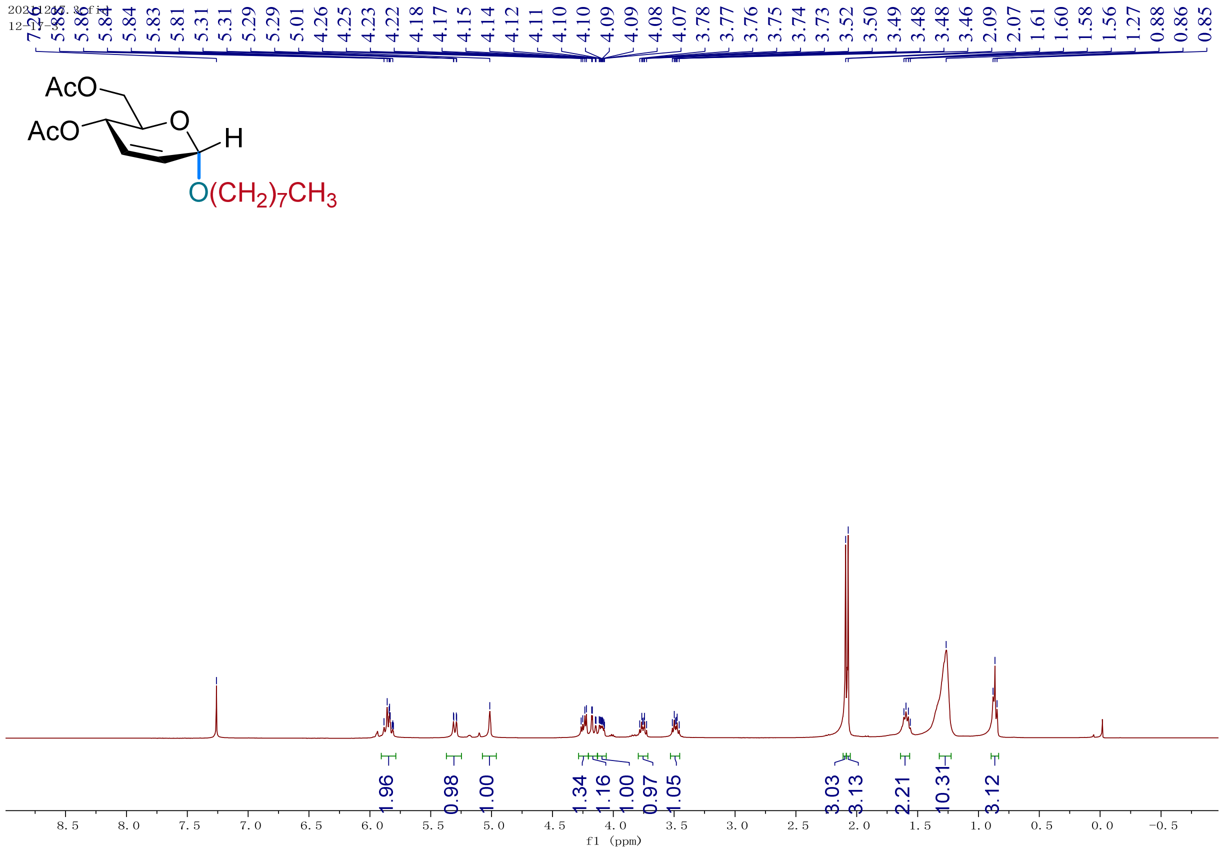

^{13}C NMR of 3h

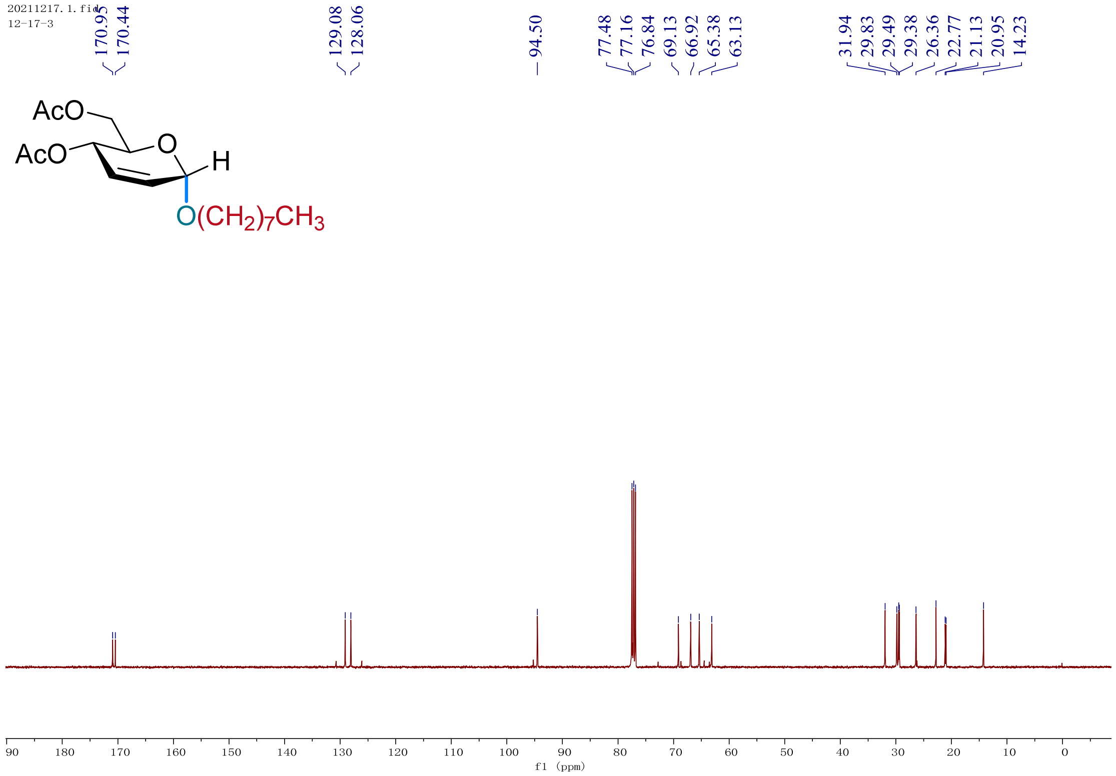

^{1}H NMR of 3i

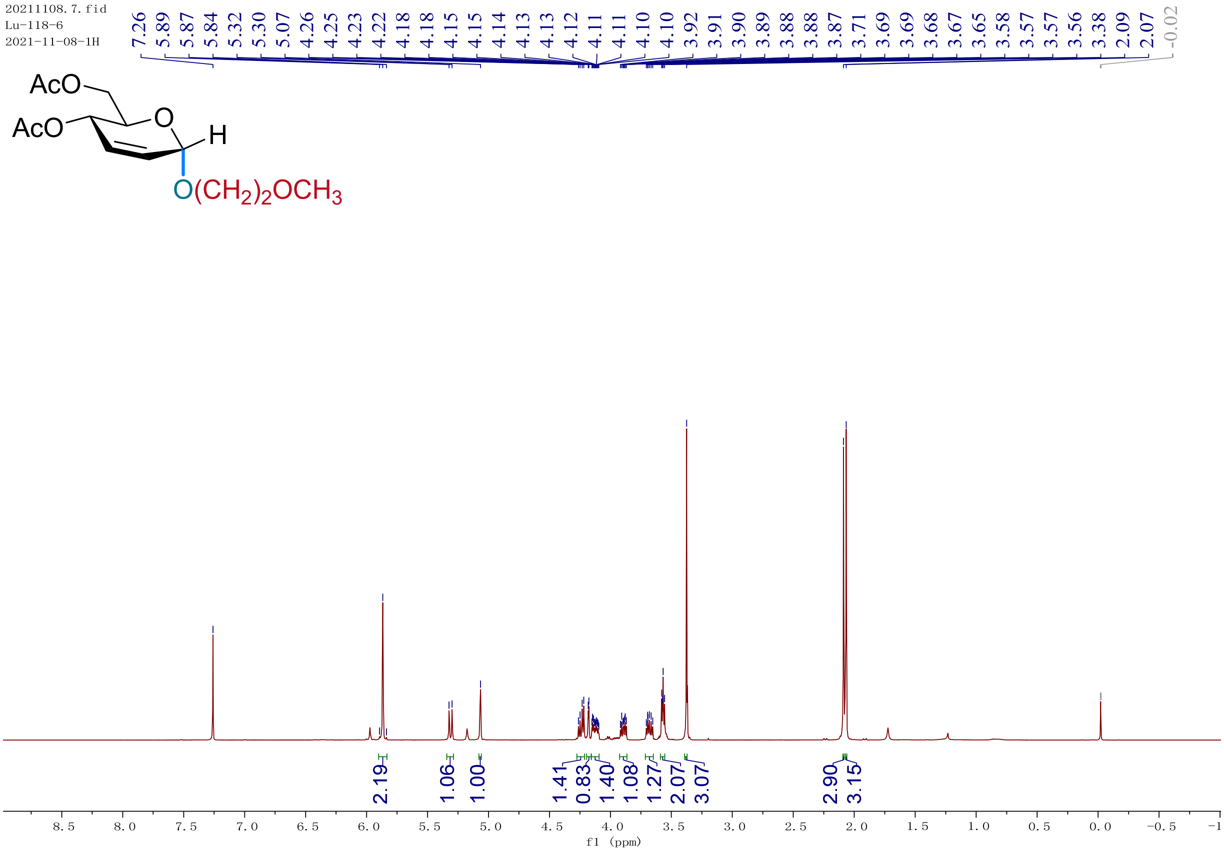

^{13}C NMR of 3i

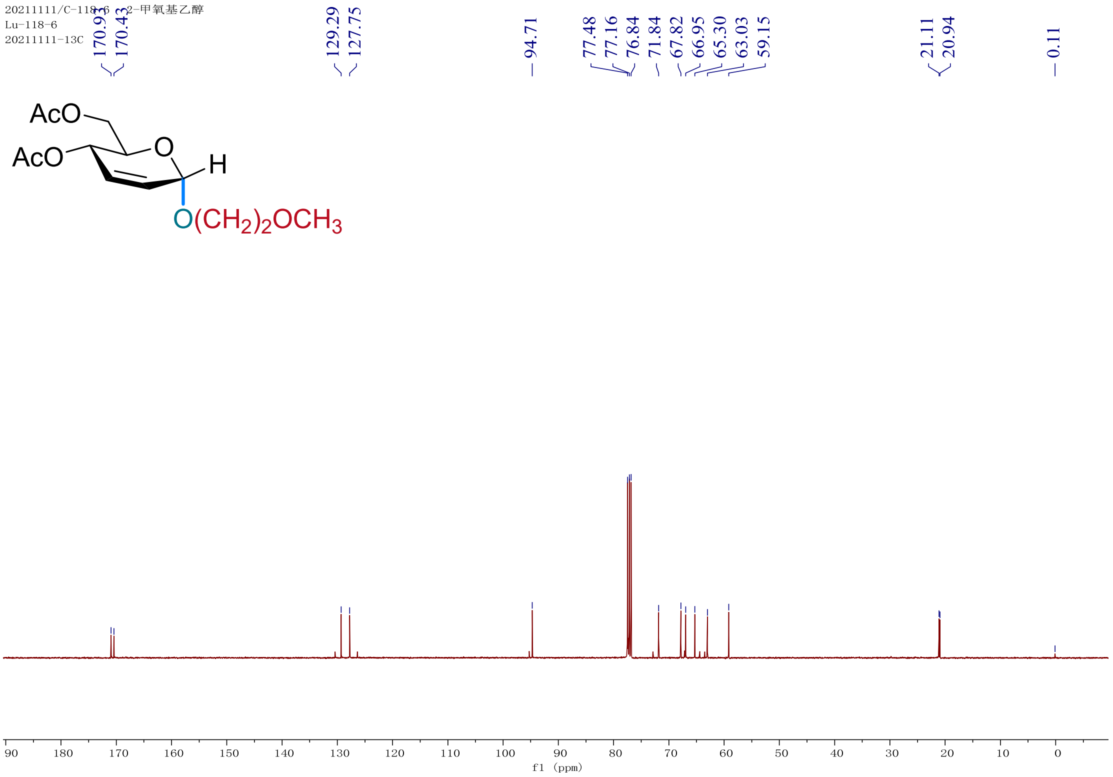

^{1}H NMR of 3j

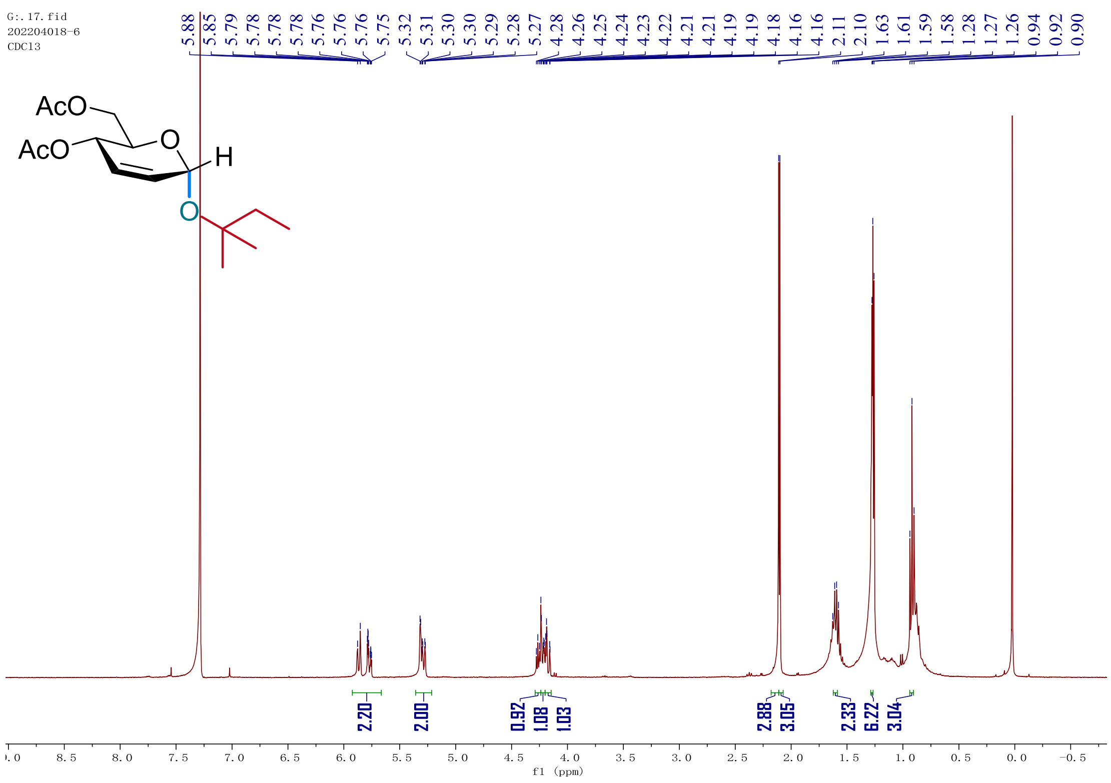

^{13}C NMR of 3j

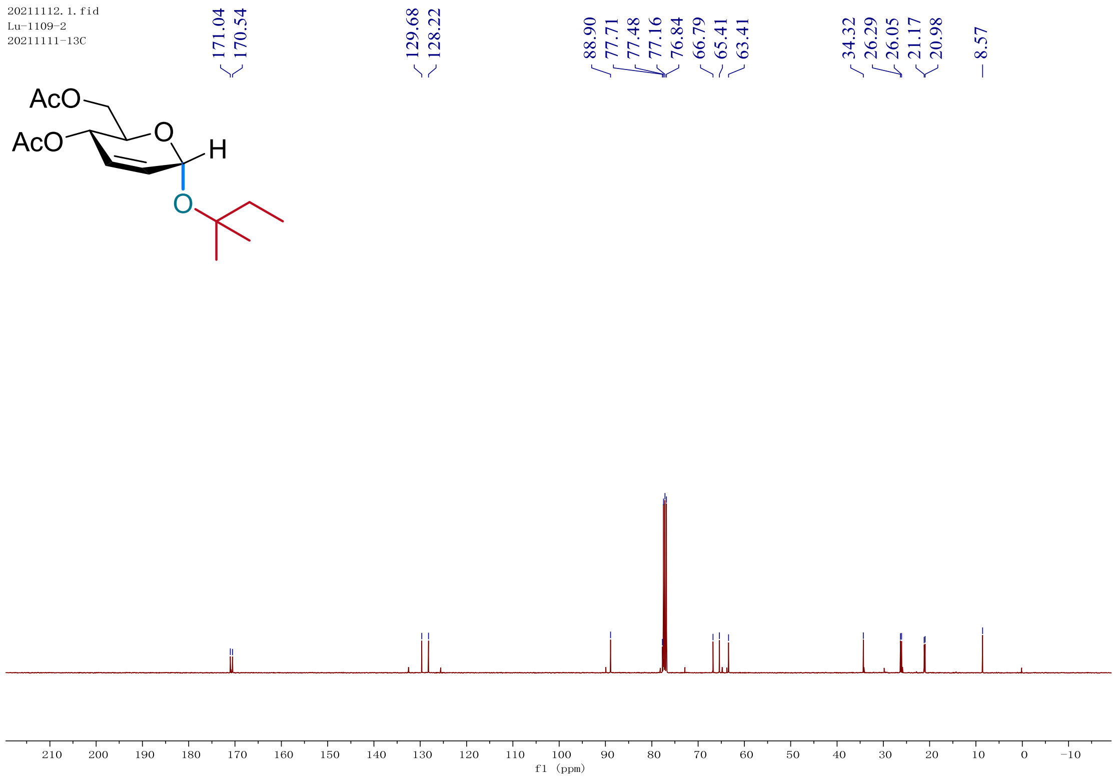

^{1}H NMR of 3k

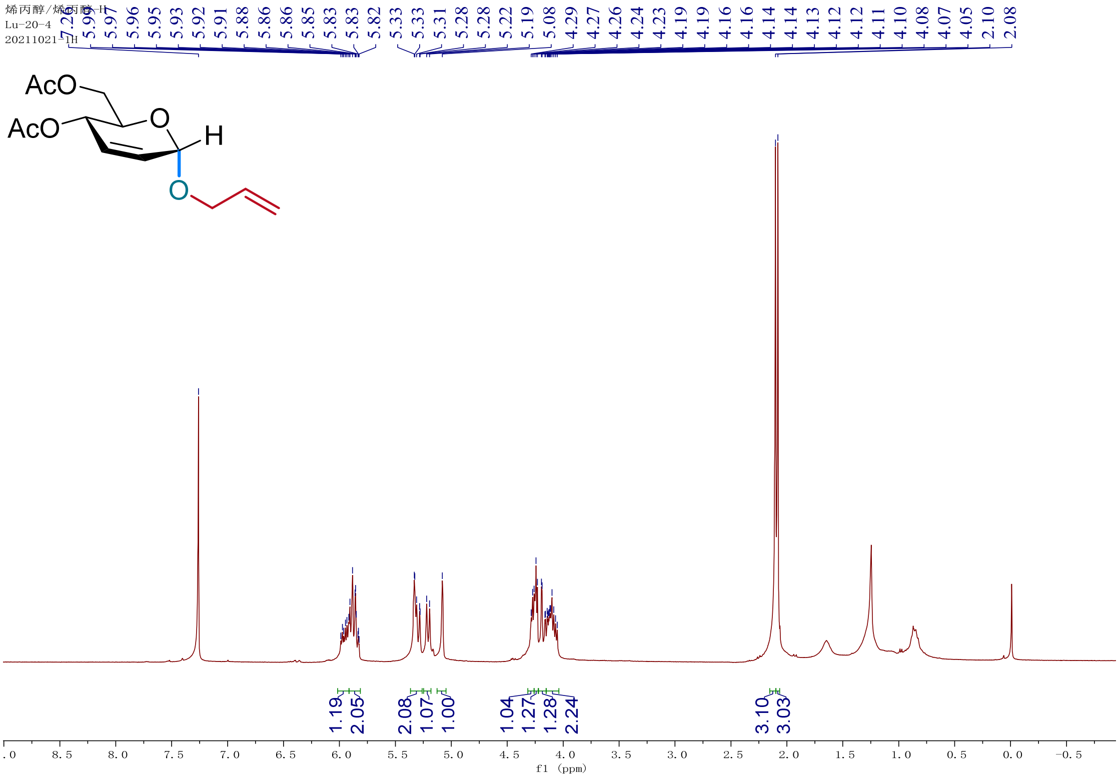

^{13}C NMR of 3k

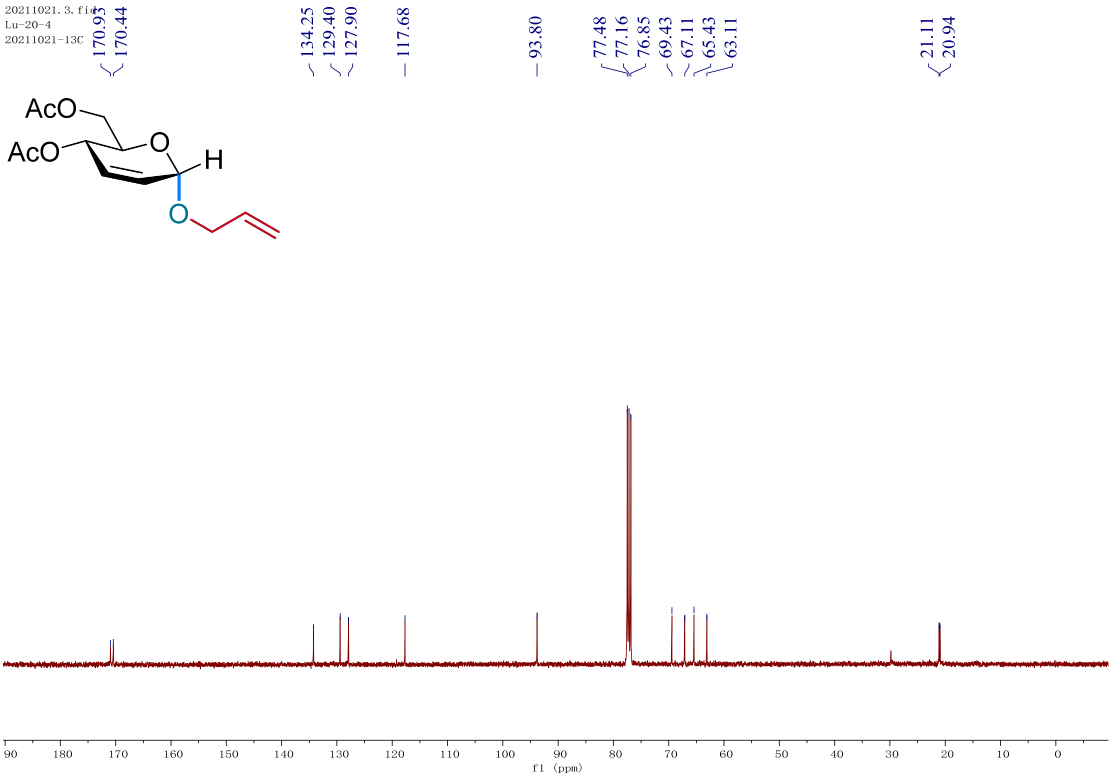

^{1}H NMR of 3l

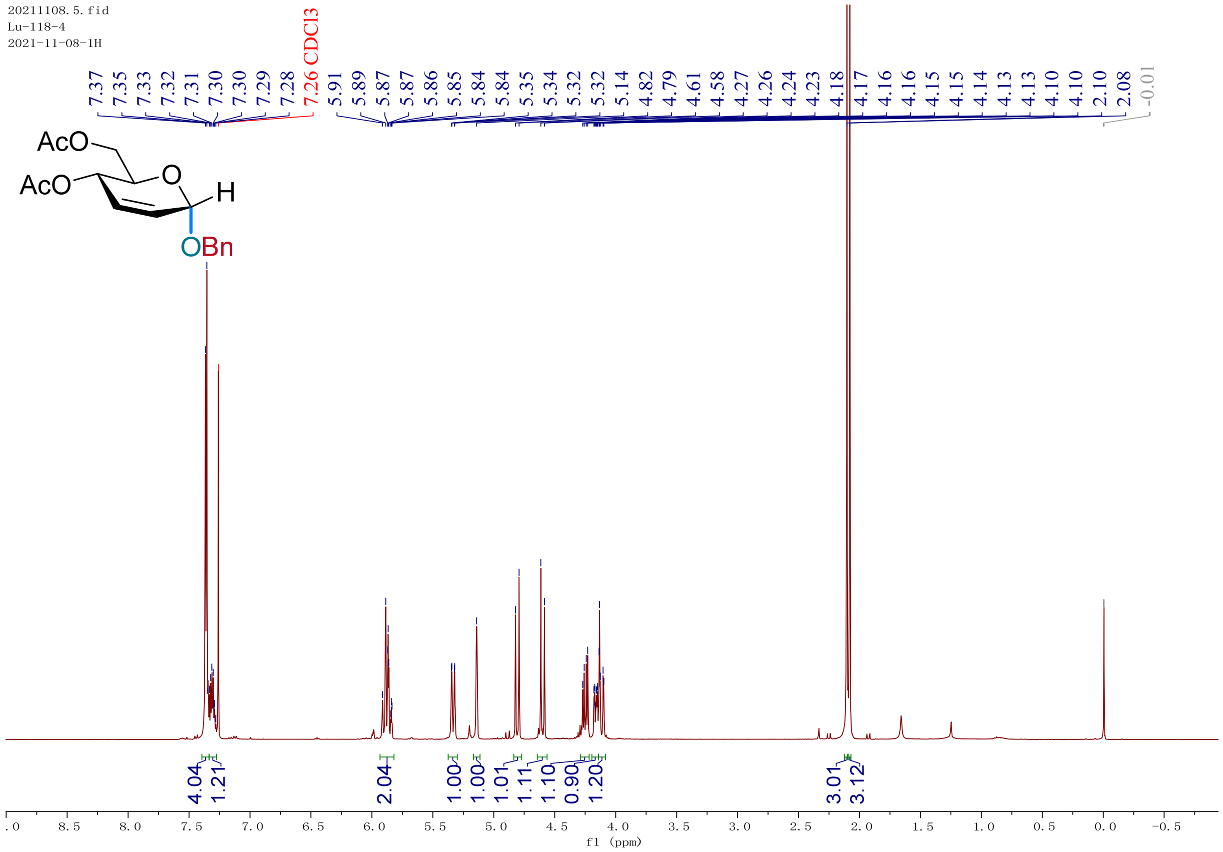

^{13}C NMR of 3l

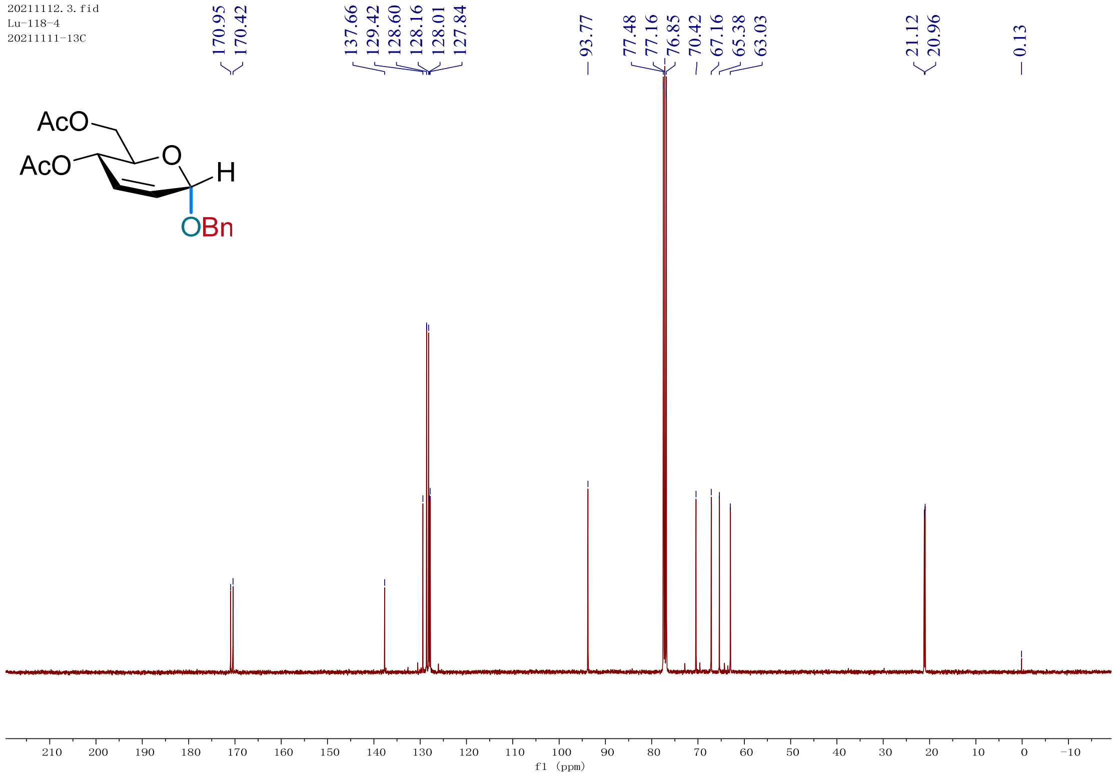

^{1}H NMR of 3m

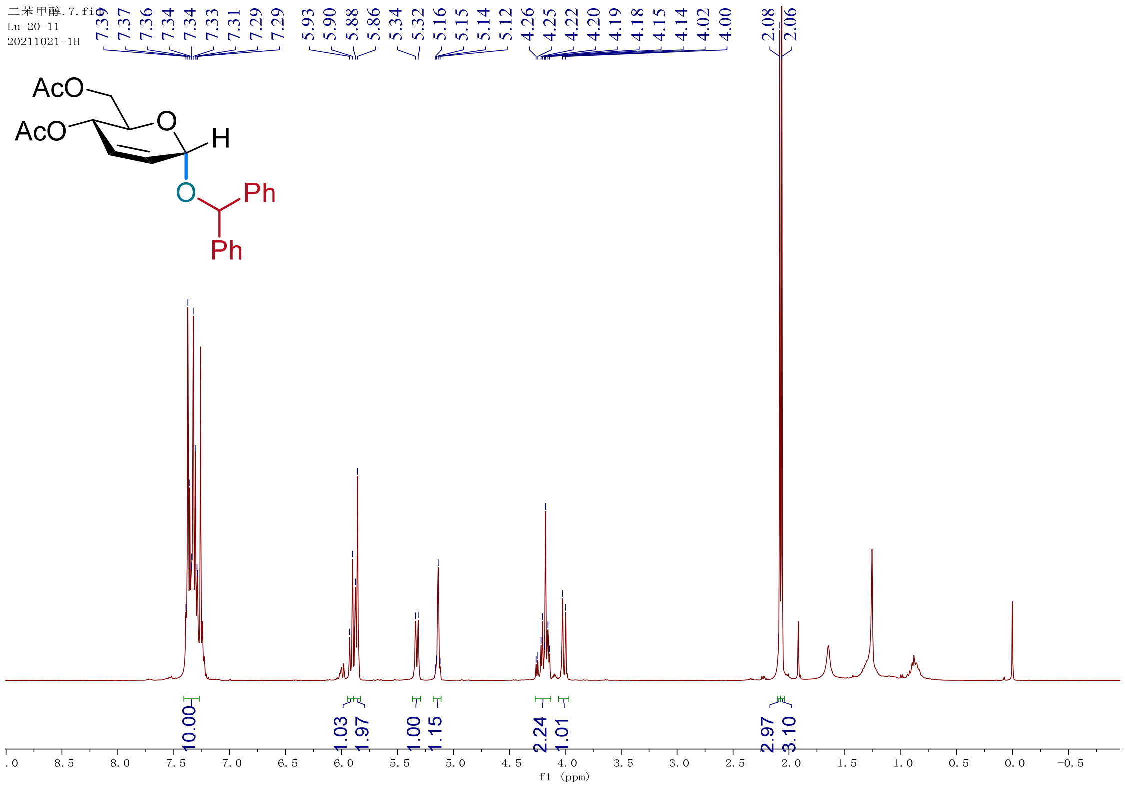

^{13}C NMR of 3m

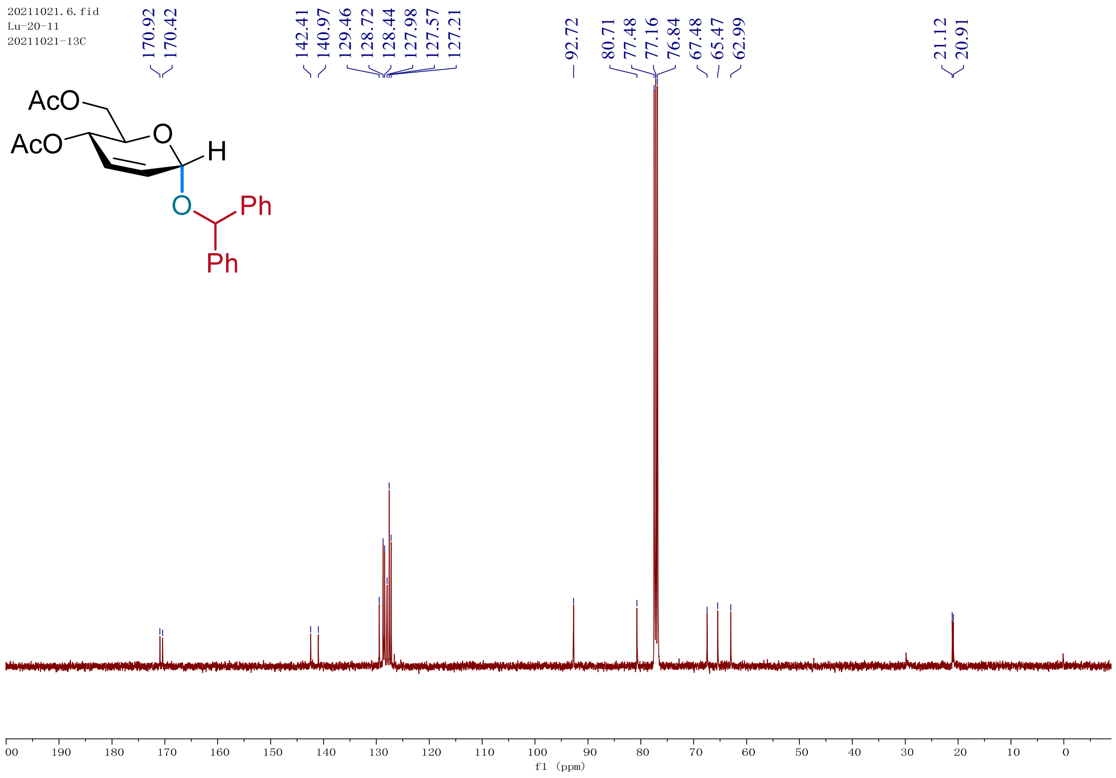

^{1}H NMR of 3n

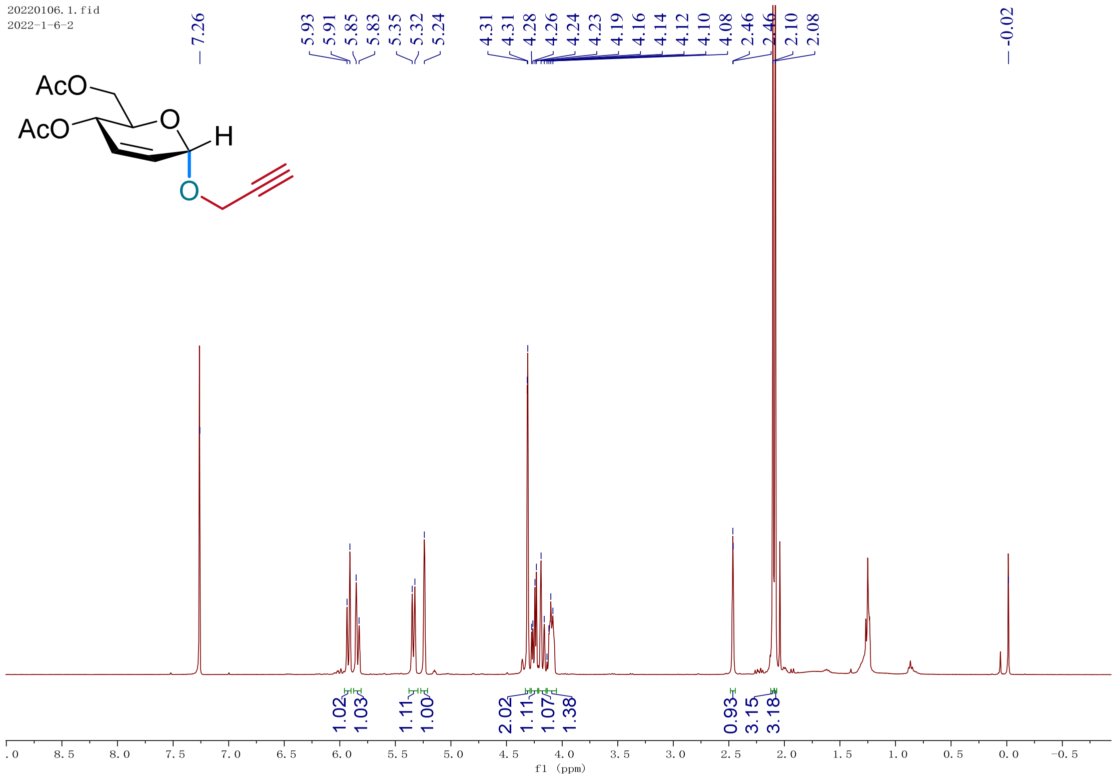

^{13}C NMR of 3n

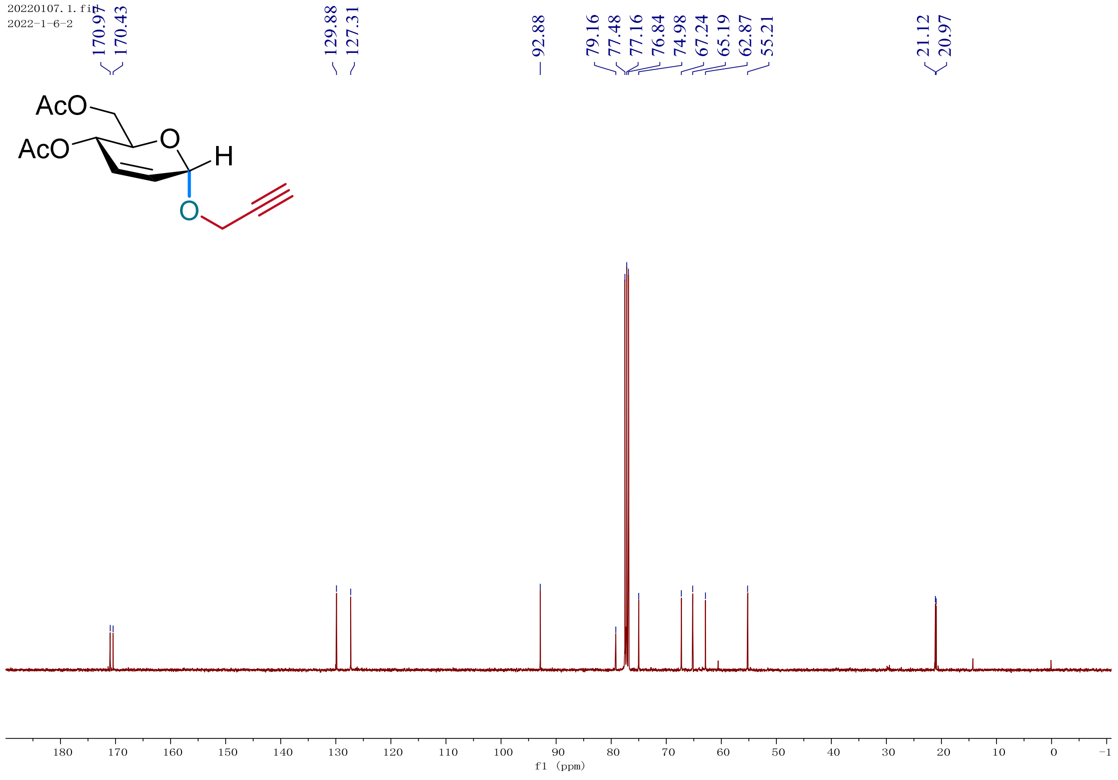

^{1}H NMR of 3o

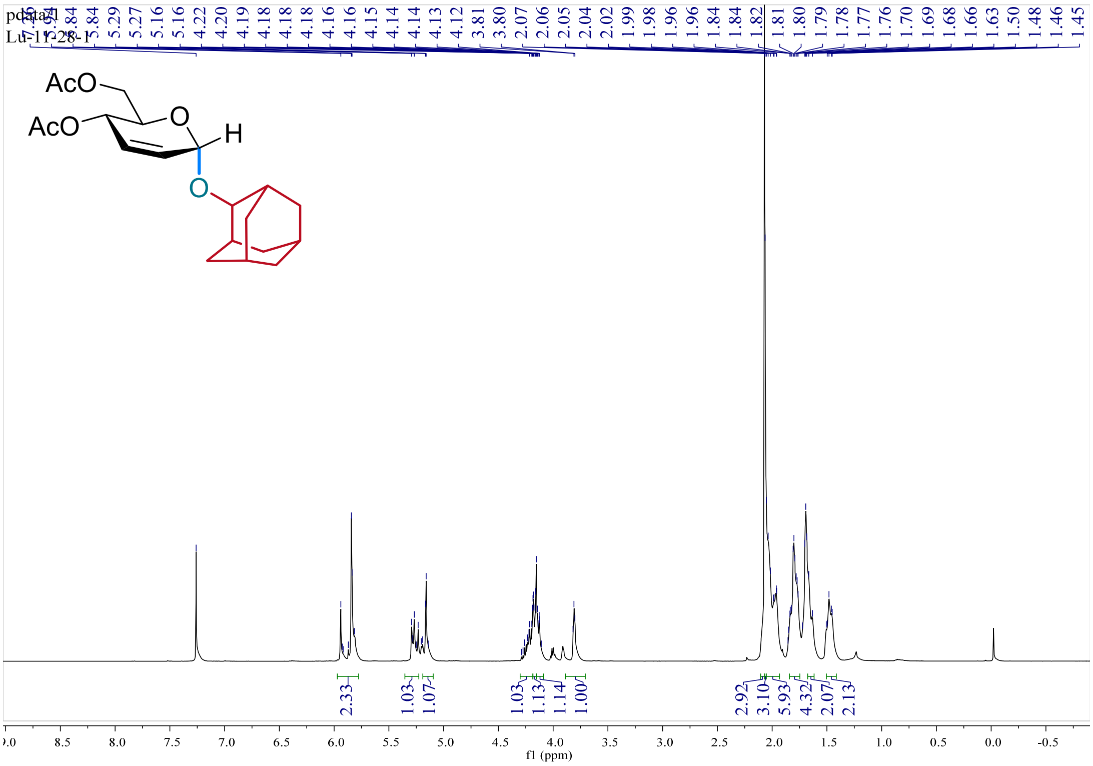

^{13}C NMR of 3o

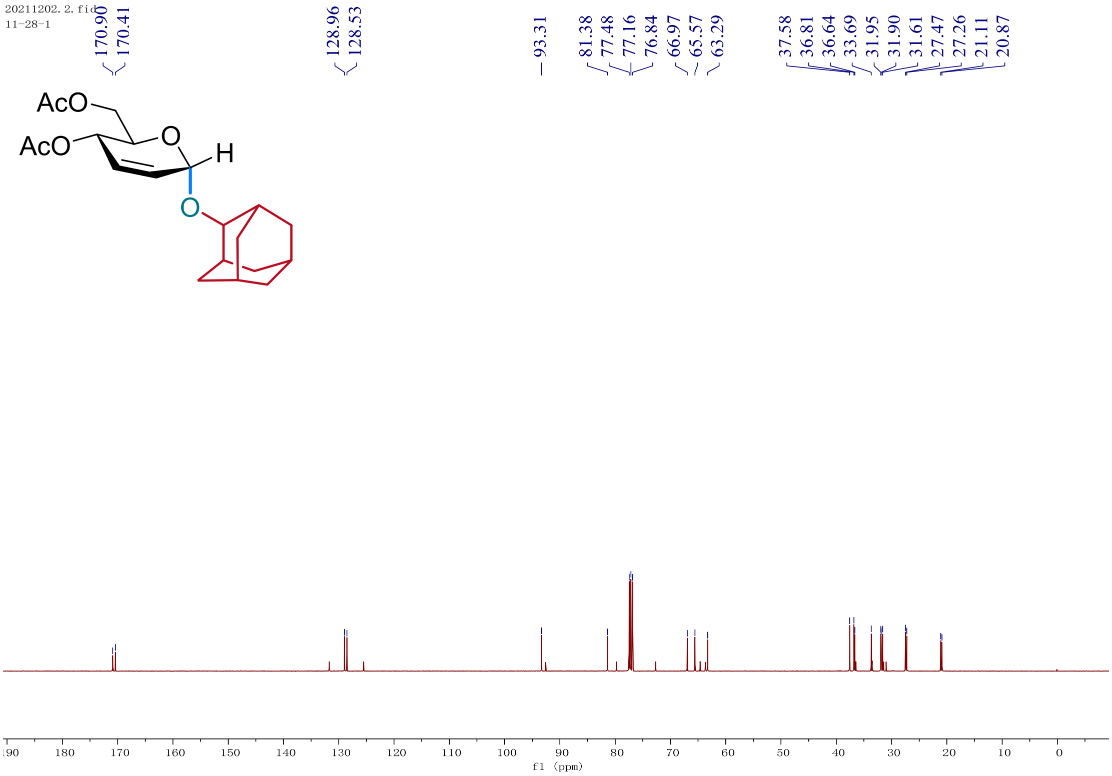

^{1}H NMR of 3p

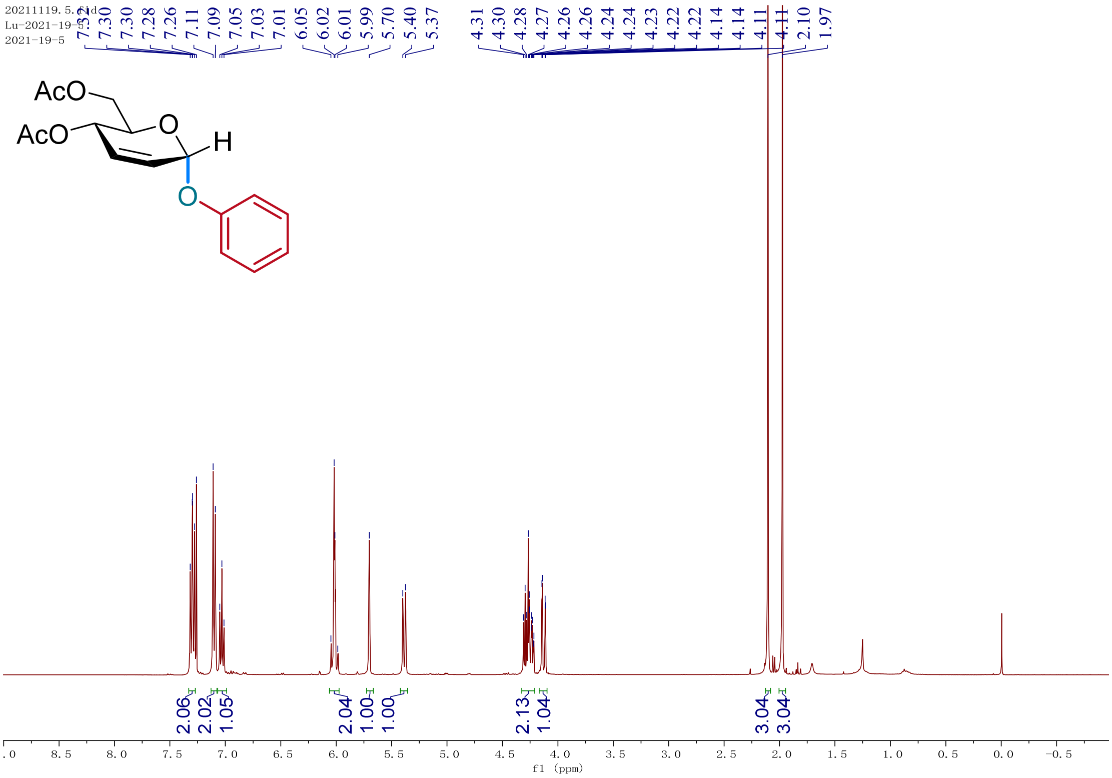

^{13}C NMR of 3p

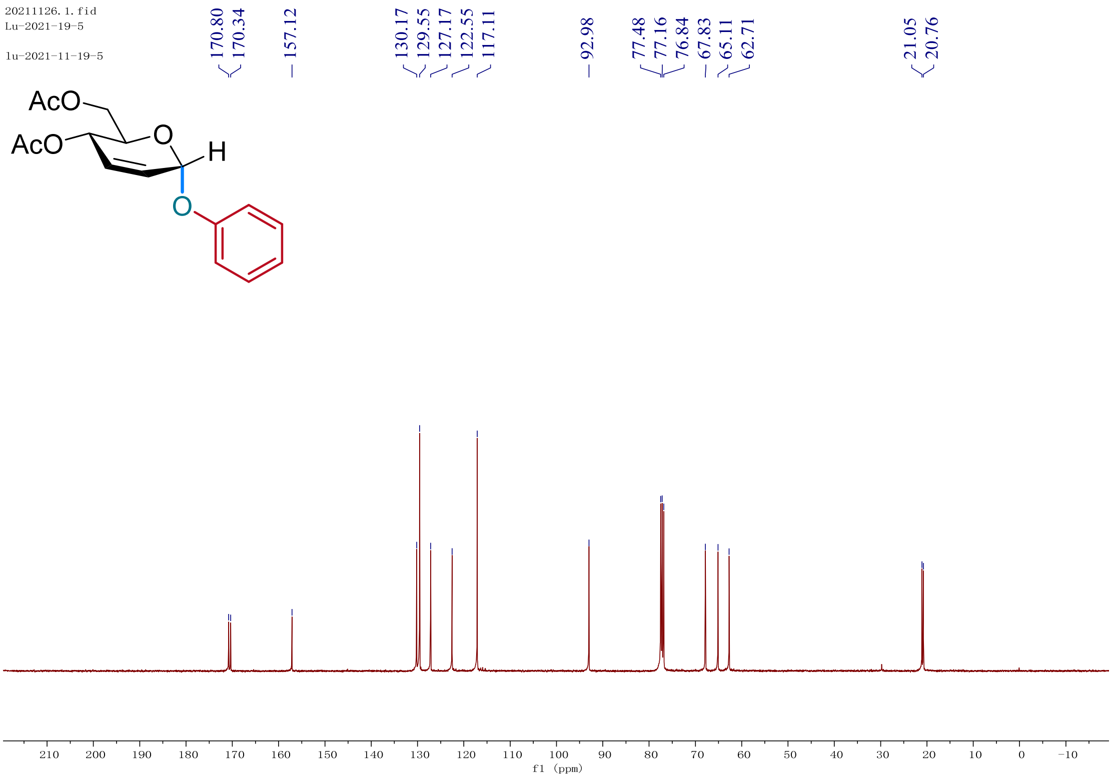

^{1}H NMR of 3q

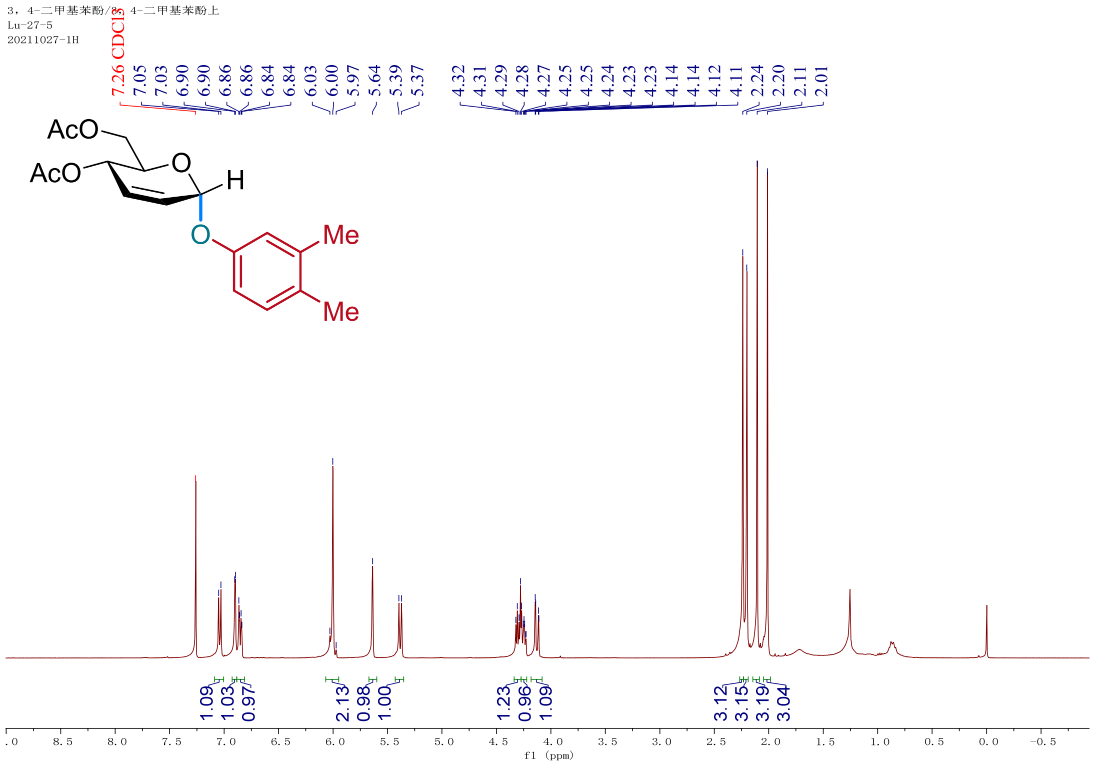

^{13}C NMR of 3q

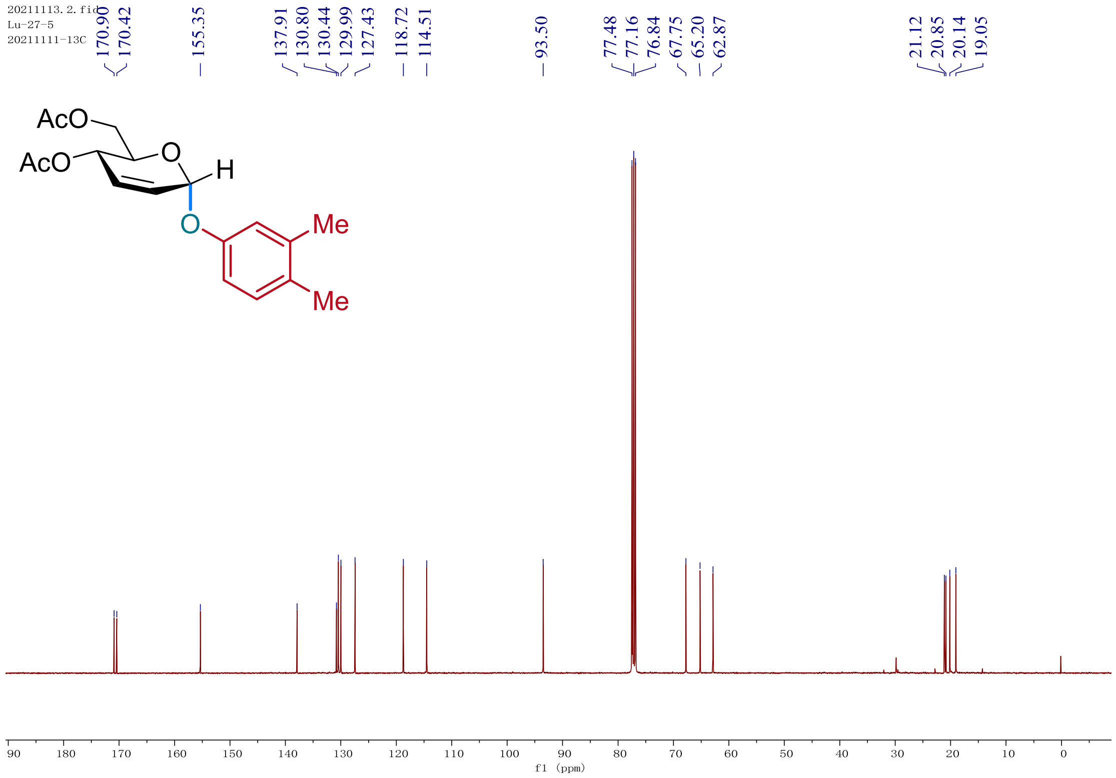

^{1}H NMR of 3r

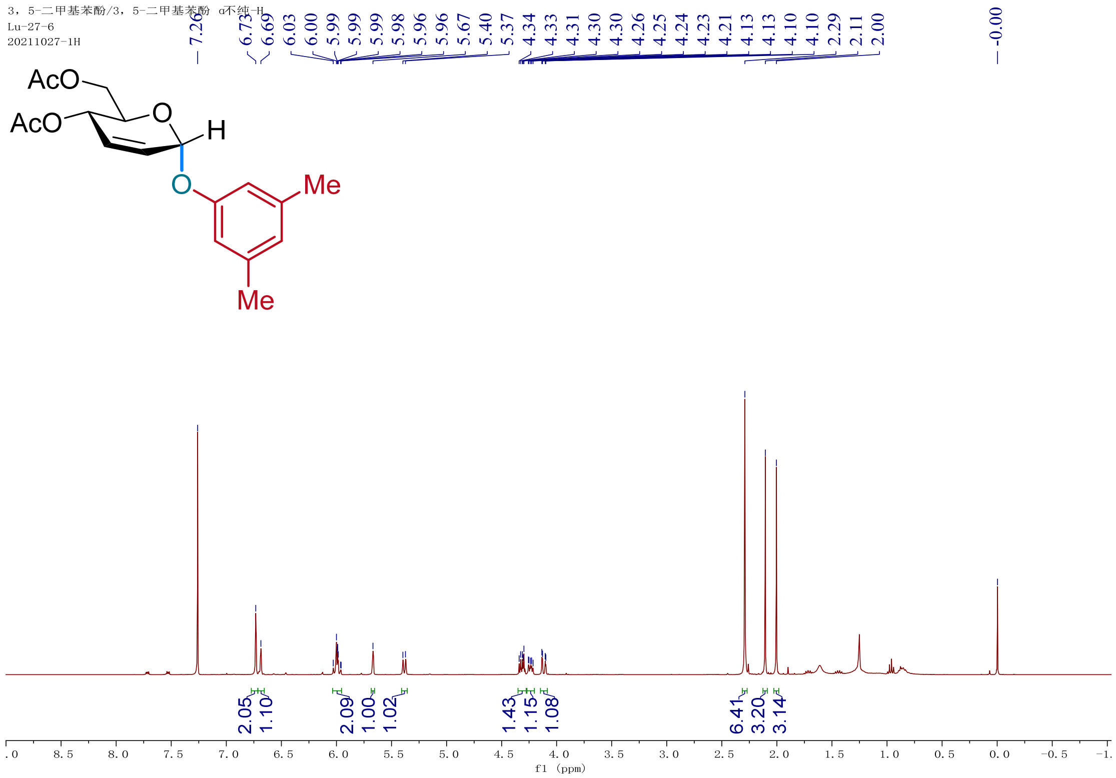

^{13}C NMR of 3r

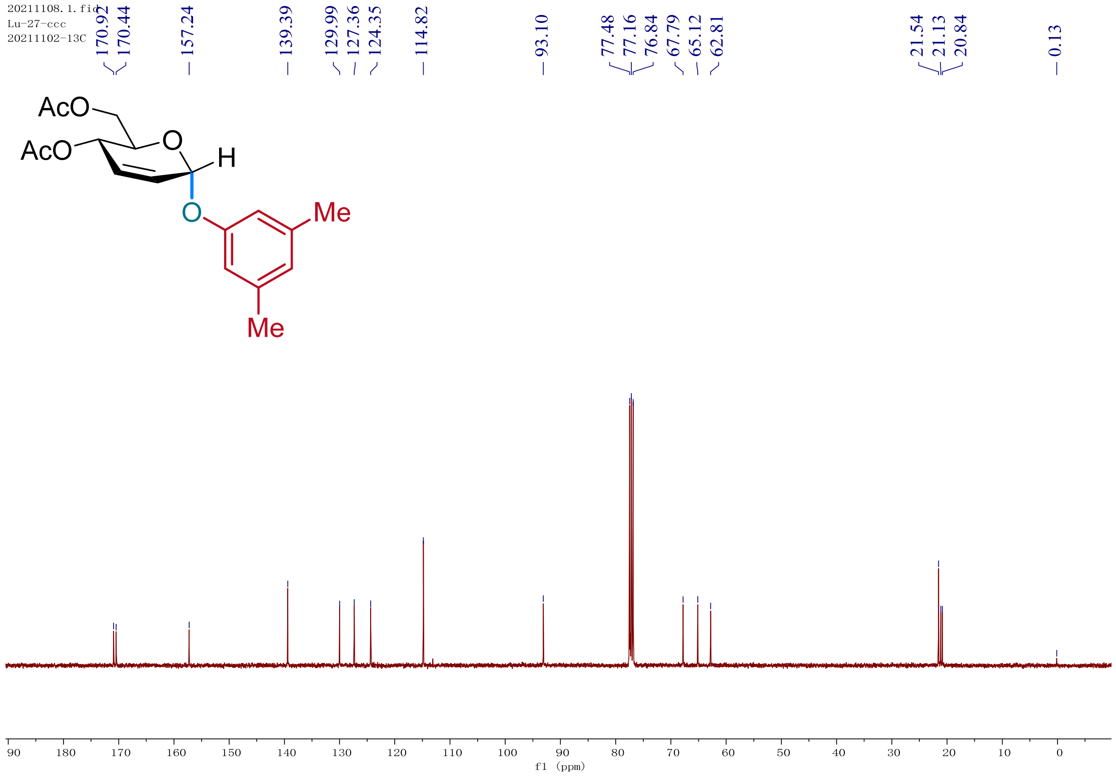

^{1}H NMR of 3s

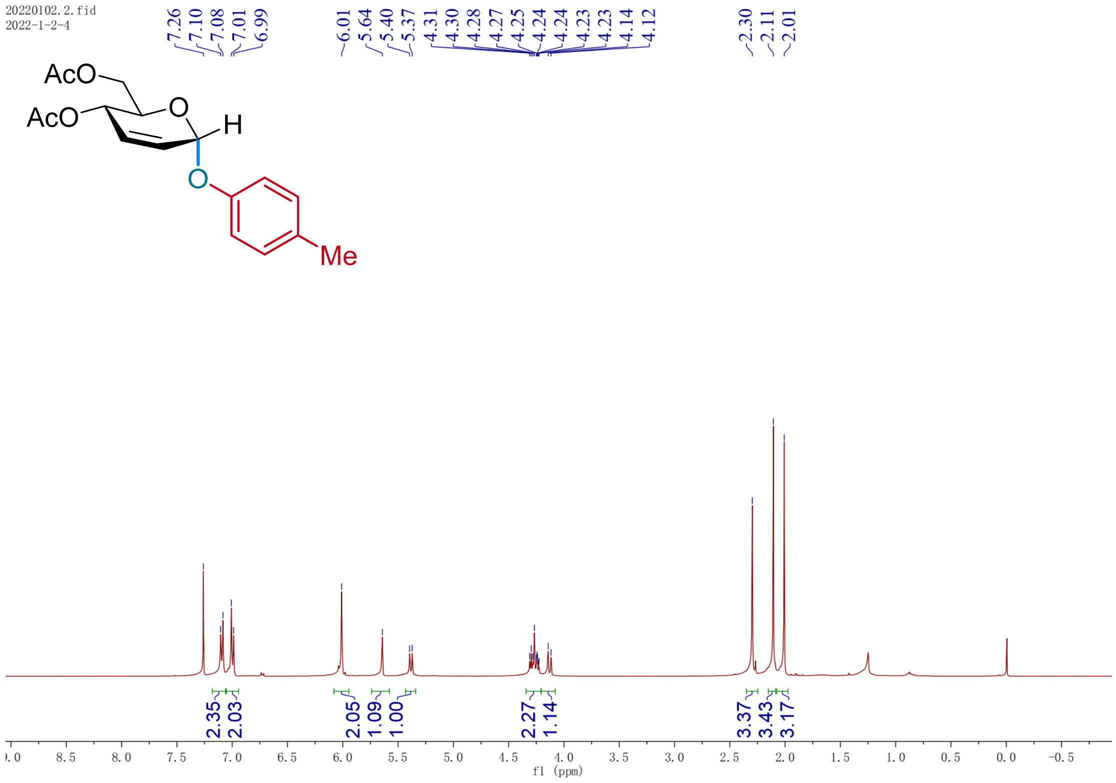

^{13}C NMR of 3s

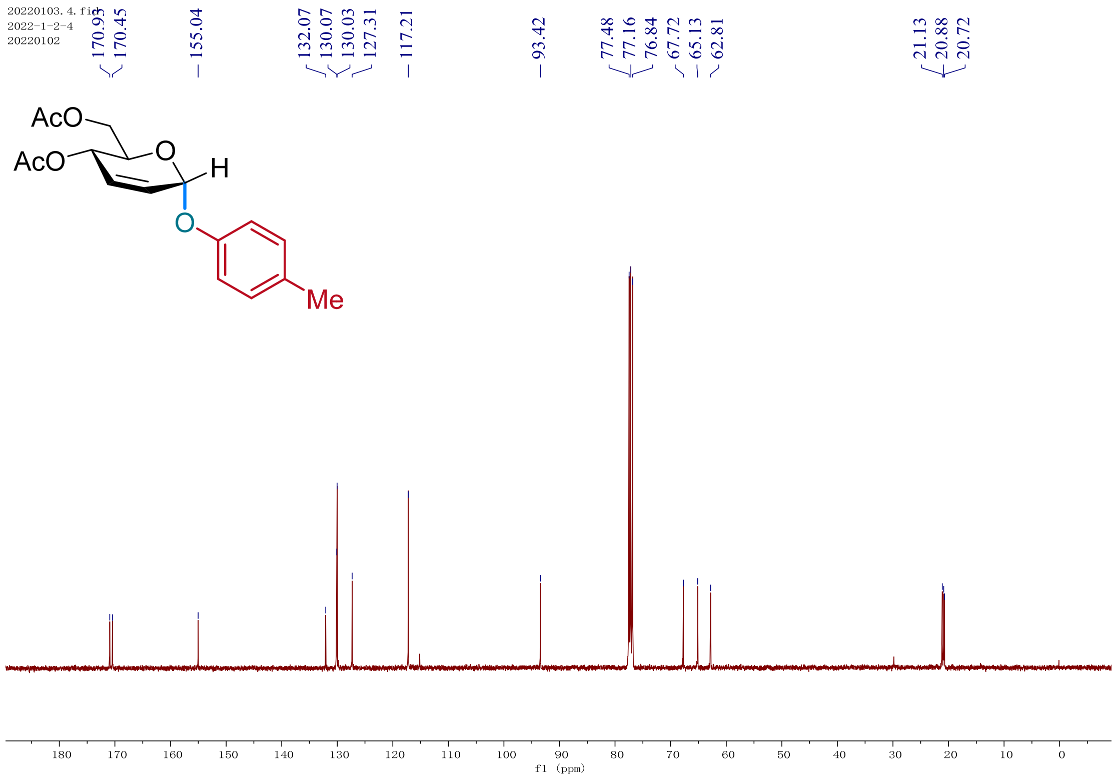

^{1}H NMR of 3t

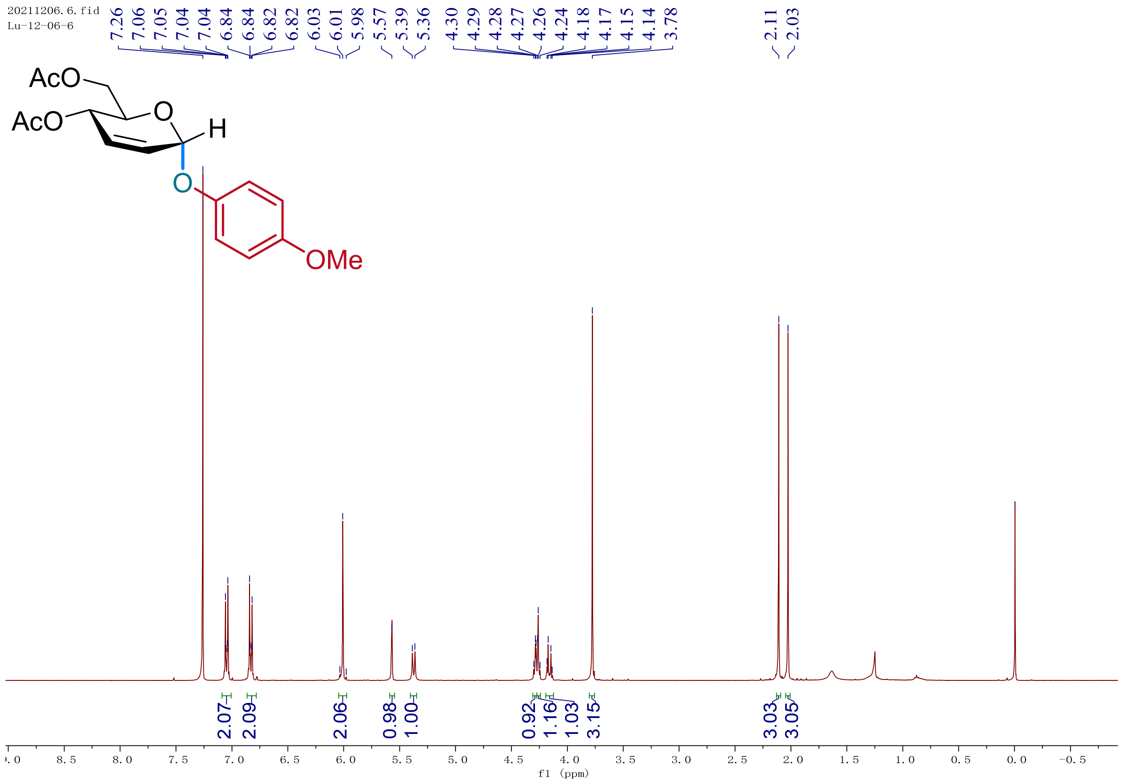

^{13}C NMR of 3t

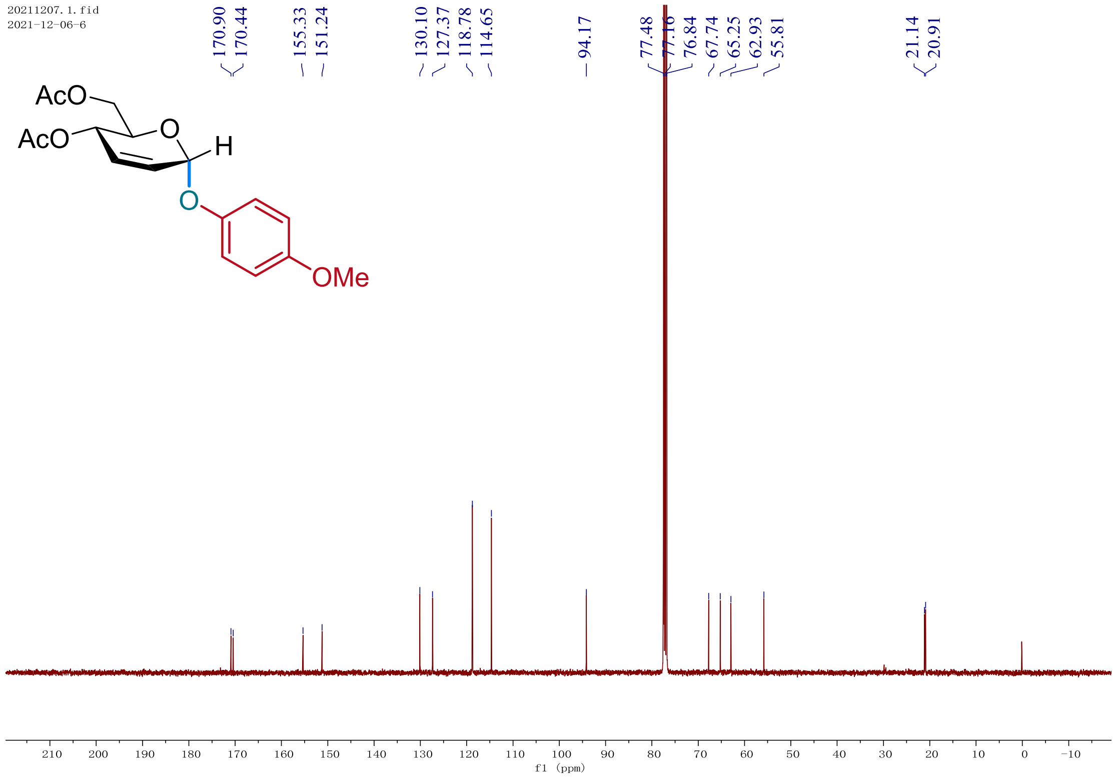

^{1}H NMR of 3u

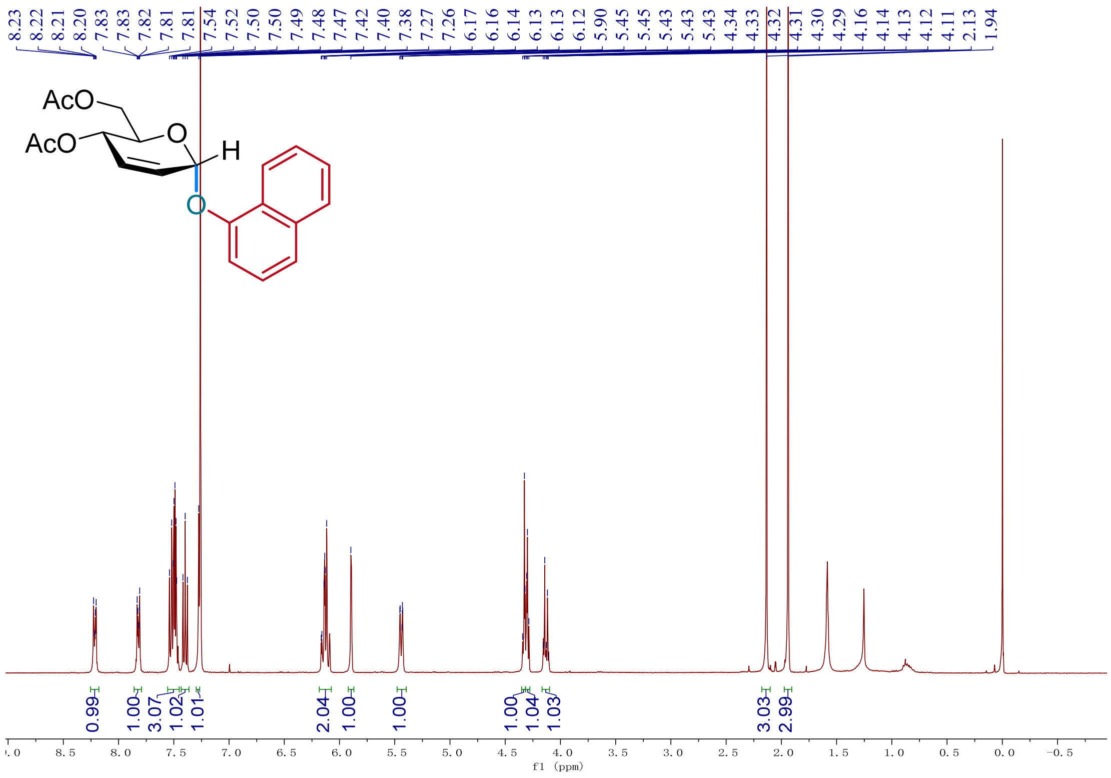

^{13}C NMR of 3u

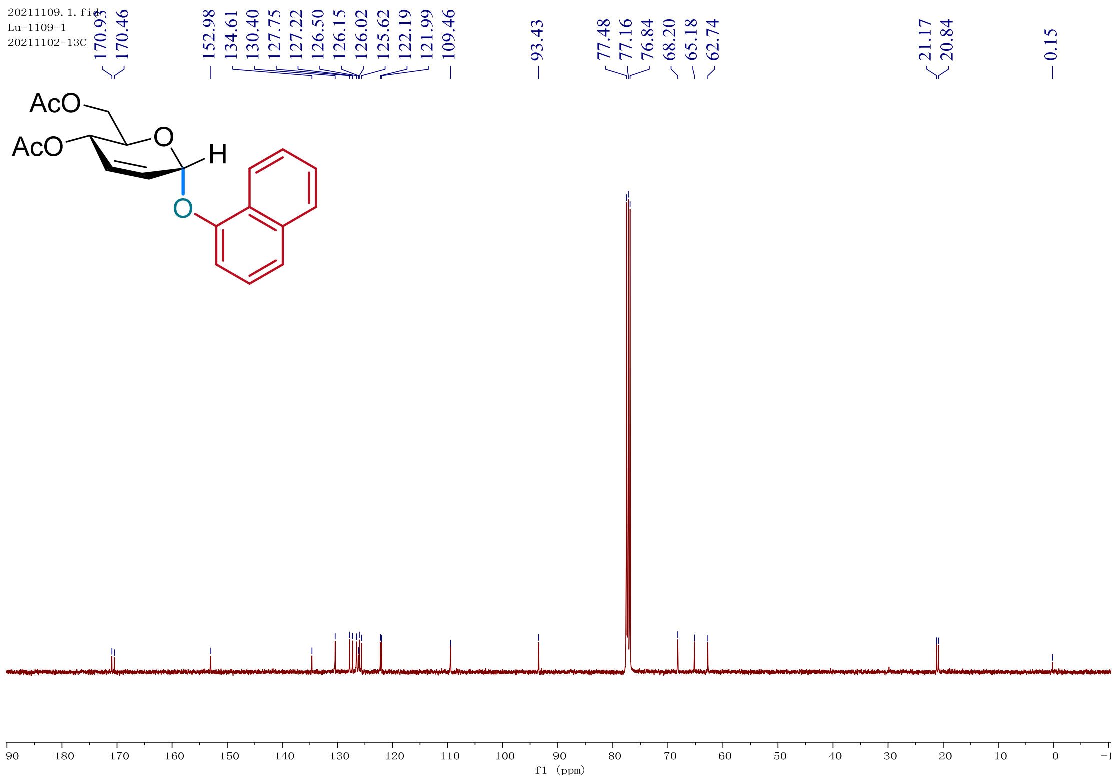

^{1}H NMR of 3v

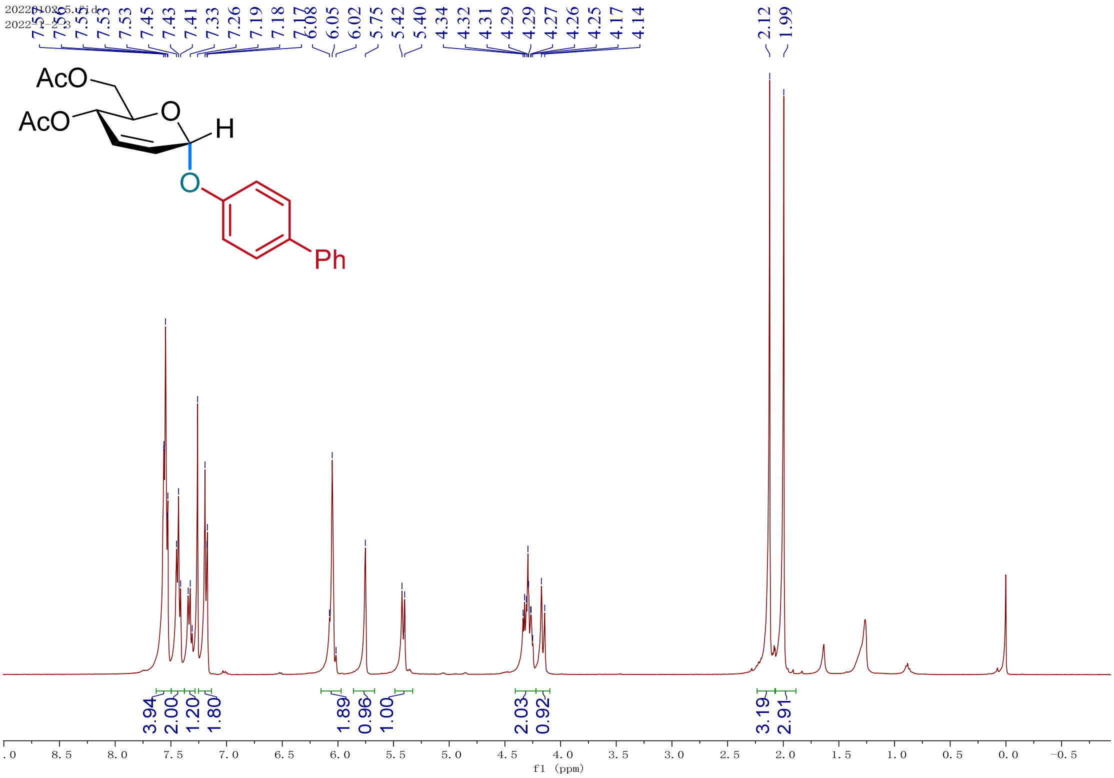

^{13}C NMR of 3v

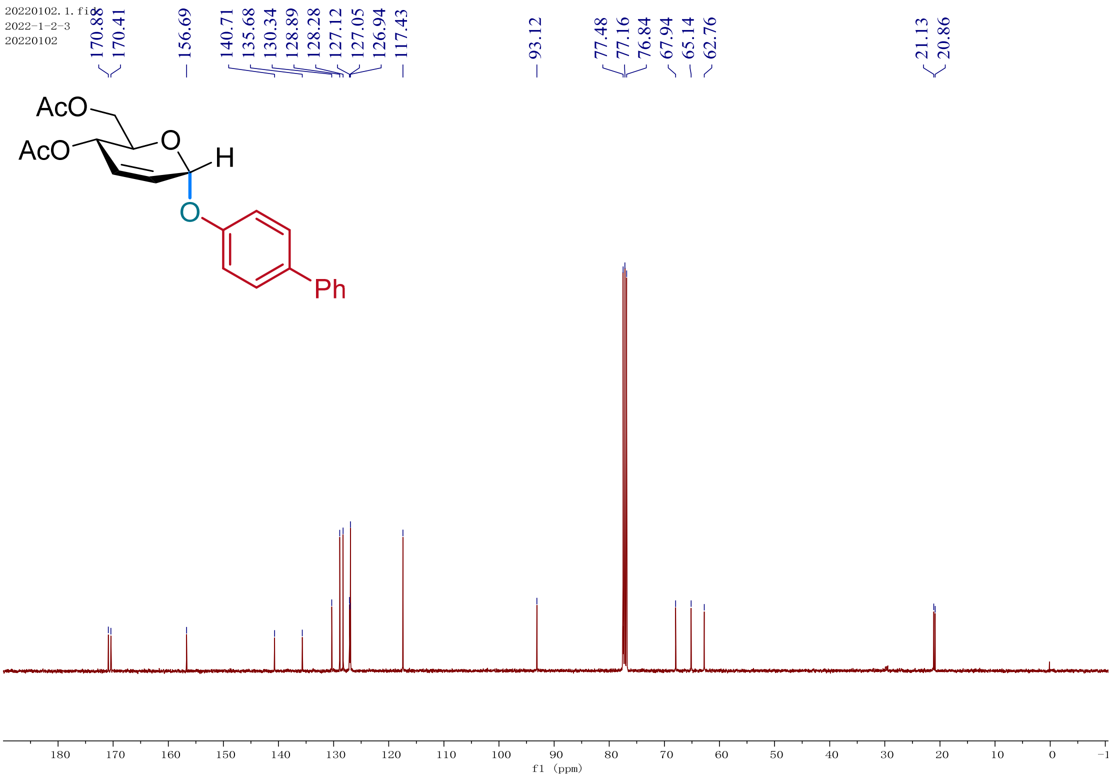

^{1}H NMR of 3w

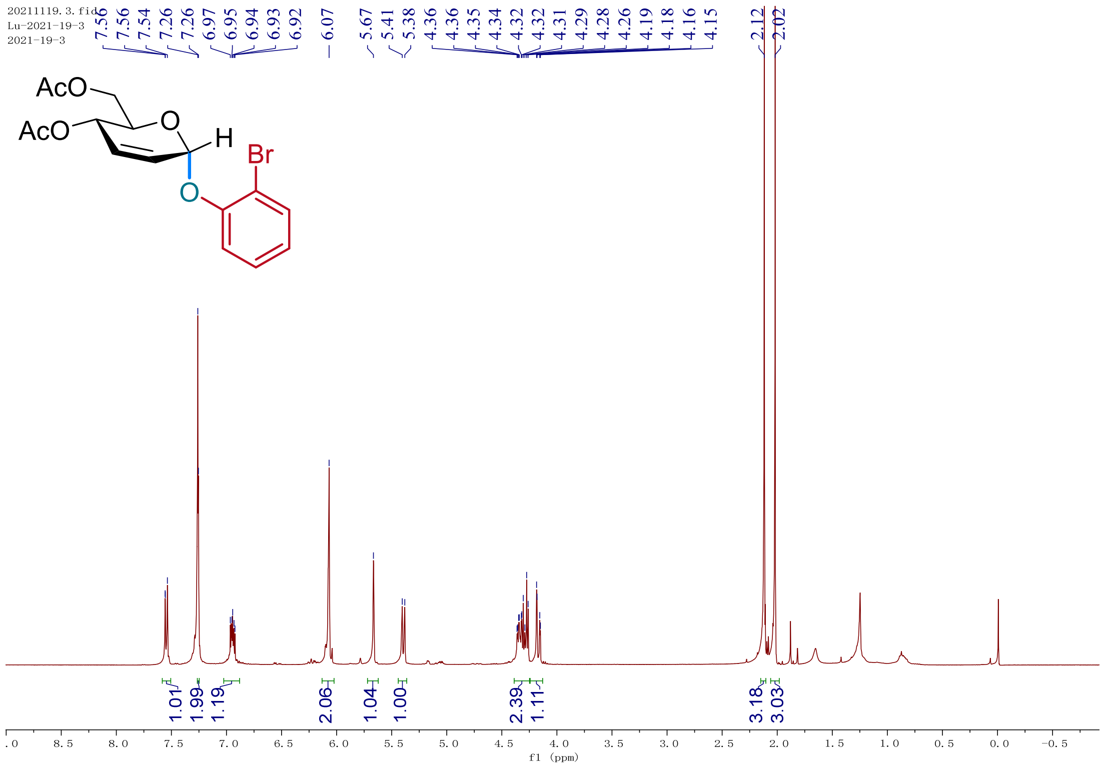

^{13}C NMR of 3w

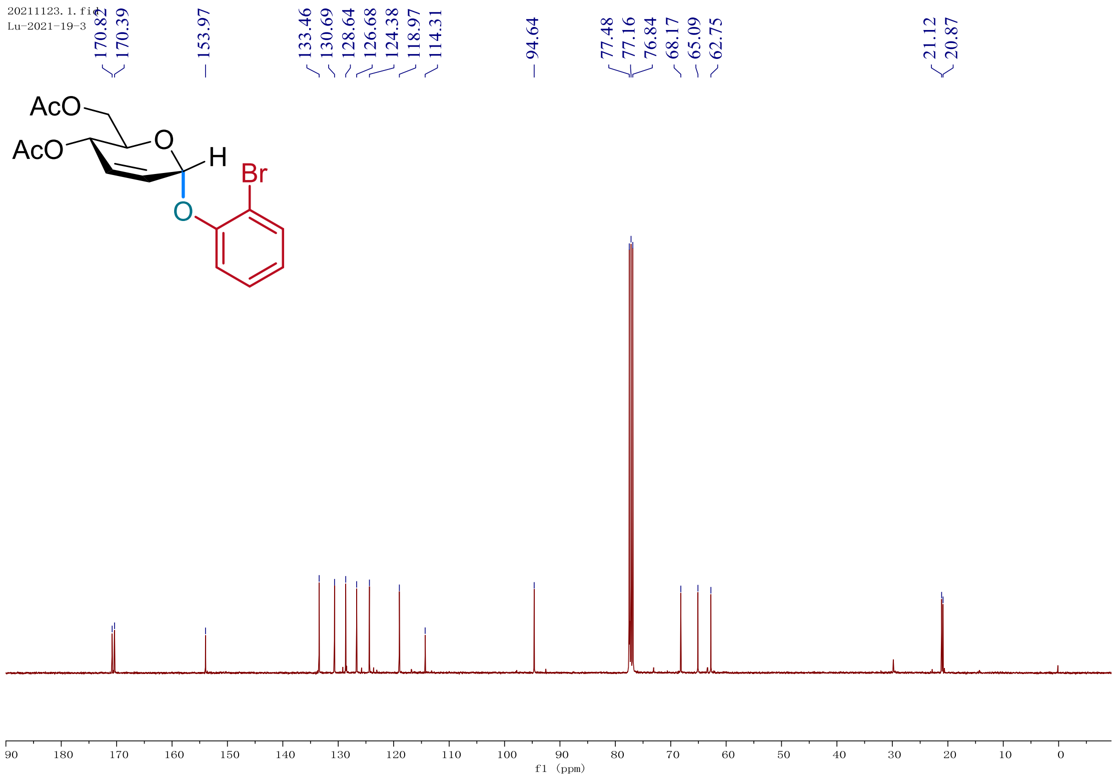

^{1}H NMR of 3x

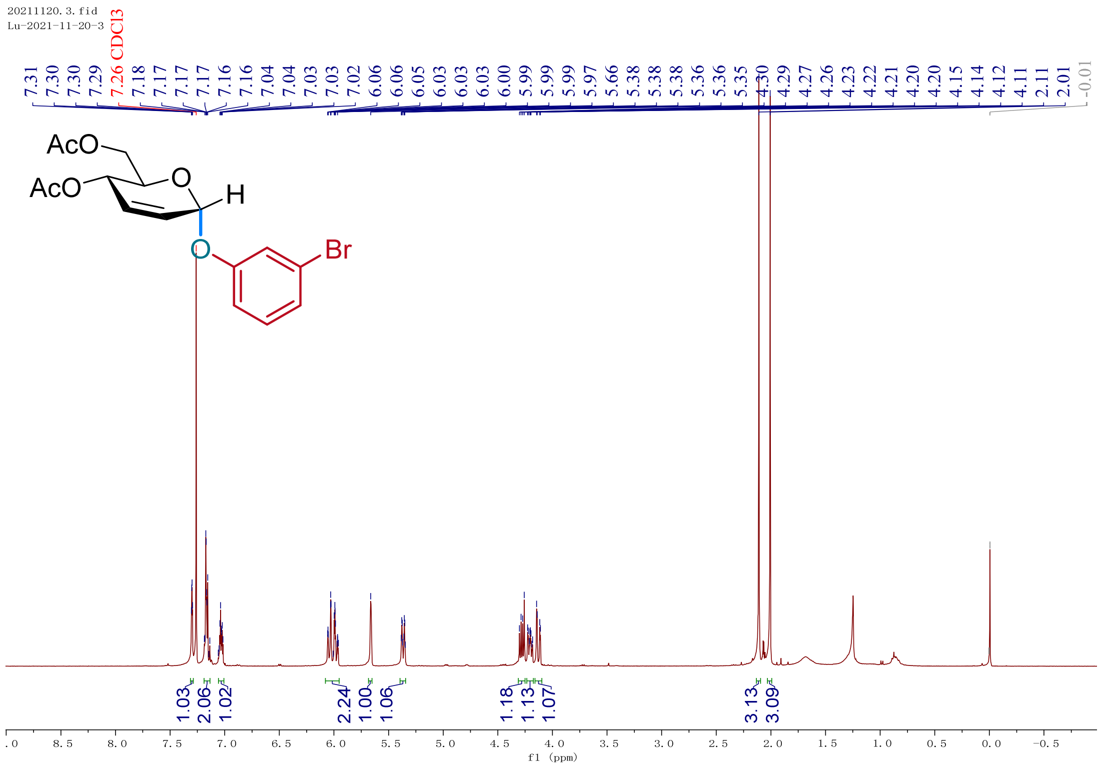

^{13}C NMR of 3x

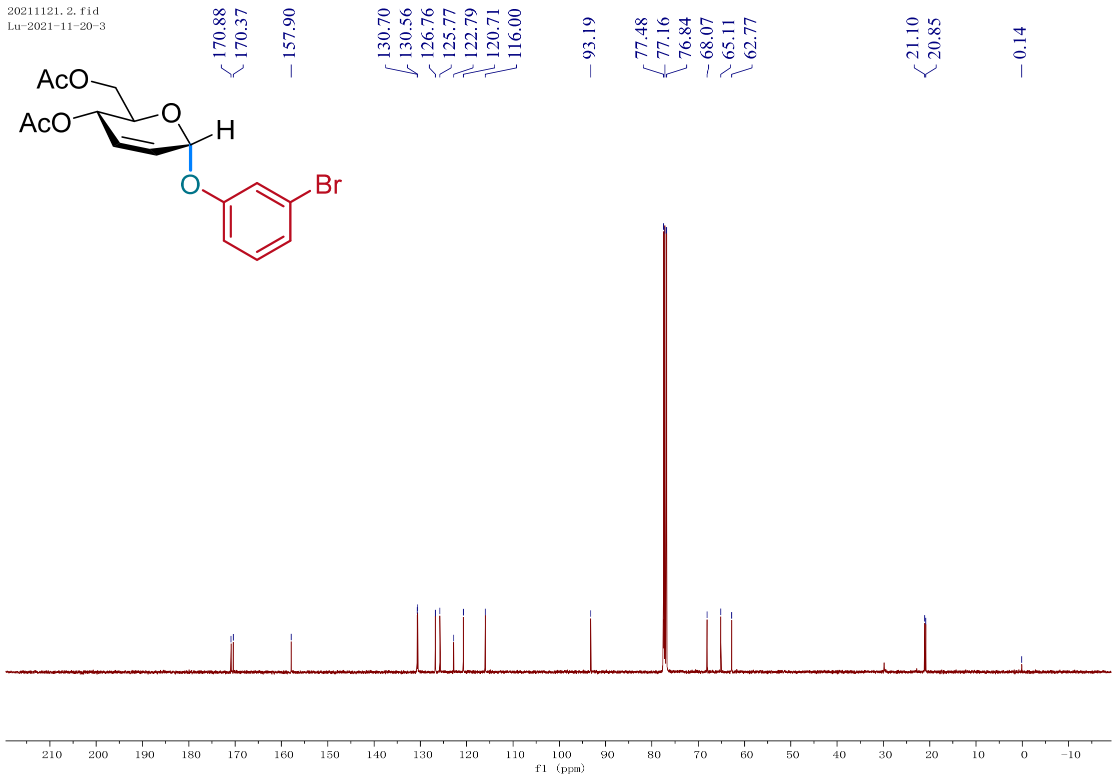

^{1}H NMR of 3y

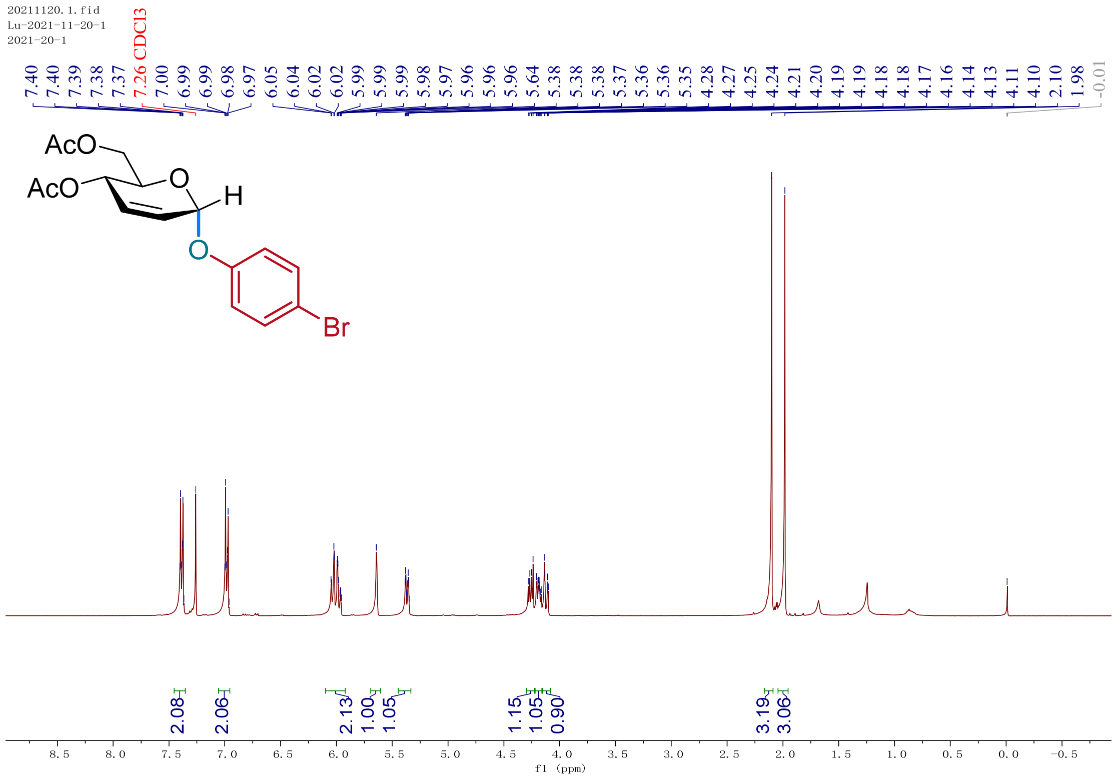

^{13}C NMR of 3y

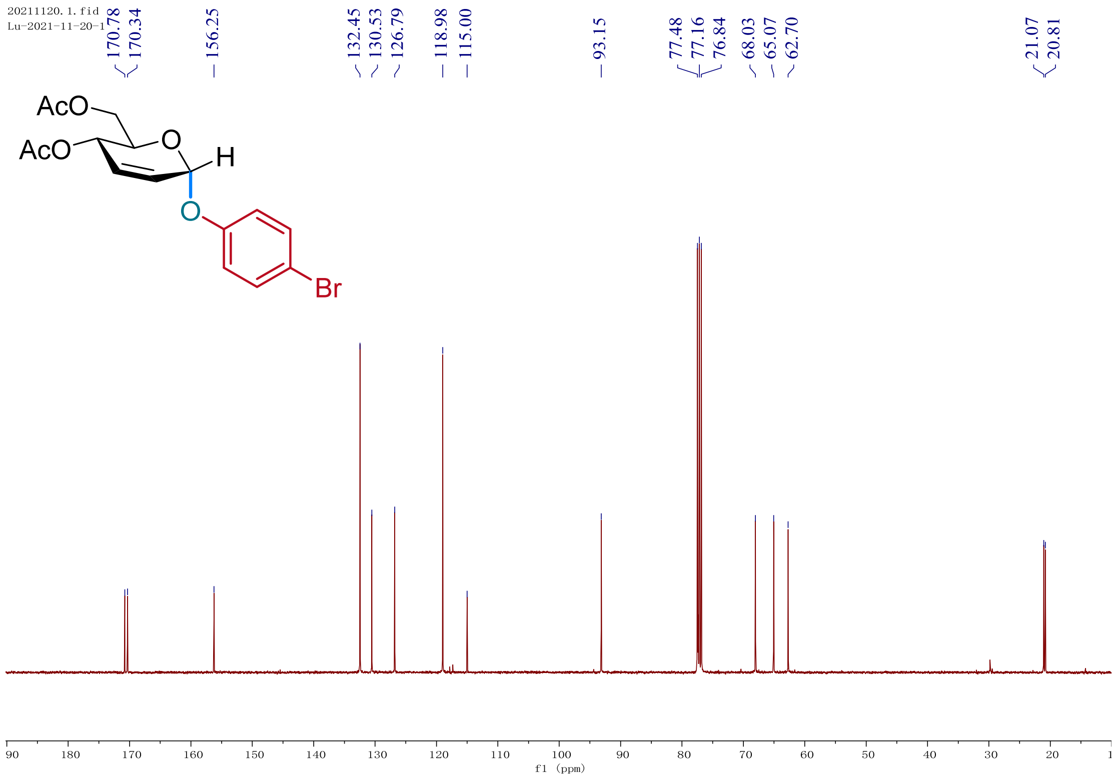

^{1}H NMR of 3z

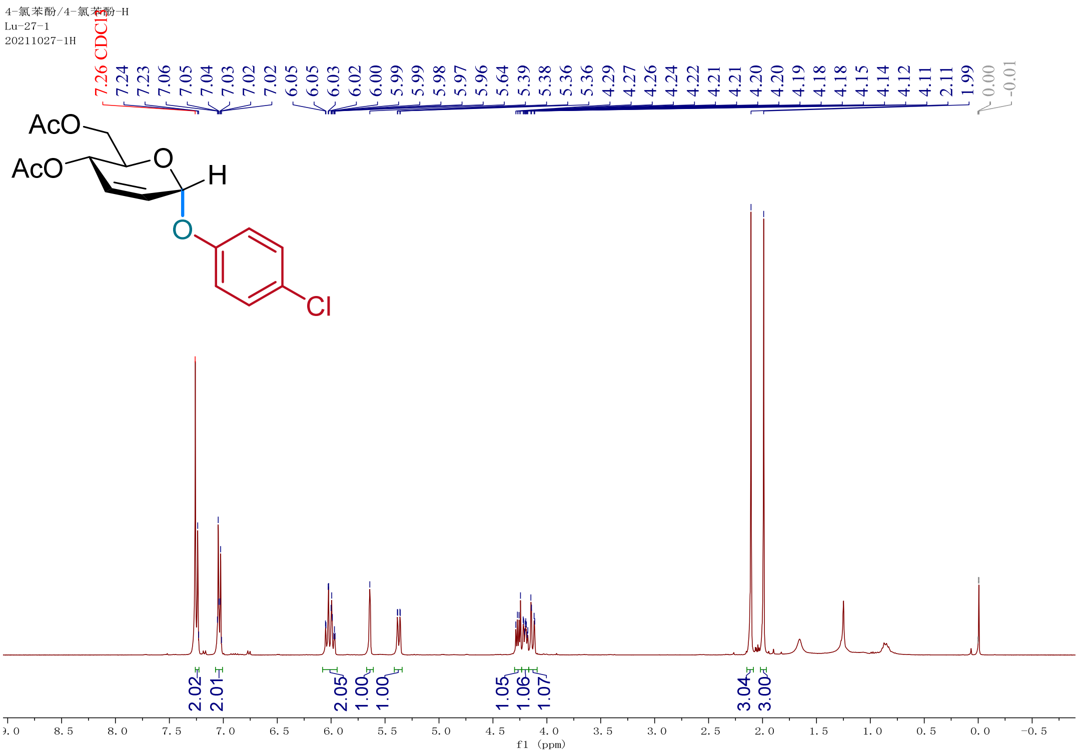

^{13}C NMR of 3z

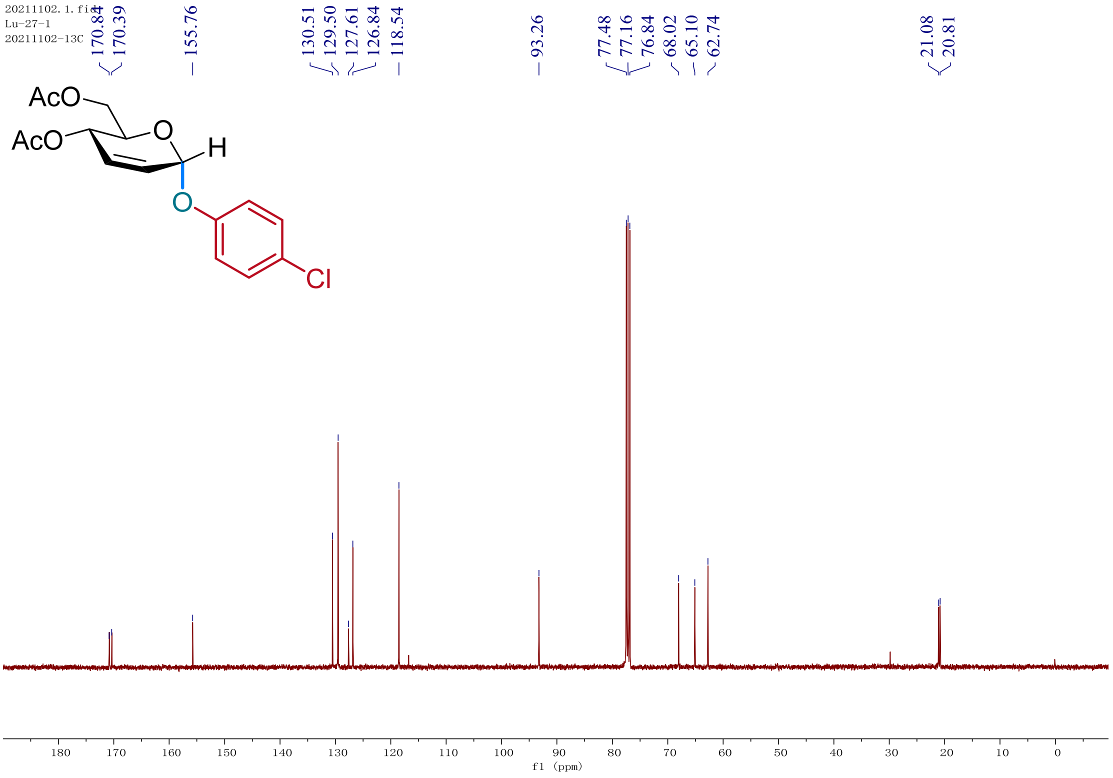

^{1}H NMR of 3aa

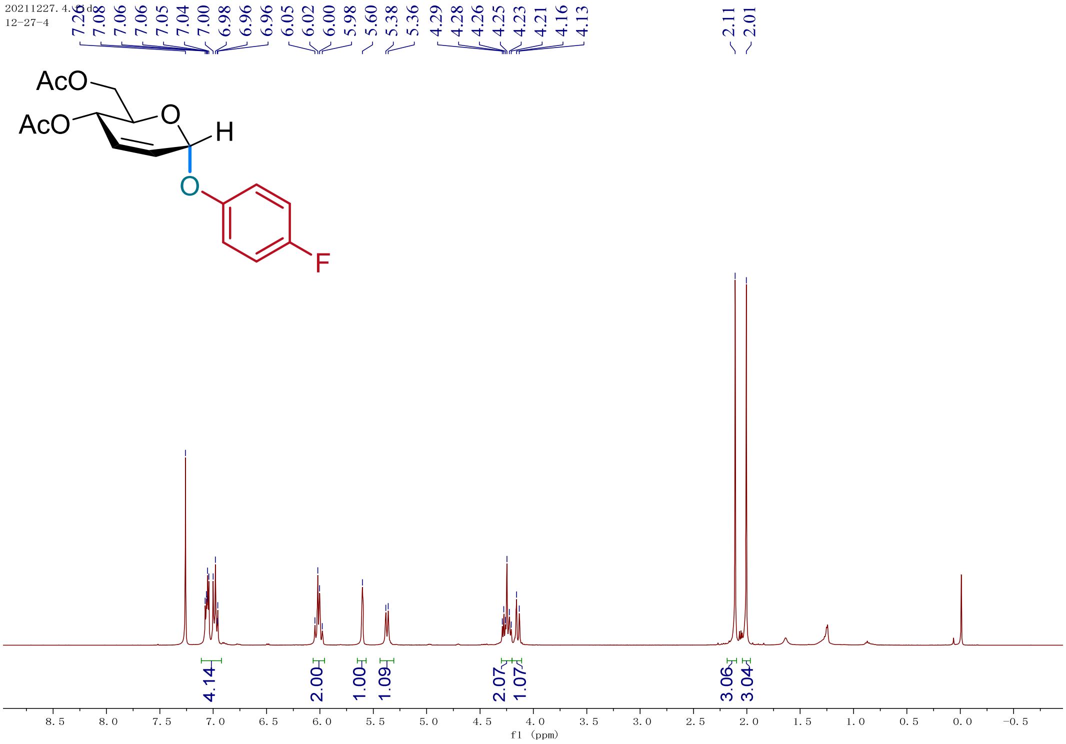

^{13}C NMR of 3aa

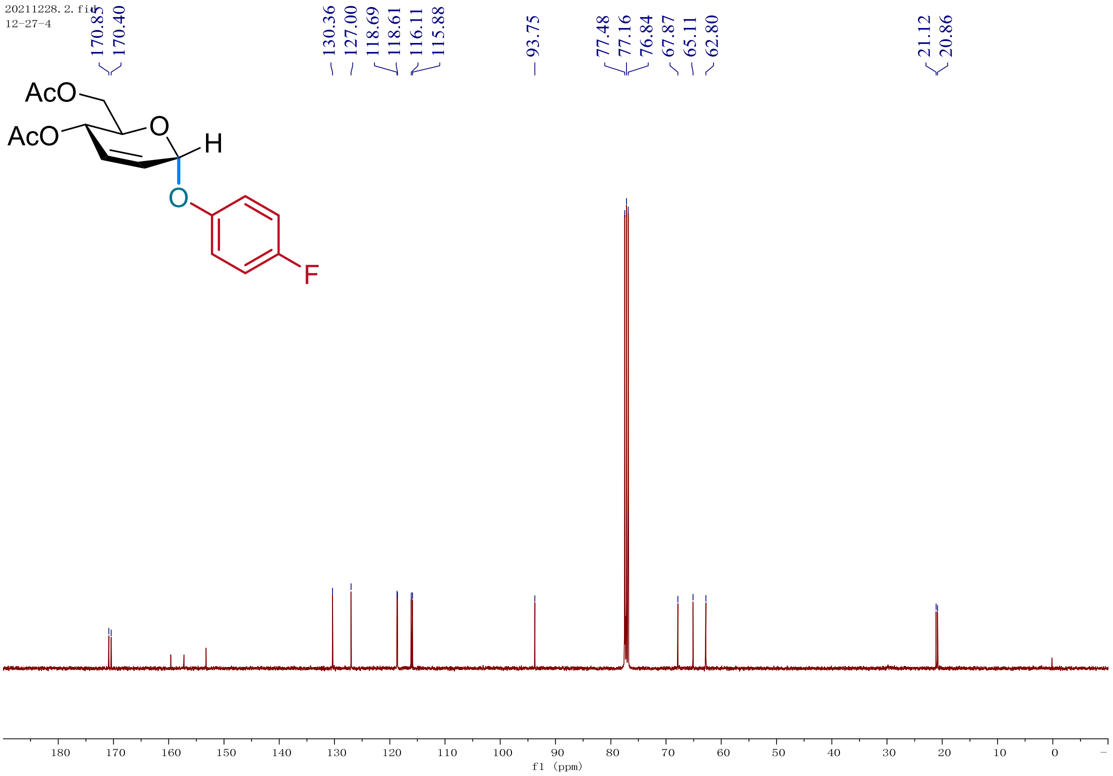

^{1}H NMR of 3ab

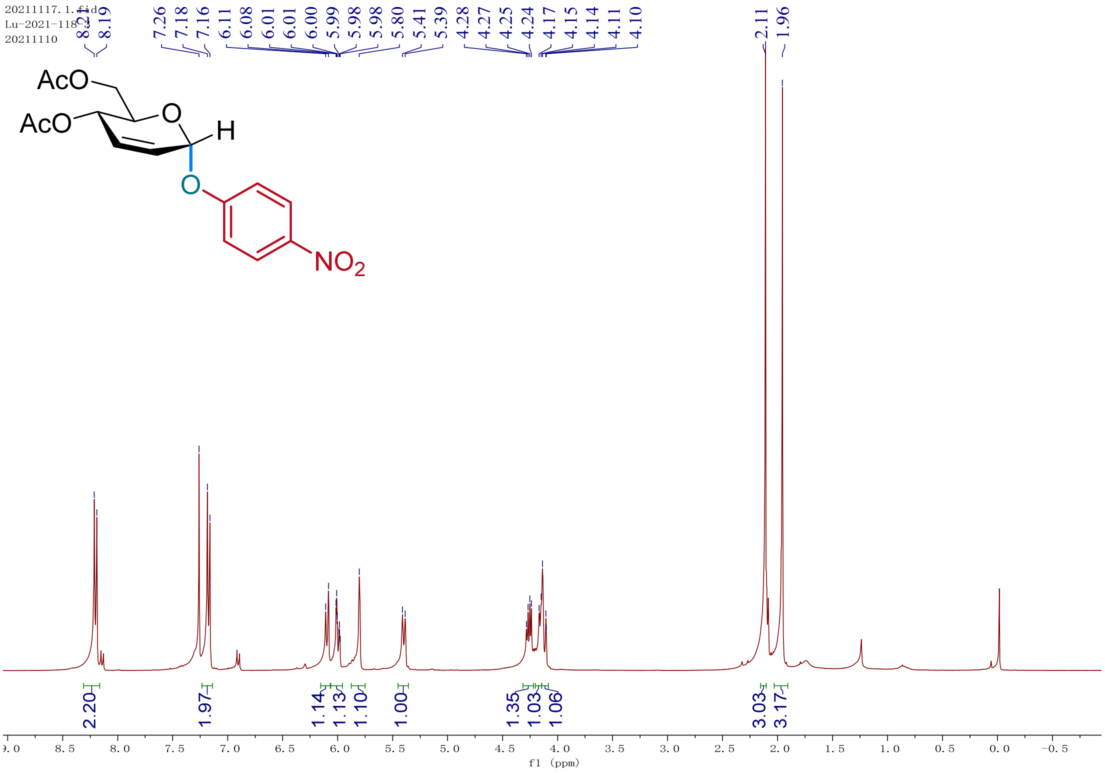

^{13}C NMR of 3ab

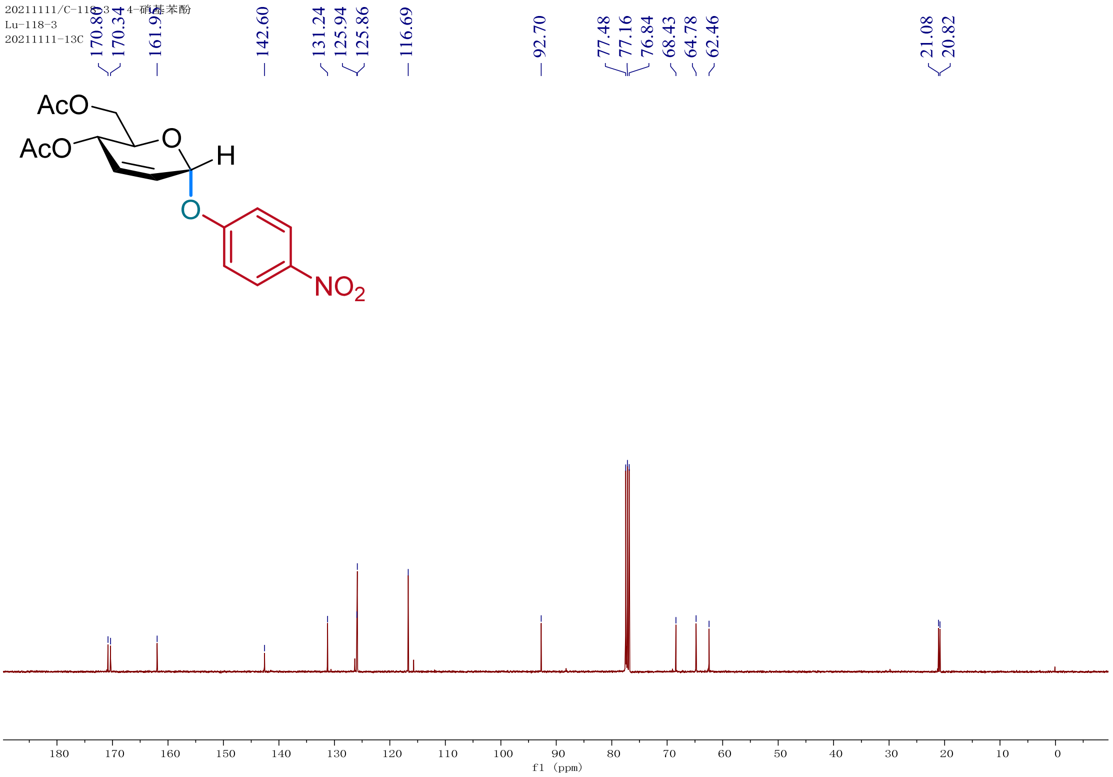

^{1}H NMR of 3ac

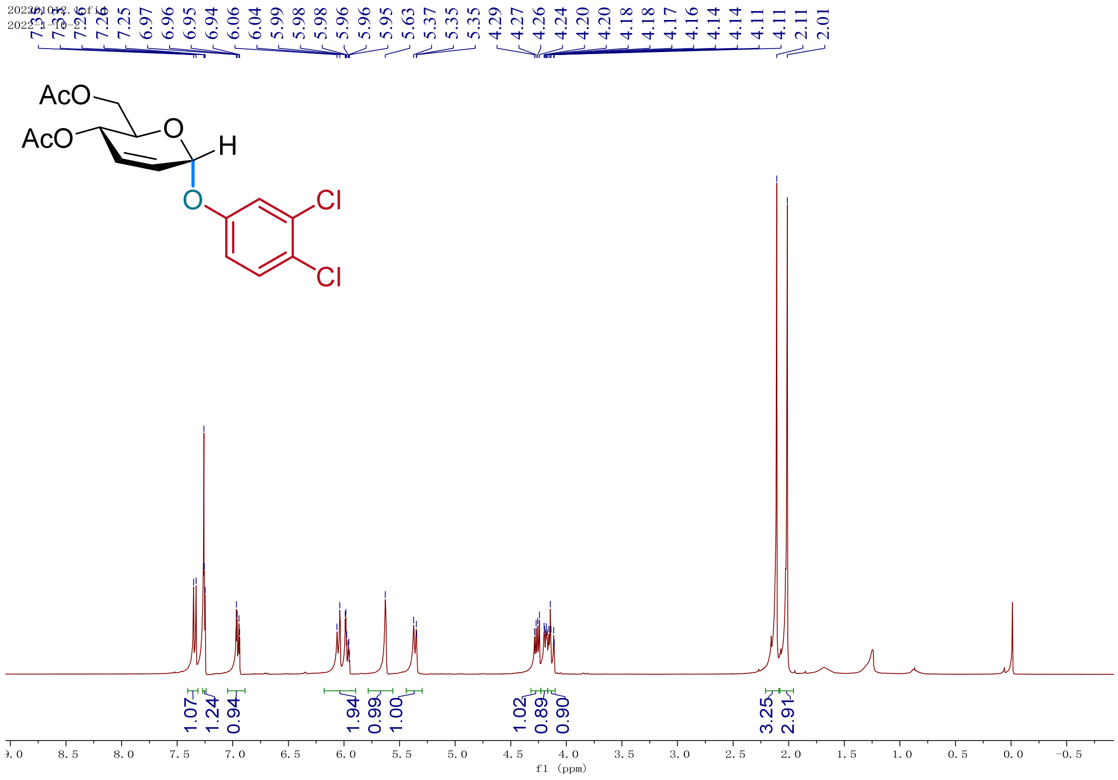

^{13}C NMR of 3ac

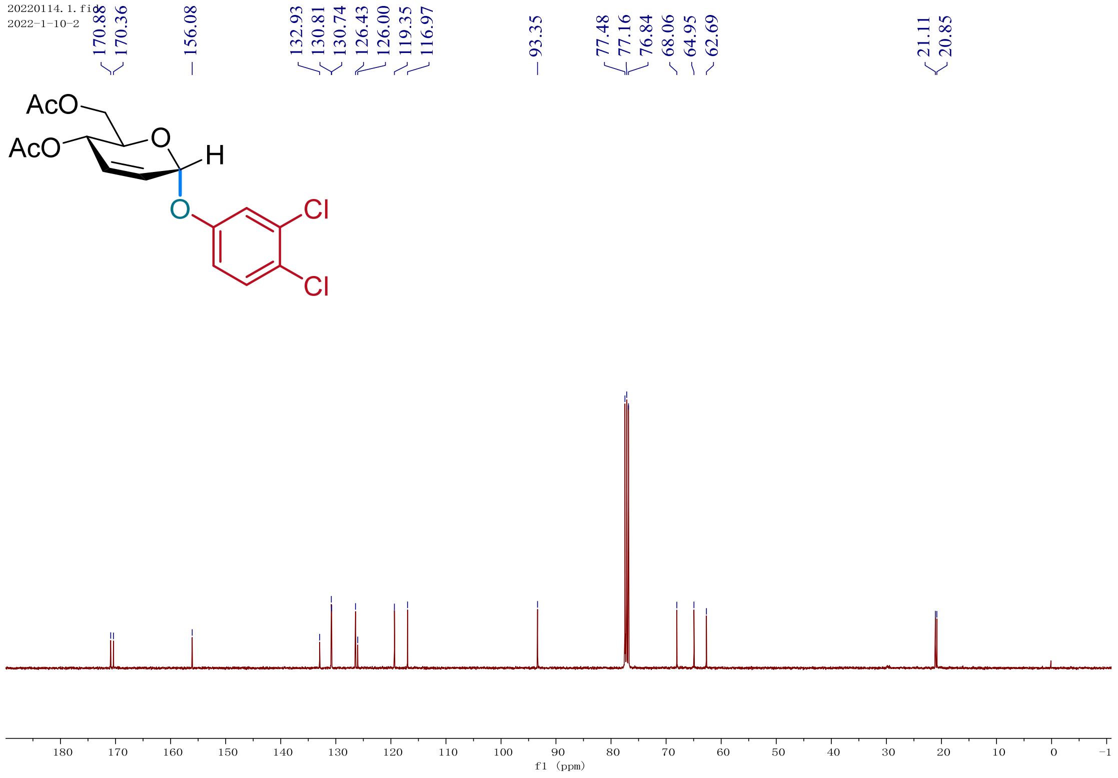

^{1}H NMR of 4a

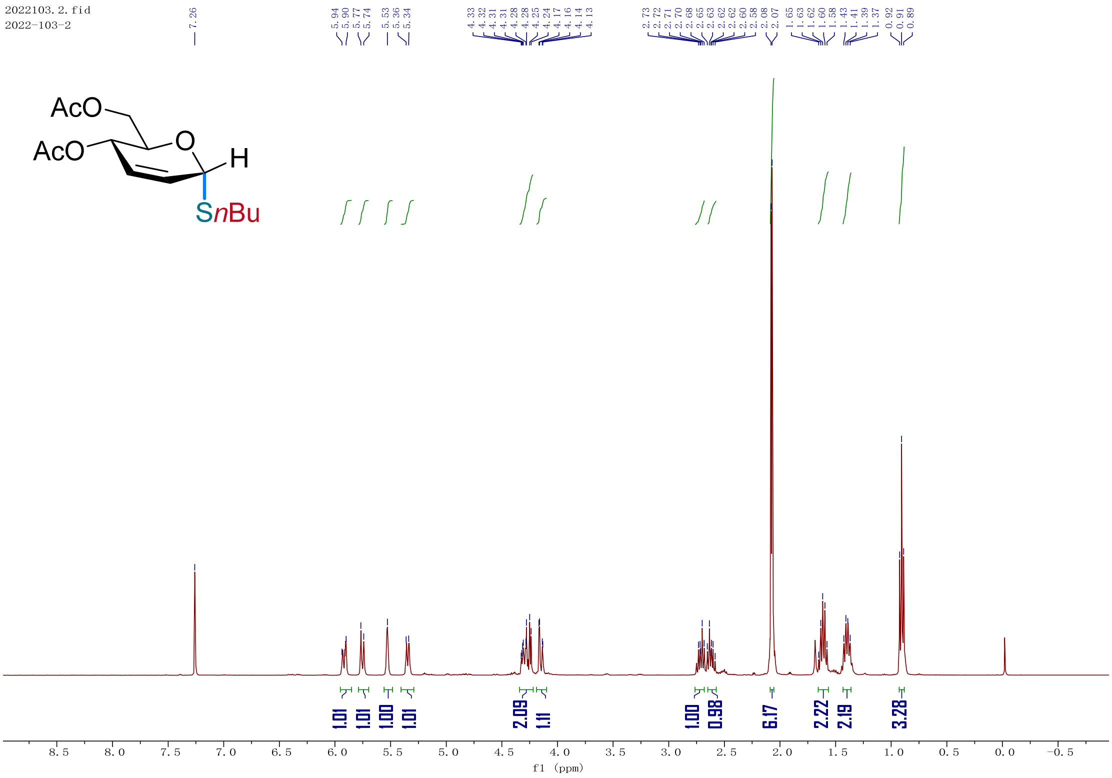

^{13}C NMR of 4a

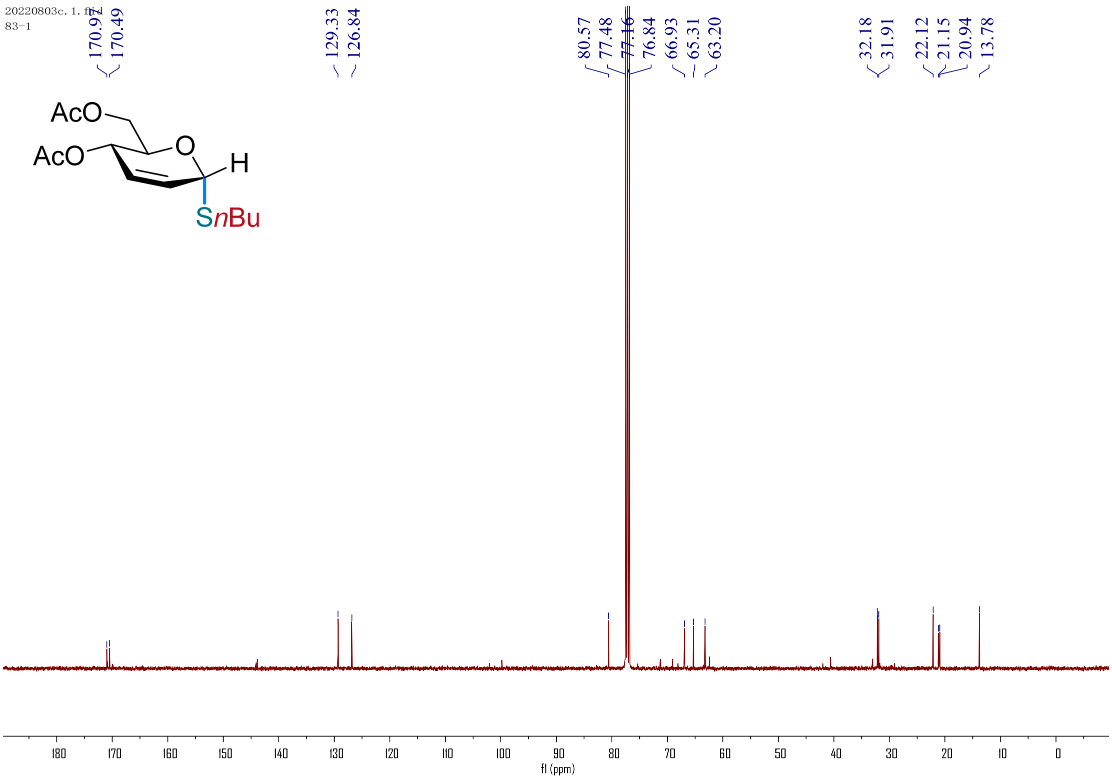

^{1}H NMR of 4b

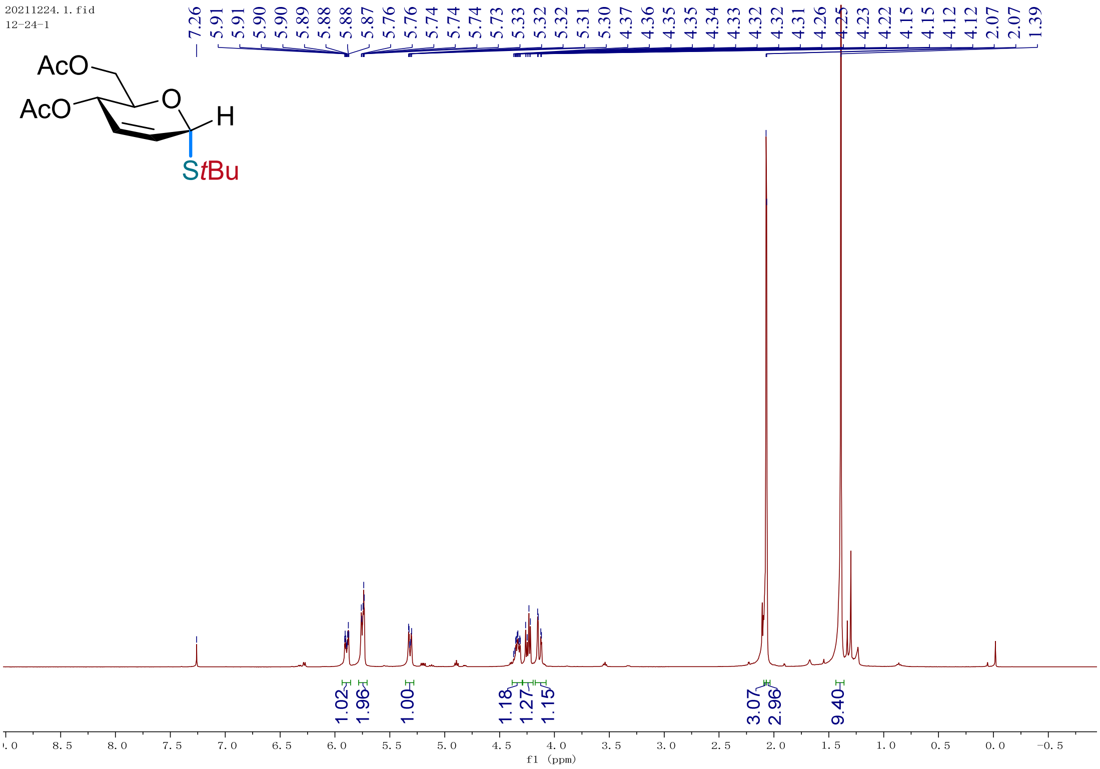

^{13}C NMR of 4b

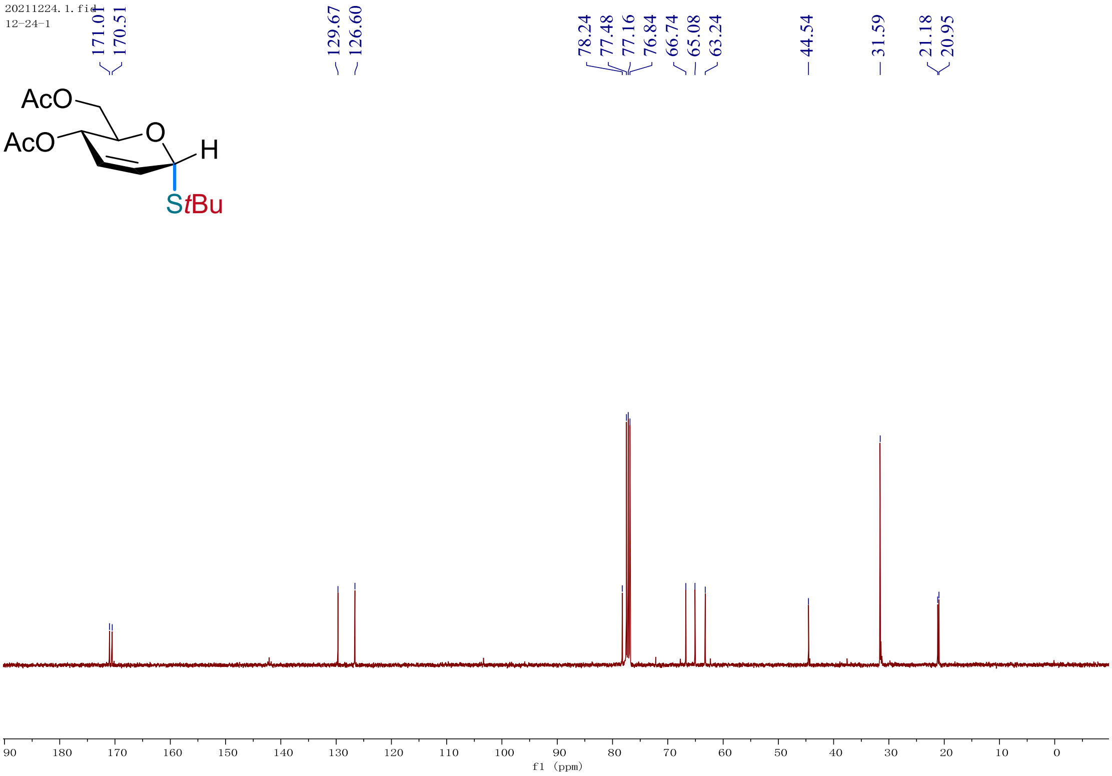

^{1}H NMR of 4c

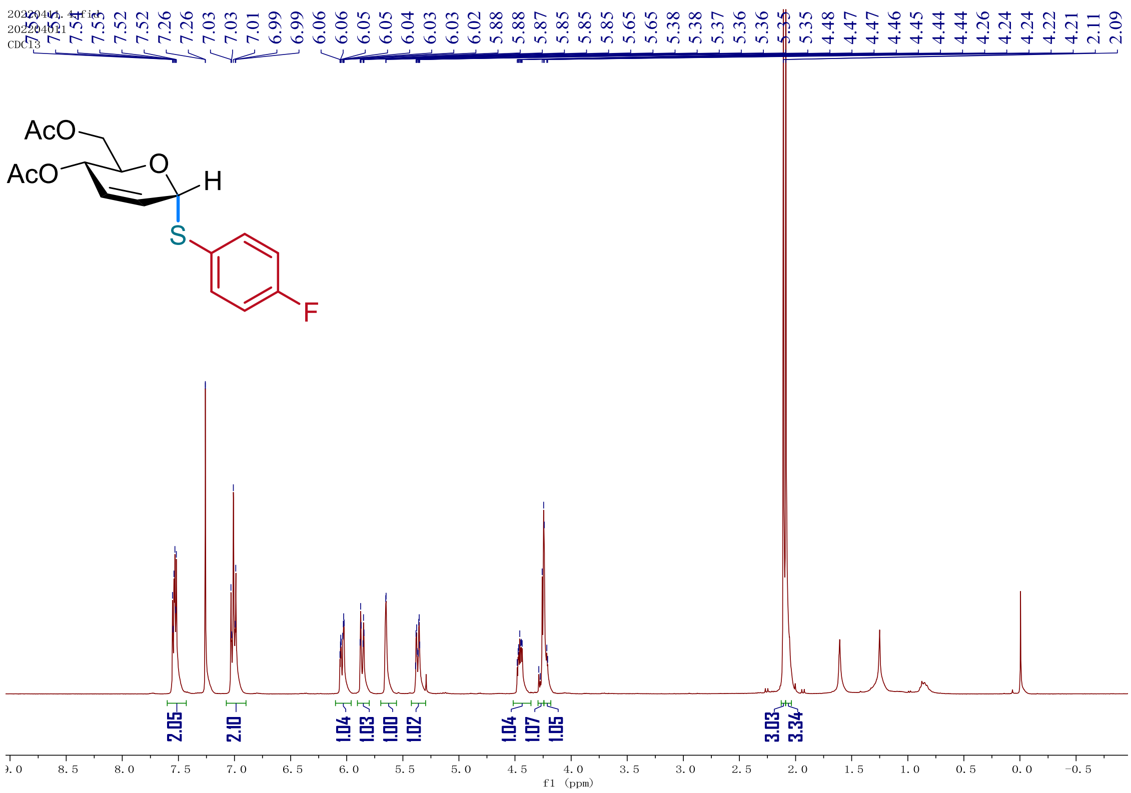

^{13}C NMR of 4c

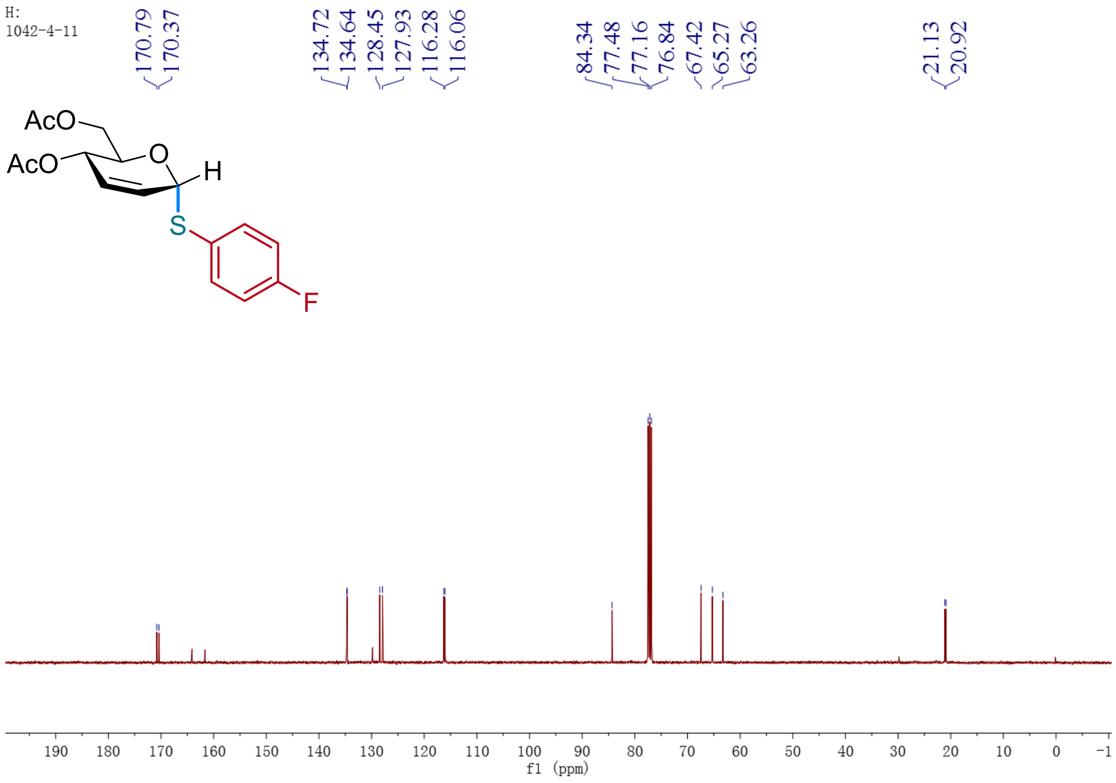

^{1}H NMR of 4c'

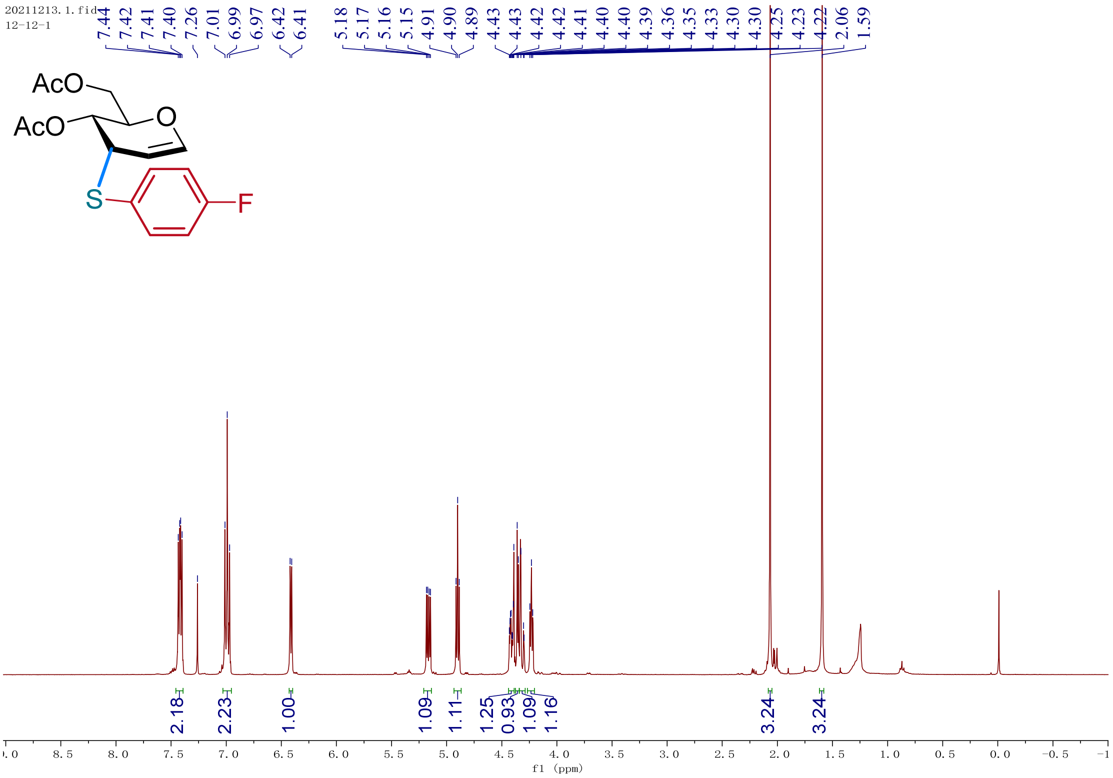

^{13}C NMR of 4c'

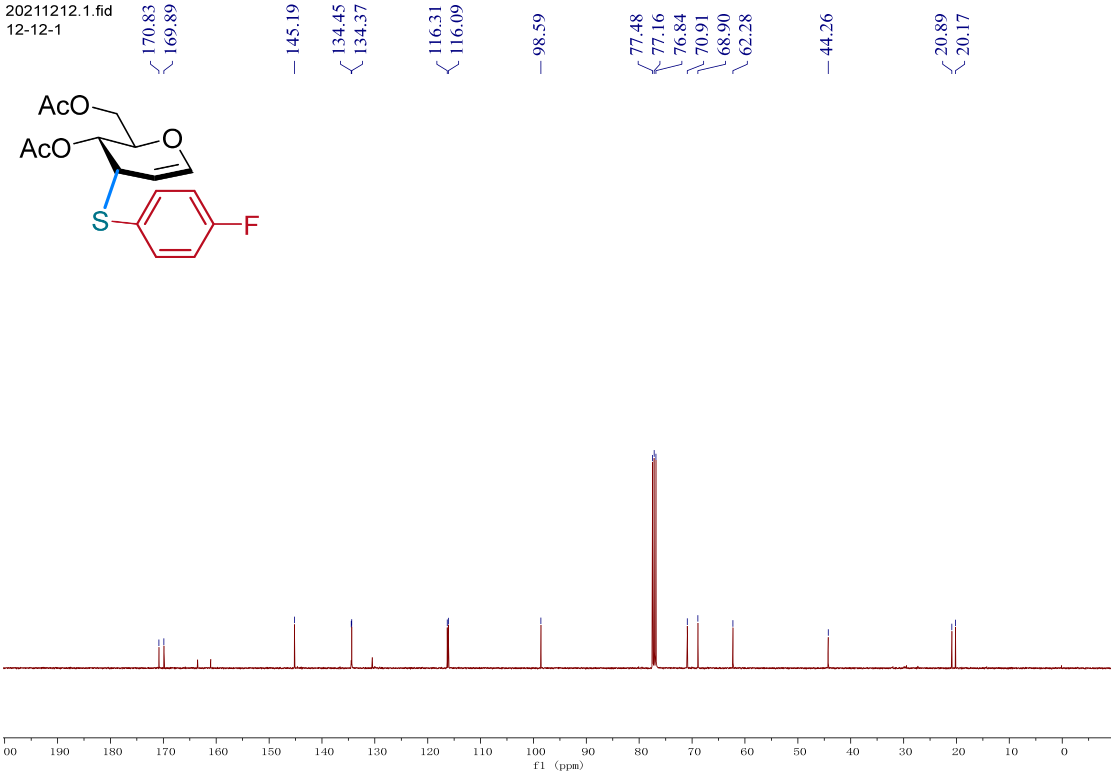

^{1}H NMR of 4d

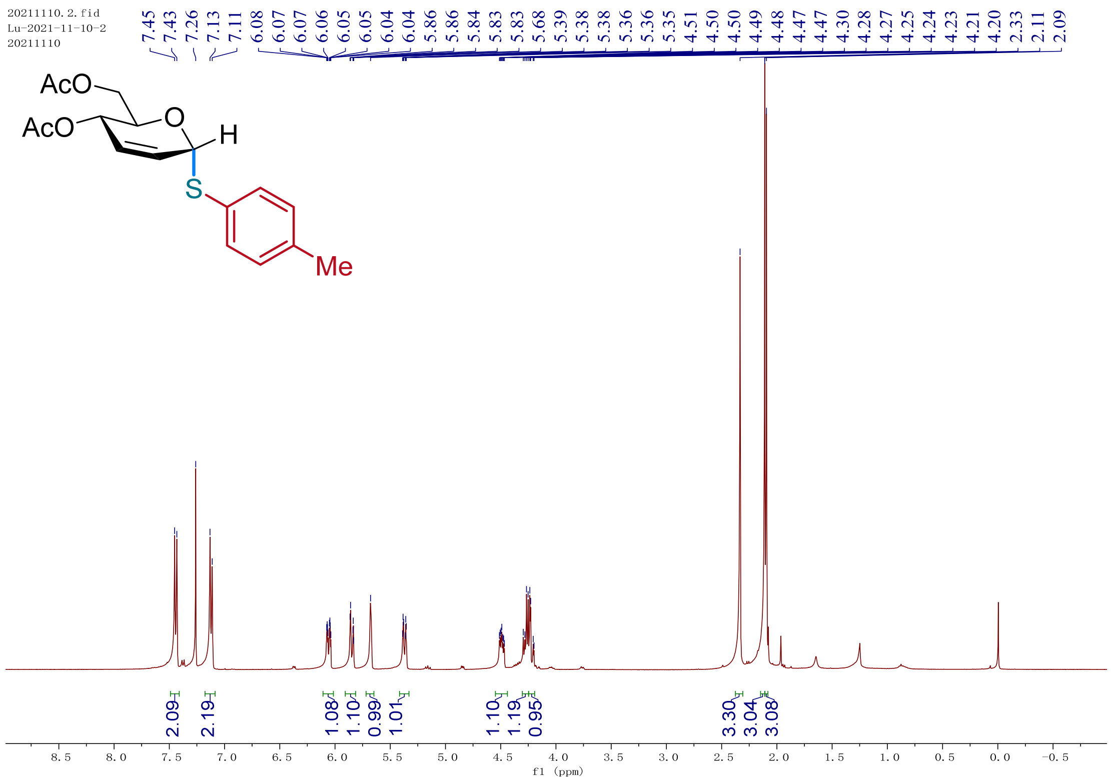

^{13}C NMR of 4d

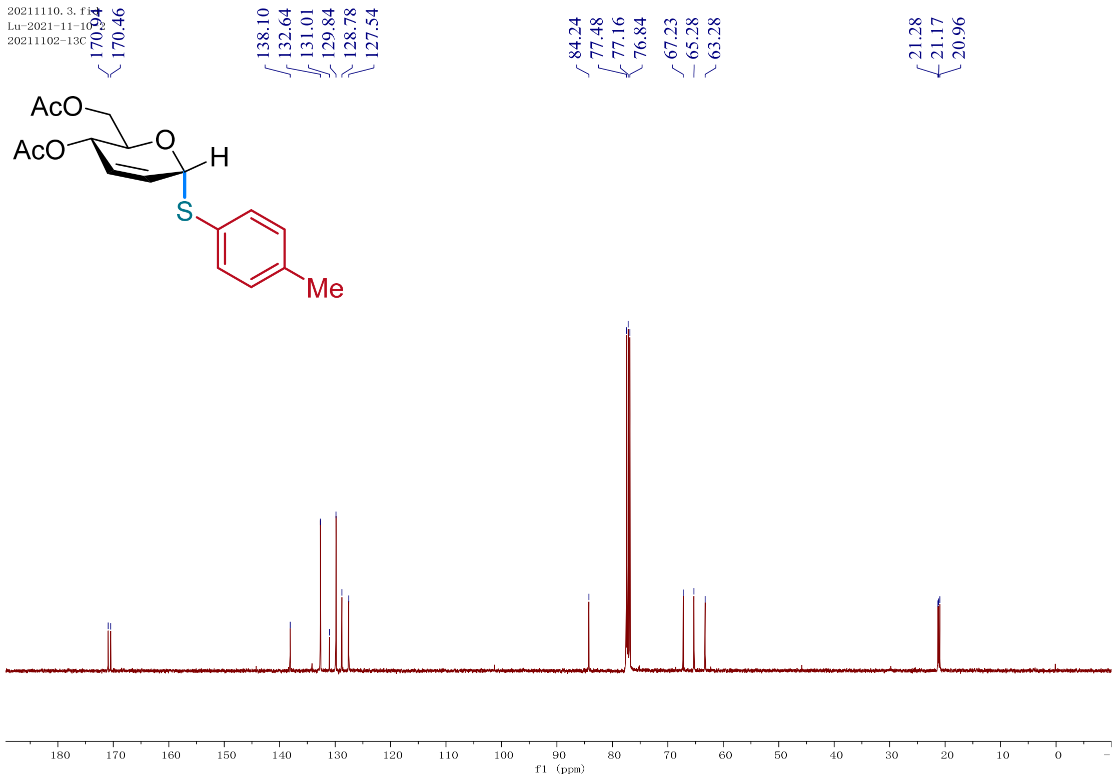

^{1}H NMR of 4e

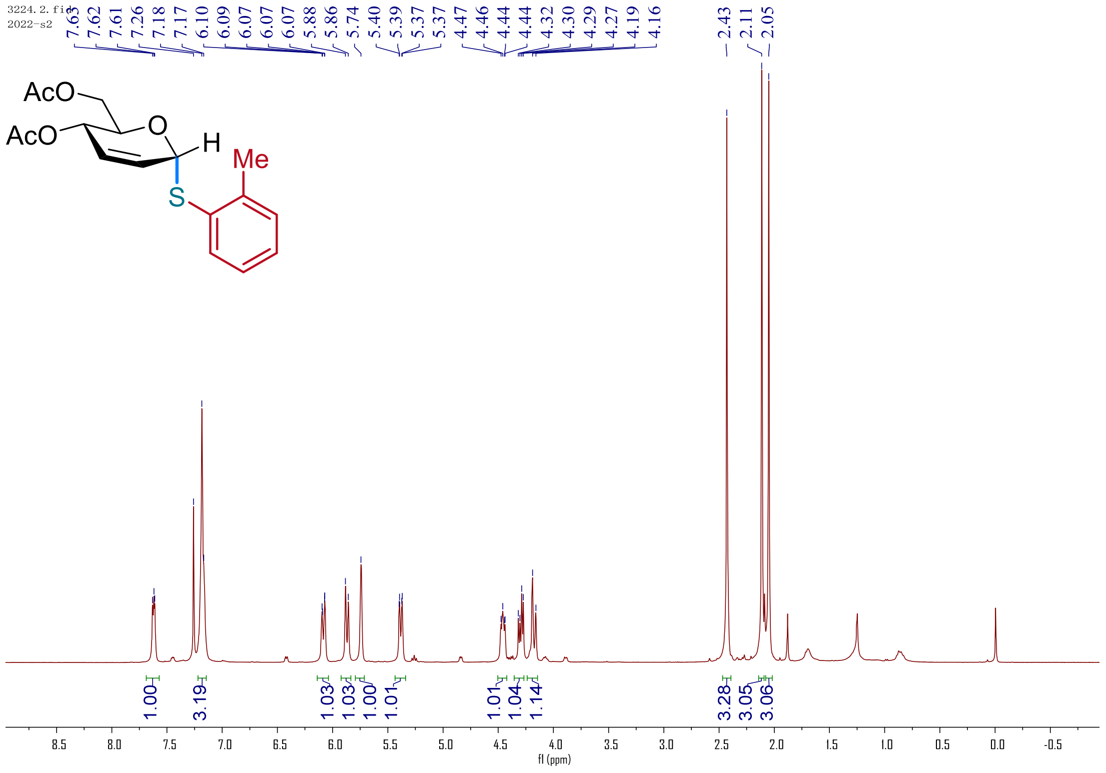

^{13}C NMR of 4e

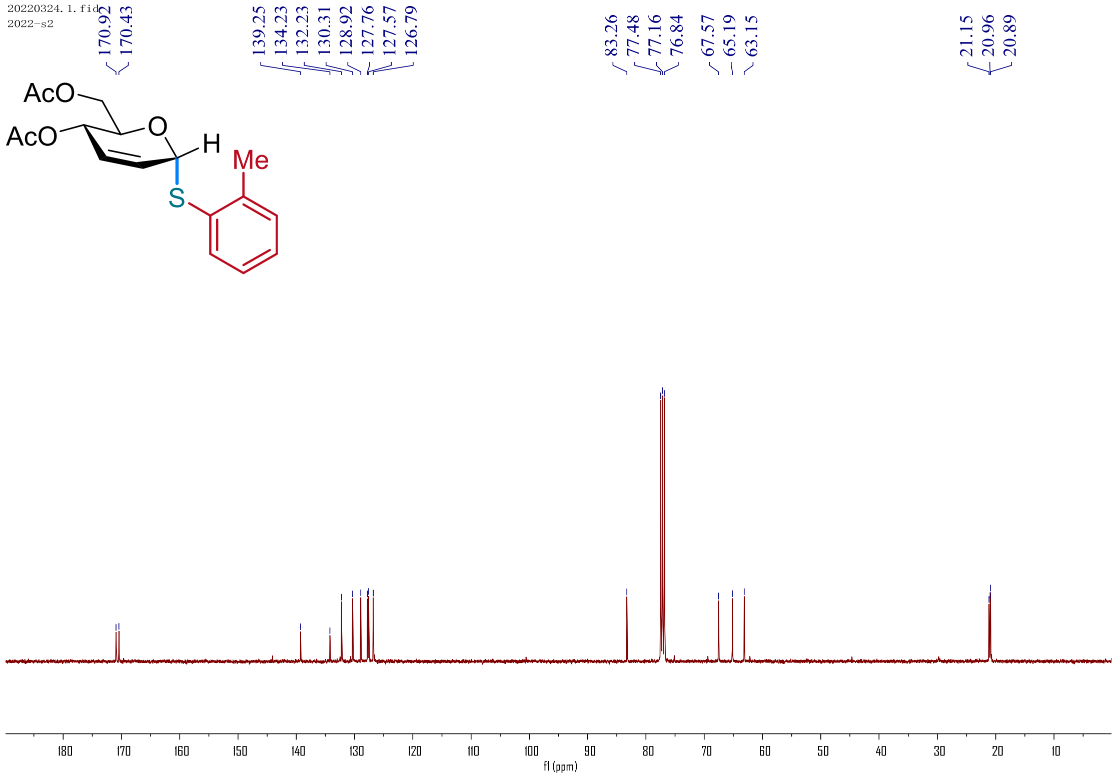

^{1}H NMR of 4e’

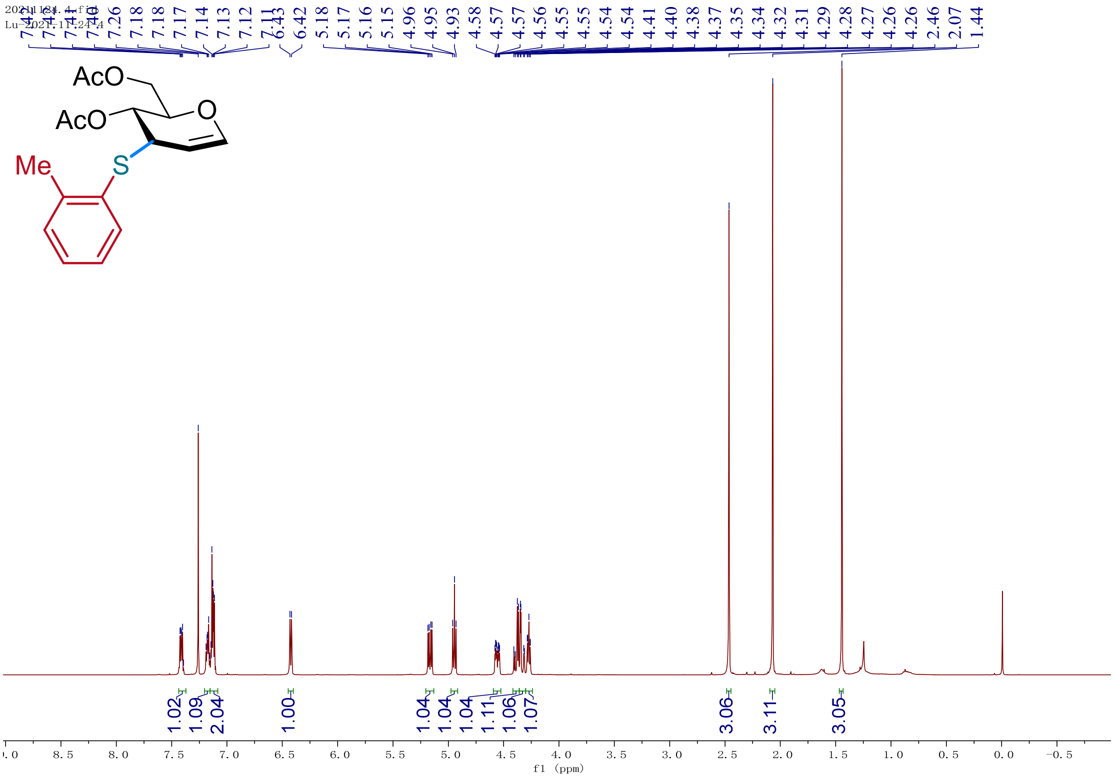

^{13}C NMR of 4e’

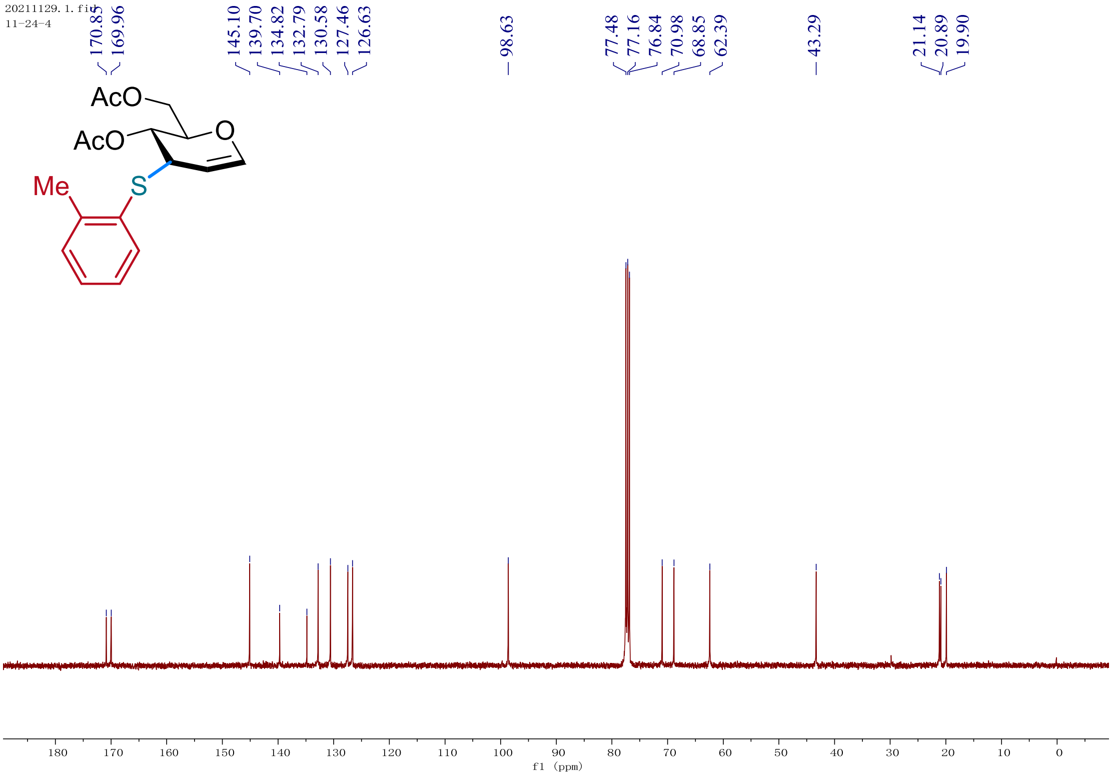

^{1}H NMR of 5

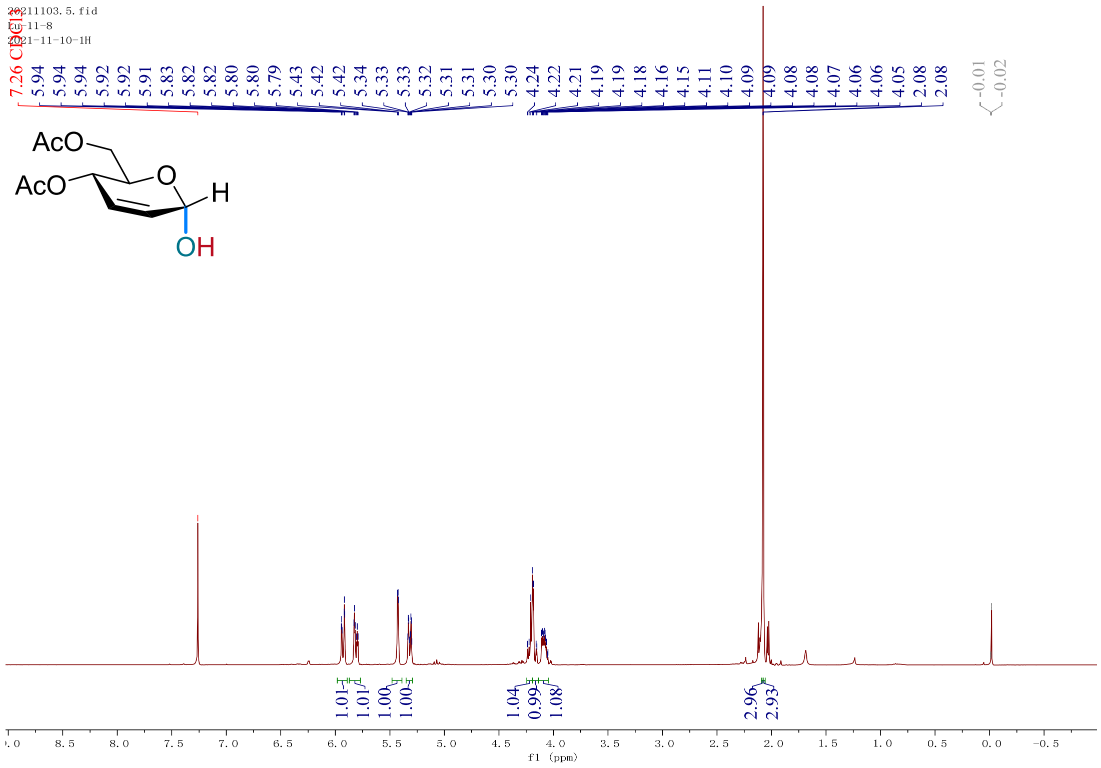

^{13}C NMR of 5

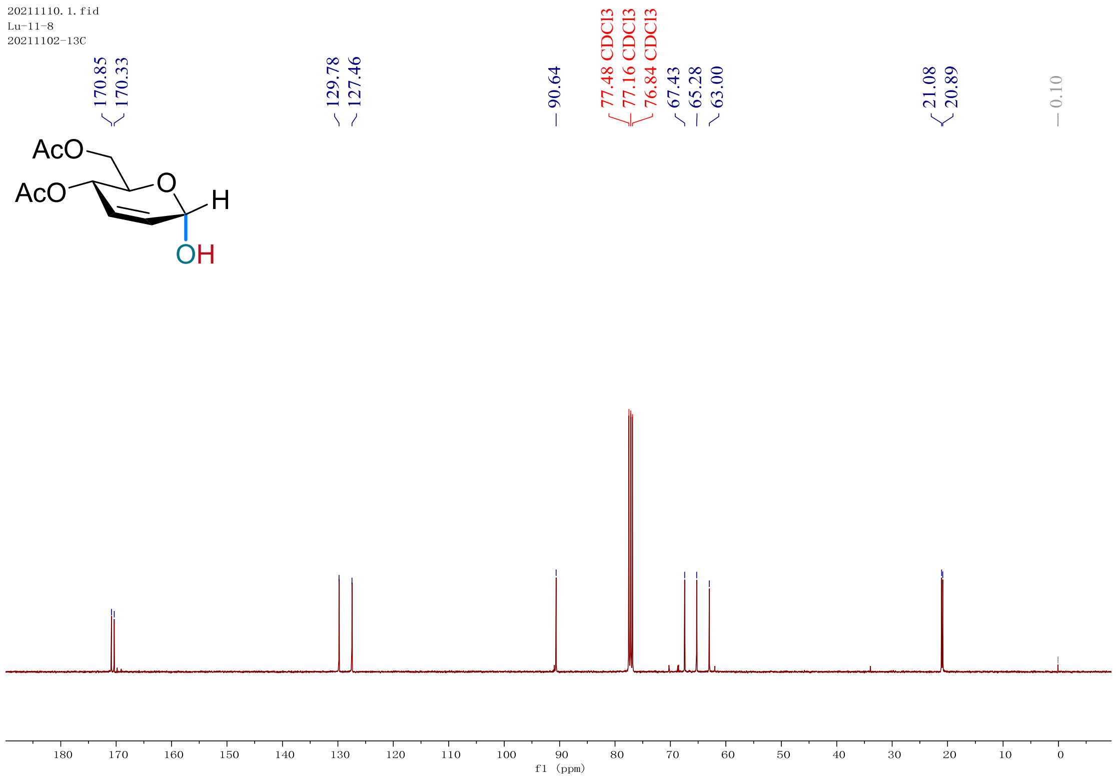

^{1}H NMR of 6a

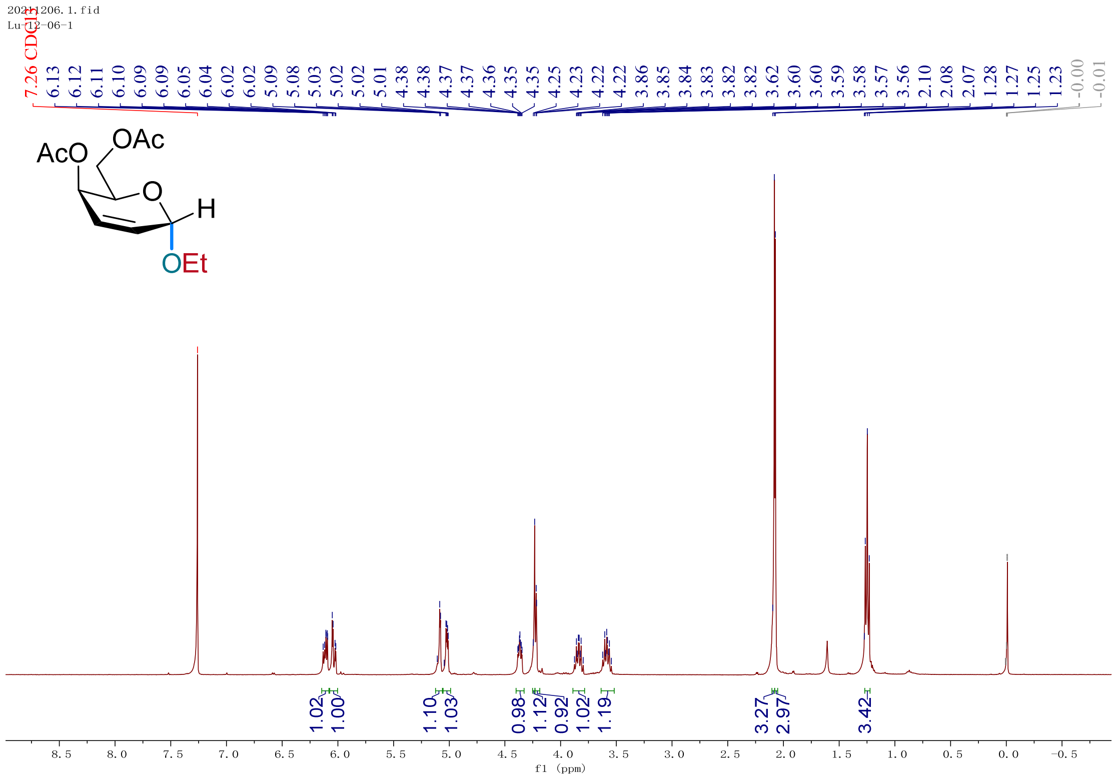

^{13}C NMR of 6a

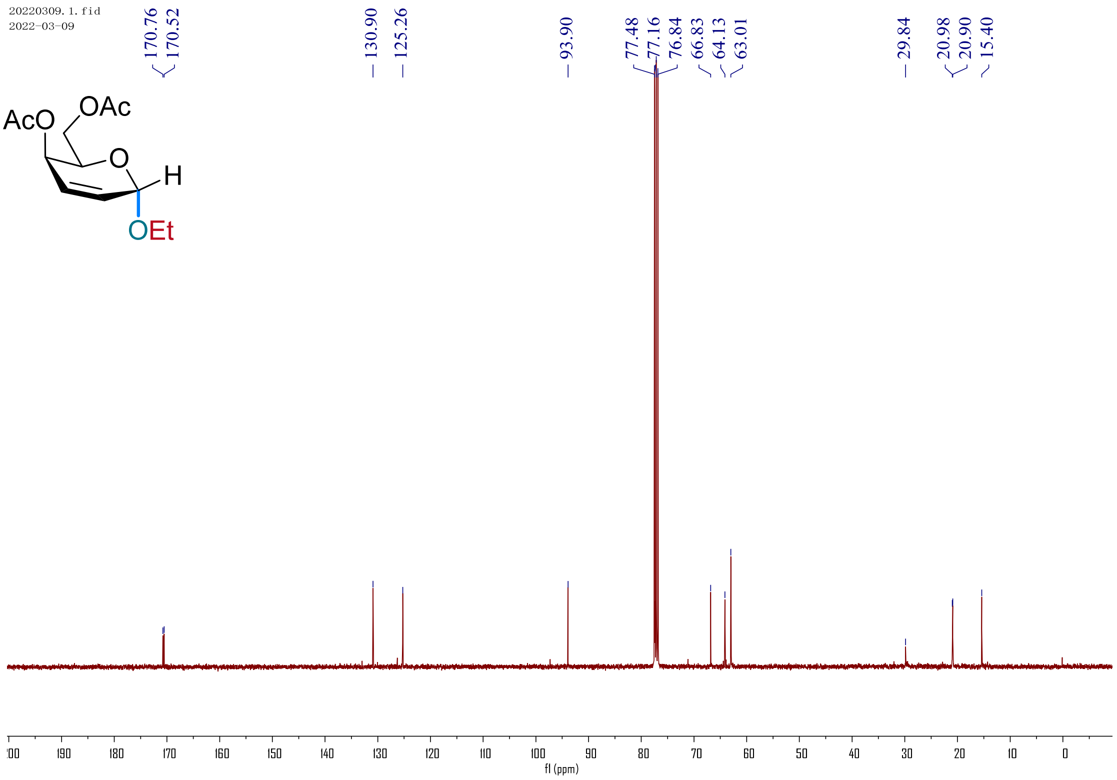

^{1}H NMR of 6b

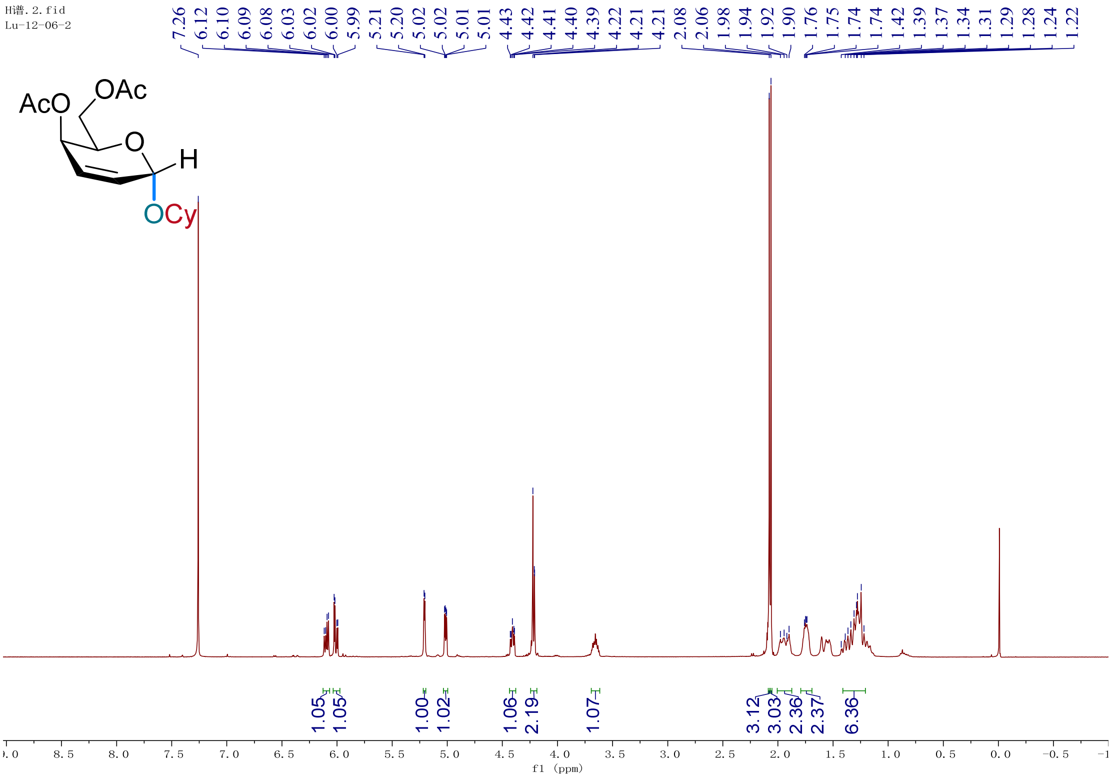

^{13}C NMR of 6b

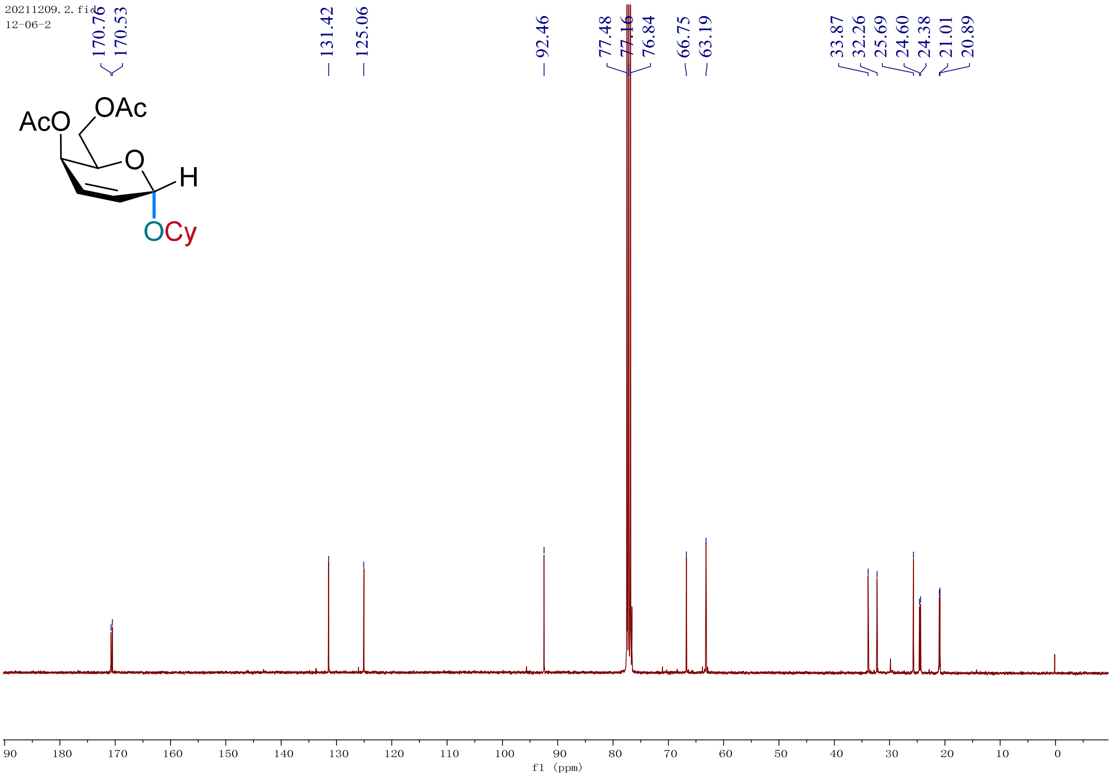

^{1}H NMR of 6c

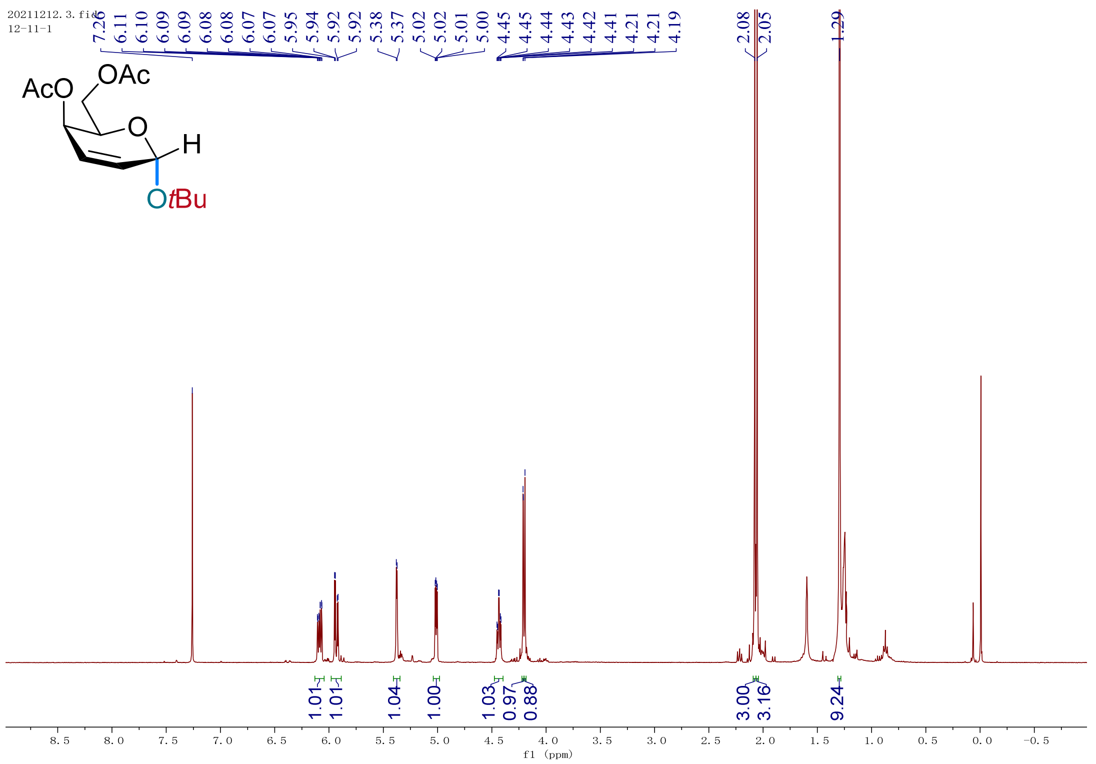

^{13}C NMR of 6c

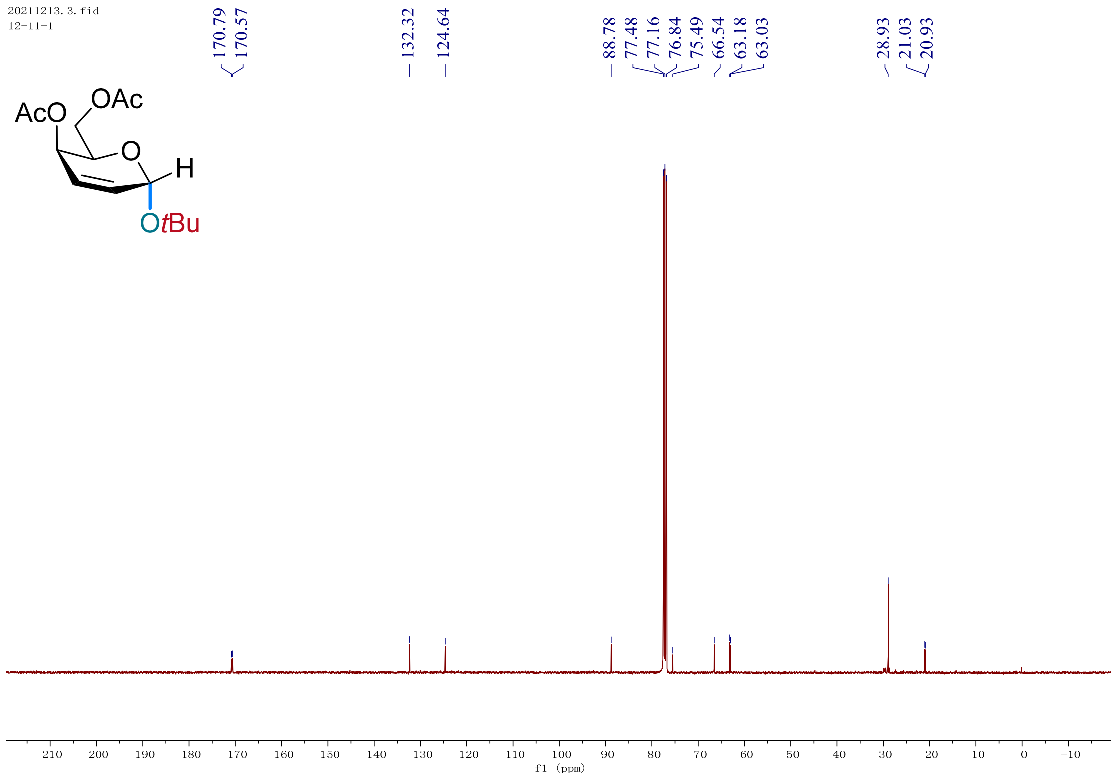

^{1}H NMR of 6d

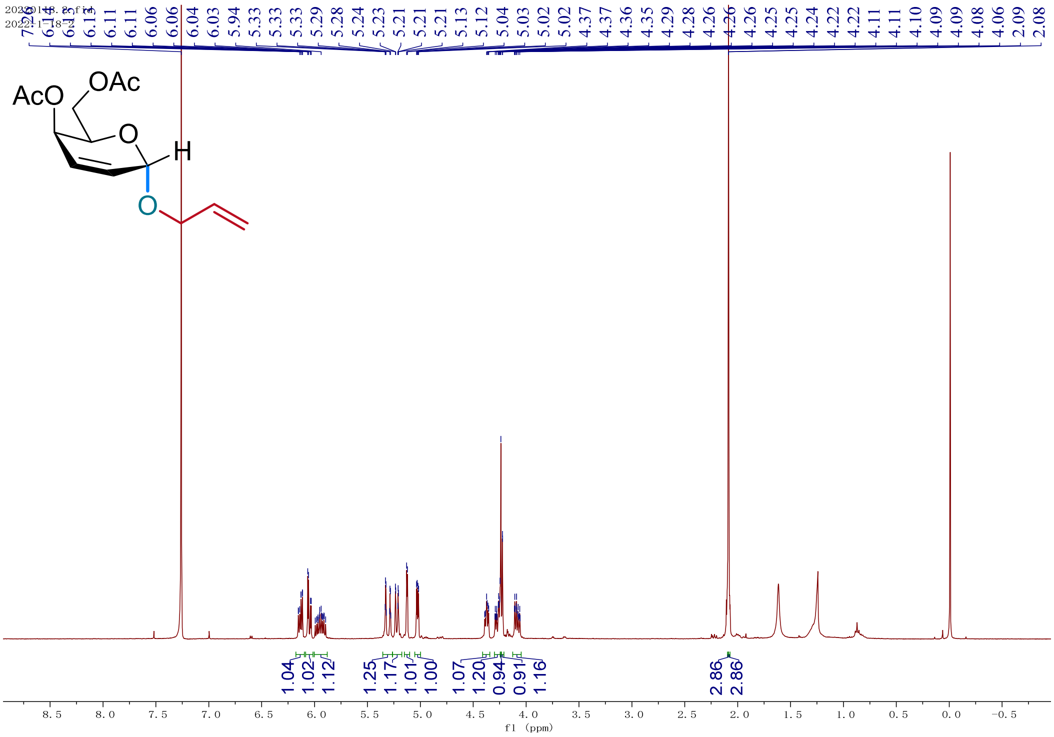

^{13}C NMR of 6d

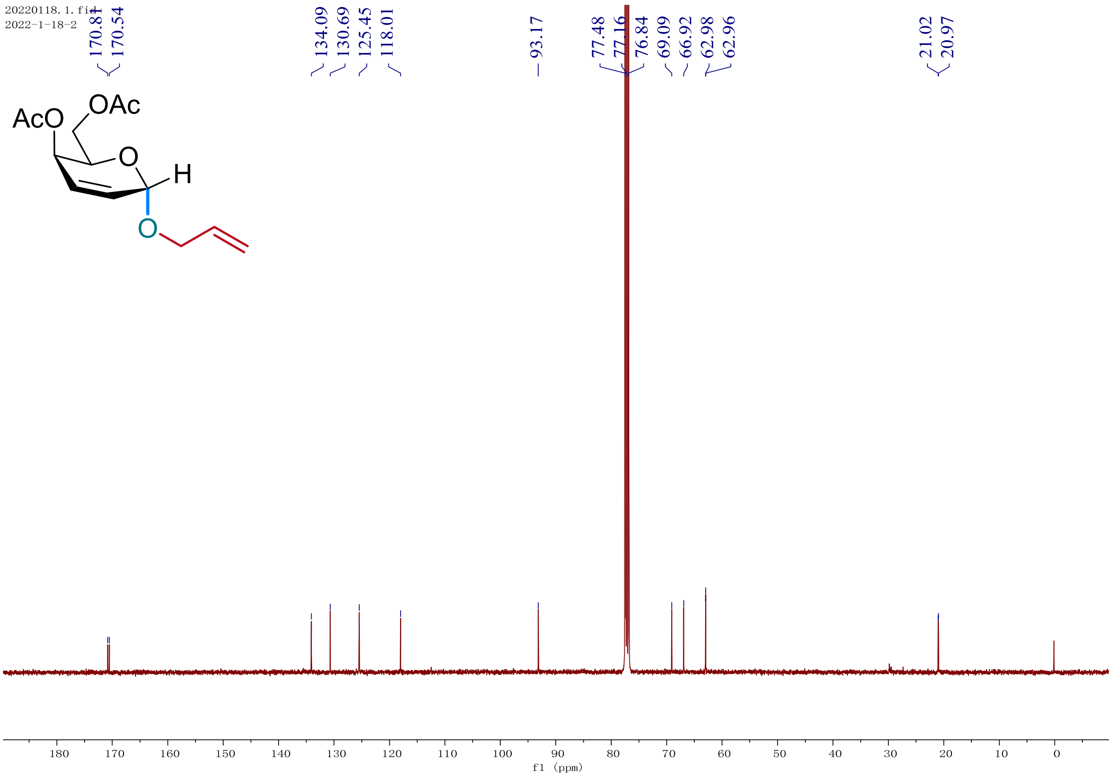

^{1}H NMR of 6e

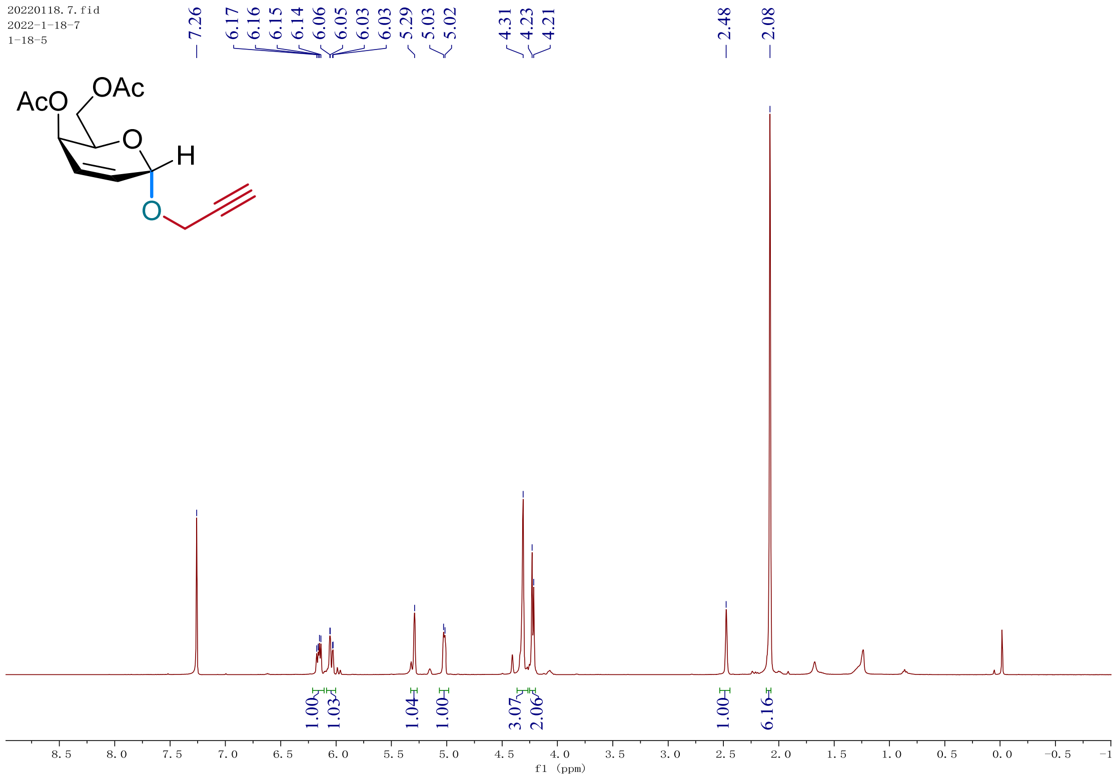

^{13}C NMR of 6e

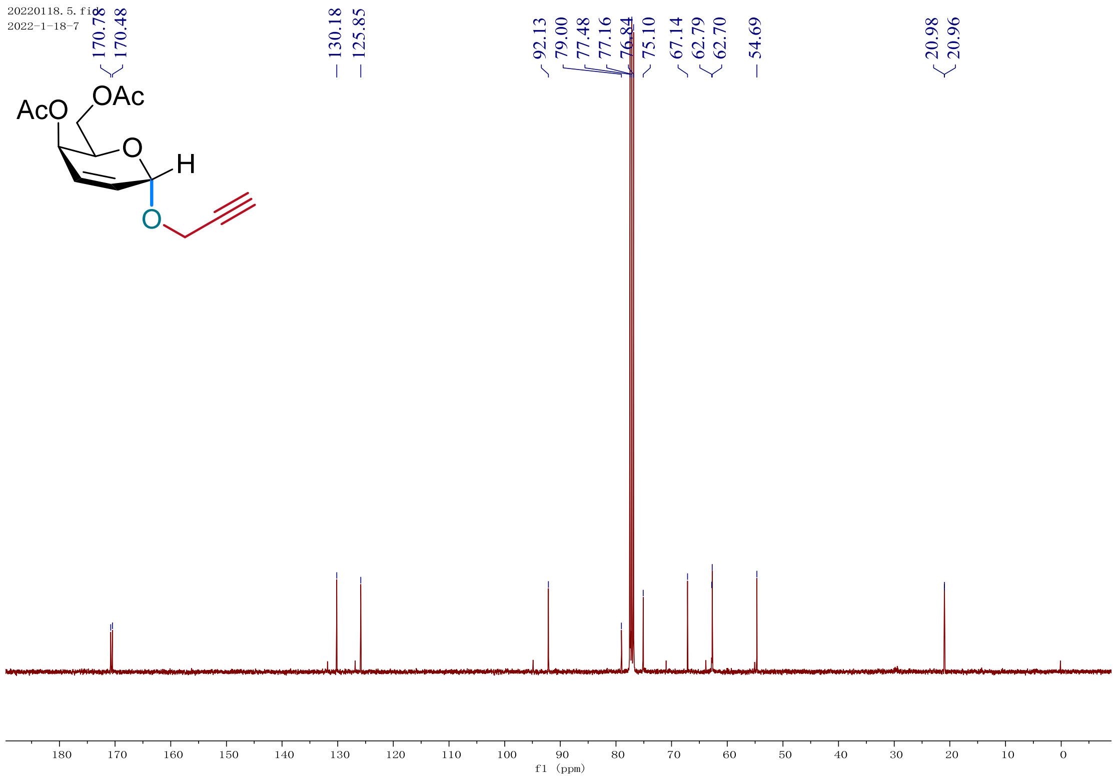

^{1}H NMR of 6f

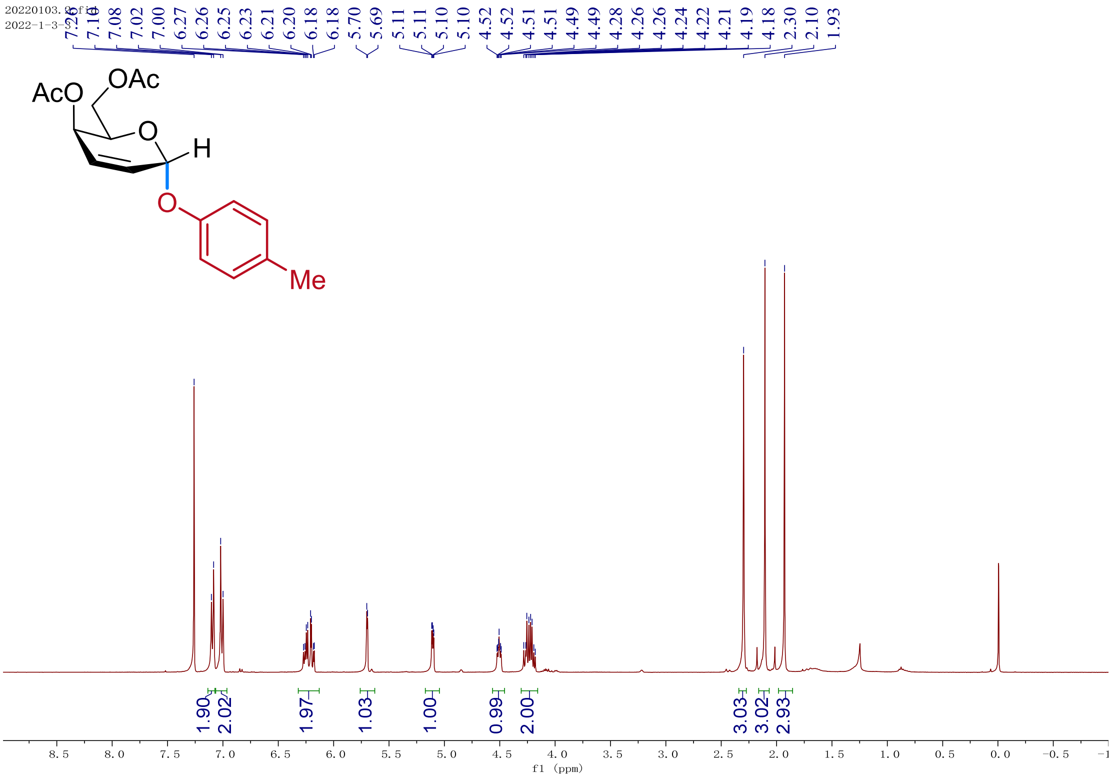

^{13}C NMR of 6f

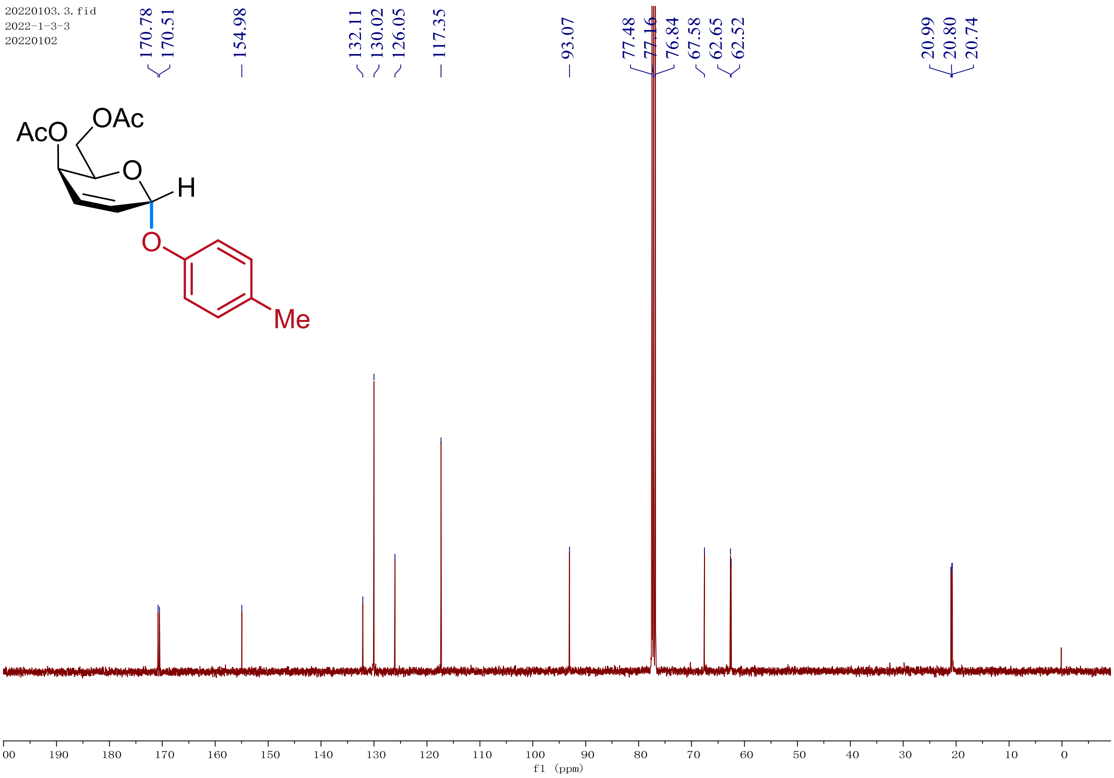

^{1}H NMR of 6g

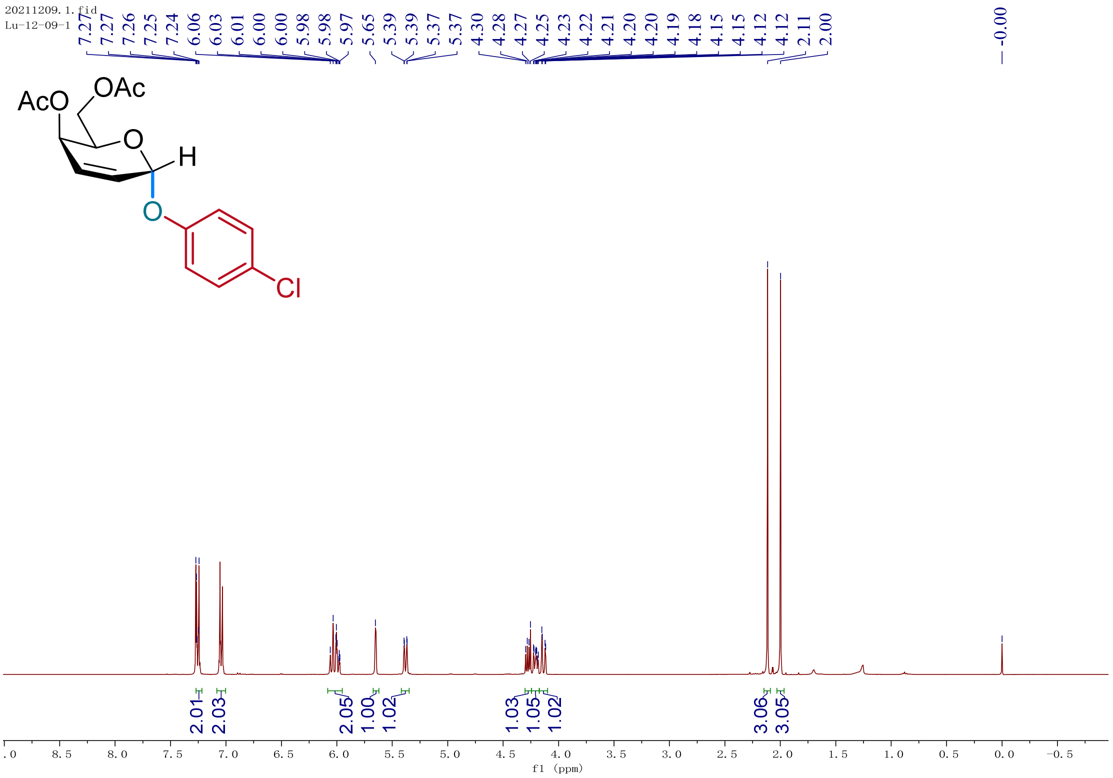

^{13}C NMR of 6g

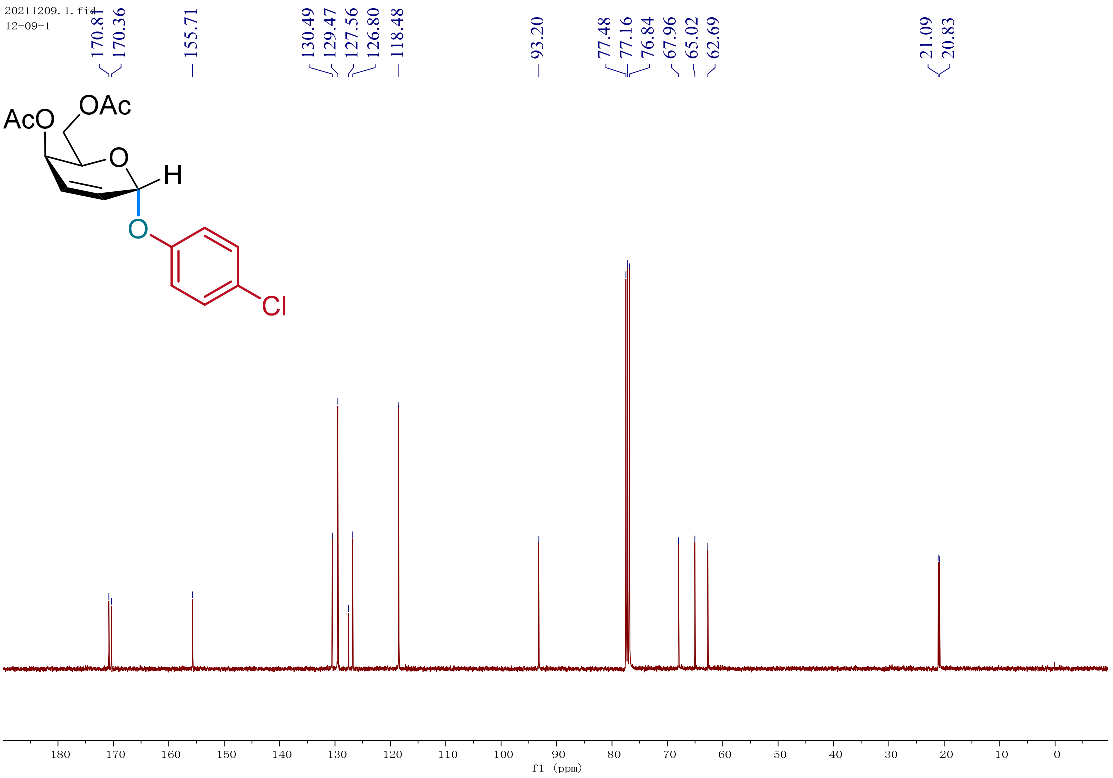

^{1}H NMR of 6h

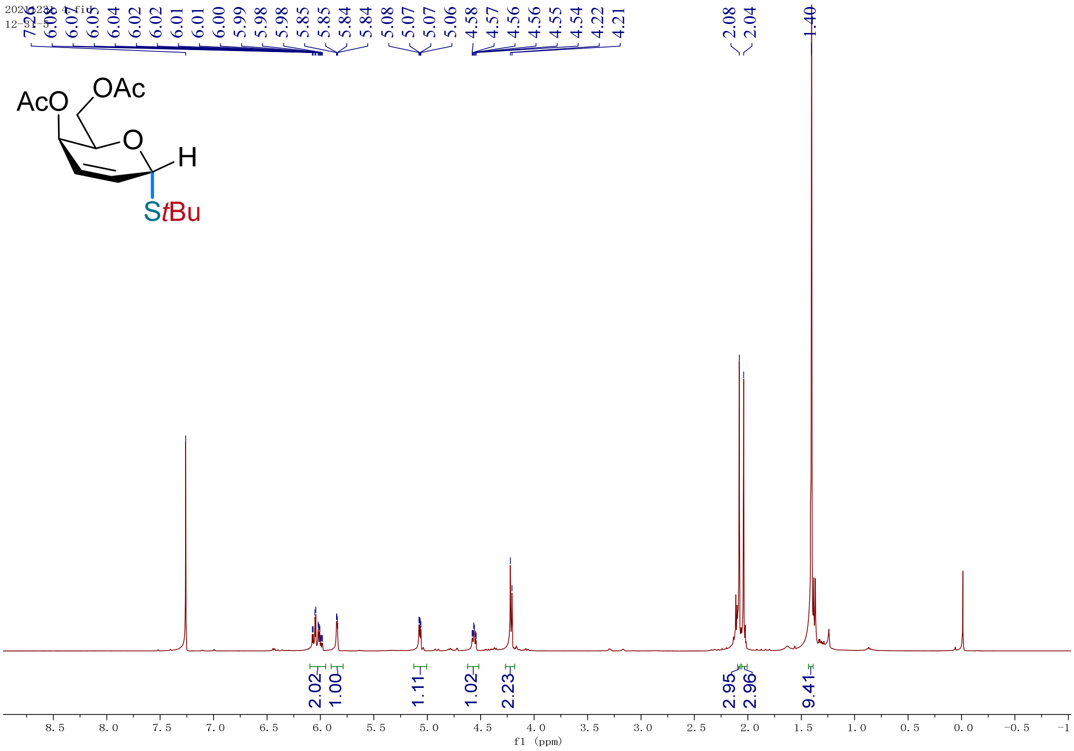

^{13}C NMR of 6h

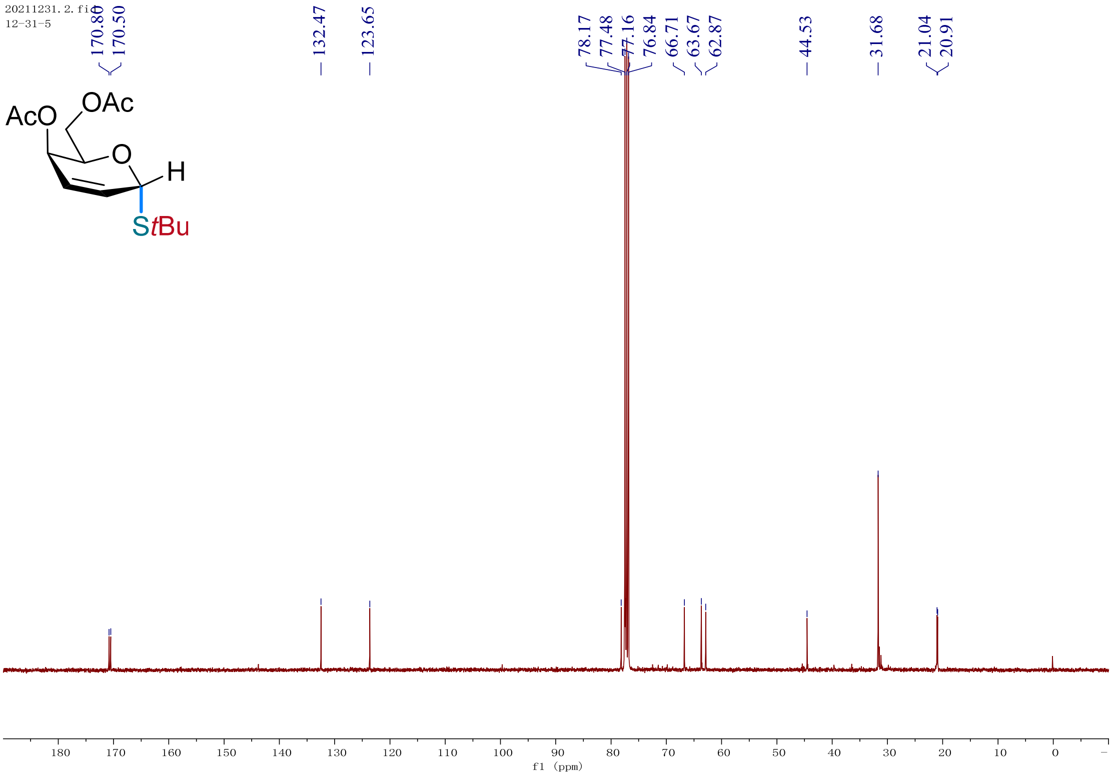

^{1}H NMR of 6i

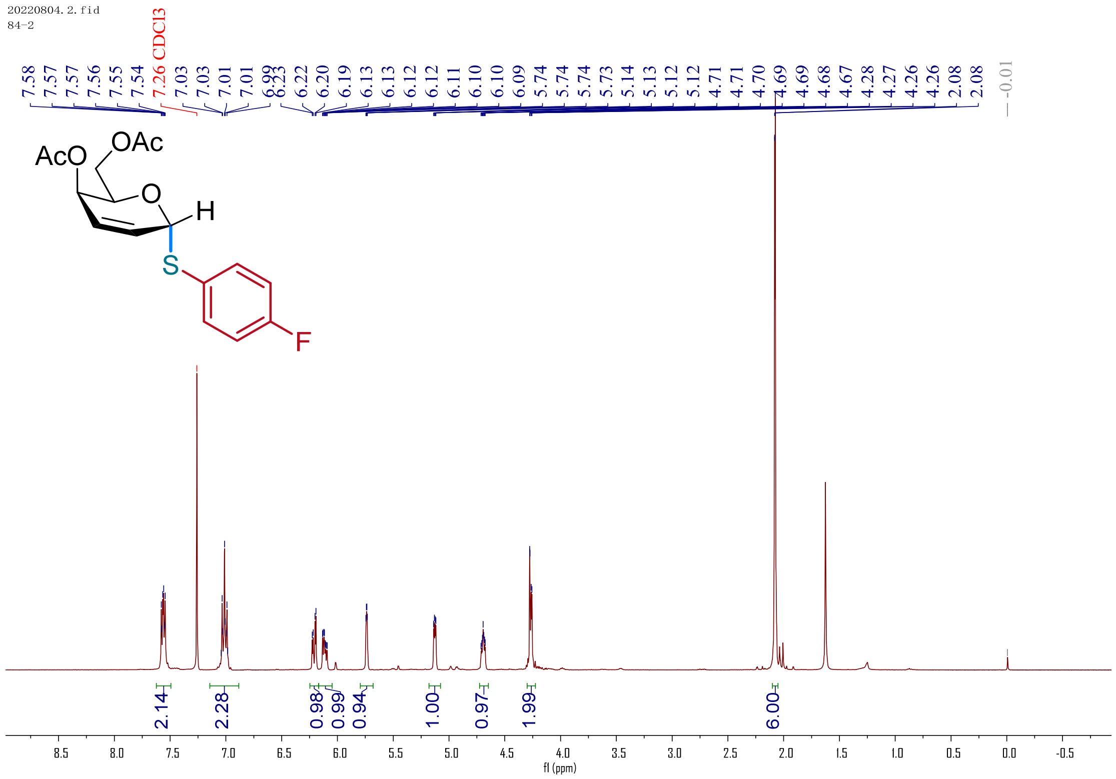

^{13}C NMR of 6i

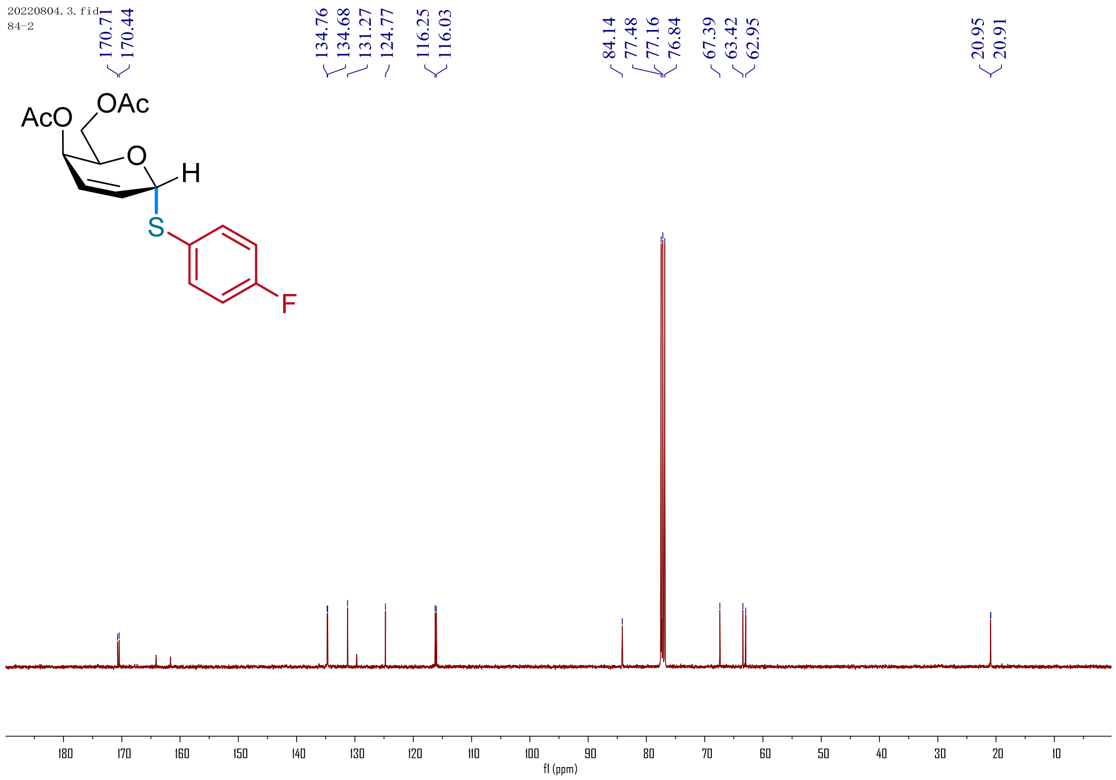

^{1}H NMR of 7a

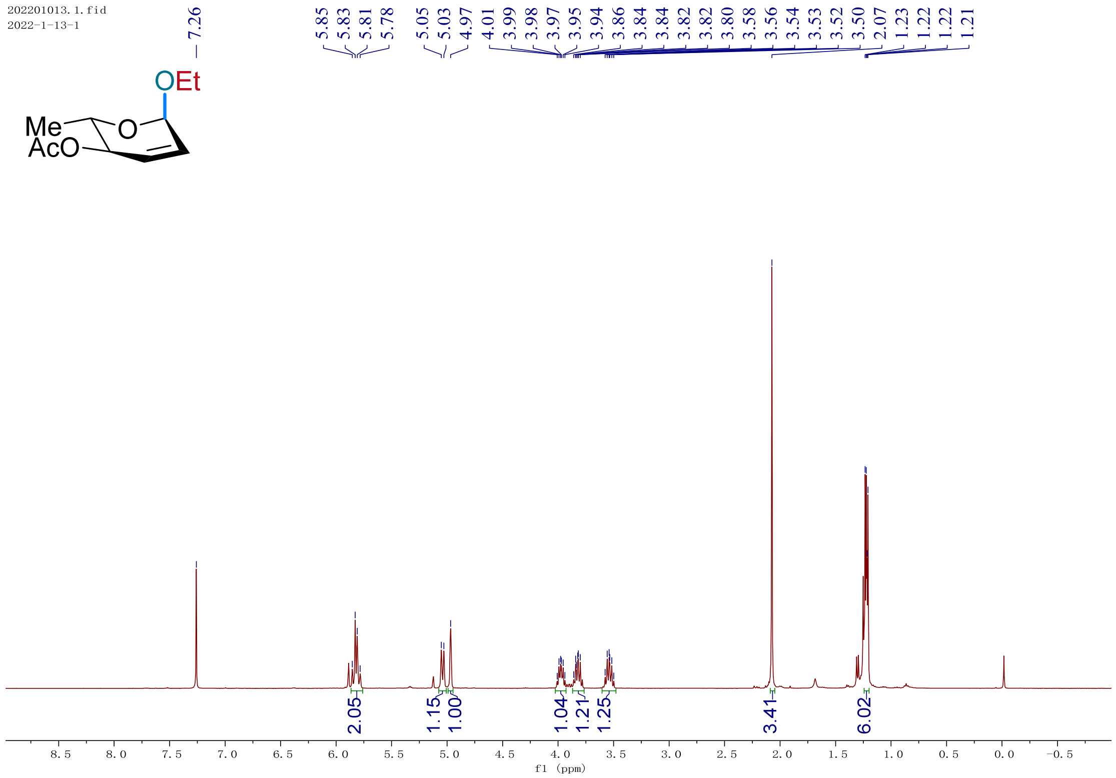

^{13}C NMR of 7a

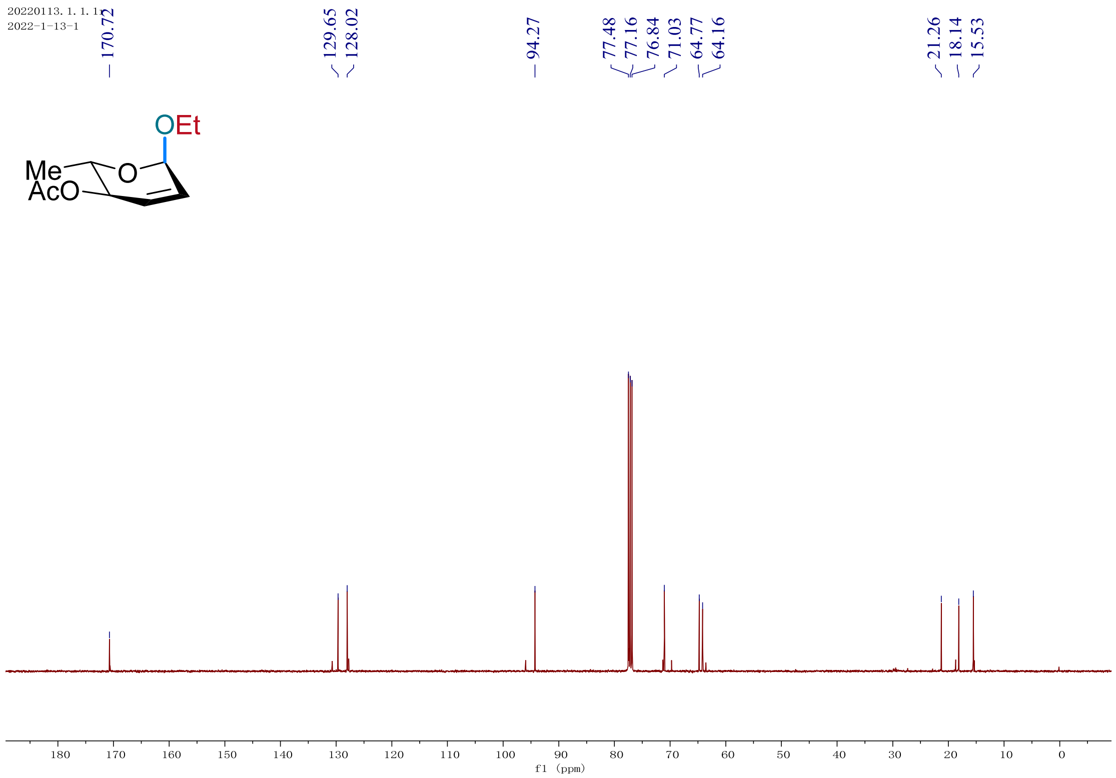

^{1}H NMR of 7b

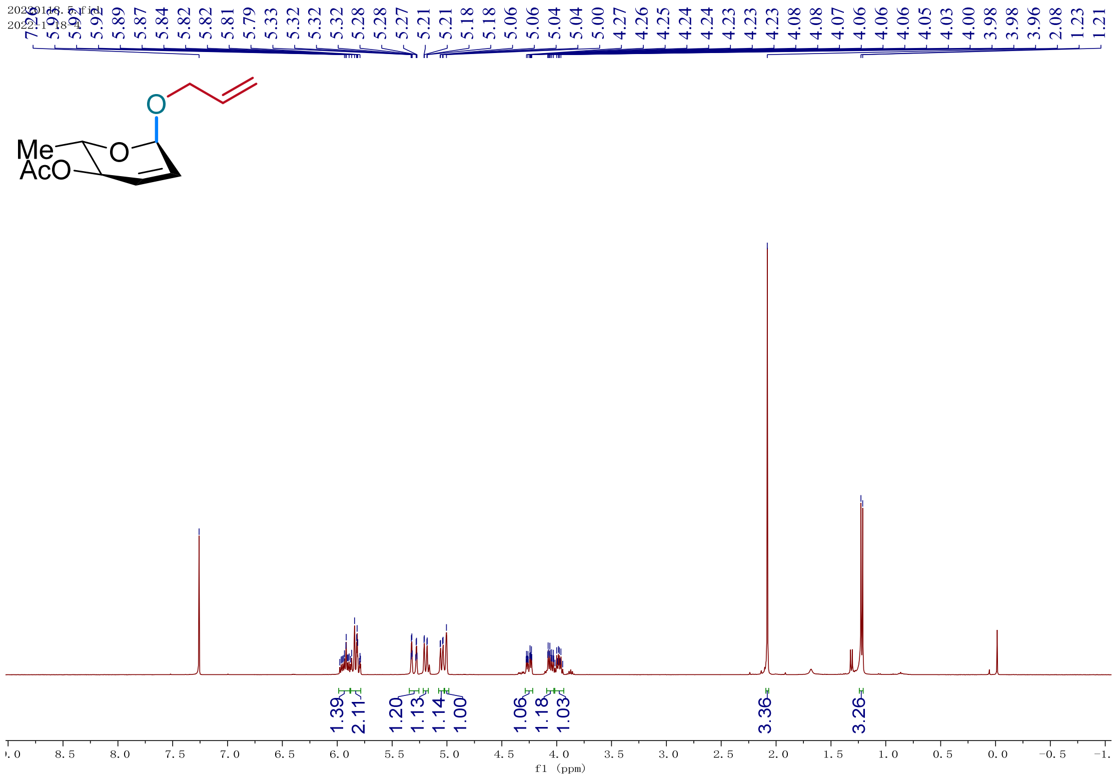

^{13}C NMR of 7b

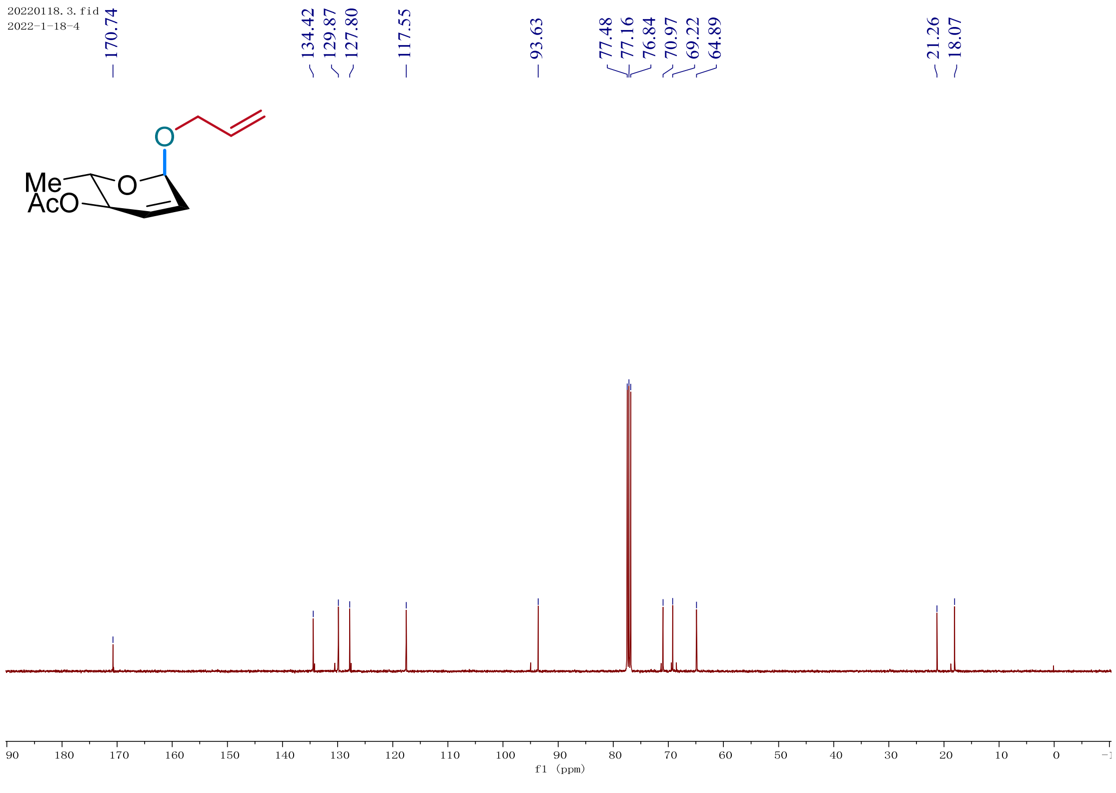

^{1}H NMR of 7c

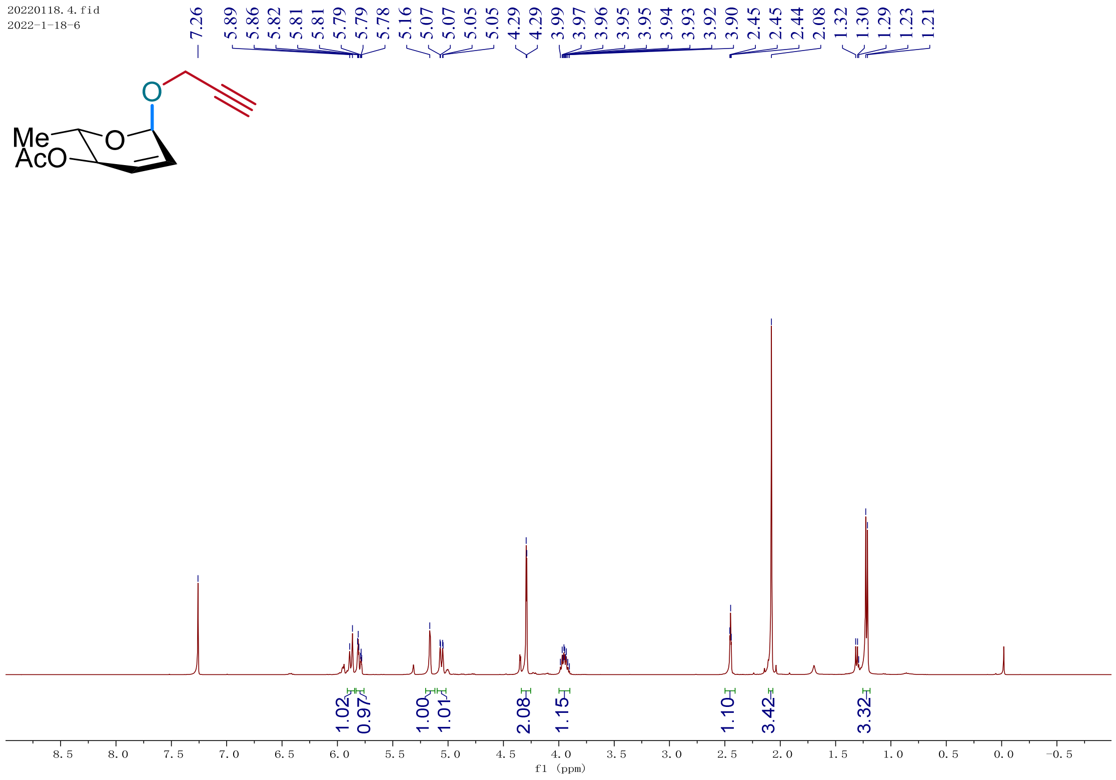

^{13}C NMR of 7c

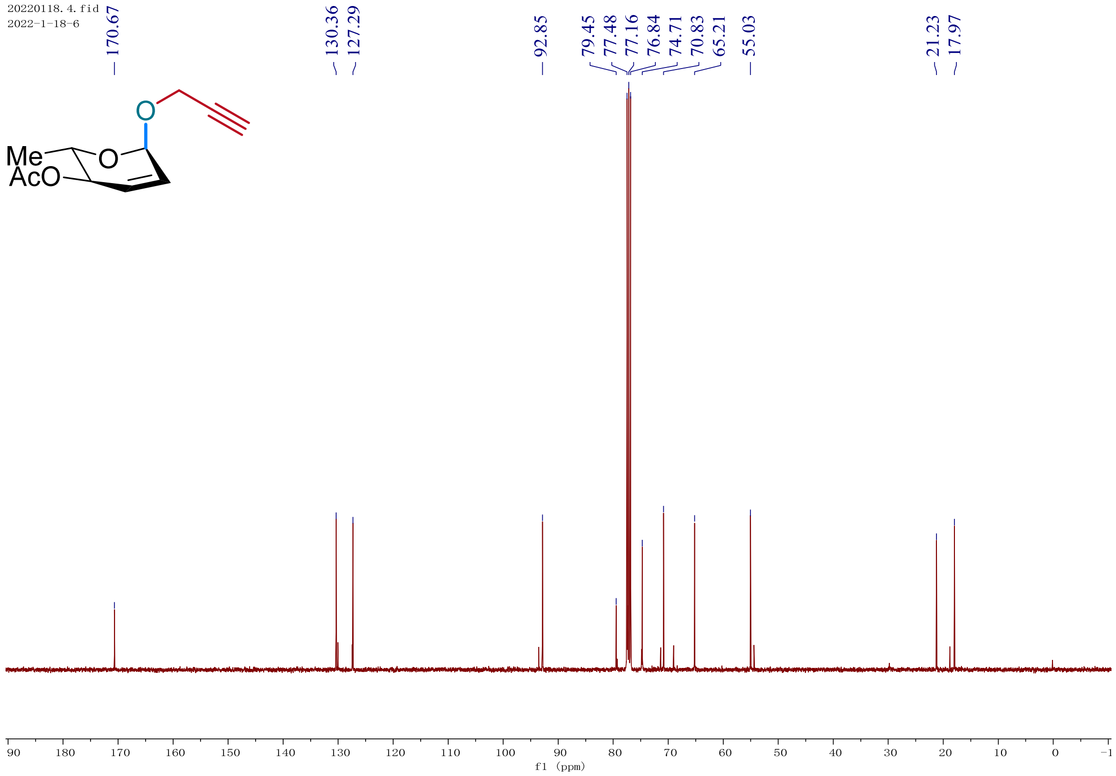

^{1}H NMR of 7d

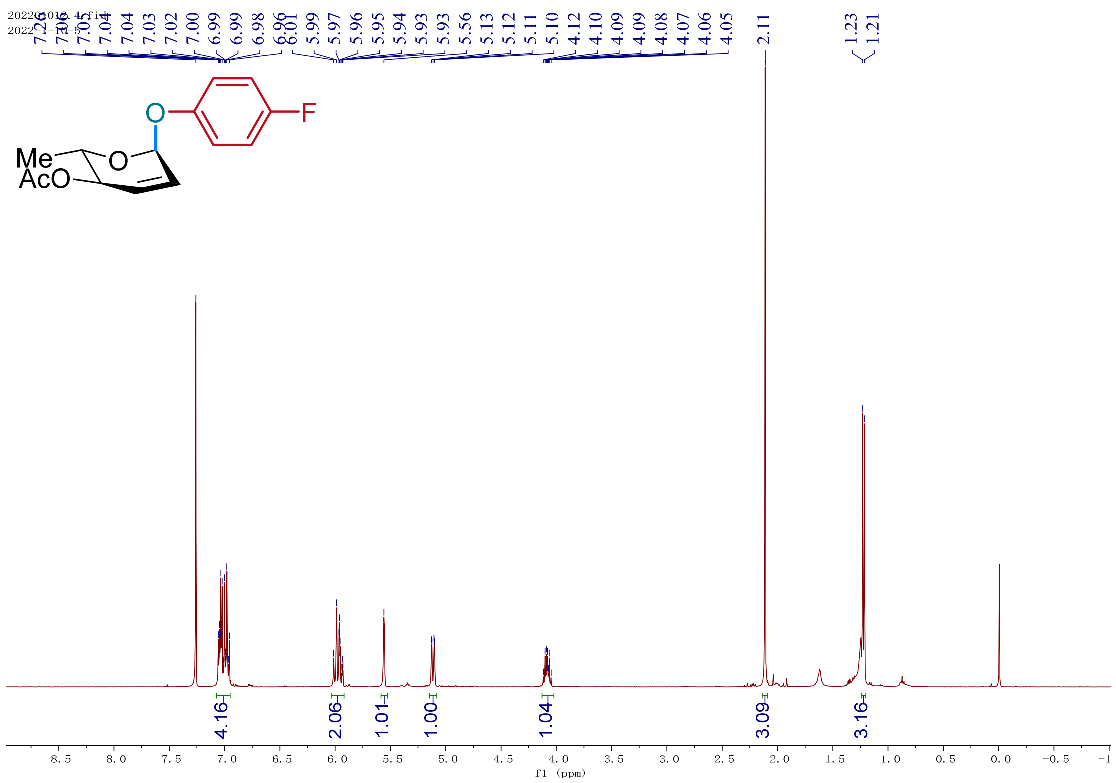

^{13}C NMR of 7d

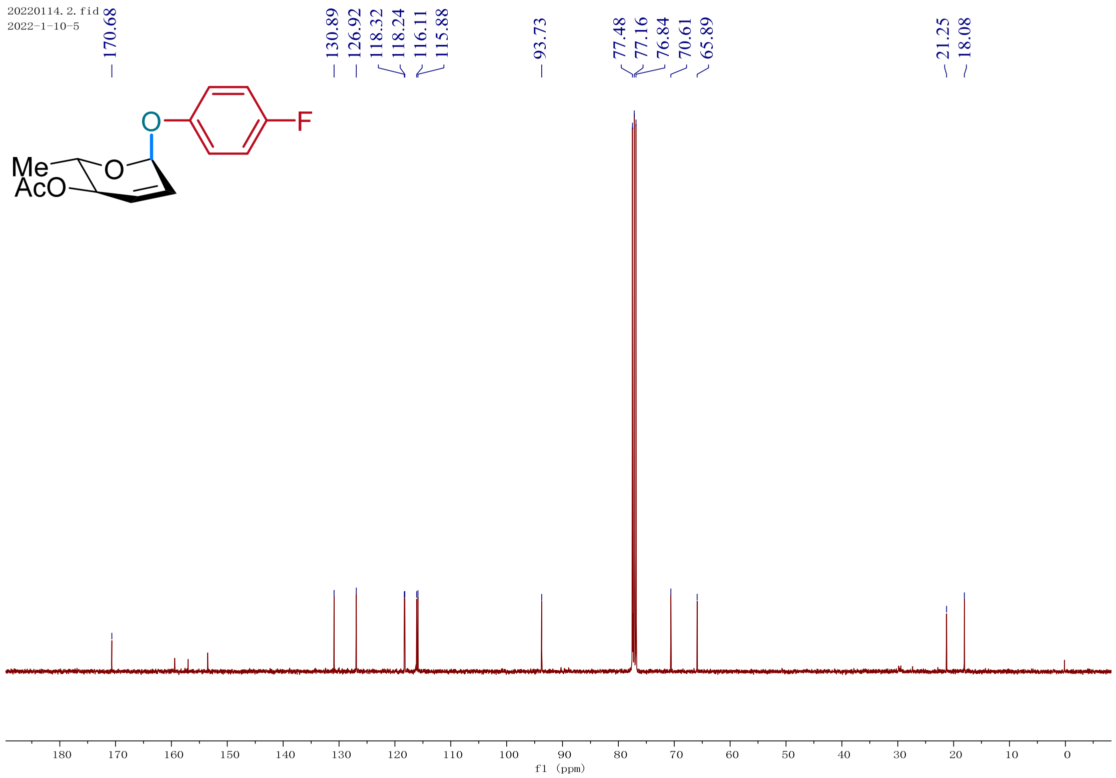

^{1}H NMR of 8a

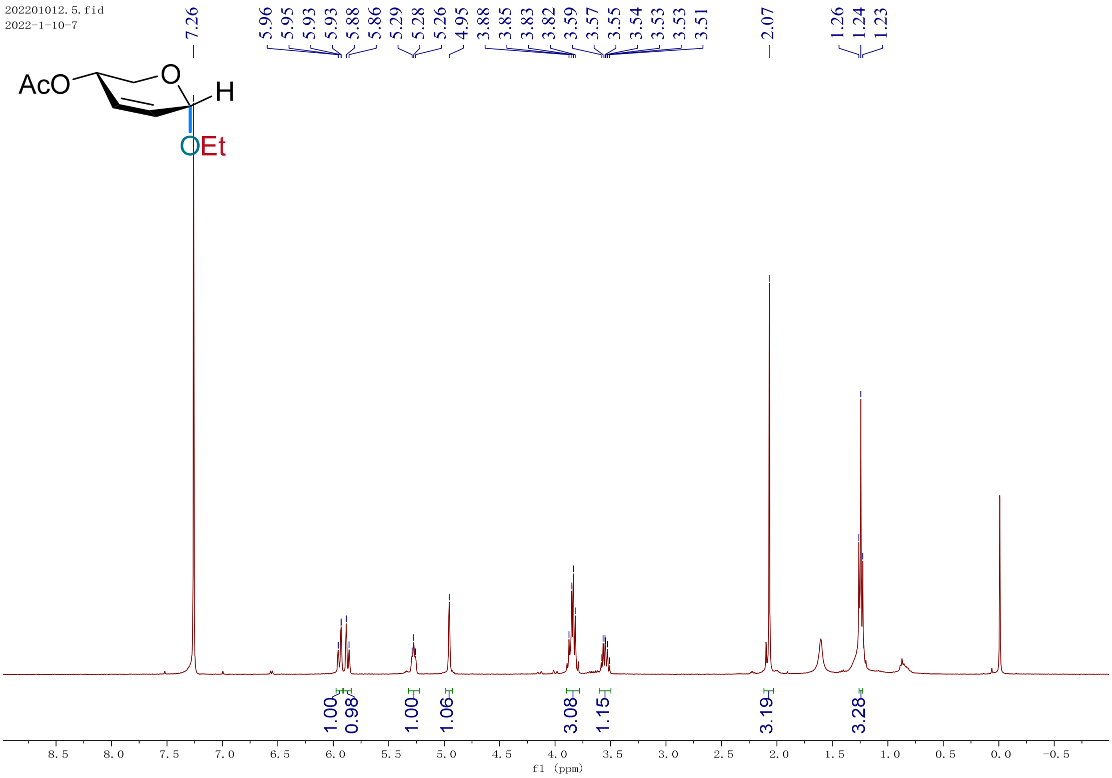

^{13}C NMR of 8a

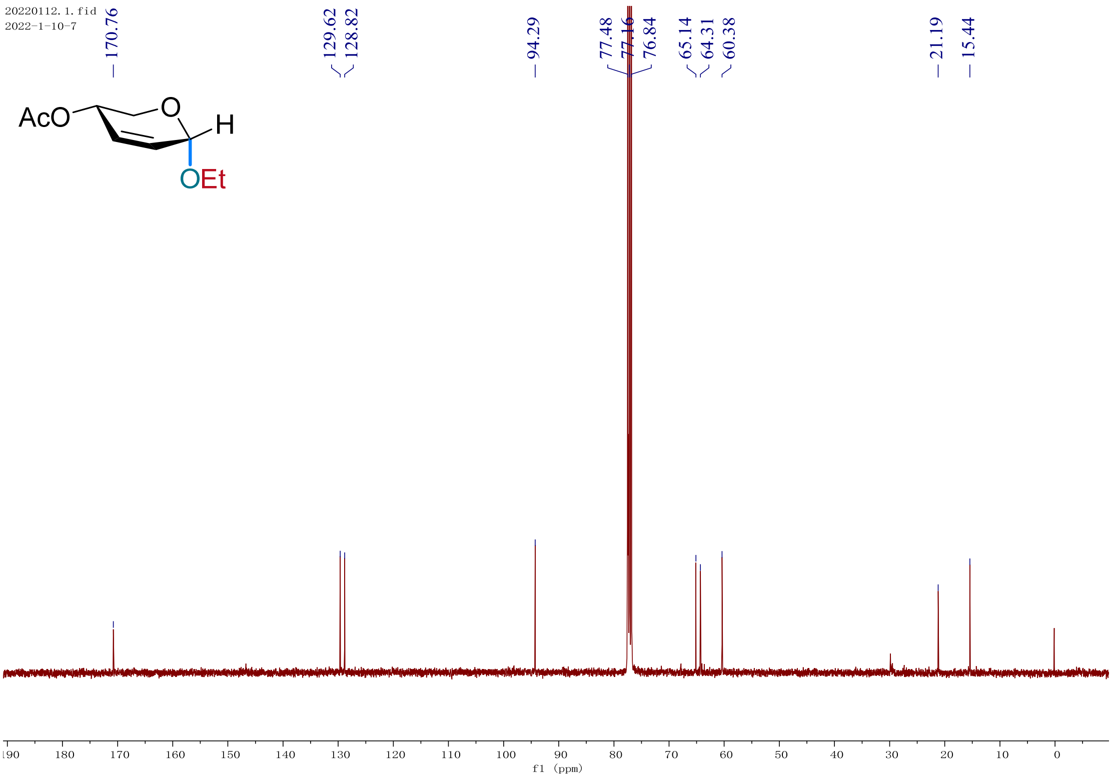

^{1}H NMR of 8b

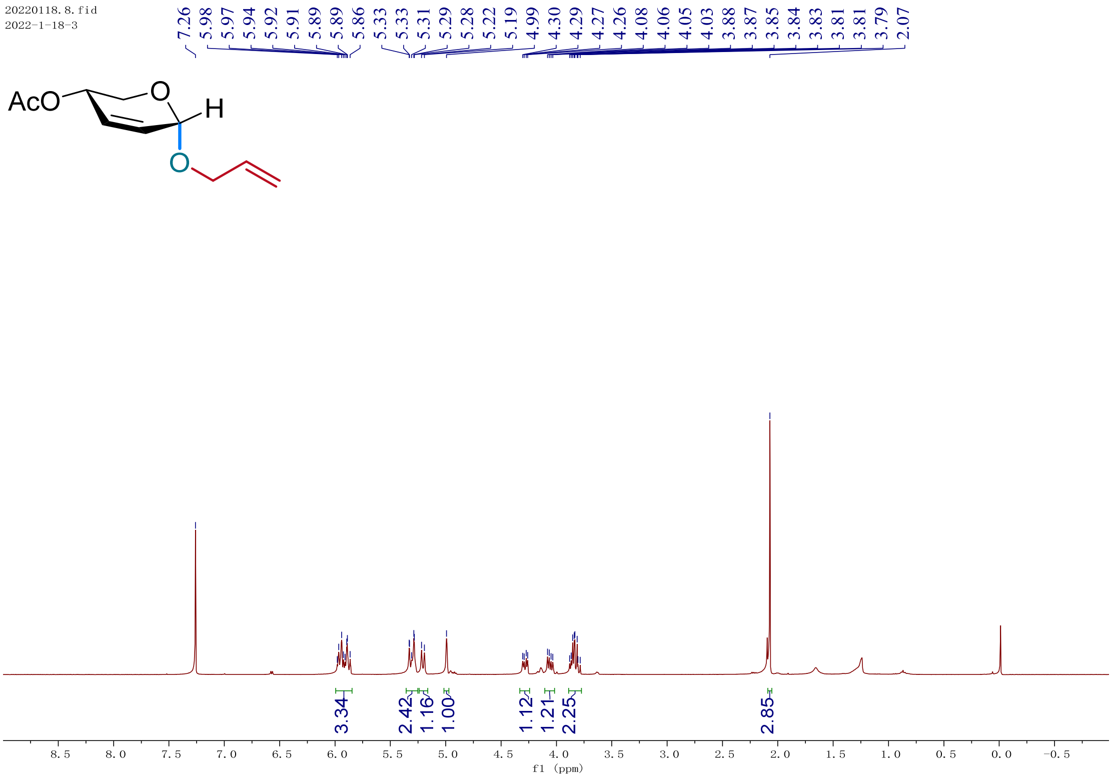

^{13}C NMR of 8b

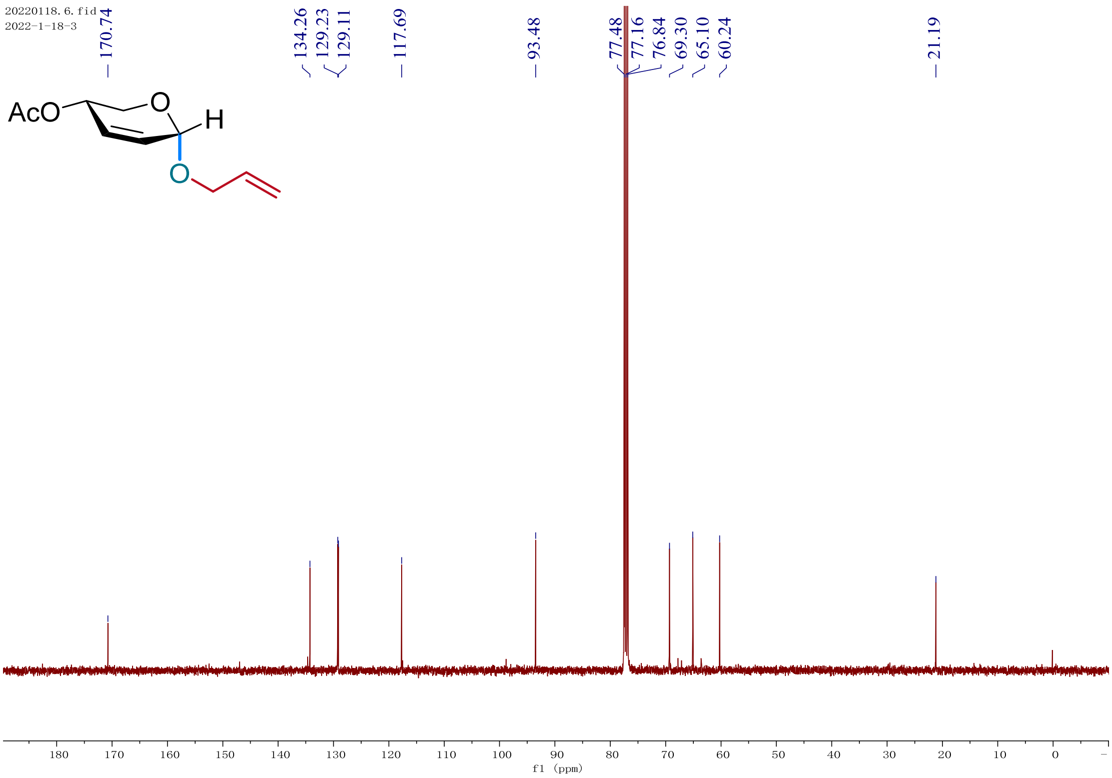

^{1}H NMR of 8c

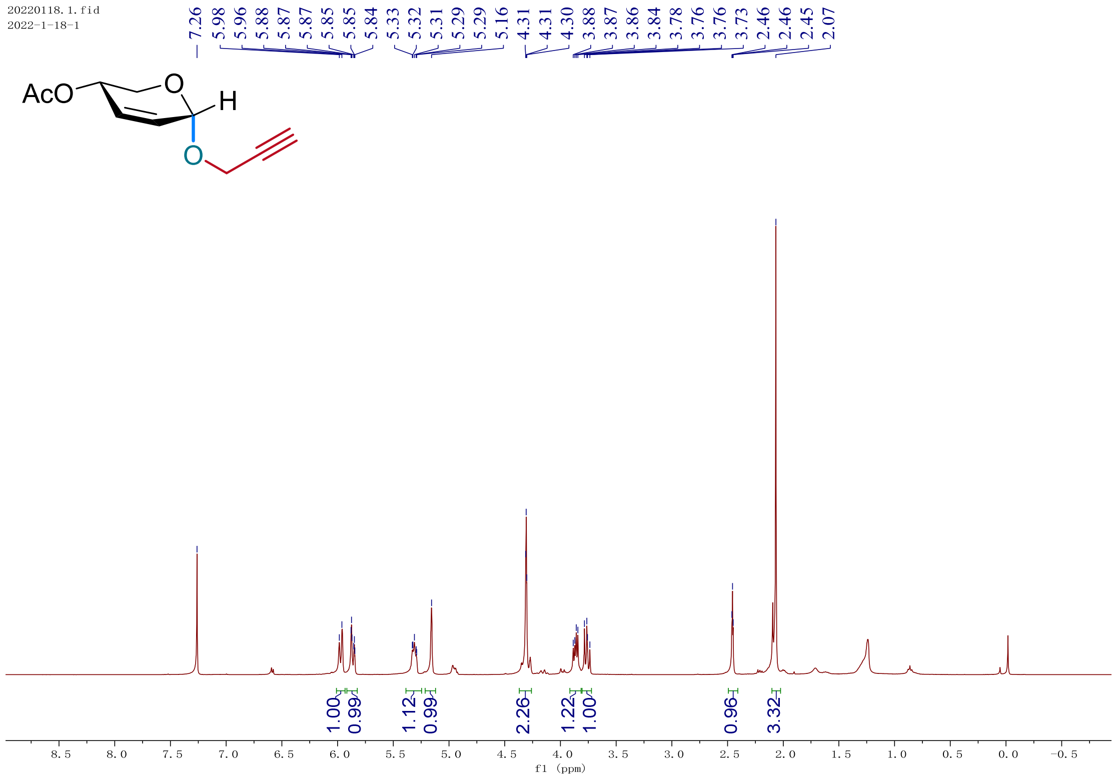

^{13}C NMR of 8c

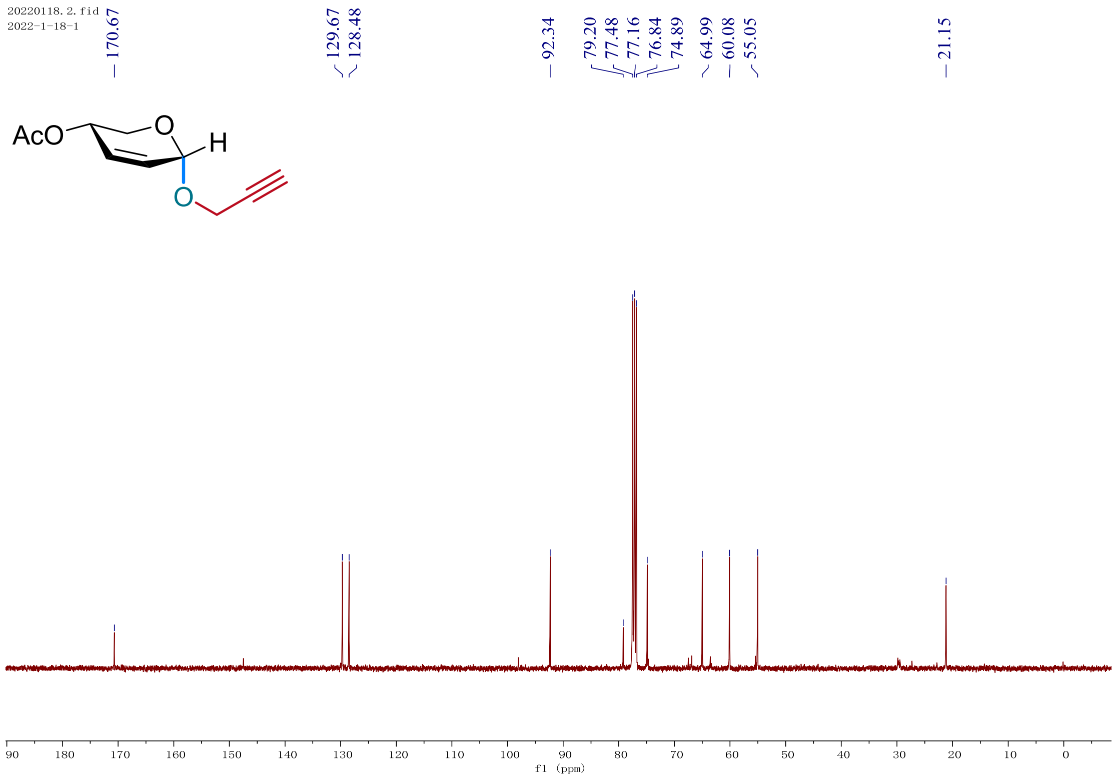

^{1}H NMR of 8d

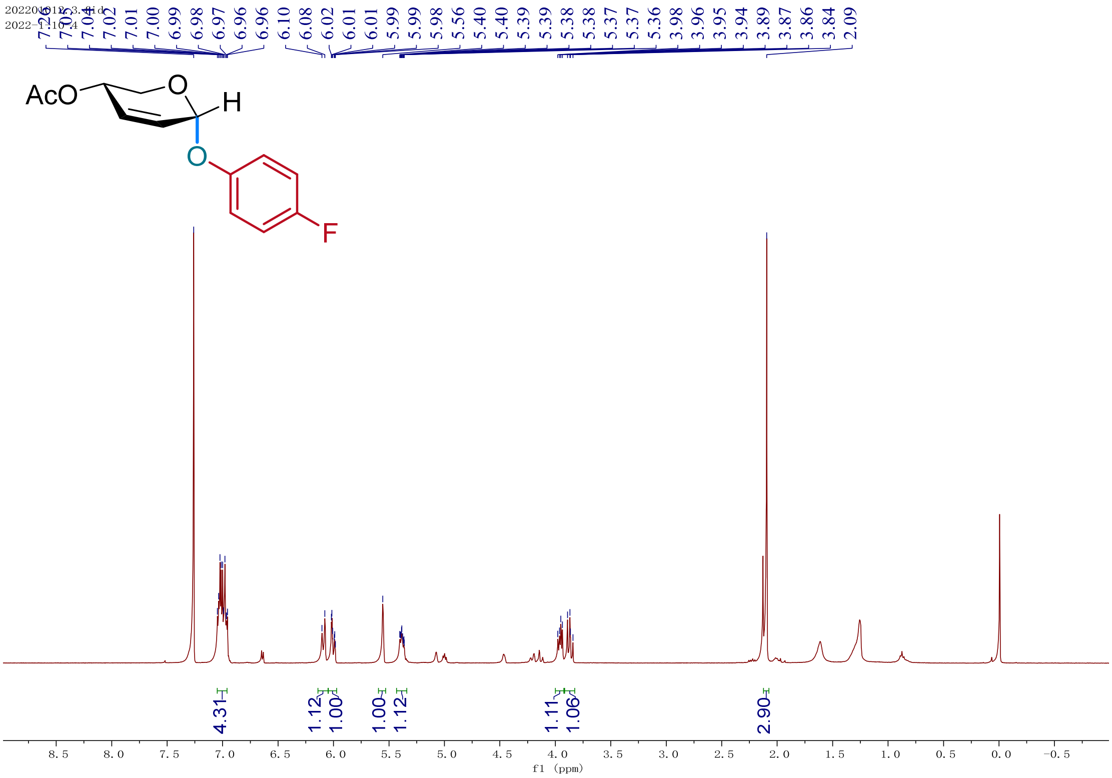

^{13}C NMR of 8d

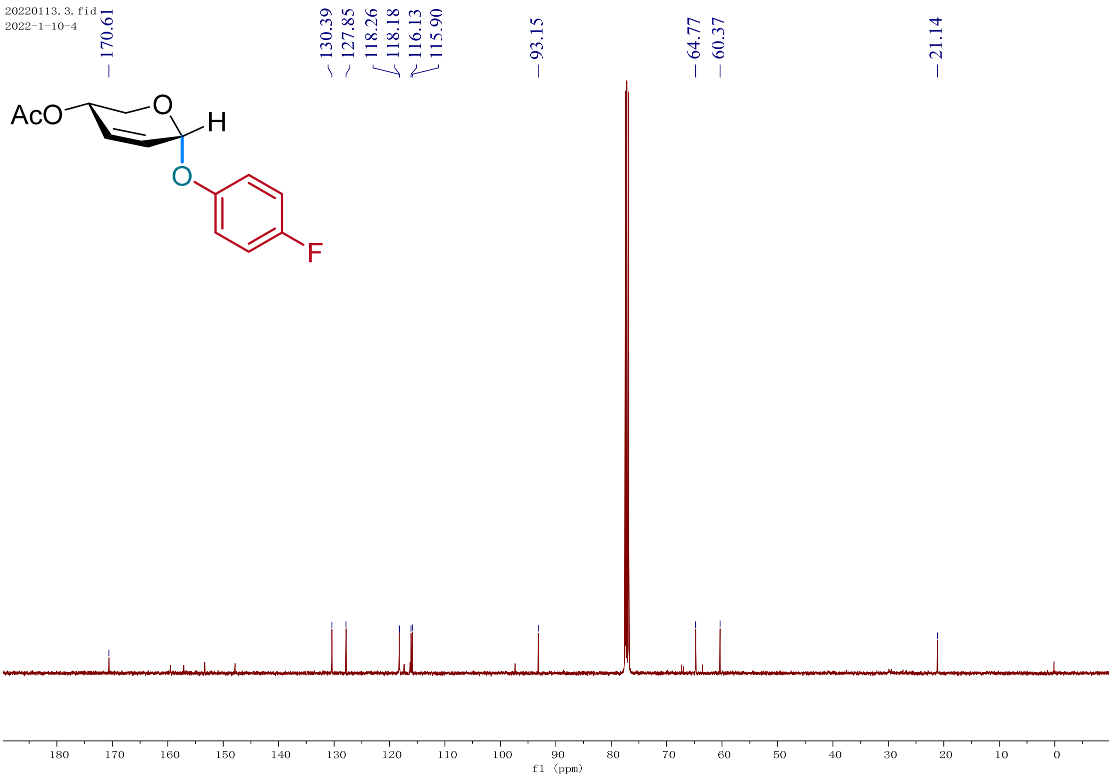

^{1}H NMR of 9a

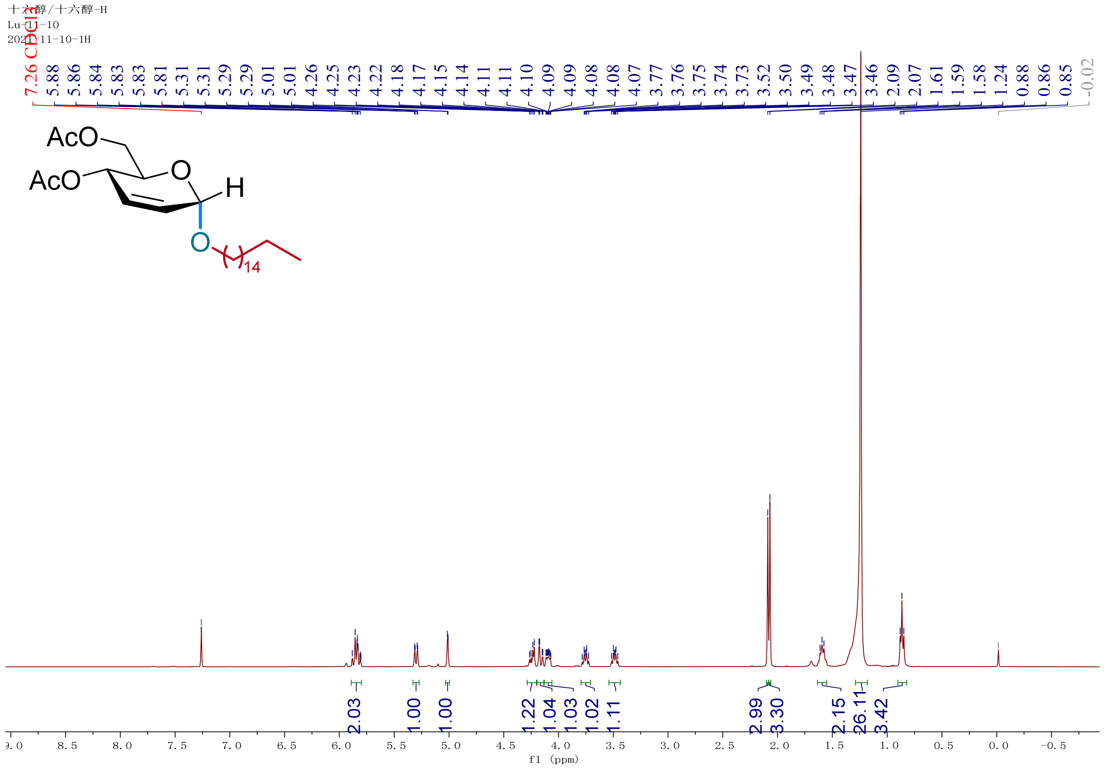

^{13}C NMR of 9a

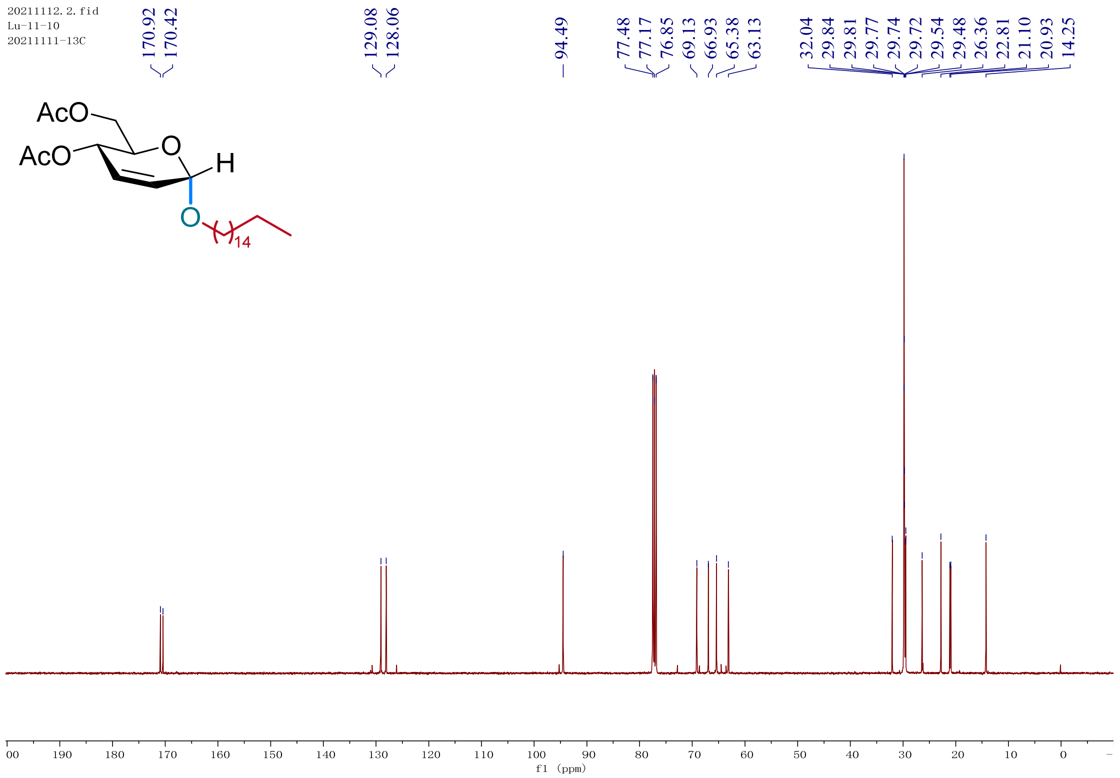

^{1}H NMR of 9b

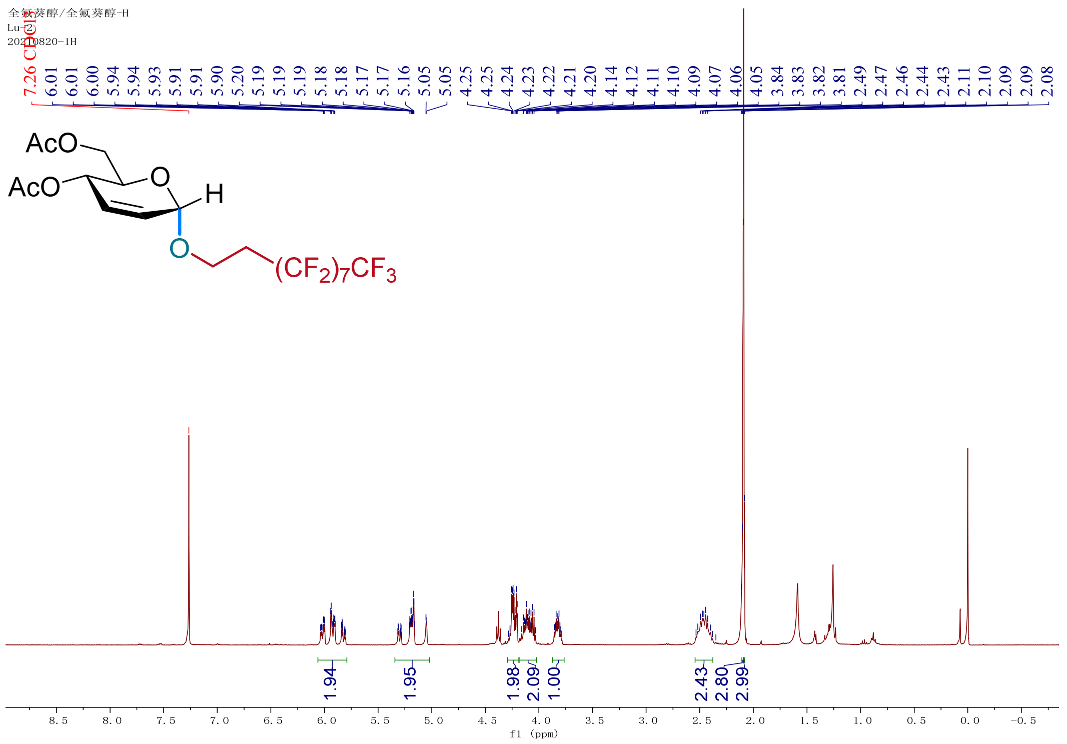

^{13}C NMR of 9b

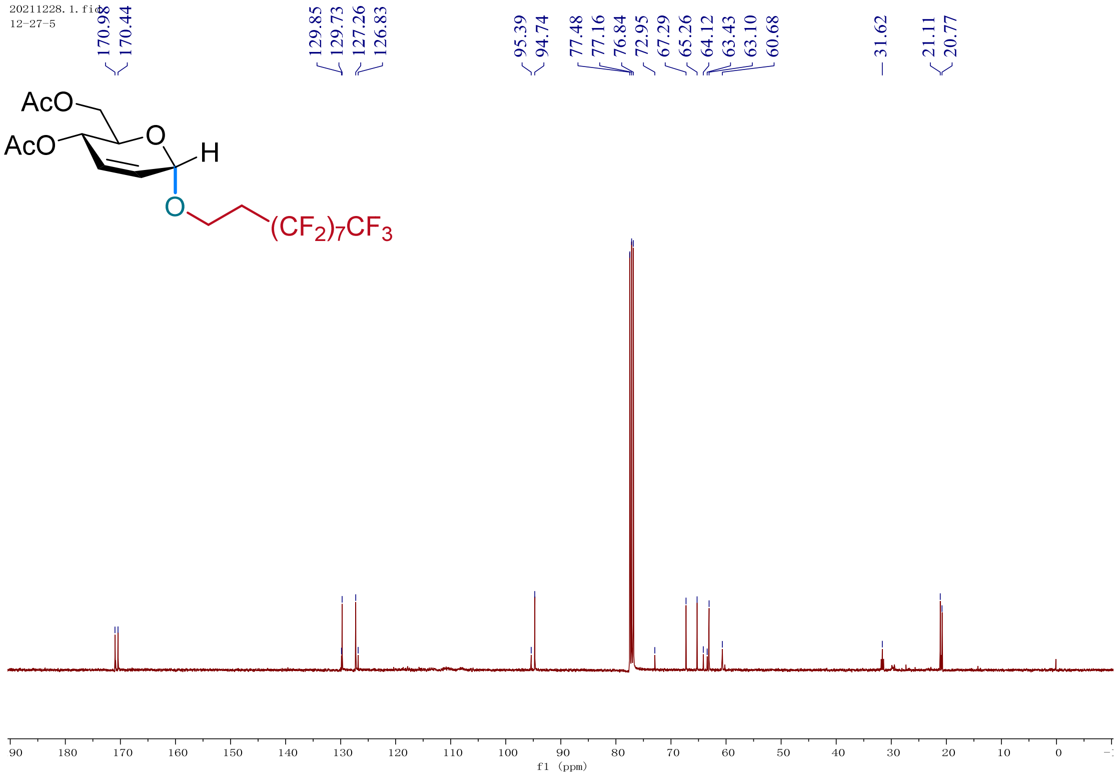

^{1}H NMR of 9c

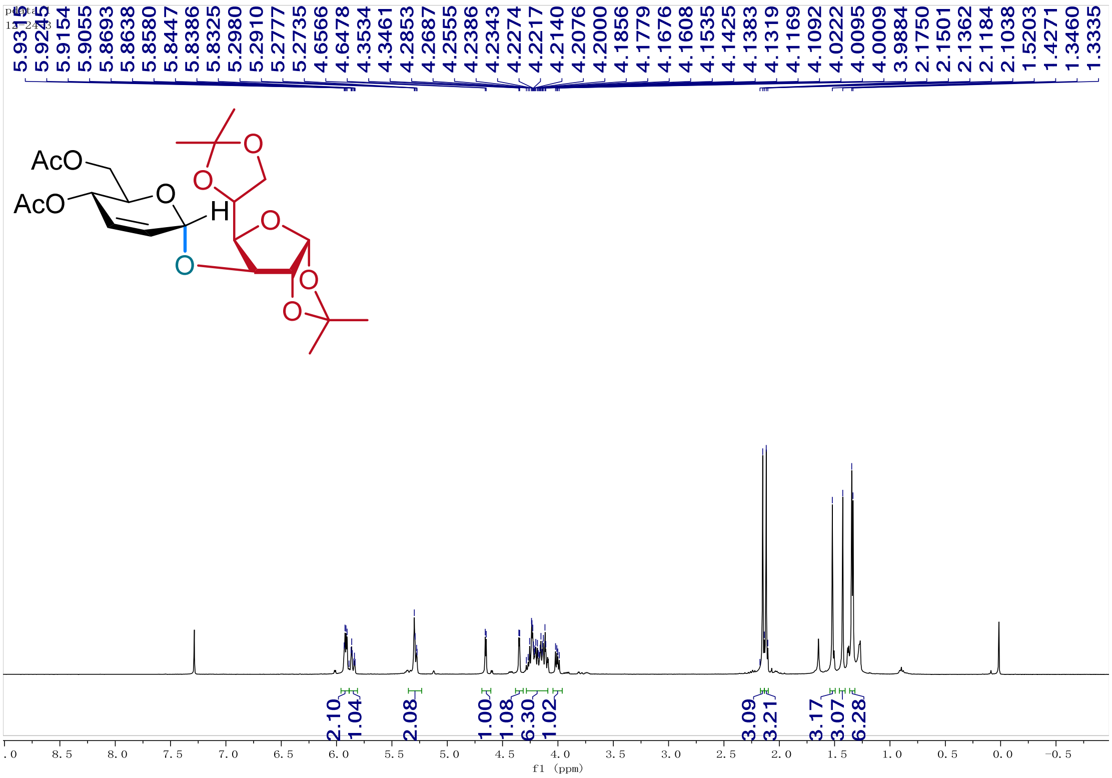

^{13}C NMR of 9c

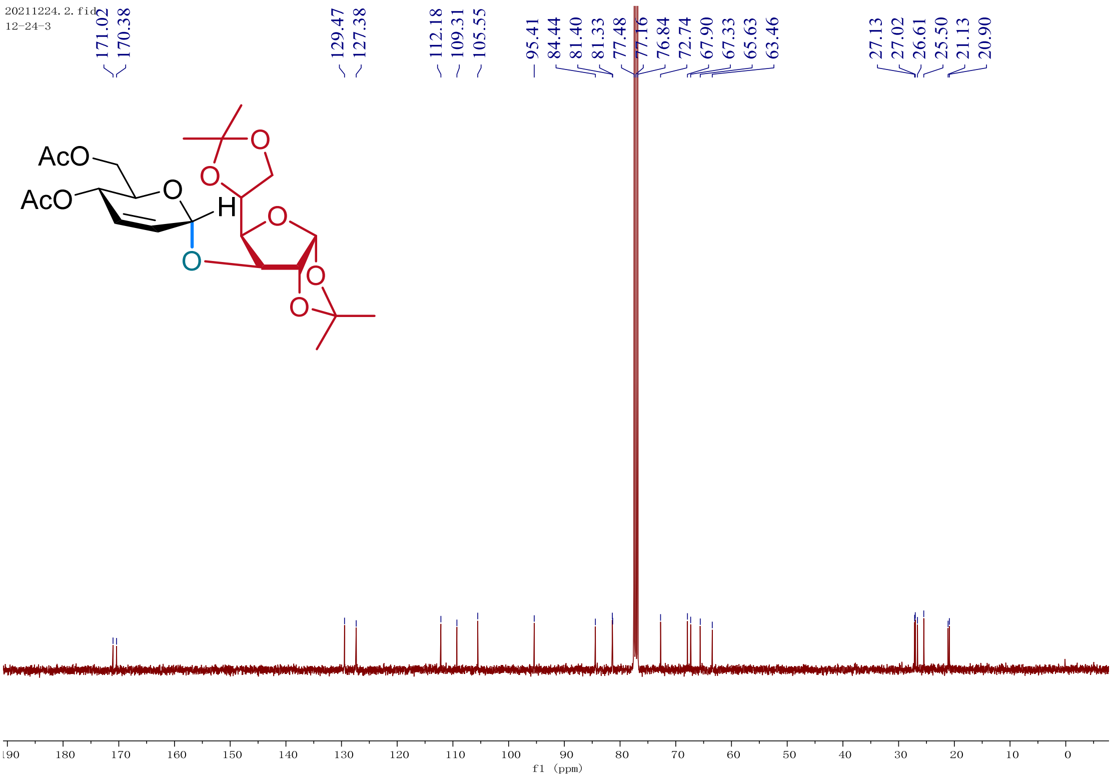

^{1}H NMR of 9d

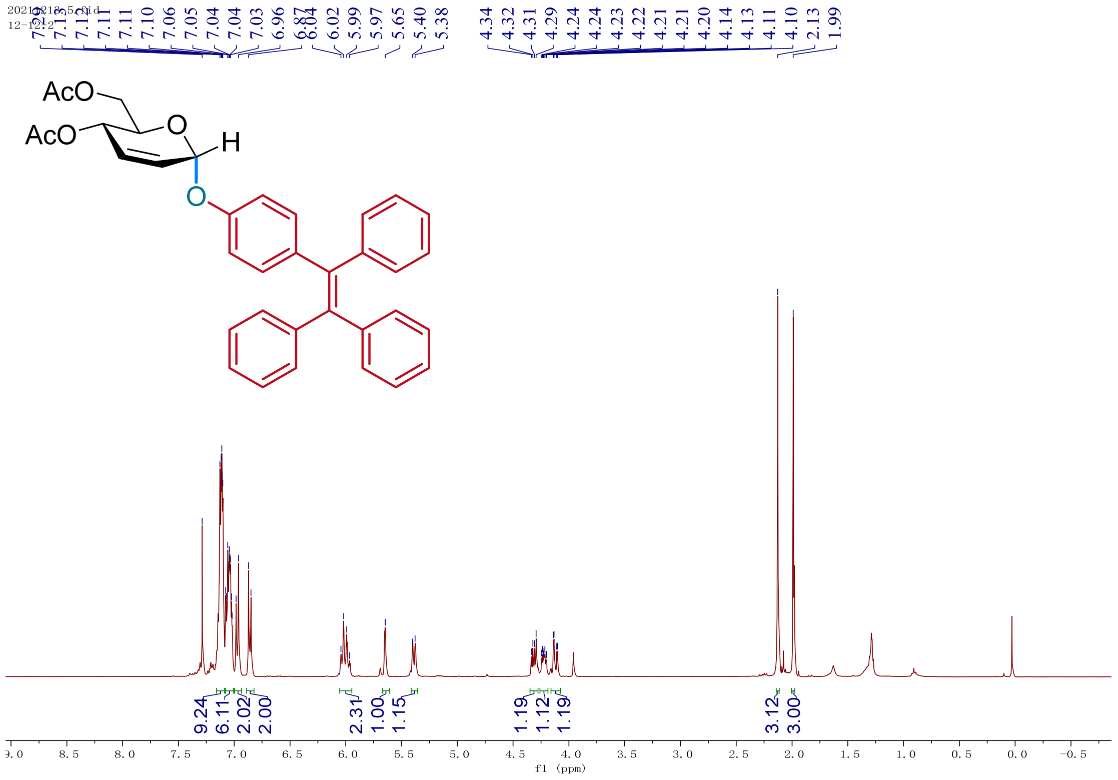

^{13}C NMR of 9d

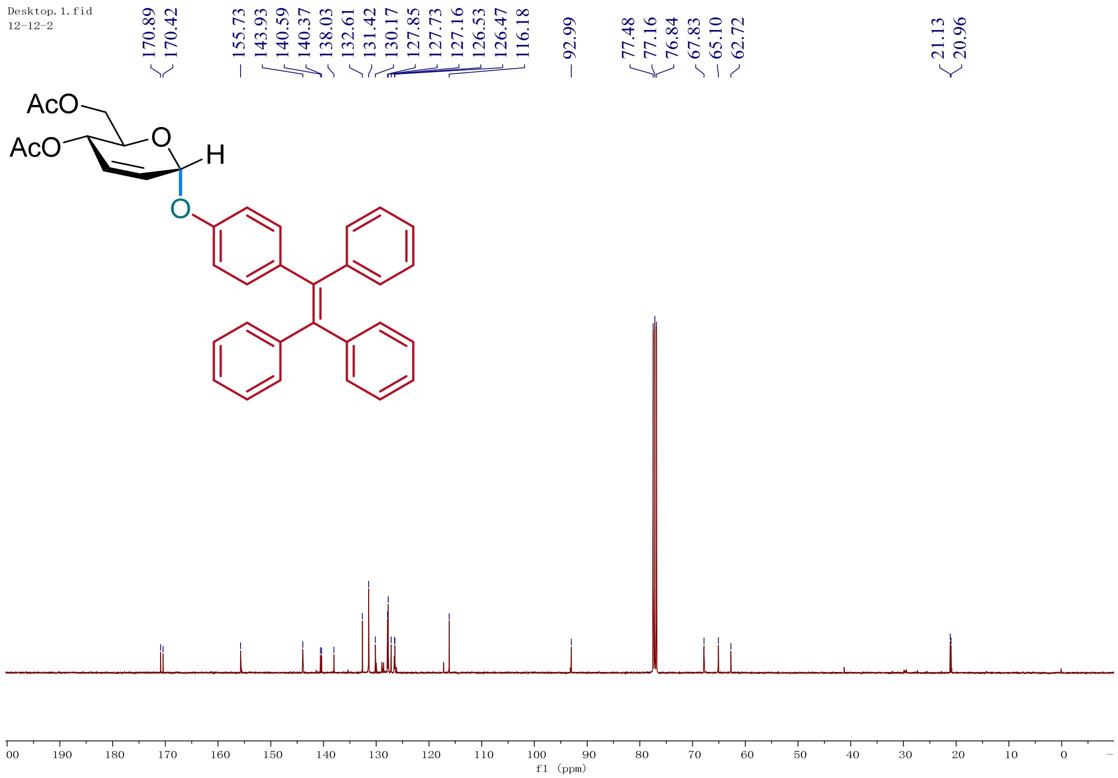

2D NOESY NMR of 9d

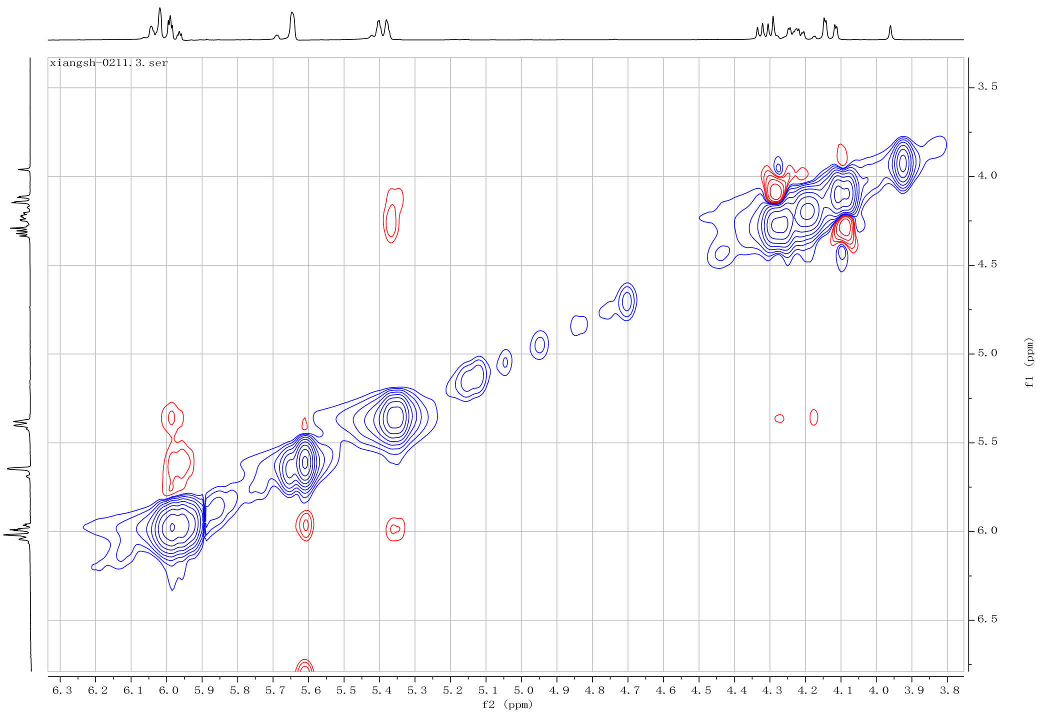

^{1}H NMR of 9e

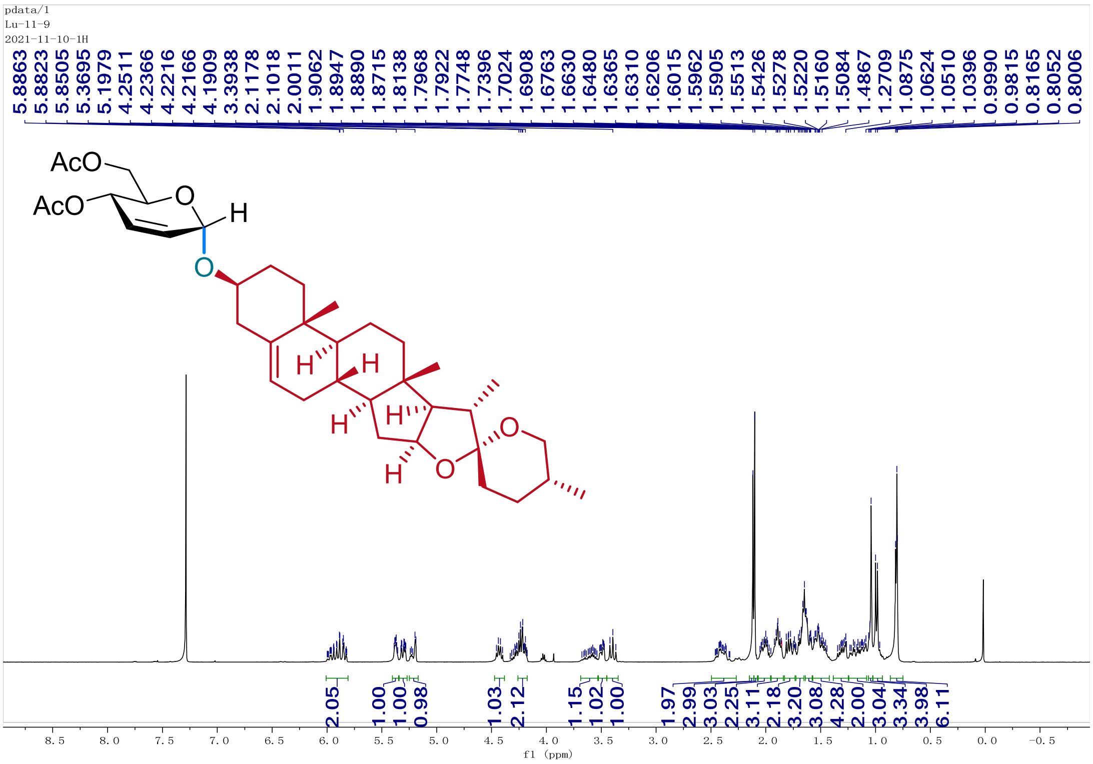

^{13}C NMR of **9e**

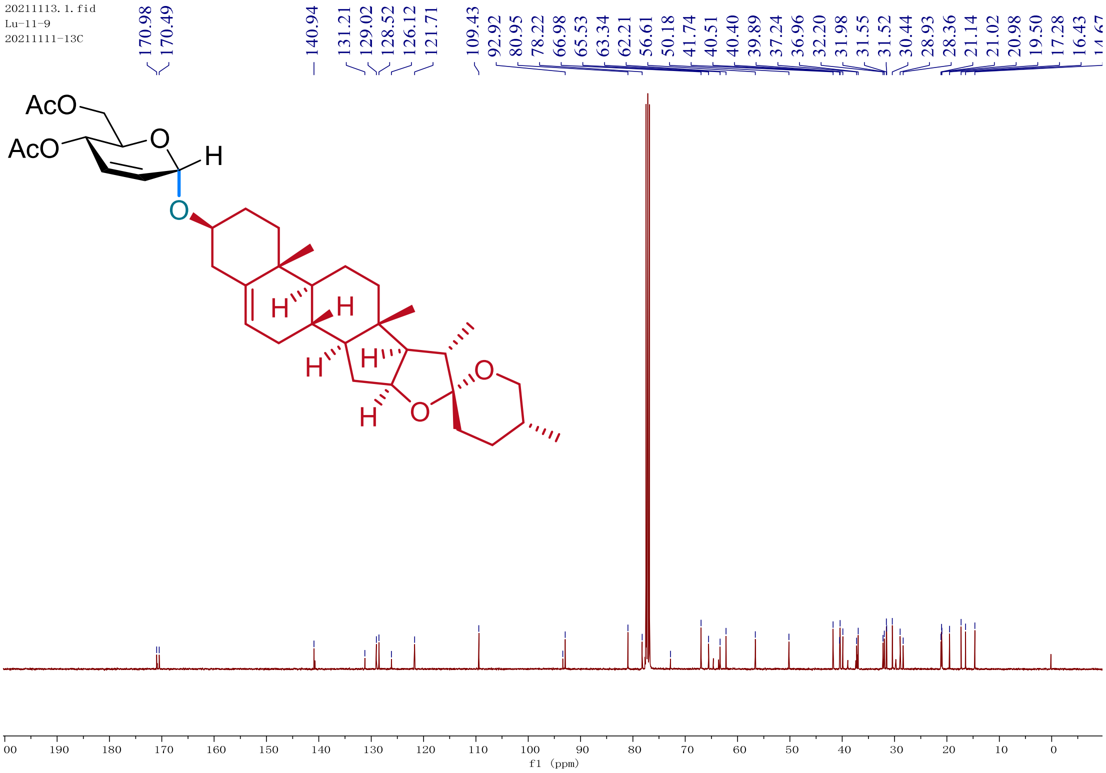

Section F: X-ray Crystal Structure Data

Table S1. Crystal data and structure refinement for **3a**.

**Compound No.**	**3a**
Empirical formula	$C_{12}H_{18}O_6$
Formula weight	258.26
Temperature/K	293
Crystal system	orthorhombic
Space group	$P2_12_12_1$
a/Å	5.3814(2)
b/Å	14.8428(10)
c/Å	16.6450(10)
α/°	90
β/°	90
γ/°	90
Volume/Å^3	1329.52(13)
Z	4
ρ_{calc}g/cm^3	1.290
μ/mm^{-1}	0.104
F(000)	552.0
Crystal size/mm^3	0.26 × 0.24 × 0.23
Radiation	Mo Kα (λ = 0.71073)
2Θ range for data collection/°	4.894 to 58.894
Index ranges	$-3 \le h \le 6$, $-19 \le k \le 12$, $-14 \le l \le 21$
Reflections collected	5034
Independent reflections	3088 [R_{int} = 0.0659, R_{sigma} = 0.0951]
Data/restraints/parameters	3088/0/166
Goodness-of-fit on F^2	0.979
Final R indexes [I>=2σ (I)]	R_1 = 0.0528, wR_2 = 0.1148
Final R indexes [all data]	R_1 = 0.0938, wR_2 = 0.1358
Largest diff. peak/hole / e Å^{-3}	0.13/-0.17
Flack parameter	2.0

Table S2. Crystal data and structure refinement for **3aa**.

**Compound No.**	**3aa**
Empirical formula	$C_{11}H_{10}NOF_{0.14}$
Formula weight	174.91
Temperature/K	293(2)
Crystal system	monoclinic
Space group	C2
a/Å	17.770(4)
b/Å	5.1918(8)
c/Å	18.291(3)
α/°	90
β/°	105.39(2)
γ/°	90
Volume/Å^3	1627.0(5)
Z	7
ρ_{calc}g/cm^3	1.250
μ/mm^{-1}	0.083
F(000)	646.0
Crystal size/mm^3	0.3 × 0.2 × 0.1
Radiation	Mo Kα (λ = 0.71073)
2Θ range for data collection/°	6.932 to 58.414
Index ranges	-13 ≤ h ≤ 24, -4 ≤ k ≤ 6, -24 ≤ l ≤ 24
Reflections collected	3816
Independent reflections	2629 [R_{int} = 0.0189, R_{sigma} = 0.0425]
Data/restraints/parameters	2629/1/210
Goodness-of-fit on F^2	1.071
Final R indexes [I>=2σ (I)]	R_1 = 0.0598, wR_2 = 0.1016
Final R indexes [all data]	R_1 = 0.1125, wR_2 = 0.1223
Largest diff. peak/hole / e Å^{-3}	0.15/-0.13
Flack parameter	-1.0(9)

Table S3. Crystal data and structure refinement for **3ab**.

**Compound No.**	**3ab**
Empirical formula	$C_8H_{8.5}N_{0.5}O_4$
Formula weight	175.65
Temperature/K	293(2)
Crystal system	monoclinic
Space group	$P2_1$
a/Å	10.7585(8)
b/Å	7.0930(3)
c/Å	12.2730(9)
α/°	90
β/°	113.230(9)
γ/°	90
Volume/Å^3	860.63(11)
Z	4
ρ_{calc}g/cm^3	1.356
μ/mm^{-1}	0.110
F(000)	368.0
Crystal size/mm^3	0.3 × 0.2 × 0.1
Radiation	Mo Kα (λ = 0.71073)
2Θ range for data collection/°	4.12 to 59.22
Index ranges	$-13 \leq h \leq 12$, $-9 \leq k \leq 9$, $-15 \leq l \leq 15$
Reflections collected	5178
Independent reflections	3889 [R_{int} = 0.0242, R_{sigma} = 0.0569]
Data/restraints/parameters	3889/1/228
Goodness-of-fit on F^2	1.013
Final R indexes [I>=2σ (I)]	R_1 = 0.0536, wR_2 = 0.1223
Final R indexes [all data]	R_1 = 0.0831, wR_2 = 0.1459
Largest diff. peak/hole / e Å^{-3}	0.21/-0.26
Flack parameter	-1.2(10)

Table S4. Crystal data and structure refinement for **4d**.

**Compound No.**	**4d**
Empirical formula	$C_{8.5}H_{10}O_{2.5}S_{0.5}$
Formula weight	168.19
Temperature/K	293
Crystal system	monoclinic
Space group	$P2_1$
a/Å	11.9973(11)
b/Å	5.4571(3)
c/Å	14.0428(13)
α/°	90
β/°	108.328(9)
γ/°	90
Volume/Å^3	872.75(13)
Z	4
ρ_{calc}g/cm^3	1.280
μ/mm^{-1}	0.207
F(000)	356.0
Crystal size/mm^3	0.3 × 0.2 × 0.1
Radiation	Mo Kα (λ = 0.71073)
2Θ range for data collection/°	3.906 to 58.256
Index ranges	$-11 \leq h \leq 15$, $-6 \leq k \leq 7$, $-18 \leq l \leq 17$
Reflections collected	5188
Independent reflections	3706 [R_{int} = 0.0309, R_{sigma} = 0.0701]
Data/restraints/parameters	3706/1/211
Goodness-of-fit on F^2	0.961
Final R indexes [I>=2σ (I)]	R_1 = 0.0481, wR_2 = 0.0907
Final R indexes [all data]	R_1 = 0.0783, wR_2 = 0.1101
Largest diff. peak/hole / e Å^{-3}	0.16/-0.20
Flack parameter	0.06(9)

Table S5. Crystal data and structure refinement for **4e'**.

**Compound No.**	**4e'**
Empirical formula	$C_{2.62}H_{3.08}O_{0.77}S_{0.15}$
Formula weight	51.75
Temperature/K	293(2)
Crystal system	orthorhombic
Space group	$P2_12_12_1$
a/Å	8.3041(6)
b/Å	8.5154(6)
c/Å	25.5741(19)
α/°	90
β/°	90
γ/°	90
Volume/Å^3	1808.4(2)
Z	26
ρ_{calc}g/cm^3	1.236
μ/mm^{-1}	0.200
F(000)	712.0
Crystal size/mm^3	0.3 × 0.2 × 0.2
Radiation	Mo Kα (λ = 0.71073)
2Θ range for data collection/°	5.042 to 58.512
Index ranges	-10 ≤ h ≤ 7, -10 ≤ k ≤ 10, -34 ≤ l ≤ 31
Reflections collected	6611
Independent reflections	4133 [R_{int} = 0.0630, R_{sigma} = 0.1081]
Data/restraints/parameters	4133/0/211
Goodness-of-fit on F^2	0.892
Final R indexes [I>=2σ (I)]	R_1 = 0.0603, wR_2 = 0.1403
Final R indexes [all data]	R_1 = 0.1083, wR_2 = 0.1638
Largest diff. peak/hole / e Å^{-3}	0.19/-0.19
Flack parameter	0.03(12)
